# tRNA-like Transcripts from the *NEAT1-MALAT1* Genomic Region Critically Influence Human Innate Immunity and Macrophage Functions

**DOI:** 10.3390/cells11243970

**Published:** 2022-12-08

**Authors:** Martina Gast, Vanasa Nageswaran, Andreas W. Kuss, Ana Tzvetkova, Xiaomin Wang, Liliana H. Mochmann, Pegah Ramezani Rad, Stefan Weiss, Stefan Simm, Tanja Zeller, Henry Voelzke, Wolfgang Hoffmann, Uwe Völker, Stefan B. Felix, Marcus Dörr, Antje Beling, Carsten Skurk, David-Manuel Leistner, Bernhard H. Rauch, Tetsuro Hirose, Bettina Heidecker, Karin Klingel, Shinichi Nakagawa, Wolfram C. Poller, Filip K. Swirski, Arash Haghikia, Wolfgang Poller

**Affiliations:** 1 Department of Cardiology, Campus Benjamin Franklin, Charité—Universitätsmedizin Berlin, Corporate Member of Freie Universität Berlin, Humboldt-Universität zu Berlin, Berlin Institute of Health, 12200 Berlin, Germany; 2 German Center for Cardiovascular Research (DZHK), Site Berlin, 12200 Berlin, Germany; 3 Institute for Chemistry and Biochemistry, Freie Universität Berlin, 12200 Berlin, Germany; 4 Department of Functional Genomics, Interfaculty Institute of Genetics and Functional Genomics, University Medicine Greifswald, 17475 Greifswald, Germany; 5 Institute of Bioinformatics, University Medicine Greifswald, 17475 Greifswald, Germany; 6 German Center for Cardiovascular Research (DZHK), Site Greifswald, 17487 Greifswald, Germany; 7 University Center of Cardiovascular Science, University Heart and Vascular Center, 20246 Hamburg, Germany; 8 German Center for Cardiovascular Research (DZHK), Site Hamburg/Lübeck/Kiel, 20246 Hamburg, Germany; 9 Institute for Community Medicine, University Medicine Greifswald, 17475 Greifswald, Germany; 10 Department of Cardiology, University Medicine Greifswald, 17475 Greifswald, Germany; 11 Institute for Biochemistry, Charité—Universitätsmedizin Berlin, Corporate Member of Freie Universität Berlin, Humboldt-Universität zu Berlin, Berlin Institute of Health, 10178 Berlin, Germany; 12 Berlin Institute of Health (BIH), 10178 Berlin, Germany; 13 Institute for Pharmacology, University Medicine Greifswald, 17487 Greifswald, Germany; 14 Department Human Medicine, Section Pharmacology and Toxicology, Carl von Ossietzky Universität, 26129 Oldenburg, Germany; 15 Graduate School of Frontier Biosciences, Osaka University, 1-3 Yamadaoka, Suita 565-0871, Japan; 16 Institute for Pathology and Neuropathology, Department of Pathology, University Hospital Tübingen, 72076 Tübingen, Germany; 17 RNA Biology Laboratory, RIKEN Advanced Research Institute, Wako, Saitama 351-0198, Japan; 18 Faculty of Pharmaceutical Sciences, Hokkaido University, Sapporo 060-0812, Japan; 19 Center for Systems Biology, Massachusetts General Hospital, Harvard Medical School, Boston, MA 02114, USA; 20 Cardiovascular Research Institute, Icahn School of Medicine at Mount Sinai, New York, NY 10029, USA; 21 Berlin-Brandenburg Center for Regenerative Therapies (BCRT), Charité—Universitätsmedizin Berlin, Corporate Member of Freie Universität Berlin, Humboldt-Universität zu Berlin, Berlin Institute of Health, 13353 Berlin, Germany

**Keywords:** immunology, innate immunity, immunogenetics, noncoding genome, tRNA biology, evolutionary genetics

## Abstract

The evolutionary conserved *NEAT1-MALAT1* gene cluster generates large noncoding transcripts remaining nuclear, while tRNA-like transcripts (mascRNA, menRNA) enzymatically generated from these precursors translocate to the cytosol. Whereas functions have been assigned to the nuclear transcripts, data on biological functions of the small cytosolic transcripts are sparse. We previously found *NEAT1^−/−^* and *MALAT1^−/−^* mice to display massive atherosclerosis and vascular inflammation. Here, employing selective targeted disruption of menRNA or mascRNA, we investigate the tRNA-like molecules as critical components of innate immunity. CRISPR-generated human ΔmascRNA and ΔmenRNA monocytes/macrophages display defective innate immune sensing, loss of cytokine control, imbalance of growth/angiogenic factor expression impacting upon angiogenesis, and altered cell–cell interaction systems. Antiviral response, foam cell formation/oxLDL uptake, and M1/M2 polarization are defective in ΔmascRNA/ΔmenRNA macrophages, defining first biological functions of menRNA and describing new functions of mascRNA. menRNA and mascRNA represent novel components of innate immunity arising from the noncoding genome. They appear as prototypes of a new class of noncoding RNAs distinct from others (miRNAs, siRNAs) by biosynthetic pathway and intracellular kinetics. Their *NEAT1-MALAT1* region of origin appears as archetype of a functionally highly integrated RNA processing system.

## 1. Introduction

The evolutionary conserved *NEAT1-MALAT1* gene cluster encounters high interest in both cardiovascular medicine and oncology. In the cardiovascular field, we observed suppression of lncRNA *NEAT1* in circulating immune cells of post-myocardial infarction (MI) patients [1]. Mice lacking lncRNAs *NEAT1* [1] or *MALAT1* [2,3,4] displayed immune disturbances affecting monocyte-macrophage and T cell differentiation and rendering the immune system highly vulnerable to stress stimuli, thereby promoting the development of atherosclerosis. Uncontrolled inflammation is also a key driver of multiple other diseases [1,2,4,5,6].

**Figure 1:**
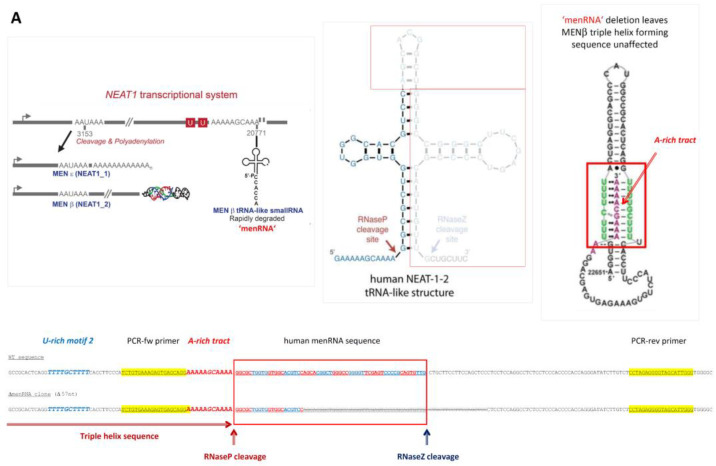

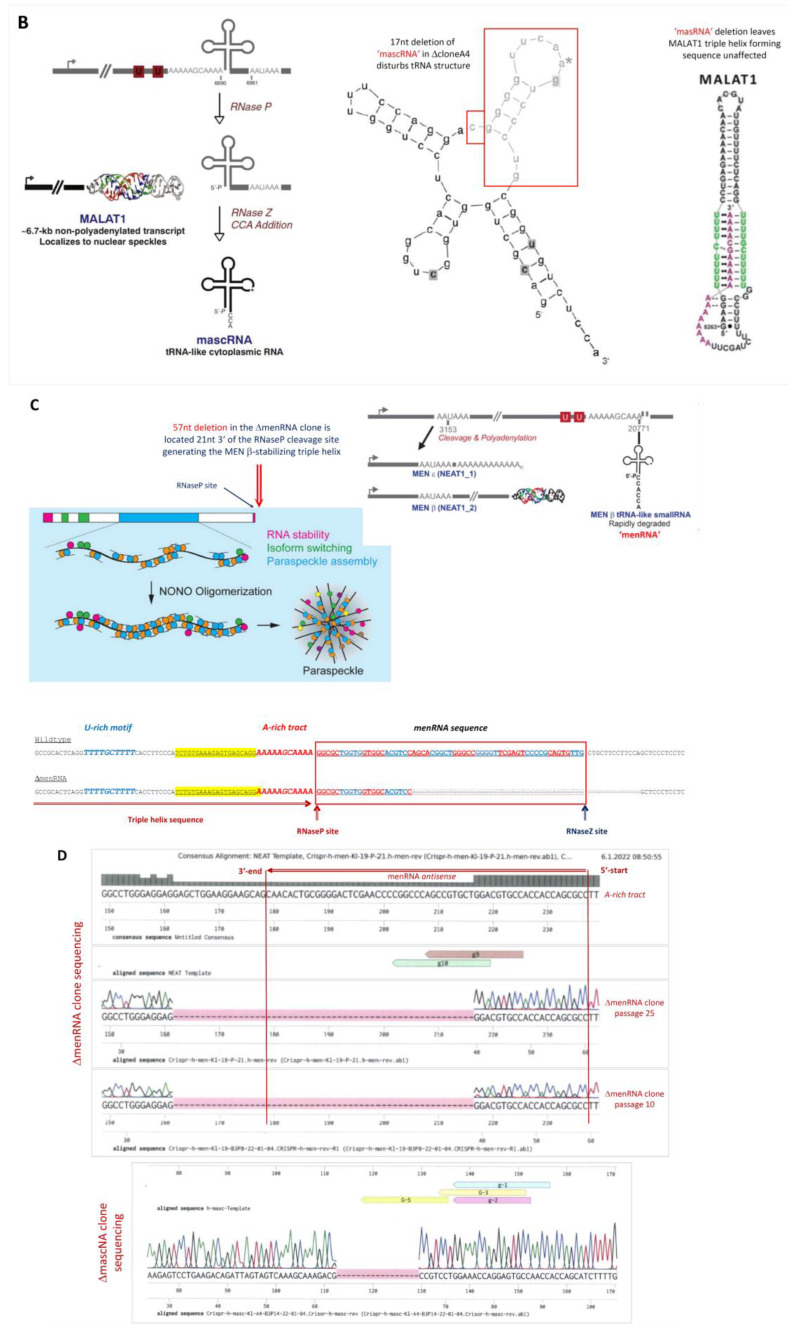

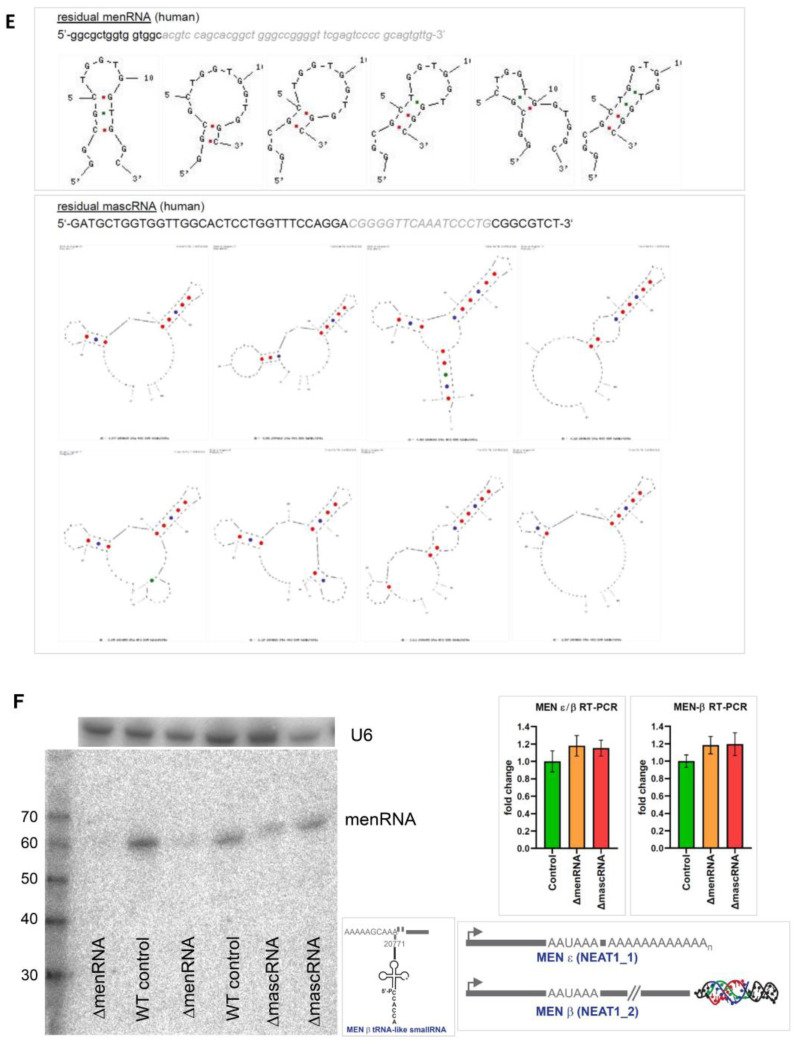

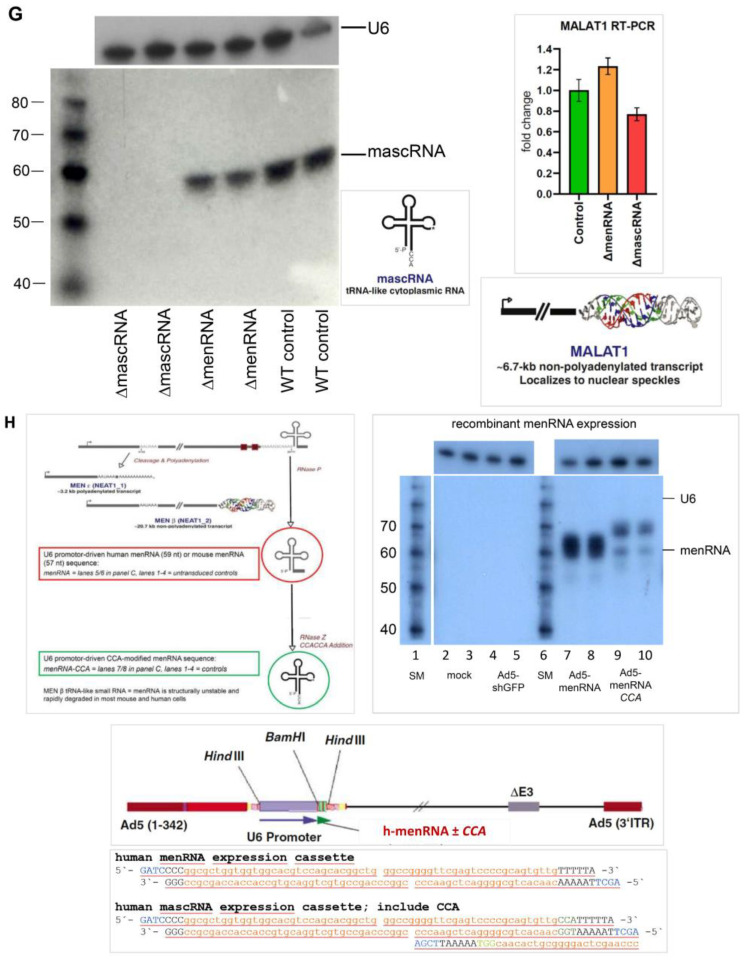
Targeted disruption of tRNA-like transcripts in the *NEAT1*-*MALAT1* gene cluster. Panel (**A**): The *NEAT1-MALAT1* gene cluster encodes two primary transcripts which are subsequently processed to transcripts of vastly different size. The *NEAT1* locus yields the 23 kb MEN-β (*NEAT1_1*) and 3.7 kb MEN-ε (*NEAT1_2*). Of note, the long MEN-β transcript forms an unusual triple helix structure at its 3′-end which has been shown to stabilize this long transcript. From the primary transcript, an additional short tRNA-like 59 nt ‘menRNA’ is generated through RNAse P and Z enzymatic cleavage. Panel (**B**): The *MALAT1* locus yields a 8.3 kb primary transcript also forms a stabilizing triple helix at the 3′-end, and through enzymatic cleavage another small tRNA-like 59 nt ‘mascRNA’. Using CRISPR-Cas9, we developed human monocyte-macrophage cell lines with menRNA or mascRNA sequence deletions as indicated. Panel (**C**): The editing procedures occur outside of the primary transcript sequences required for regular formation of the triple-helix structures at their 3′-ends which support stabilization of the respective lncRNAs. The inset shows the functional domains of MEN-β as determined through CRISPR-Cas9 deletion mapping by Yamazaki et al. [12] (by permission). This illustrates that the 57 nt deletion in the ΔmenRNA clone is located 21 nt 3′ of the A-rich motif and RNaseP cleavage site generating the MEN β-stabilizing triple helix. Panel (**D**): From direct sequencing data of the ΔmenRNA THP-1 cell clone it is obvious that menRNA cannot be generated in these cells since almost the entire menRNA coding sequence is deleted. Importantly, repeat sequencing confirmed deletion of this same sequence in all subsequent cell passages used for the experiments reported in this manuscript (>25 passages) (shown are the sequences of passages 10 and 25. The arrow indicates the human menRNA antisense sequence obtained with the reverse primer shown in panel (**A**). g-9 and g-10 indicate the single guide RNAs (sgRNAs) for CRISPR-Cas9 disruption of menRNA. Below, sequence data are shown for the DmasRNA clone. Panel (**E**): Structure prediction for possible residual transcripts from the CRISPR-Cas9-targeted ΔmenRNA and ΔmascRNA region shows that none of these exhibit any tRNA-like features. In vitro transcription of single-guide RNAs for CRISPR-Cas9 experiments (Supplementary Figure S3A) and PCR analysis of the deletion clones (Supplementary Figure S3B) are provided in the Supplement. Panel (**F**): Northern blot analysis showed that menRNA is present in wildtype control THP-1 cells and the ΔmascRNA clone, whereas in the ΔmenRNA clone no menRNA signal was detectable. Due to very low abundance of menRNA, Northern blot analysis was conducted with high total RNA loading (20 μg of total RNA per lane) and 7 days of exposure time. Since this experiment is performed at the lower level of detection for menRNA, absence of any menRNA signal in ΔmenRNA could not be considered proof of deletion per se. Sequencing does clearly confirm this assumption, however. We examined whether disruption of the menRNA sequence in the ΔmenRNA cells affects, by cytosolic-nuclear or other feedback mechanisms, the cellular expression level of the long precursors. Unlike monocytes/macrophages in *NEAT1^−/−^* [1] mice, CRISPR/Cas9-generated ΔmenRNA monocytes retained apparently normal MEN-ε/β and MEN-ε expression according to RT-PCR, as far as can be assessed by RT-PCR (mean ± SD of triplicate measurement). Primers in the 5′-region which amplify both MEN-ε and β (forward: 5′-GGGCCATCAGCTTTGAATAA-3′; reverse: 5′-CTTGAAGCAAGGTT-CCAAGC-3′), and other primers in the 3′-region which selectively amplify MEN-ε only (forward: 5′-GCTGAGAAGGAAGGTGCTTG-3′, reverse: 5′-CTGGCTAGTCCCAGT-TCAGC-3′) were taken from Sunwoo et al. [17]. Panel (**G**): The Northern blots show high abundance (as compared to menRNA) of mascRNA in wildtype control THP-1 cells, and absence of any mascRNA signal in the ΔmascRNA clone. ΔmascRNA monocytes preserved expression of the *MALAT1* precursor as assessed by RT-PCR. Under the experimental conditions employed there was no apparent change of *MALAT1* expression. Panel (**H**): Details of the adenoviral vectors employed for recombinant expression of menRNA. The U6 promoter enables efficient RNA polymerase III (RNAPIII)-dependent expression of short transcripts without poly-A tail. We previously characterized short hairpin RNA (shRNA) expression and RNA interference (RNAi) induction from this vector type [13,14]. The mascRNA and menRNA sequences to be cloned into this vector were annealed using four oligonucleotides for each sequence. These constructs were cloned into the pAd5-TetO7-U6 plasmid employing the BamHI and HindIII cloning sites. For detection of the recombinant expressed sequences the following radioactively labeled single-stranded oligonucleotides were used: (a) U6 antisense probe: 5′-gctaatcttctctgtatcgttccaattttagtatatgtgctgccg-3′; (b) hMALAT1 8368–8417 antisense probe (h-mascRNA probe): 5′-gcaaagacgccgcagggatttgaaccccgtcctggaaaccaggagtgcca-3′.

Here, we report biological functions of two tRNA-like transcripts from the *NEAT1-MALAT1* cluster (Figure 1) and describe their deep impact upon innate immunity and macrophage functions. While we previously investigated mice deficient in the entire *NEAT1* or *MALAT1* locus, we now aimed to selectively disrupt only the tRNA-like transcript ‘menRNA’ arising from *NEAT1* [7,8], or ‘mascRNA’ arising from *MALAT1.* No biological function independent of its precursor *NEAT1* has been assigned to menRNA so far, while a few studies have addressed mascRNA. After a report that mascRNA is involved in cardiovascular innate immunity [3], Sun et al. conducted an in-depth study demonstrating that mascRNA differentially regulates TLR-induced proinflammatory and antiviral responses [9]. Lu et al. showed that mascRNA promotes global protein translation, uncovering another role of mascRNA that is independent of *MALAT1* [10]. 

The two closely neighboring gene loci give rise to transcripts of vastly different size (*NEAT1*: 23 kb MEN-β, 3.7 kb MEN-ε, 59 nt ‘menRNA’; *MALAT1*: 8.3 kb primary, 58 nt ‘mascRNA’), and traditional knockout methods are unsuitable to selectively inactivate one of the small transcripts only. Through CRISPR-Cas9 editing [11], we therefore developed human monocyte-macrophage cell lines with short deletions in the respective tRNA-encoding sequences to disrupt normal menRNA or mascRNA formation, respectively. These editing procedures occur outside of the primary transcript sequences required for regular formation of the triple-helix structures at their 3′-ends which support stabilization of the respective lncRNAs (Figure 1A,B). Our study may be considered an extension of a previous pioneering study which identified, for the first time, functional domains within MEN-β through CRIPSR-Cas9 based deletion mapping [12]. While covering the entire length of MEN-β, that study has not reported on deletions downstream of the 3′-terminal A-rich motif essential for triple-helix formation and stabilization of MEN-β. Our ΔmenRNA clone disrupts the menRNA sequence 21 nt downstream of the A-rich motif, thus leaving triple helix formation and MEN-β intact (Figure 1C). Our CRISPR-Cas9-based editing of menRNA or mascRNA selectively prevented only the normal transcript folding and formation of mature menRNA or mascRNA, respectively. Unlike monocytes and macrophages in *NEAT1^−/−^* mice [1], CRISPR-Cas9-generated ΔmenRNA cells retained MEN-β and MEN-ε expression (Figure 1D). Similarly, ΔmascRNA monocytes preserved expression of the long *MALAT1* precursor while mascRNA, normally highly enriched in this cell type, became ablated (Figure 1E). 

Beyond prior work documenting immune function of the *NEAT1-MALAT1* cluster, the current study identifies menRNA as a novel element of innate immunity impacting upon cytokine regulation, immune cell-endothelium interactions, angiogenesis, and monocyte-macrophage differentiation and functions. For mascRNA, impact upon cell–cell interactions and angiogenesis are described. These small transcripts may be considered as prototypes of a new class of RNAs distinct from other small transcripts (miRNAs, siRNAs) by biosynthetic pathway and intracellular kinetics. From an evolutionary perspective, the *NEAT1-MALAT1* genomic region appears as archetype of a functionally highly integrated RNA processing system.

## 2. Materials and Methods

### 2.1. SHIP Population Study and Cohorts

For the human studies, approval was granted by the institutional ethics review board and the regulatory authorities. Investigation of the human tissues conformed to the principles in the Declaration of Helsinki. SHIP-TREND is a cross-sectional population-based study in Northeast Germany. From 2008 to 2012, 8826 randomly selected individuals aged 20 to 79 years were invited to participate in a comprehensive health examination (*Volzke H., Ittermann T., Schmidt C.O., Baumeister S.E., Schipf S., Alte D., Biffar R., John U., Hoffmann W. Prevalence trends in lifestyle-related risk factors. Dtsch. Arztebl. Int. 2015;112:185–192. doi: 10.3238/arztebl.2015.0185*). Written informed consent was obtained from all subjects and/or their legal guardian(s). The study was approved by the ethics committee of the University of Greifswald with ethics approval number BB 39/08 and complies with the Declaration of Helsinki. The study design has been published elsewhere (*Völzke H. Study of Health in Pomerania (SHIP) Bundesgesundheitsblatt-Gesundheitsforschung-Gesundheitsschutz. 2012;55:790–794. doi: 10.1007/s00103-012-1483-6*).

SHIP samples were genotyped using the Affymetrix Genome-Wide Human SNP Array 6.0. Genotyping of samples within SHIP-TREND was obtained in two batches using Illumina Infinium HumanOmni2.5 BeadChip and the Illumina Infinium Global Screening Array, respectively. Genotypes were determined using Birdseed 2 for SHIP and the GenomeStudio 2.0 Genotyping Module (GenCall algorithm) for SHIP-TREND. Standard genotype quality control was performed excluding arrays < 92% sample call rate for SHIP and <94% sample call rate for SHIP-TREND and duplicates (based on estimated IBD), mismatches between reported and genotyped sex, genetic PCA outliers, and arrays with extreme heterozygosity. Variants with call rate < 0.95 and a Hardy–Weinberg equilibrium *p*-value < 0.0001 were removed before imputation. Pre-phasing and Imputation of genotypes was performed with the Eagle and Minimac3 software to HRC reference v1.1 panel on Michigan Imputation Server v1.0.1 (https://imputationserver.sph.umich.edu/ (last accessed on 1 November 2022). Further details regarding the SHIP cohorts are provided in the Supplement.

### 2.2. CRISPR-Cas9 Experiments

THP-1 cells were grown in RPMI medium containing FCS (10% (*v*/*v*)) and penicillin/streptomycin (50 I.U./50 μg/mL). Parts of the menRNA and mascRNA regions were deleted from THP-1 cells using an adaptation of the CRISPR/Cas9 protocol described in Gundry et al. (2016). Protospacer sequences for each target gene were identified using the CRISPRscan scoring algorithm [http://www.crisprscan.org (last accessed on 1 November 2022) (Moreno-Mateos et al.)]. Extensive target searches employing http://www.crisprscan.org (last accessed on 1 November 2022), http://crispor.tefor.net/ (last accessed on 1 November 2022), and https://cctop.cos.uni-heidelberg.de (last accessed on 1 November 2022) identified no off-targets of potential relevance. DNA templates for single guide RNAs (sgRNAs) were generated by PCR (KAPA HiFi HotStart ReadyMix PCR Kit) using the pX458 plasmid containing the sgRNA scaffold sequence and using the following primers. 

PCR products were used to generate sgRNAs by in vitro transcription using HiScribe T7 High Yield RNA Synthesis Kit. 0.5 µg of purified sgRNA was incubated with Cas9 protein (1 μg; PNA Bio) for 15–20 min at room temperature. 2 × 10^5^ THP-1 cells were electroporated with the sgRNA/Cas9 complex using the Neon Transfection System at 1400 V, 20 ms, and one pulse. For the small RNA-deficient cell lines, two sgRNAs were selected at either end of the target sequence to delete the region in between. 

Single-cell clones were generated by single-cell plating of the parental cell line. Gene deletions in the single-cell clones were confirmed by sequencing and proved to be stable over >25 passages so far. 

### 2.3. Cell Culture Studies

#### 2.3.1. Human Monocyte Cultures

Human THP-1 cells were cultured in RPMI 1640 medium with 10% fetal bovine serum, 2 mM L-glutamine, 100 U/mL penicillin, and 100 μg/mL streptomycin. For gene expression analysis of wildtype (WT) and CRISPR-Cas9-targeted THP-1 cells under immune challenge, cells were stimulated with 100 ng/mL LPS, 10 ng/mL LPS, 1 ng/mL LPS, or 1 μg/mL Concanavalin A (Con A). After 24 h, RNA was isolated by TRIzol/Chloroform.

#### 2.3.2. THP-1 Monocyte Adhesion to Flow-Primed Human Aortic Endothelial Cells

For analysis of WT and CRISPR-Cas9-targeted THP-1 monocytes adhesion to flow-primed endothelial cells, primary human aortic endothelial cells (HAECs) (Source) were cultured in Endothelial Growth Medium-2 with 10% fetal bovine serum, 100 U/mL penicillin and 100 μg/mL streptomycin and seeded to confluence in µ-Slide y-shaped ibiTreat chambers. Endothelial cells were exposed to unidirectional flow (20 dyn/cm^2^) for 48 h using yellow/green perfusion sets prior to the experiment. THP-1 monocytes were labelled with 1,1′-dioctadecyl-3,3,3′,3′-tetramethylindocarbocyanine (DiI) for 15 min at 37 °C, respectively, and after washing three times, 1 × 10^6^ labelled cells were added to the flow reservoir for 30 min. After flow-termination, non-adherent cells were gently washed out with PBS, then cells were fixed with 4% paraformaldehyde, and the number of cells adhering to both the straight and branched channel regions was assessed by fluorescence phase-contrast microscope quantified using ImageJ Software (https://imagej.nih.gov/ij/ (accessed on 2 October 2022)).

#### 2.3.3. Tube Formation Angiogenesis Assay

(a) Conditioned media transfer experiment: Tube formation by HAECs treated with ΔmenRNA and ΔmascRNA monocyte-conditioned media was assayed on reduced growth factor basement membrane extract (BME) in 96-well tissue cultured-treated clear-bottom plates (15 × 10^3^ cells per well). Briefly, 60 µL of ice-cold BME was added per well and incubated at 37 °C and 5% CO_2_ for 30 min to allow gel formation. HAECs were plated on top of the gelled BME at a density of 1.5 × 10^4^ cells/well in 50 µL culture medium followed by transfer of 50 µL of monocyte-conditioned media of both ΔmenRNA and ΔmascRNA and incubated further for 24 h. After tube formation was observed, endothelial cells were stained by adding 50 µL of 6 µM Calcein AM solution per well for 15 min at 37 °C. Tubular network of the cells was captured using a fluorescent inverted microscope and quantified by ImageJ Software.

(b) Monocyte-HAEC co-cultures in matrigel: Endothelial tube formation in reduced growth factor matrigel was performed similarly as described above. Here, direct DiI-labeled co-cultured ΔmenRNA and ΔmascRNA monocytes at a cell density of 5 × 10^3^ per well together with HAECs (1.5 × 10^4^ cells/well) were added on the BME gel and cultured for 24 h at 37 °C and 5% CO_2_ to form tubular networks. 

### 2.4. Reactive Oxygen Species Assay

Intracellular ROS production in WT and CRISPR-Cas9-targeted THP-1 macrophages was determined as previously described using 2′,7′-dichlorodihydrofluorescein diacetate (H2DCFDA). Upon cleavage of the acetate groups by intracellular esterases, the cell-permeant H_2_DCFDA is retained within the cells and easily oxidized to the highly fluorescent 2′,7′-dichlorofluorescin (DCF) in response to ROS production/oxidative stress. Control and CRISPR/Cas9-targeted THP-1 cells were cultured in black 96-wells (overnight treatment with 0.1 µM PMA) before LPS addition for 24 h. After washing with HBSS, cells were incubated with 5 μM H2DCFDA (Molecular Probes) in HBSS for 1 h at 37 °C. Cells were washed again, and ROS production was induced by 400 μM H_2_O_2_. Fluorescence intensity was quantified every 10 min (excitation 485 nm, emission 535 nm) at 37 °C using a fluorescence plate reader (Tecan, https://lifesciences.tecan.com (accessed on 2 October 2022)).

### 2.5. Cytokine Measurements

Conditioned cell culture media of wildtype (WT) and CRISPR-Cas9-targeted THP-1 cells were tested for IFNγ, TNF, IL6, MCP1, IL10, and IL12p70 by use of Mouse Inflammation Cytometric Bead Assay (CBA) (BD Biosciences, Heidelberg, Germany) on a FACS CantoII flow cytometer (BD Biosciences) according to the manufacturer’s protocol. 

### 2.6. Cell Proliferation Studies

The proliferation assay of WT and CRISPR-Cas9-targeted THP-1 cells was conducted as follows: 5 × 10^4^ cells were seeded into clear 96-well plates, one plate for each time point. Proliferation was determined using WST1 reagent (Sigma-Aldrich, St. Louis, MO, USA) according to the manufacturer’s instructions. Absorbance at 450 nm was measured 2 h after addition of WST1 and incubation at 37 °C on a Tecan plate reader. Absorbance of WT cells was set to one at each time point.

### 2.7. Foam Cell Formation and oxLDL Uptake

To induce foam cell formation, we seeded 5 × 10^5^ THP-1 monocytes per well containing glass cover slips. Monocyte differentiation into macrophages was induced by adding 100 nM PMA to each well on days 0 and 1 and incubation for 48 h. Thereafter, the generated macrophages were washed with PBS, then incubated with 50 µg/mL human ox-LDL for 24 h in serum-free medium to induce foam cell formation. After ox-LDL incubation, Oil Red O staining (ORO) of the cells was performed as follows: wash with PBS, fix cells with 4% PFA/PBS for 15 min at RT, wash 3 times with PBS, rinse with 60% isopropanol for 15 s to facilitate the staining of neutral lipids, stain cells with filtered ORO working solution for 15 min at RT and in dark. ORO stock solution was prepared by dissolving 0.5 g ORO powder in 80 mL isopropanol (100%), mixed at 56 °C overnight, adjusted to 100 mL, mixed under gentle stirring, and filtered after pre-warming to 60 °C. Working solution was prepared by diluting ORO stock solution with ddH2O 3:2 = factor 1.5. For staining, cells were rinsed with water and then hematoxylin used as a counterstain (cell nuclei) for 10 s. Destaining by washing with 60% isopropanol for 15 s, then PBS 3 times. Glass cover slips were taken out and mounted on slides for polarization microscopy and microphotography. 

### 2.8. Monocyte-Macrophage Transition and Macrophage Polarization Experiments

Monocyte-M0 macrophage differentiation and subsequent M1/M2 macrophage polarization studies employing FACS and TaqMan were conducted as follows: First, M0 macrophages were generated by incubation of THP-1 monocyte clones for seven days, with PMA at a concentration of 100 ng/mL. Thereafter, the cells were further incubated for another seven days, either with IFN-γ at 20 ng/mL plus LPS at 100 ng/mL to induce M1 polarization or with IL-4 at 20 ng/mL plus IL-13 at 20 ng/mL to induce M2 polarization. The ‘M0’ TaqMan-based expression profiles and FACS data were obtained on day 7 of culture to characterize monocyte–M0 macrophage transition. The ‘M1’ and ‘M2’ expression profiles were obtained on day 14 of culture.

### 2.9. FACS Analyses of the Macrophage Clones

After macrophage polarization, cells were washed once with ice-cold PBS and scraped off using a mini scraper. Subsequently, macrophages were again washed with PBS + 5% FBS and incubated with 50 µL Fcγ-receptor block (BD Biosciences) in PBS for 10 min at RT to block unspecific binding. Cells were centrifuged at 300× *g* for 5 min at 4 °C. Then, cells pellets were resuspended with 50 µL of FACS Buffer (PBS + 0.5%FBS + 0.05% NaN_3_) and stained with APC mouse anti-human CD14 (BioLegend, San Diego, CA, USA), PE mouse anti-human CD11b (BD Biosciences), and Live/Dead Fixable Aqua Dead Cell Stain (Invitrogen, Waltham, MA, USA) for 30 min at 4 °C in the dark. Cells were resuspended in 450 µL of FACS Buffer and analyzed with Attune™ NxT Flow Cytometer (Thermo Fisher Scientific, Waltham, MA, USA) Statistical analysis was performed using GraphPad Prism 9 software. Experimental data were analyzed by using one-way analysis of variance (ANOVA) with Dunnett’s post hoc test for multiple comparisons. The distribution of variables was assessed by Kolmogorov–Smirnov tests of normality. 

### 2.10. Human Adenovirus and Coxsackievirus B3 Studies

THP-1 cells were cultured in *RPMI 1640* medium (ATCC modification + 10% fetal calf serum + 1% P/S) at 37 °C, 5% CO_2_. 5 × 10^5^ cells were seeded in 12-well culture plates and differentiation to macrophage-like cells was triggered by addition of 0.1 μM PMA o/n. Afterwards, cells were transduced with coxsackievirus B3 at MOI 30. Detection of CVB3 genome, replicative intermediates, and plaque-forming units (PFU) was conducted as described. In the adenovirus experiments, cells were instead transduced with a recombinant adenoviral virus expressing GFP (AdV5-GFP) or an “empty” adenovector expressing no transgene at MOI 25, 12 h post PMA addition. Virus structures were described previously.

### 2.11. RNA Sequencing and Data Analysis

For the transcriptome mapping of control vs. CRISPR-Cas9 generated ΔmenRNA or ΔmascRNA cells, four biological cell culture replicates were grown for each of the three clones. From each of these cultures, separate total RNA isolations were conducted by TRIzol/Chloroform method. Thereafter, the individual RNA preps were pooled for each of the clones, and the three resulting RNA pools (control, ΔmenRNA, ΔmascRNA) were subsequently used for RNA-seq analyses as follows. RNA integrity was visualized using Agilent Bioanalyzer 2100. For NGS-library preparation, we used Illumina TruSeq Stranded Total RNA Library Prep Human/Mouse/Rat (S45–S56) or NEBNext Ultr II Directional RNA Library Prep Kit Illumina (E7760S) in combination with the NEBNext rRNA Depletion Kit (Human/Mouse/Rat) (E6310L) (S145–S154). For all samples paired end (2 × 75 bp) sequencing was carried out on an Illumina NextSeq platform using NextSeq 500/550 High Output Kit v2.5 (150 cycles). The resulting reads were mapped to the human genome (GRCh37 release 87/hg19) using STAR v2.7.5b with standard options. We then ran the htseq-count module from software package HTSeq with the stranded = reverse option reflecting the used library kit. As we used rRNA-depleted samples, all with rRNA-annotation were excluded from further analyses. To detect differentially expressed genes, we used the R package DESeq v1.34.1. Next, we normalized the raw gene specific read counts as Transcripts per Million base pairs (TPM) in order to perform a gene set enrichment analysis (GSEA) using the R package ssGSEA2.0 by mapping them against a selection of gene set collections from Molecular Signature Database (MsigDB). We thus generated Enrichment Scores (ES) for each sample and various gene sets. ES reflects how strongly the majority of genes from an individual gene set are expressed per regarded sample. This ES was then normalized to account for variations in gene set size. To correct for multiple testing, the ssGSEA package uses the Benjamini–Hochberg method, which calculates false discovery rates (FDR). For any combination of gene set and sample with an FDR value ≥ 0.05, we set the NES to zero. The gene set collections we used were: C2.CP: Canonical pathways; KEGG selection as a subset of CP; C2.CGP: chemical and genetic perturbations; C7: immunologic signature gene sets. 

### 2.12. Quantitative RT-PCR

cDNA transcription and qPCR were conducted using standard methods. Reference gene was HPRT. Expression levels measured by qPCR were quantified as DDC_t_ values, determined by the C_t_ value of a candidate RNA minus the C_t_ of the reference gene. The TaqMan RT-PCR probes used are given in the Supplement.

### 2.13. Cloning and Recombinant Expression of Human menRNA and mascRNA

For oligonucleotide cloning of the human mascRNA sequence (with or w/o 3′ CCA terminus) into the BamHI and HindIII vector sites the following oligos were used: 

h-mascRNA 1: 5′-GATCCCCGATGCTGGTGGTTGGCACTCCTGGTTTCCA-3′; 

h-mascRNA 2: 5′-GGACGGGGTTCAAATCCCTGCGGCGTCTTTTTTA -3′; 

h-mascRNA 2+CCA: 5′-GGACGGGGTTCAAATCCCTGCGGCGTCTCCATTTTTA-3′; 

h-mascRNA 3: 5′-CGTCCTGGAAACCAGGAGTGCCAACCACCAGCATCGGG-3′;

h-mascRNA 4: 5′-AGCTTAAAAAAGACGCCGCAGGGATTTGAACCC-3′; 

h-mascRNA 4+CCA: 5′-AGCTTAAAAATGGAGACGCCGCAGGGATTTGAACCC-3′. 

Resulting h-mascRNA expression cassette w/o CCA terminus: 

5′-GATCCCCGATGCTGGTGGTTGGCACTCCTGGTTTCCAGGACGGGGTTCAA ATCCCTGCGGCGTCTTTTTTA-3′; 

3′-GGGCTACGACCACCAACCGTGAGGACCAAAGGTCCTGCCCCAAGTTTA-GGGACGCCGCAGAAAAAATTCGA-5′.

h-mascRNA expression cassette with CCA terminus: 

5′-GATCCCCGATGCTGGTGGTTGGCACTCCTGGTTTCCAGGACGGGGTTCA-AATCCCTGCGGCGTCTCCATTTTTA-3′; 

3′-GGGCTACGACCACCAACCGTGAGGACCAAAGGTCCTGCCCCAAGTTTA-GGGACGCCGCAGAGGTAAAAATTCGA-5′.

For the oligonucleotide cloning of the human menRNA sequence (ggcgctggtggtggcacgtc cagcacggct gggccggggt tcgagtcccc gcagtgttg) (with or w/o 3′ CCA tail) into the BamHI and HindIII vector sites the following oligos were used: 

h-menRNA 1: 5′-GATCCCCggcgctggtggtggcacgtccagcacggctg-3′;

h-menRNA 2: 5′-ggccggggttcgagtccccgcagtgttgTTTTTA-3′;

h-menRNA 2+CCA: 5′-ggccggggttcgagtccccgcagtgttgCCATTTTTA-3′;

h-menRNA 3: 5′-cggcccagccgtgctggacgtgccaccaccagcgccGGGGATC-3′;

h-menRNA 4: 5′-AGCTTAAAAAcaacactgcggggactcgaaccc-3′;

h-menRNA 4+CCA: 5′-AGCTTAAAAATGGcaacactgcggggactcgaaccc-3′.

These yielded the following h-menRNA expression cassette w/o CCA terminus: 

5′-GATCCCCggcgctggtggtggcacgtccagcacggctgggccggggttcgagtccccgcagtgttgTTT-TTA-3; 

3′-GGGccgcgaccaccaccgtgcaggtcgtgccgacccggccccaagctcaggggcgtcacaacAAAAATT-CGA-5. 

h-menRNA expression cassette with CCA terminus:

5′-GATCCCCggcgctggtggtggcacgtccagcacggctgggccggggttcgagtccccgcagtgttgCCA-TTTTTA-3′; 

3′-GGGccgcgaccaccaccgtgcaggtcgtgccgacccggccccaagctcaggggcgtcacaacGGTAAA-AATTCGA-5′

For target detection in the Northern blots, we used the following single-stranded oligonucleotides radioactively labeled by polynucleotide kinase:

U6 antisense probe:

5′-gctaatcttctctgtatcgttCCAattttagtatatgtgctgccg-3′

h-mascRNA antisense probe:

5′-gcaaagacgccgcagggatttgaaccccgtcctggaaaCCAggagtgCCA-3′

h-menRNA antisense probe:

5′-gactcgaaccccggcCCAgccgtgctggacgtg-3′

### 2.14. Statistical Analyses

Cell culture experiments: Statistical data analyses were done using IBM SPSS Statistics 24 or GraphPad software. Descriptive statistics include absolute and relative frequencies for categorial variables and mean and standard deviation, median, and range for quantitative measurements. For inter-group comparisons, Student’s *t*-test or the χ^2^ test was used for quantitative or categorical variables, respectively. *p*-values ≤ 0.05 are considered significant, and no Bonferroni adjustment has been performed. 

Human molecular genetics: Genome-wide association analyses of healthy samples versus samples with inflammatory, metabolic, and/or cardiovascular conditions were performed via logistic regression analysis implemented in snptest v2.5.2. For sensitivity analysis, samples were stratified by sex. Summary-level results were meta-analyzed with *METAL* (*Willer CJ, Li Y, Abecasis GR. METAL: fast and efficient meta- analysis of genomewide association scans. Bioinformatics. 2010;26:2190–1*) using the classical approach, which utilizes the effect size estimates and standard errors. Only variants with an imputation quality > 30% and Hardy–Weinberg equilibrium *p*-value > 0.0001 were included in the meta-analysis. Variants with *p*-value < 5 × 10^−8^ (the standard threshold) were considered to be genome-wide significant. For further details, please refer to the online Supplement.

## 3. Results

### 3.1. Targeted Deletion of tRNA-like Transcripts from the NEAT1-MALAT1 Cluster

While previously investigated mice were deficient in the entire *NEAT1* or *MALAT1* locus [1,2,4], here we aimed to selectively disrupt only the novel 59-nt tRNA-like transcript ‘menRNA’ with as yet unknown biological functions (Figure 1A). Through CRISPR-Cas9 editing (Table 1), we developed human THP-1 monocyte-macrophage cell lines with deletions downstream of the triple-helix ends of MEN-β (Figure 1A,C) or *MALAT1* (Figure 1B), respectively. All of these prevent normal transcript folding and formation of menRNA or mascRNA, respectively, as shown in detail in Figure 1E.

We examined whether the absence of menRNA or mascRNA in ΔmenRNA of ΔmascRNA monocytes affects, by some cytosolic-nuclear or other feedback mechanism, the cellular expression levels of the long precursors. Unlike monocytes/macrophages in *NEAT1^−/−^* [1] or *MALAT1^−/−^* [2] mice, the CRISPR/Cas9-generated ΔmenRNA monocytes/macrophages retained apparently normal MEN-β and MEN-ε expression, as assessed by RT-PCR (Figure 1D). Similarly, ΔmascRNA monocytes/macrophages preserved expression of the *MALAT1* precursor (Figure 1D), although mascRNA, normally highly enriched in this cell type, became ablated (Figure 1E). 

As a consequence of its rapid turnover, the cellular steady-state level of menRNA is very low. Low menRNA abundance in immune cells is in sharp contrast to the enrichment of mascRNA in monocytes first described by our group [3]. In that former study, we documented high efficacy of antisense oligonucleotides (ASOs) targeting the mascRNA sequence, regarding reduction of the mascRNA level in monocytes [3]. Similarly, in the present CRISPR-Cas9 study it was straightforward to confirm absence of mascRNA or mascRNA-homologous transcripts in ΔmascRNA cells by Northern blot analysis (Figure 1G). The high abundance of mascRNA in the human THP-1 monocytic cell line, as observed here, is consistent with our previous finding that mascRNA is highly enriched in human PBMCs and their subtypes [3].

Proof of efficacy and specificity of menRNA deletion was less straightforward due to its low abundance in organs and cell lines. Successful and precise disruption of the menRNA-forming sequence 3′ of the triple-helix was proven by regular sequencing of the target region (Figure 1D) in each of the ΔmenRNA clone passages used for the experiments in this project. Of note, this deletion remained stable over >30 passages, enabling conduction of a major series of experiments. On the other hand, proof of menRNA expression in wildtype THP-1 cells required Northern blot analysis with high RNA input loading and very long exposure times (Figure 1F).

Conventionally, rescue experiments employing vector-mediated overexpression of menRNA in ΔmenRNA cells would be considered suitable to further prove specificity of cellular effects of menRNA deletion. We conducted “rescue” experiments with recombinant menRNA overexpression, employing vectors as specified in Figure 1H. We previously used and characterized these vectors for short hairpin RNA (shRNA) expression and RNA interference (RNAi) induction [13,14]. The U6 promoter therein allows for efficient RNA polymerase III-dependent expression of short transcripts without poly-A tail, e.g., menRNA. The results of a short-term experiment with partial recovery of cytokine gene deregulations are reported below in the section Excessive inflammatory cytokine production. Of note, it was technically unfeasible to conduct long-term “rescue” in the more complex experiments addressing angiogenesis, cell–cell interactions, antiviral response, foam cell formation/oxLDL uptake, and M1/M2 polarization, because recombinant menRNA levels decayed rapidly and repeat vector transduction was unpracticable in these complex long-term assays. 

Beyond these technical issues, a principal problem of menRNA “rescue” arises from the fact that recombinant menRNA is generated by direct linear synthesis out of the vector upon its entry into the cytosol via endocytosis. In contrast, endogenous menRNA biosynthesis occurs through RNase P and Z cleavage out of the nuclear MEN-β transcript, normally followed by 3′-terminal CCA and CCACCA addition [8,15] with strong impact upon menRNA stability [15] and induction of the immune response [16]. As a side remark, it may be noted that the menRNA is stabilized when expressed from the constitutive RNA polymerase III promoter in the vector and that an additional CCA motif in the construct impairs its stability. In the particular case of menRNA, we therefore consider clearcut “rescue” precisely recreating the wildtype situation in ΔmenRNA cells technically unfeasible.

Due to their known post-transcriptional mechanisms of action, neither ASOs nor siRNAs selectively interact only with the short menRNA or mascRNA sequences. Instead, they will also interact with these sequences when still embedded in the long precursors’ transcripts from *NEAT1* and *MALAT1* and may mediate their premature decay, making unequivocal distinction between consequences of menRNA/mascRNA ablation from those of *NEAT1*/*MALAT1* reduction [1,2,4] impossible. In the peculiar case of the high-turnover menRNA, the CRISPR-Cas9 deletion clone with its deletion at the DNA level is obviously a particularly useful highly specific and stable system for selective menRNA ablation and functional assignment of cellular functions to menRNA. 

Regarding in vivo studies, any attempt to generate germline menRNA- or mascRNA-deficient animal models will be technically demanding and likely also display developmental/embryonic anomalies and disturbance of diverse somatic cells other than immune cells/monocytes. One would instead need mice with a monocyte-specific, adult-age inducible selective knockout of the menRNA or mascRNA sequence only to resolve these issues. Beyond these technical challenges, there exists no murine homologue to human IL-8, which was, however, the most strongly deregulated cytokine in circulating immune cells of post-MI patients [1]. As a first step, these ambiguities are avoided by CRISPR-mediated deletion in one specific cell type/line of human origin. We examined whether absence of menRNA in ΔmenRNA monocytes affects, by some cytosolic-nuclear feedback mechanism, the cellular expression level of *NEAT1*. Under the experimental conditions employed here, however, there was no change detectable by RNA-sequencing (RNA-seq) or qRT-PCR.

### 3.2. Defective Innate Immune Sensing by ΔmenRNA and ΔmascRNA Cells

**Figure 2:**
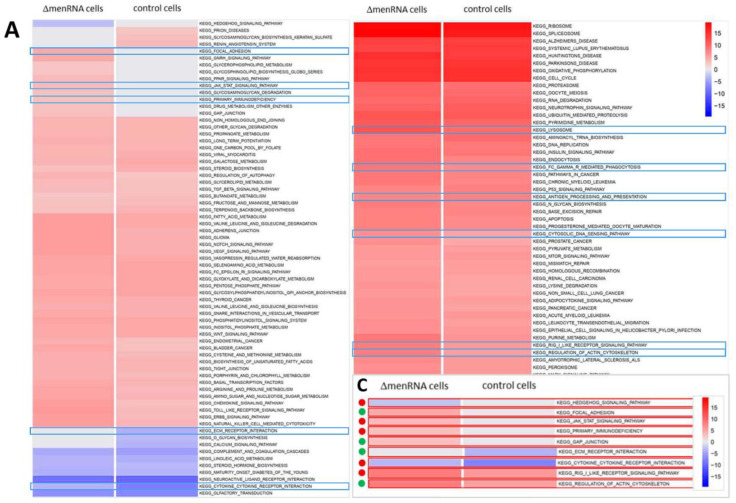

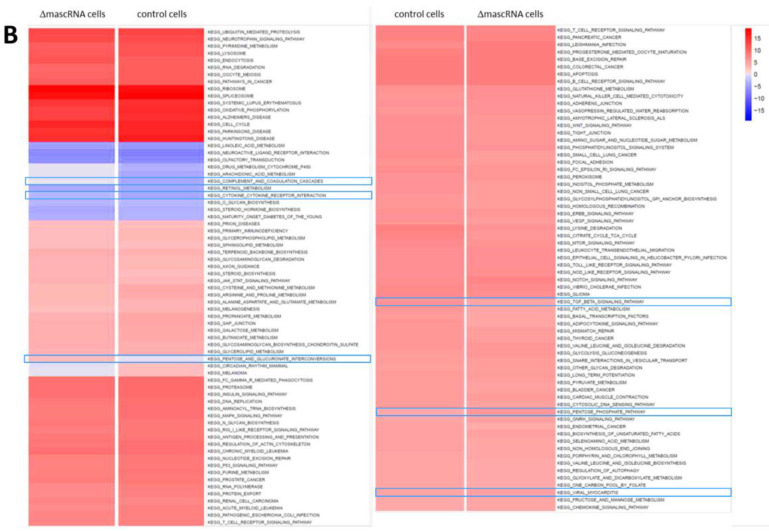
Gene set enrichment analysis of ΔmenRNA and ΔmascRNA monocytes. RNA-seq was employed as an initial genome-wide screening tool to detect any alterations in the protein-coding or noncoding transcriptome of wildtype vs. ΔmenRNA or ΔmascRNA cells. This RNA-seq screening identified profound alterations in the baseline transcriptomes of ΔmenRNA (panel (**A**)) and ΔmascRNA (panel (**B**)) monocytes. Gene set enrichment analysis (GSEA) of RNA-seq datasets mapped against gene sets from Molecular Signature Database’s (MSigDB) collection derived from Kyoto Encyclopedia of Genes and Genomes (KEGG) pathway database. The shown color code bar represents the normalized enrichment score. Panel C highlights immune response and cell–cell interaction-associated biological processes enriched by genes deregulated in ΔmenRNA cells: primary immunodeficiency, cytokine-cytokine receptor interaction, JAK-STAT-signaling, focal adhesion, GAP junction, extracellular matrix receptor interaction, regulation of actin cytoskeleton, and hedgehog signaling [19,20]. The red dots indicate related immune response, and the green dots indicate cell–cell interaction-associated biological processes. Taking these initial RNA-seq screening data as a starting point, we subsequently validated a large number of deregulated genes by qRT-PCR and extensively investigated them in monocyte (Figure 3, Figure 4, Figure 5 and Figure 6) and differentiated macrophage (Figure 7, Figure 8 and Figure 9) clones. Further details regarding the RNA-seq analyses are provided in Supplementary Table S1/Table S2. Supplementary Table S3 displays all genes encompassed within the KEGG pathways highlighted in Panel C with their respect. For high-resolution maps of the GSEA, please refer to the figure’s source file.

**Figure 3:**
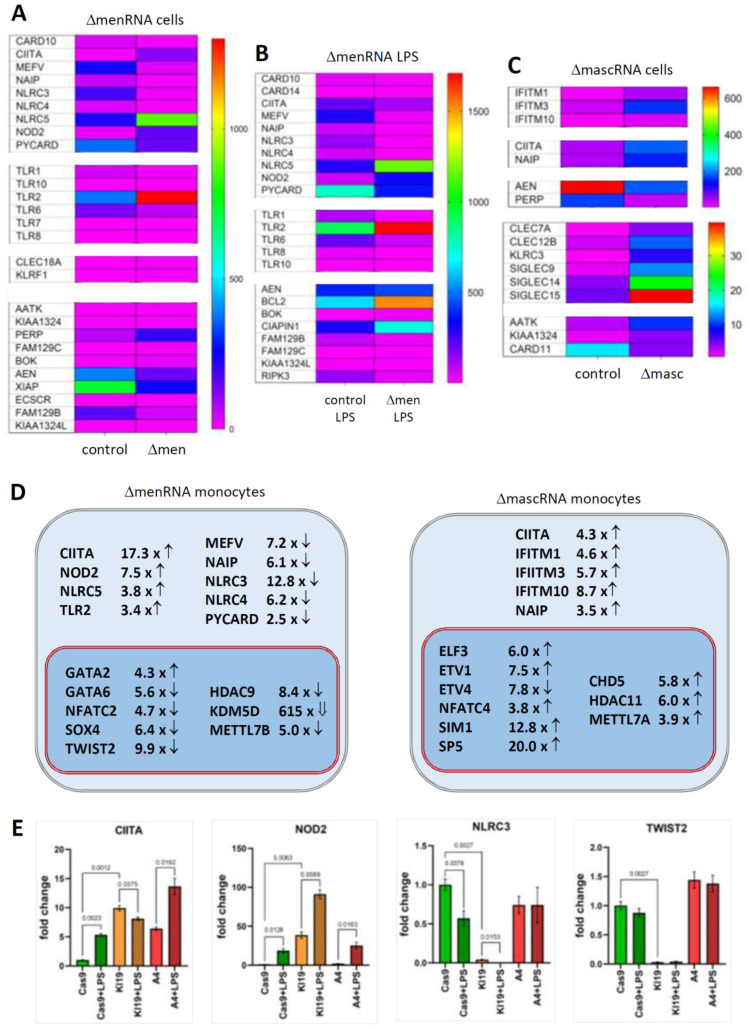

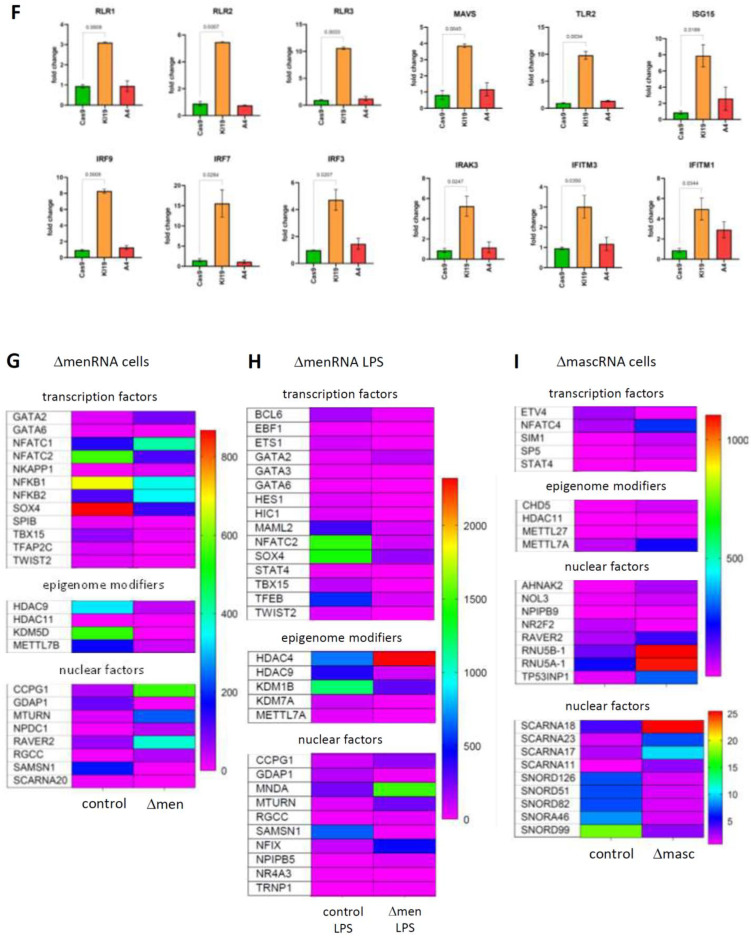

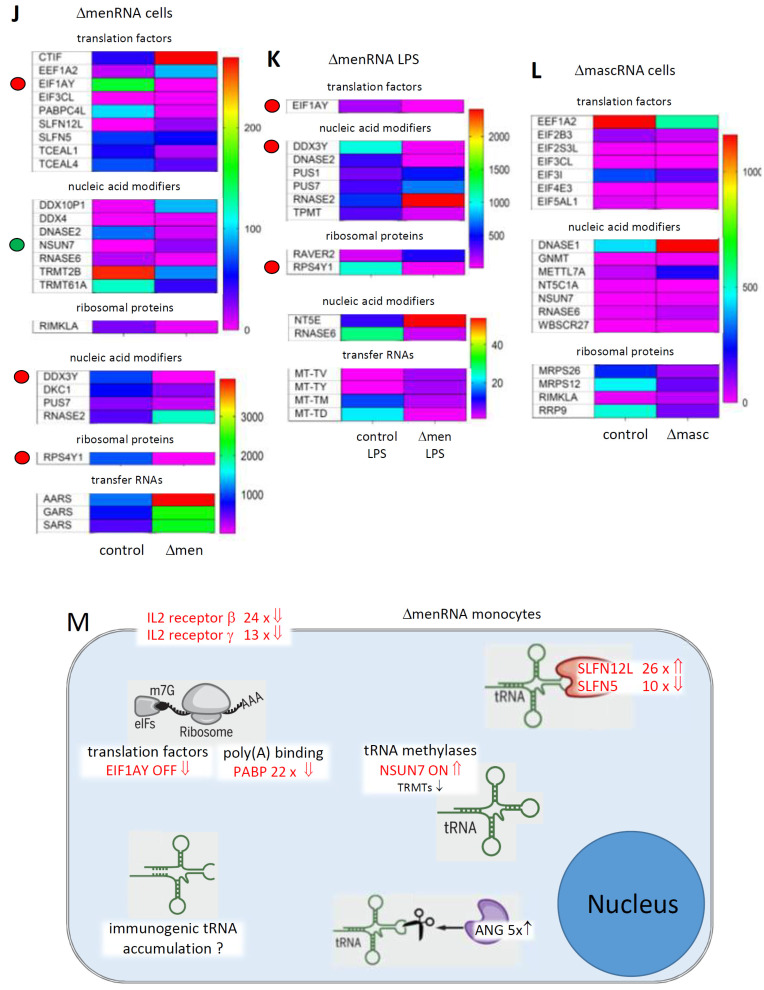
Disturbances of innate immune sensing, transcription, and translation in defective monocytes. Panels (**A**–**E**) (Innate immune sensing): A key finding of the RNA-seq was imbalance between multiple innate immune sensors in ΔmenRNA cells under baseline (panel (**A**)) and LPS-stimulated (panel (**B**)) conditions (Table S1A). The color bars associated with the rainbow plots in panels (**A**–**C**,**G**–**I**,**J**–**L**) represent FPKM values. Amongst cytosolic receptors, NOD2 [21] was ~8-fold- and CIITA [35] ~17-fold-induced, whereas NLR-class receptors NLRC3, NLRC4, NAIP, and MEFV were suppressed in ΔmenRNA monocytes. Amongst membrane-bound Toll-like receptors, TLR 2 showed ~10-fold elevated baseline expression in ΔmenRNA cells (compare Figure 5A). In contrast, defective induction was seen for TLR6, TLR1, TLR8, TLR10, and TLR7 in ΔmenRNA upon LPS challenge (panel (**B**)). Twist family bHLH transcription factor 2 (TWIST2) [26] was ~10-fold down in these ΔmenRNA cells (panels (**G**,**H**)), and loss of NLRC3 and TWIST2 expression in ΔmenRNA monocytes could not be rescued by LPS (panel (**E**), compare Figure 6A for related findings in macrophages). mascRNA cells likewise displayed anomalies in immune sensor expression (panel (**C**)) (Table S2A), however different from those observed in ΔmenRNA cells. Prominent in ΔmascRNA monocytes was the induction of interferon (IFN)-induced transmembrane (IFITM) proteins which represent primary cellular defenses against multiple viruses [27]. Upon PMA-induced differentiation of ΔmenRNA and ΔmascRNA monocytes into macrophages, multiple further baseline anomalies of antiviral defense genes became apparent (Supplementary Figure S9). In both defective clones, there was imbalance of apoptosis-related genes. In LPS-stimulated ΔmenRNA cells (panel (**B**)) apoptosis regulator BOK and Niban apoptosis regulator FAM129C were upregulated, whereas serine/threonine kinase RIPK3 and apoptosis inhibitors NAIP and CARD14 were suppressed here. In ΔmascRNA cells (panel (**C**)), NAIP was induced while apoptosis-enhancing nuclease AEN [36] was ~3.-fold- and p53 apoptosis effector PERP [37] ~5.1-fold-suppressed (Table S2A). In addition to alterations of protein-coding genes, RNA-seq identified deregulation of long noncoding RNAs (lncRNAs) and antisense (AS) RNAs in ΔmenRNA and ΔmascRNA monocytes (Table S1D/Table S2D). Thus, ΔmenRNA cells showed ~7-fold downregulation of a recently discovered novel transcript designated ’MARCKS cis-regulating lncRNA promoter of cytokines and inflammation’ or ‘regulator of cytokines and inflammation’ (ROCKI) (see Figure 4D,E) (Table S1D/Table S2D). Panels (**F**–**I**) (Transcriptional level): Several transcription factors (TF), nuclear factors and epigenome modifiers were deregulated in defective cells. At the transcriptional level, in addition to TWIST2, TF GATA2 was ~4-fold-induced and GATA6 ~20-fold-suppressed, and NFATC2 switched off in ΔmenRNA cells (panels (**G**,**H**)) at baseline already (compare Figure 5A,D for related findings in macrophages). Further TFs (SOX4, ETS1, STAST4) and epigenome modifiers turned into disequilibrium upon LPS challenge, among them histone deacetylases (HDAC4, HDAC9, HDAC11), lysine demethylases (KDM1B, KDM7A), and METTL methyltransferase family members [28] known or predicted to methylate DNA, RNA, or proteins (METTL7A, METTL7B). Panel (**D**) summarizes the most prominent changes of cytosolic and membrane-bound innate immune sensors and of transcription factors and epigenome modifiers in ΔmenRNA and ΔmascRNA monocytes. Panel (**E**) exemplifies how LPS-stimulation exacerbates several of these deregulations (e.g., CIITA and NOD2), while this stimulation it is incapable of overcoming certain blocks (e.g., of NLRC3 or TWIST2) (see Figure 6A for related findings in macrophages). Bar graphs show means ± SE from three biological replicates. Panels (**J**–**M**) (Translational level): Particular anomalies were identified regarding translation factors, ribosomal proteins and nucleic acid modifiers in ΔmenRNA (panels (**J**,**K**)) and ΔmascRNA monocytes (panel (**L**)). In ΔmenRNA cells translation initiation and elongation factors EIF3CL, EEF1A2, and CTIF were induced, whereas initiation factor EIF1AY and ribosomal protein RPS4Y1 were respectively 33-fold and 45-fold down in ΔmenRNA cells (**J**,**K**). Nucleic acid-modifying DEAD-box helicase DDX3Y was 333-fold down, whereas methyltransferase NSUN7 [30,31,32], undetectable in controls, was robustly expressed in ΔmenRNA cells (**J**), (Table S1C). Certain tRNAs and tRNA methyltransferases (TRMT61A, TRMT2B) displayed major deregulation in ΔmenRNA cells (panel (**J**,**K**)). ΔmascRNA cells (panel (**L**)) showed none of these, but cytosolic nucleotidase NT5C1A is ~10-fold suppressed here. Panel (**M**) summarizes changes regarding the translational machinery in menRNA-deficient monocytes, including translation factors, a poly(**A**) binding protein, tRNA methylases, two SLFN family members, angiogenin (ANG), and IL2 receptor subunits β and γ.

**Figure 4:**
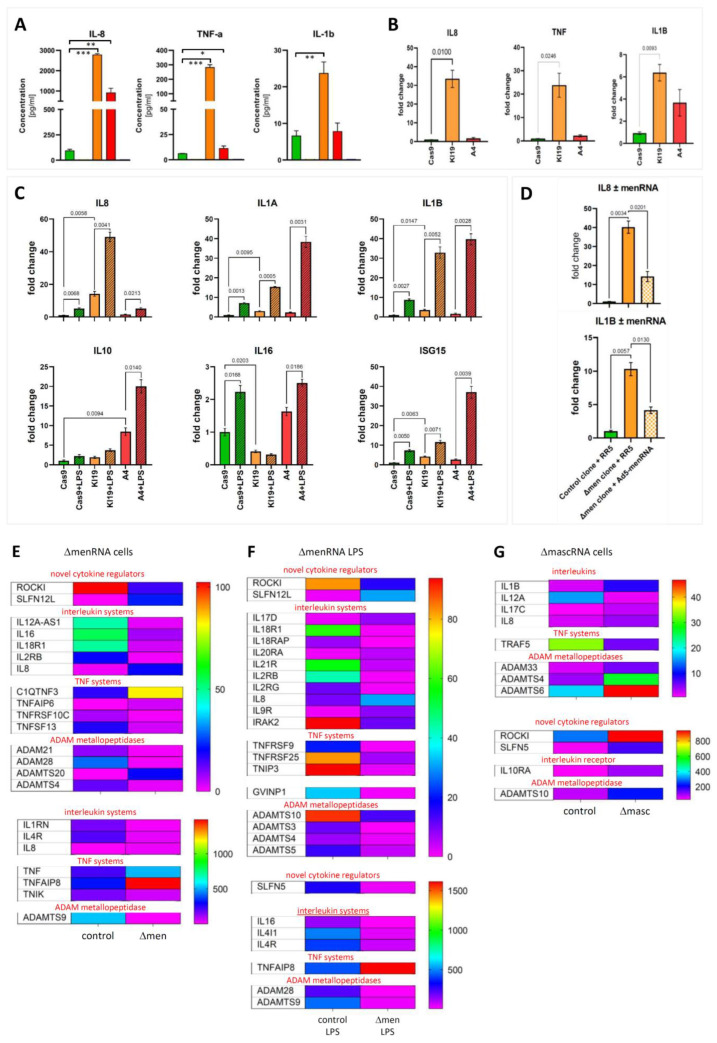
menRNA and mascRNA deletion results in loss of cytokine control and imbalance of interleukin systems. Panel (**A**): A prominent finding was the massively elevated expression and secretion of IL-8, TNF, and IL1B in unstimulated ΔmenRNA cells. Cytometric bead assay protein data on media conditioned by control monocytes (green), ΔmenRNA (orange), and ΔmascRNA cells (red). Panel (**B**): qRT-PCR quantification of gene expression levels. For all three proinflammatory cytokines, ΔmenRNA displayed far more pronounced induction than ΔmascRNA cells. Panel (**C**): ΔmascRNA monocytes displayed exaggerated induction of IL1A, IL1B, IL10, and ISG15 expression upon LPS-stimulation as compared to controls. Only stimulated ΔmenRNA cells showed a further increase of IL8 expression, whereas IL16 [38] was suppressed in this clone at baseline and failed to become induced upon LPS challenge. Panel (**D**): Partial “rescue” of cytokine control in ΔmenRNA cells by recombinant human menRNA expressed from the adenovector Ad5-menRNA (without CCA terminus) described in Figure 1H. RR5 is an “empty” control vector without an expression cassette (means ± SD of triplicate measurements) (* denotes *p* < 0.05, ** *p* < 0.01, *** *p* < 0.001). Panels (**E**,**F**): RNA-seq identified further anomalies of IL and TNF systems. The color bars associated with the rainbow plots in panels (**E**–**G**) represent FPKM values. Among receptors, IL4R was ~7-fold- and IL4-induced gene (IL4I1) ~11-fold-suppressed in stimulated ΔmenRNA cells (panels (**E**,**F**)). Even more pronounced was disequilibrium in the IL18 system (IL18R1 > 70-fold inhibited, IL18 receptor accessory protein IL18RAP switched off). In the IL2 system, receptor subunit IL2R-β was ~52-fold- and IL2R-γ ~13-fold-suppressed in LPS-stimulated ΔmenRNA monocytes (panel (**F**)). Regarding the IL1 system, IL1 receptor antagonist IL1RN was ~12-fold and IL1 receptor-associated kinase IRAK2~8-fold down in ΔmenRNA cells. Among the ligands, IL16 expression remained ~18-fold-blunted even upon LPS-challenge (compare panel (**C**)).

**Figure 5:**
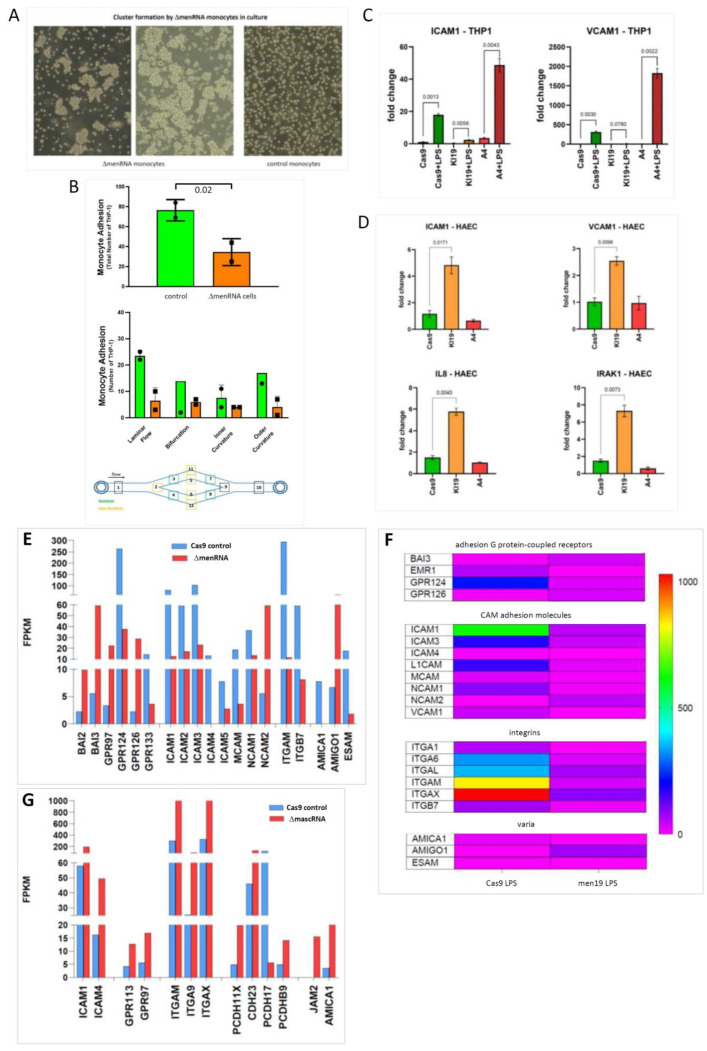

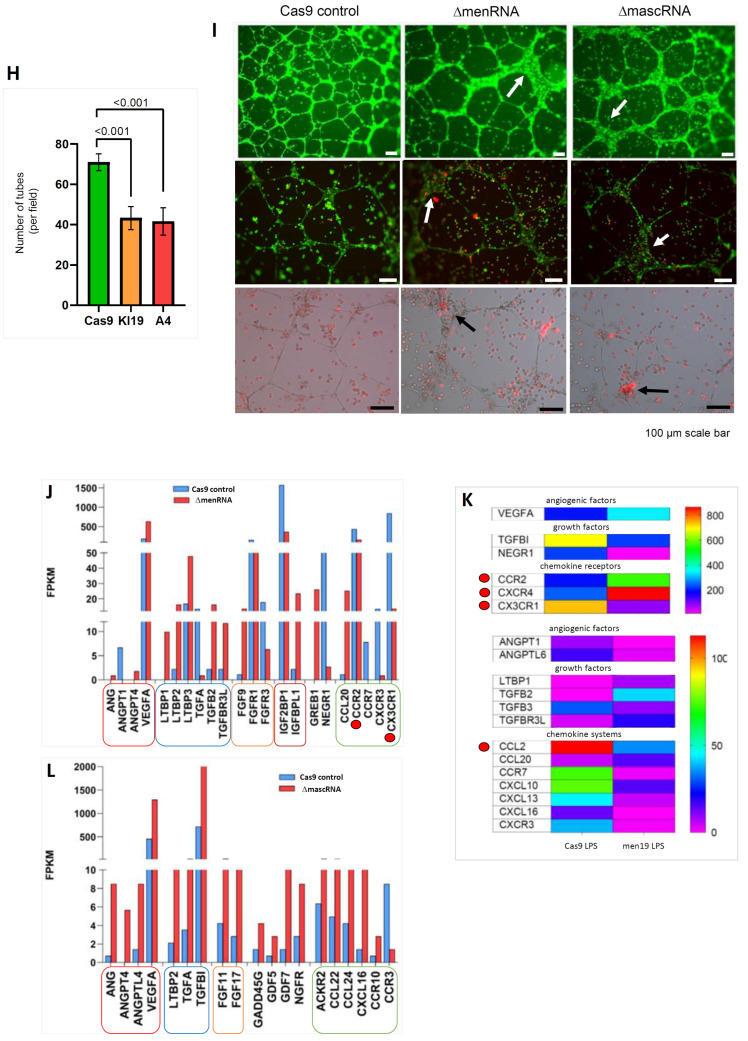
Impact of menRNA and mascRNA deletions upon monocyte-endothelium interactions and angiogenesis. Panels (**A**–**D**) (Endothelial cell interactions): At the cellular level, ΔmenRNA monocytes displayed an anomalous growth pattern with spontaneous cell cluster formation in liquid culture (panel (**A**)). Despite this morphological anomaly, there was no significant difference in their cell proliferation kinetics compared to ΔmascRNA and control monocytes (data not shown). ΔmenRNA cells showed defective endothelial adhesion under multiple flow conditions (panel (**B**)). While expression of vascular cell adhesion molecule 1 (VCAM-1) was ~200-fold-induced by LPS, starting from a very low baseline level in control cells, this induction was almost entirely blunted in ΔmenRNA cells. Similarly, the LPS-dependent induction of ICAM1 was strongly reduced in these defective cells (panel (**C**)). Transfer of ΔmenRNA monocyte-conditioned medium to HAEC endothelial monolayers triggered changes in the expression of multiple genes including ~10-fold-increased ISG15, ~8-fold-increased IL8, ~7-fold-increased IRAK1, and ~7-fold-increased ICAM1, whereas ΔmascRNA-conditioned medium exerted no such influence. Bar graphs show means ± SE from two biological replicates (panel (**D**)). Panels (**E**–**G**) (Cell adhesion molecule profiles): RNA-seq identified profound deregulation of multiple cell adhesion molecules in ΔmenRNA cells (panels (**E**,**F**)), including adhesion G protein-coupled receptors (GPRs, BAIs), intercellular adhesion molecules (ICAMs), neural cell adhesion molecules (NCAMs), integrins (α_M_, α_L_, α_X_, α_1_, α_4_, α_6_, β_7_), vascular cell adhesion molecule (VCAM1), and ESAM (endothelial cell-specific adhesion molecule). (α_M_, α_L_, α_X_, α_1_, α_4_, α_6_, β_7_), mascRNA cells (panel (**G**)) displayed deregulation of several cell adhesion molecules as well, however distinct from those observed in menRNA cells. Thus, neuronal growth regulator NEGR1, involved in neuronal growth and connectivity and cell–cell interactions in general, is ~28-fold down in Δmen19 but unaltered in Δmasc19 cells. Cytoskeleton-regulating NCKAP1 was ~14-fold-increased in ΔmascRNA but unaltered in ΔmenRNA cells. Panels (**H**–**L**) (Angiogenesis, growth factors and chemokine systems): Quantitative effects of both ΔmenRNA (orange) and ΔmascRNA (red) monocyte-conditioned media upon HAEC-based tube formation in an angiogenesis assay, as compared to Cas9 controls (green). Supernatant from each of the defective cell clones highly significantly reduced the number of tubes formed. Bar graphs show means ± SE from three biological replicates (panel (**H**)). Beyond a quantitative effect of secreted factors from the cells, direct co-culture of ΔmenRNA, as well as of ΔmascRNA monocytes with the HAECs, lead to profound alterations of HAEC cell morphology. In co-cultures with any of the defective monocytic clones, there was a massive increase in the size of HAECs at many branch side nodes where ≥3 endothelial cells came into contact (white arrows in the upper and middle rows). When the defective monocytes clones were red-stained (with DiI) before addition to the HAEC-matrigel mixture, there was significant accumulation of red cells (black arrows in the lower row) at branch side nodes where HAEC hypertrophy occurred (panel (**I**)). For a high-resolution version of this figure, please refer to the source file. There was grave imbalance of angiogenesis-associated factors (ANG, VEGF), chemokine receptors (CCR2, CX3CR1), and TGF system components in ΔmenRNA (panels (**J**,**K**), Table S1G) and ΔmascRNA monocytes (panel (**L**), Table S2G). The color bars associated with the rainbow plots in panels (**F**,**K**) represent FPKM values.

**Figure 6:**
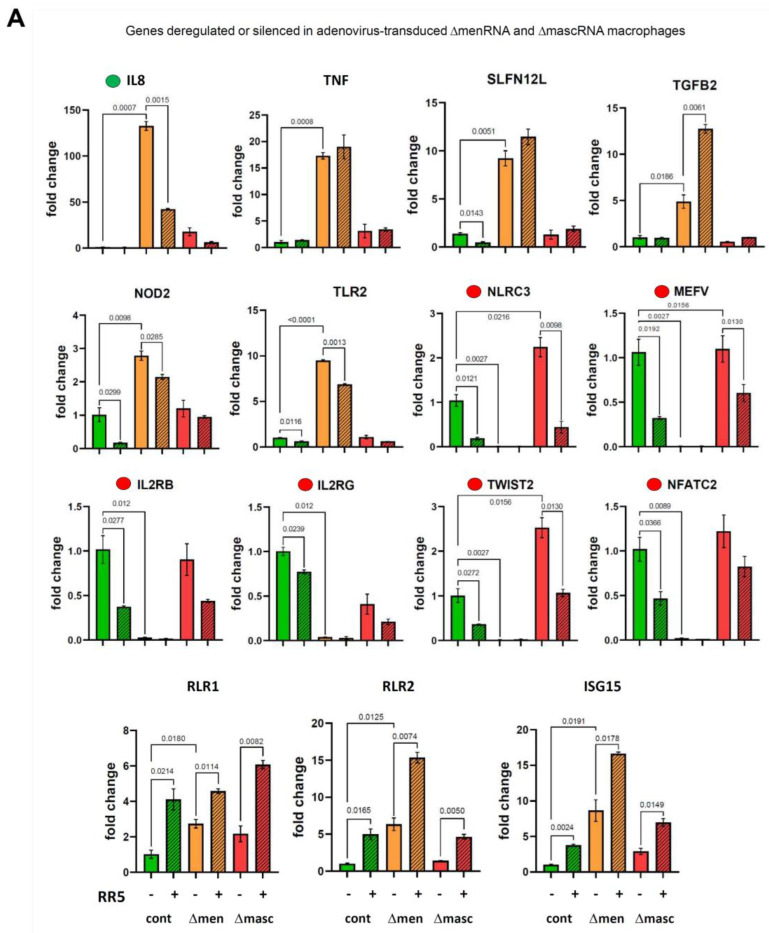

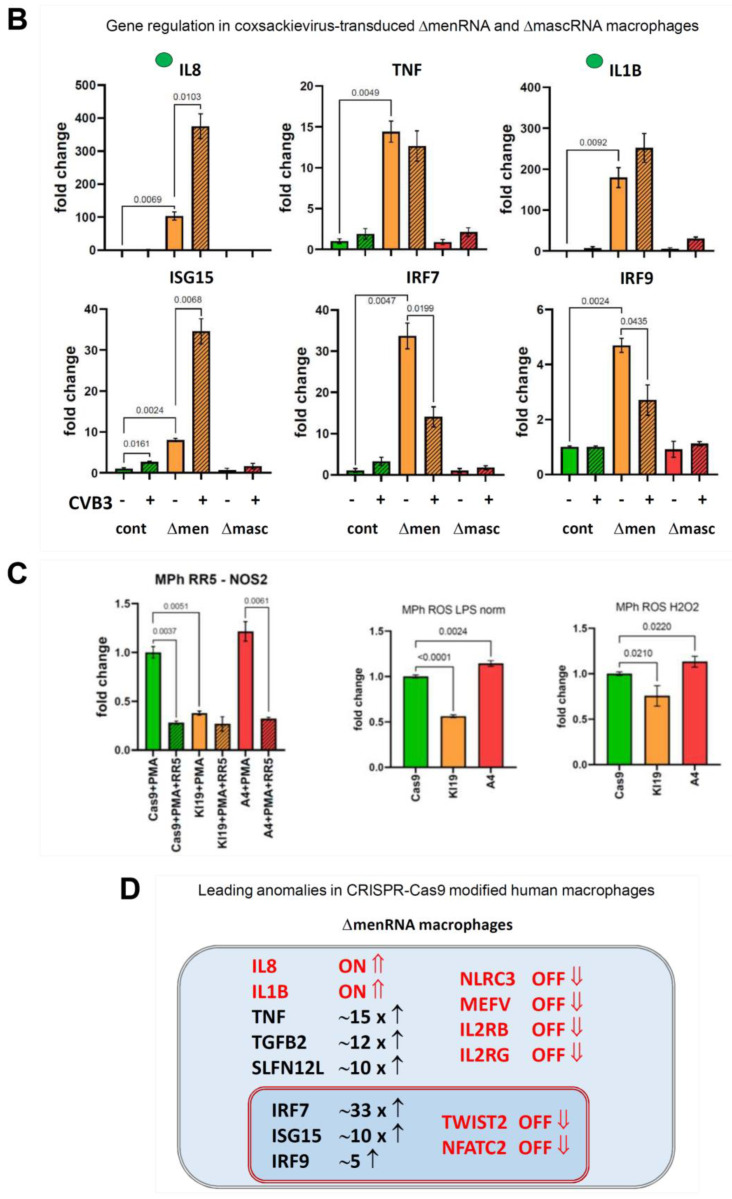
Antiviral response of ΔmenRNA and ΔmascRNA macrophages. PMA-induced differentiation of ΔmenRNA cells to adherent macrophages rendered the cells susceptible to efficient transduction by recombinant viruses derived from the human-pathogenic double-stranded DNA (dsDNA) adenovirus type 5. At a multiplicity of infection (MOI) of 25, the GFP-expressing virus AdV-CMV-GFP efficiently transduced these macrophages, resulting in strong GFP expression in >50% of cells (Supplementary Figure S8A). In the generated macrophages, IL8 baseline expression without LPS stimulation or virus exposure was massively ~130-fold-elevated compared to controls (panel (**A**)). Transduction with a recombinant adenovirus without the GFP expression cassette (AdV-RR5) resulted in a decrease of IL8 expression to ~40-fold of the level in controls. In contrast, control cells displayed no change of their baseline IL8 expression in response to virus. ΔmenRNA macrophages displayed ~17-fold-elevated TNF and ~5-fold-elevated TLR2 baseline expression compared to controls, without response to virus. Several other genes were induced in response to transduction (RIG-like receptors RLR1 and RLR2, ISG 15), but without significant differences between defective and control cells. Most striking was the complete shutdown of several genes already suppressed in ΔmenRNA monocytes (Figure 3A–E, Figure 4E,F) before their transformation into macrophages: NOD-like innate immune genes NLRC3 and MEFV, IL2 receptor subunits β and γ, transcription factor TWIST2, and NFATC2. None of these genes fully silenced in macrophages could be ’rescued’ by adenovirus exposure, similar to findings in LPS-stimulated monocytes (Figure 3A–E, Figure 4E,F). Bar graphs show means ± SE from three biological replicates. Panel (**B**) shows the response of ΔmenRNA macrophages to Coxsackievirus B3, an important human-pathogenic single-stranded RNA (ssRNA) virus. Their IL8 response to CVB3 (~5-fold induction upon virus exposure) was opposite to that to AdV-RR5 (reduction of IL8 to ~2/3 of the high baseline level). Efficient transduction by CVB3 was detected by qRT-PCR, while CVB3 (−) minus strand-specific RT-PCR indicative of active replication was negative for all clones (Supplementary Figure S8B). Bar graphs show means ± SE from two biological replicates. Panel (**C**) displays blunted NOS2 expression (qRT-PCR) by ΔmenRNA but not ΔmascRNA macrophages at baseline and upon adenovirus exposure, as well as impaired ROS production after LPS or H_2_O_2_ challenge. Bars graphs show means ± SE from three biological replicates. Panel (**D**) summarizes the leading anomalies in CRISPR-Cas9-modified human ΔmenRNA macrophages.

**Figure 7:**
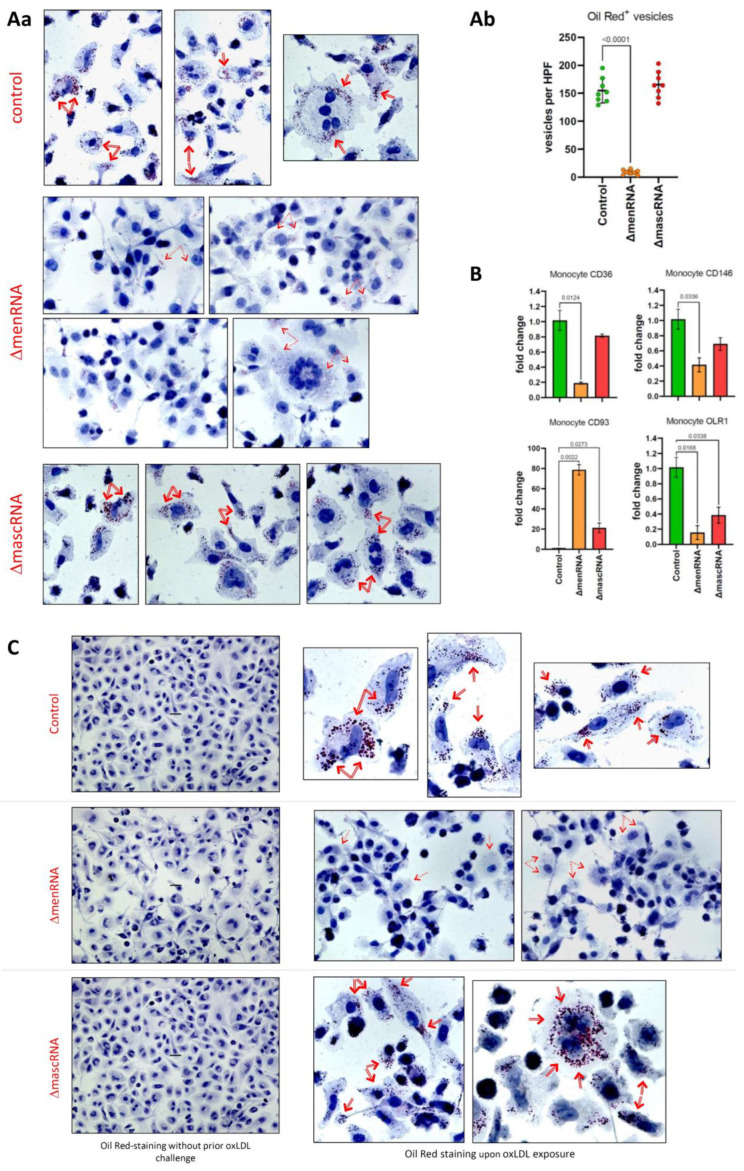
Defective foam cell formation and oxLDL uptake by ΔmenRNA and ΔmascRNA macrophages. Panel (**Aa**): ΔmascRNA macrophages displayed an oxLDL uptake pattern indistinguishable from that of control cells (intracellular Oil Red-positive OR^+^ vesicles as indicated by red arrows). In contrast, essentially no normal-sized intracellular OR^+^ vesicles were observed in ΔmenRNA macrophages (*p* < 0.0001) 24 hrs after oxLDL exposure. A minor number of very small OR^+^ particles is visible in ΔmenRNA cells. It appears these minute particles are not artifacts, and do not represent spontaneous intracellular accumulation of any OR^+^ material in the ΔmenRNA cells, but residues of defective oxLDL endocytosis since they do not show up without prior oxLDL challenge (panel (**C**)). Panel (**Ab**): Quantification and statistics of OR^+^ vesicles in the different cell clones. Panel (**B**): The striking defect of oxLDL uptake by ΔmenRNA macrophages is paralleled by loss of scavenger receptors CD36, MCAM (CD146) [67], MSR1, and OLR1 (LOX1) [68]. In contrast, the immunoregu-latory lectin receptor CD93 [52] is ~80-fold upregulated in PMA-generated ΔmenRNA macrophages compared to controls, an effect similarly observed (~35-fold induction) in unstimulated ΔmenRNA monocytes (Figure 8). Bar graphs show means ± SE from four biological replicates. Please note the graphs in panel B display TaqMan data (normalized to control) from PMA-differentiated monocytes (macrophage clones), whereas Figure 8 displays RNA-seq data (FPKM values) from the respective undifferentiated monocyte clones.

**Figure 8:**
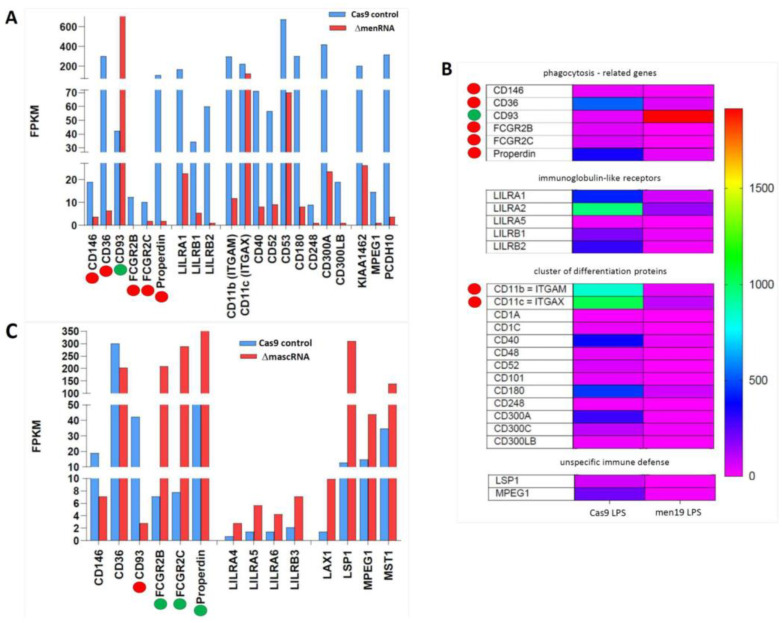
Disturbance of scavenger receptor expression in ΔmenRNA and ΔmascRNA macrophages. Panels (**A**–**C**): These changes occur in the context of further anomalies, identified by RNA-seq of monocytes, affecting phagocytosis as an evolutionarily conserved general defense mechanism involving, beyond scavenger receptors, Fcγ receptors (FcγRs) [59,60] and complement receptors (CRs). FCGR2 genes encoding Fc fragments of inhibitory FcγRIIB are suppressed in ΔmenRNA cells and not rescuable by LPS (panel (**A**,**B**)). Complement component properdin (CFP) is likewise down. Of note, ΔmascRNA cells (panel (**C**)) display opposed deregulation of the same FCGR2 genes, and induction of properdin and other complement components. Also Remarkable is the strong opposed deregulation of CD93 in ΔmenRNA compared to ΔmascRNA monocytes. While massively induced in ΔmenRNA cells, CD93 was shut down in ΔmascRNA monocytes, contrasting with robust expression in controls. CD93 is a lectin receptor involved in control of the immune response [52]. Another distinction between the defective clones regards MPEG-1 (perforin-2), an evolutionary ancient protein involved in the unspecific immune defense [53], which was ~16-fold down in ΔmenRNA but upregulated in ΔmascRNA cells. Scavenger receptor CD36 [45,47] was ~48-fold down in ΔmenRNA monocytes. They also show suppression of Cluster of Differentiation (CD) marker CD40, a member of the TNF receptor family, and of CD52, a glycoprotein-modulating T-cell activation. CD300A and CD300C, involved in viral immune evasion, were ~26-fold- and ~5-fold-suppressed, respectively. None of the group 1 CD1 molecules, normally expressed on cells specialized in antigen presentation, could be induced by LPS-treatment of ΔmenRNA cells. Finally, leukocyte immunoglobulin-like receptors (LILRs) were broadly deregulated. ΔmascRNA cells (panel **C**) displayed none of these ΔmenRNA-associated CD molecule or LILR deregulations. The color bar asociated with the rainbow plot in panel (**B**) represents FPKM values.

**Figure 9:**
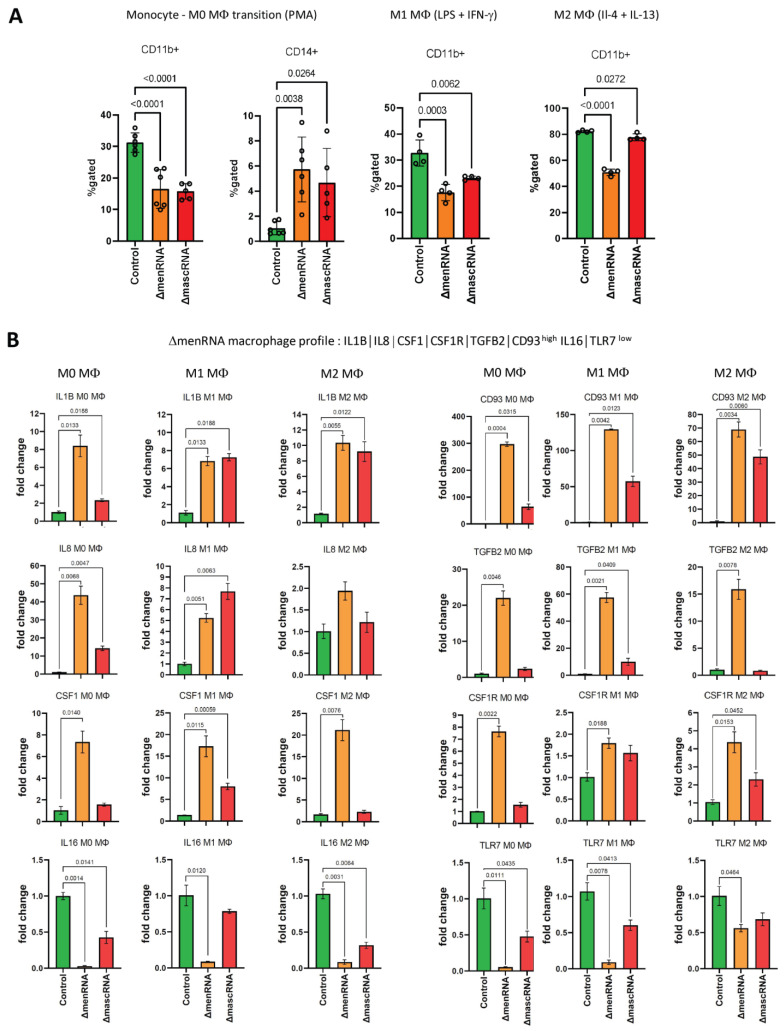

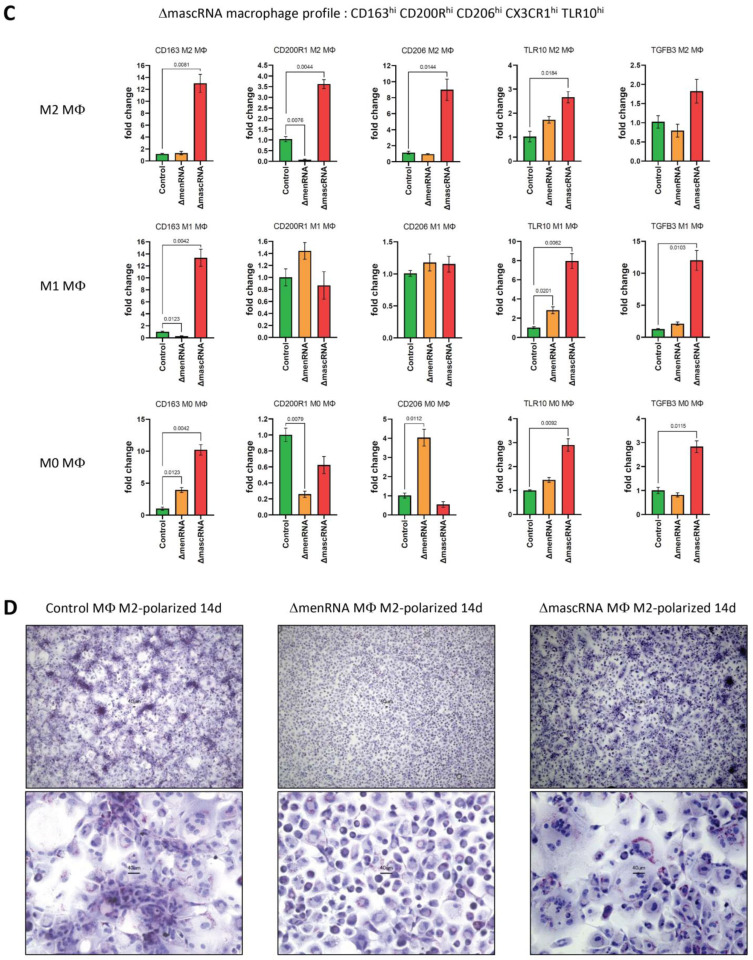
Anomalous monocyte-macrophage differentiation and polarization. Monocyte-M0 macrophage differentiation and subsequent M1/M2 macrophage were conducted as follows: First, M0 macrophages were generated by incubation of THP-1 monocyte clones for seven days, with PMA at a concentration of 100 ng/mL. Thereafter, the cells were further incubated for another seven days, either with IFN-γ at 20 ng/mL plus LPS at 100 ng/mL to induce M1 polarization or with IL-4 at 20 ng/mL plus IL-13 at 20 ng/mL to induce M2 polarization. ‘M0’ expression profiles and FACS data in panels (**A**–**C**) were obtained on day 7 of culture. The ‘M1’ and ‘M2’expression profiles in panels (**B**,**C**) were obtained on day 14 of culture. Expression profiling allowed unequivocal distinction between each of the three clones (ΔmenRNA, ΔmascRNA, controls). Panel (**A**): ΔmenRNA monocytes were incapable of normal differentiation into M0 macrophages upon PMA exposure, consistent with the grave disturbance of CD molecule expression in these monocytes (including CD11b, CD11c). In accordance with these transcription level alterations, FACS analysis identified defective transition from monocytes (both ΔmenRNA and ΔmascRNA) to M0 macrophages, as assessed by CD11b and CD14 expression. The graphs display the statistics of 5–6 independent biological samples (one-way ANOVA multiple comparisons, FACS gatings in Supplementary Figure S9B). Panel (**B**): A distinctive expression profile was characteristic of ΔmenRNA cells. It was observed in ΔmenRNA monocytes (Figure 4A–C), ΔmenRNA M0 macrophages, and preserved upon their treatment with M1 or M2 polarization protocol. This conserved pattern encompasses IL1B, CD93, TGFB2, CSF1 and its receptor CSF1R. While high pro-inflammatory IL1B expression is common to M1-polarized cells, the massive induction of CD93 or the CSF1–CSF1 receptor system [66] observed here is not commonly associated with either M1 or M2 polarized cells. Bar graphs in panels (**B**,**C**) show means ± SE from four biological replicates. The expression levels of each gene are normalized to the control clone level at the respective time, i.e., day 7 for M0 macrophages and day 14 for M1 and M2 polarized cells. Panel (**C**): Beyond M0 differentiation, another characteristic expression profile developed for ΔmascRNA cells. A “M2-prone” pattern comprising CD163^hi^ CD200R1^hi^ CD206^hi^ CX3CR1^hi^ TLR10^hi^ was observed in ΔmascRNA macrophages upon the M2 polarization protocol. This is easily distinguished from the profile of control and ΔmenRNA cells after this treatment. TLR10 was recently identified as an anti-inflammatory pattern-recognition receptor [64] and added to this “M2-prone” pattern. Panel (**D**): At the end of the 14 day-M2 polarization protocol, the morphological aspect of M2-polarized ΔmenRNA macrophages was grossly different from that of control cells and ΔmascRNA cells. At that time, ”colony-like” cell clusters became visible in both control and ΔmascRNA cultures, whereas the distribution of the M2-polarized ΔmenRNA macrophage remained essentially homogeneous. For a high-resolution version of this figure, please refer to the source file.

RNA-seq was employed as a genome-wide screening tool to detect any alterations in the protein-coding or noncoding transcriptome of wildtype vs. ΔmenRNA or ΔmascRNA cells (Figure 2). Based on the RNA-seq data, we subsequently investigated a large number of deregulated genes by qRT-PCR under different conditions (Figure 3, Figure 4, Figure 5, Figure 6, Figure 7, Figure 8 and Figure 9), in monocytes (Figure 4, Figure 5 and Figure 6) as well as the corresponding differentiated macrophage (Figure 7, Figure 8 and Figure 9) clones.

RNA-seq identified profound alterations in the baseline transcriptomes of ΔmenRNA and ΔmascRNA monocytes. These are shown in Figure 2A,B as Gene set enrichment analysis (GSEA) plots. The Supplementary Table S1/Table S2 provide FPKM details of this RNA-seq study for a large number of key deregulated transcripts. The rainbow heat maps in Figure 3, Figure 4 and Figure 5 and Figure 8 are excerpts of functional gene groups relevant for the respective chapter (e.g., innate immune sensors in Figure 3A,B). GSEA identified immune response (red dots) and cell–cell interaction (green dots)-associated biological processes enriched by genes deregulated in ΔmenRNA cells (Inset): primary immunodeficiency, cytokine-cytokine receptor interaction, JAK-STAT-signaling [18], hedgehog signaling [19,20], focal adhesion, GAP junction, extracellular matrix receptor interaction, regulation of actin cytoskeleton. For additional background information regarding these KEGG pathways, we have added Supplementary Table S3, providing all genes in the KEGG pathways which are highlighted in the Figure 2C inset.

A prominent finding was gross disbalance between multiple innate immune sensors in ΔmenRNA monocytes (Figure 3A,B, Table S1A), including cytosolic NOD-like receptors NOD2 [21] and CIITA [22], NLR-class receptors NLRC3, NLRC4, and MEFV [23,24,25], and membrane-bound Toll-like receptors (TLR1, TLR2, TLR7, TLR10). In the context of baseline induction of NOD2 and TLR2, it is notable that twist family bHLH transcription factor 2 (TWIST2) [26] was ~10-fold down in ΔmenRNA cells (Figure 3G,H) and loss of NLRC3 and TWIST2 expression in ΔmenRNA monocytes could not be rescued by LPS (Figure 3E) (compare Figure 6A). ΔmascRNA monocytes displayed induction of interferon (IFN)-induced transmembrane (IFITM) proteins [27] (Figure 3C, Table S2A). 

### 3.3. Transcription, Translation, and Epigenome Level Anomalies in Defective Monocytes

Transcription and nuclear factors and epigenome modifiers (Figure 3G–I, Table S1B/Table S2B), as well as translation factors, ribosomal proteins, and nucleic acid modifiers (Figure 3J–L, Table S1C/Table S2C), were deregulated in defective cells. At the transcriptional level, in addition to TWIST2, transcription factor (TF) GATA2 was ~4-fold-induced and GATA6 ~6-fold-suppressed (Figure 3D,E), and NFATC2 switched off in ΔmenRNA cells (Figure 3G). Further TFs and epigenome modifiers turned into disequilibrium upon LPS challenge (Figure 3H), among them histone deacetylases, lysine demethylases, and METTL methyltransferases [28]. At the level of translation, three initiation and elongation-related factors (EIF3CL, EEF1A2, CTIF) were induced, while initiation factor EIF1AY [29] and ribosomal protein RPS4Y1 were respectively 33-fold- and 45-fold-downregulated in ΔmenRNA cells (Figure 3J,K). Deregulated nucleic acid-modifying enzymes include DEAD-box helicase DDX3Y (333-fold-downregulated) and NOP2/Sun methyltransferase NSUN7 [30,31,32], undetectable in controls but robustly expressed in ΔmenRNA cells (FPKM 22) (Figure 3J, Table S1C). At the level of tRNAs, genomically or mitochondrially encoded tRNAs [31,32] and tRNA methyltransferases turned into imbalance. In ΔmascRNA cells (Figure 3L), cytosolic nucleotidase NT5C1A [33] dephosphorylating 5′ and 2′(3′)-phosphates of deoxyribonucleotides with broad substrate specificity is suppressed. Furthermore, RNA-seq identified deregulation of long noncoding RNAs (lncRNAs) and antisense (AS) RNAs in ΔmenRNA and ΔmascRNA monocytes (Table S1D/Table S2D). Thus, ΔmenRNA cells showed ~7-fold downregulation of a recently discovered novel transcript designated ‘MARCKS cis-regulating lncRNA promoter of cytokines and inflammation’ or ‘regulator of cytokines and inflammation’ (ROCKI) (Figure 4D,E, Table S1D/Table S2D). This transcript of particular interest was identified by Zhang et al. [34] through genome-wide scanning of macrophages for pairs of cis-acting lncRNAs and protein-coding genes involved in innate immunity. 

### 3.4. Excessive Inflammatory Cytokine Production by ΔmenRNA and ΔmascRNA Cells

We found massively elevated basal expression and secretion of IL-8, TNF, and IL1B in ΔmenRNA monocytes (Figure 4A,B). ΔmascRNA monocytes displayed exaggerated LPS induction of IL1A, IL1B, IL10, and ISG15 (Figure 4C). Conversely, ΔmenRNA but not ΔmascRNA macrophages displayed blunted NOS2 expression and impaired ROS production upon LPS or H_2_O_2_ challenge (Figure 6C). There were anomalies of further interleukin (IL)/receptor and TNF cytokine/receptor systems (Figure 4E–G, Table S1E/Table S2E). Receptor IL4R was ~7-fold and M2 macrophage polarization regulator IL4I1 [39,40] ~11-fold down in ΔmenRNA monocytes (Figure 4E,F). Even more pronounced was disequilibrium within the IL18 system. Receptor IL18R1 and IL18 receptor accessory protein (IL18RAP) were switched off. IL2 receptor IL2R-β (IL2RB) was ~52-fold- and IL2R-γ (IL2Rc) ~13-fold-suppressed. IL1 receptor antagonist (IL1RN) was ~12-fold and IL1 receptor associated kinase 2 (IRAK2) ~8-fold down in ΔmenRNA cells. Among ligands, IL16 [38] remained ~18-fold-suppressed, even after LPS stimulation. 

Partial “rescue” of cytokine control in ΔmenRNA monocytes was obtained by recombinant human menRNA (Figure 4D) expressed from the adenovector Ad5-menRNA (without CCA terminus), as described in Figure 1H.

‘Regulator of cytokines and inflammation’ (ROCKI) was ~7-fold- and SLFN5 (a transcriptional co-repressor of interferon (IFN) responses and antiviral restriction factor [41,42,43,44]) ~10-fold-suppressed in ΔmenRNA monocytes (Figure 4E,F), while another member (SLFN12L) of the SLFN family of IFN-induced genes was ~26-fold up. Several ADAM metallopeptidases (Table S1E/Table S2E), as well as cluster of differentiation (CD) and other leukocyte marker proteins [45,46,47,48,49,50,51,52,53,54] (Table S1F/Table S2F), were likewise deregulated, and some of them switched off in ΔmenRNA (Figure 4E,F) or ΔmascRNA cells (Figure 4G).

### 3.5. Disturbed Growth Pattern and Endothelium Interactions of ΔmenRNA Monocytes

At the cellular level, ΔmenRNA monocytes displayed an anomalous growth pattern with spontaneous cell cluster formation in liquid culture (Figure 5A) and defective endothelial adhesion under multiple flow conditions (Figure 5B). The LPS-dependent induction of cell adhesion molecules (ICAM1, VCAM1) was defective in ΔmenRNA monocytes (Figure 5C), contrasting with exacerbated induction in ΔmascRNA monocytes. ΔmenRNA monocyte-conditioned medium altered expression of multiple genes in HAEC monolayers (Figure 5D), whereas ΔmascRNA monocyte medium had no such effect. This observation of transcriptome changes in endothelial cells under influence of the ΔmenRNA monocyte secretome prompted further studies regarding indirect influence of the anomalous monocyte clones upon endothelial cell behaviour, mediated via their secretomes or via interaction between the monocytes and endothelial cells in co-cultures of both cell types in matrigel (Figure 5H,I). RNA-seq identified profound deregulation of further cell adhesion molecules in ΔmenRNA monocytes (Figure 5E,F, Table S1G), including adhesion G protein-coupled receptors [55], intercellular and neural cell adhesion molecules, integrins, vascular cell adhesion molecule, melanoma cell adhesion molecule MCAM (CD146), and endothelial cell-specific adhesion molecule (ESAM). ΔmascRNA cells also displayed anomalous cell adhesion molecule expression (Figure 5G, Table S2G), however distinct from ΔmenRNA monocytes. Neuronal growth regulator NEGR1 is ~28-fold-suppressed in ΔmenRNA but unaltered in ΔmascRNA monocytes, whereas cytoskeleton regulating NCKAP1 was ~14-fold-increased in ΔmascRNA but unaltered in ΔmenRNA cells. lncRNA SENCR, involved in maintenance of endothelial cell homeostasis, was ~12-fold down in ΔmenRNA monocytes (Table S1D), suggesting menRNA loss may lead to SENCR downregulation and cellular dysfunction in endothelial cells as well. 

### 3.6. Impact of ΔmenRNA and ΔmascRNA Monocytes upon Angiogenesis

A quantitative effect of ΔmenRNA and ΔmascRNA monocyte-conditioned media upon HAEC-based tube formation was observed in matrigel assays. Supernatant from each of the defective cell clones significantly reduced the tube number (Figure 5H). Beyond this quantitative effect of secreted factors from the cells, direct co-culture of ΔmenRNA, as well as of ΔmascRNA monocytes with the HAECs in the matrigel assay, lead to profound alterations of HAEC cell morphology (Figure 5I). In co-cultures with either of the defective monocyte clones, there was a massive increase in the size of HAECs at branch side nodes where ≥3 endothelial cells came into contact. When the defective monocytes clones were stained red before addition to the HAEC-matrigel mixture, there was significant accumulation of red cells at branch side nodes where HAEC hypertrophy occurred (Figure 5I). Consistent with these observations, multiple growth and angiogenesis-associated factors and chemokines [56,57] were in disequilibrium in ΔmenRNA (Figure 5J,K, Table S1H) and ΔmascRNA monocytes (Figure 5L, Table S2H).

### 3.7. Response of ΔmenRNA and ΔmascRNA Macrophages to Human-Pathogenic Viruses

PMA-based in vitro differentiation of ΔmenRNA and ΔmascRNA monocytes into adherent macrophages rendered the cells susceptible to transduction by recombinant adenovirus-expressing GFP (Supplementary Figure S8A). Similarly, ΔmenRNA and ΔmascRNA macrophages became transducible with human coxsackievirus B3 (CVB3) and displayed an immune response to the internalized CVB3 ssRNA genome in the absence of active CVB3 virus replication (Supplementary Figure S8B). In ΔmenRNA macrophages, without LPS stimulation or adenovirus transduction, IL8 expression was >100-fold-induced compared to controls (Figure 6A). Adenovirus resulted in a moderate IL8 decrease, while control cells retained extremely low IL8 expression upon transduction. Several other genes had significantly higher baseline expression in ΔmenRNA macrophages, either *without* response to virus (TNF, SLFN12L, NOD2, TLR2, Il1B), or with significant *further* induction upon exposure (RIG-like receptors RLR1 and RLR2, ISG 15, TGFB2). Most conspicuous was a complete shutdown of genes suppressed in ΔmenRNA monocytes (Figure 3A–E, Figure 4E,F) already before their transformation into macrophages: NOD-like innate immune genes NLRC3 and MEFV, IL2 receptor subunits β and γ, transcription factor TWIST2, and NFATC2. None of these genes fully silenced in macrophages could be ’rescued’ by adenovirus exposure (Figure 6A), similar to findings in LPS-stimulated monocytes (Figure 3A–E, Figure 4E,F). Figure 6B shows the response of ΔmenRNA macrophages to CVB3. Regarding IL8, their response to CVB3 with further IL8 induction beyond their already very high baseline level was opposite to their anti-adenovirus reaction. ISG15 and Il1B responded similarly to CVB3 and RR5, while IRF7 and IRF9 expression were downregulated by CVB3 only. Inducible NO synthase (NOS2) expression, as well as ROS production upon LPS or H_2_O_2_ challenge, was significantly reduced in ΔmenRNA macrophages (Figure 6C). Figure 6D summarizes key defects of the antiviral response.

### 3.8. menRNA Deletion Critically Disturbs Scavenger Receptor Expression and oxLDL Uptake

ΔmenRNA macrophages display loss of oxLDL uptake, consistent with their loss of scavenger receptor expression (Figure 7A–C) [58]. ΔmenRNA cells showed further anomalies regarding receptors involved in phagocytosis: Fcγ receptors (FcγRs) [59,60] and complement receptors (CRs) (Figure 8).

### 3.9. Defective Monocyte–Macrophage Transition and Polarization of ΔmenRNA and ΔmascRNA Cells

ΔmenRNA monocytes are unable to normally differentiate into M0 macrophages upon PMA exposure (Figure 9A). This is consistent with disturbances of CD molecule expression in these cells, including CD11b (ITGAM), CD11c (ITGAX), and CD93 [61,62,63]. 

Beyond monocyte–macrophage transition, there was defective M1/M2 polarization of ΔmenRNA and ΔmascRNA cells. Rather simple expression profiles allowed distinction between ΔmascRNA and ΔmenRNA macrophages and controls (Figure 9B,C). An “M2-like” pattern CD163^hi^ CD200R^hi^ CD206^hi^ TGFB3^hi^ TLR10^hi^ was observed in ΔmascRNA cells [64]. A profile involving IL1B, CD93, TGFB2, TLR7, CSF1, and its receptor CSF1R [65,66] unequivocally characterizes ΔmenRNA monocytes-macrophages and is preserved through polarization. Upon prolonged culture, the morphological aspect of M2-polarized ΔmenRNA cell cultures was clearly distinct from control and ΔmascRNA cells (Figure 9D).

## 4. Discussion

*NEAT1* and *MALAT1* are involved in two fields of medicine (cardiovascular and malignant diseases), linked to some degree by common immune-related mechanisms [5]. A unified concept to explain the connections of this genomic region to apparently diverse diseases may be derived from recent observations regarding immunoregulatory functions of transcripts from this cluster, encompassing the current study assigning novel immune functions to both tRNA-like transcripts. Despite differences in their specific regulatory properties, it appears that the fundamental principle of ‘employing’ this peculiar type of small ncRNAs was evolutionarily advantageous.

Prior studies of the *NEAT1-MALAT1* cluster: We reported suppression of lncRNA *NEAT1* in circulating immune cells of post-MI patients. In mice lacking lncRNAs *NEAT1* [1] or *MALAT1* [2,3,4], we observed immune disturbances rendering the immune system unstable and highly vulnerable to immune stress. *MALAT1*^−/+^ ApoE^−/−^ mice suffered accelerated atherosclerosis despite normal diet compared to ApoE^−/−^ mice. *NEAT1*^−/−^ mice showed anomalous T cell and monocyte-macrophage differentiation and systemic inflammation. *NEAT1* promotes inflammasome activation in macrophages, regulates M2 polarization [69], and influences Th17/CD4^+^ T cell differentiation. *NEAT1* knockdown induces a tolerogenic phenotype in dendritic cells by inhibiting NLRP3 inflammasome activation. Further *NEAT1* or *MALAT1*-related anomalies were reported in non-immune cell types: cardiomyocytes, endothelial cells, and smooth muscle cells, where an HDAC9-*MALAT1*-BRG1 complex mediates dysfunction. Clinically, *NEAT1* correlated with increased exacerbation risk, severity, and inflammation in asthma [70] and with worse disease condition and poor recurrence-free survival in acute ischemic stroke. *NEAT1* is elevated in peripheral blood cells of Parkinson’s disease patients [71] and abnormally expressed in a wide variety of human cancers [72]. 

Functional dissection of the *NEAT1*-menRNA system: A subset of lncRNAs, termed architectural RNAs (arcRNAs), function in formation and maintenance of phase-separated membraneless organelles. In the crowded intracellular environment, these are important forms of compartmentalization. Thus, *NEAT1* is a well-characterized arcRNA acting as an essential scaffold of paraspeckle nuclear bodies. 

In contrast, no biological function of menRNA independent of its precursor *NEAT1* have been described, while a few studies have already addressed mascRNA in that regard. Our functional data were obtained by CRISPR-Cas9-mediated highly selective disruption of the menRNA sequence 21 nt downstream of the 3′-terminus MEN-β. Importantly, this leaves the regular triple-helix formation at the 3′-end of MEN-β unaffected, which is essential for its stabilization. Consistent with the current model of the *NEAT1*-menRNA system, the CRISPR-Cas9-generated ΔmenRNA cells displayed no alteration of MEN-β or MEN-ε expression levels. 

Although the very low abundance of menRNA generates experimental challenges, specificity of the observed effects of menRNA disruption may be derived by synopsis of the following findings: 1. Northern analysis detects menRNA in the experimental model cells (wildtype and CRISPR-Cas9 control THP-1 cells); 2, Sequencing confirms exactly stable deletion of essentially all of the menRNA-generating sequence; 3. Consistent with this, no menRNA signal is detected in the ΔmenRNA clone; 4. This deletion is clearly separate by 21 nt from the upstream MEN-β transcript with its 3′ triple-helix terminus; 5. The cellular levels of MEN-β and MEN-ε appear unaffected by the menRNA disruption.

Our study may be considered an extension of previous pioneering work by Yamazaki et al. in identifying the key functional domains of MEN-β through CRIPSR-Cas9-based deletion mapping [12]. While covering the entire length of MEN-β, that study has not reported on deletions downstream of the 3′-terminal A-rich motif essential for triple-helix formation and stabilization of MEN-β. 

Unless the RNaseZ cleavage site at the 3′-end of menRNA (deleted in the ΔmenRNA clone) has some unknown function other than supporting menRNA formation, the data indicate deep impact of selective menRNA disruption upon innate immunity and macrophage functions. While the current dataset does not allow to derive a mechanism of action for menRNA, the chosen experimental design should allow assignment of the observed cellular anomalies (discussed below) to menRNA per se.

*MALAT1*-independent functions of mascRNA: After a report that mascRNA is involved in cardiovascular innate immunity [3], Sun et al. conducted an in-depth study demonstrating that mascRNA differentially regulates TLR-induced proinflammatory and antiviral responses [9]. Lu et al. showed that mascRNA promotes global protein translation, uncovering another role of mascRNA that is independent of *MALAT1* [10].

Our CRISPR-Cas9-based editing of mascRNA essentially confirmed the prior reports on immunomodulating functions of mascRNA. Permanent mascRNA depletion obviates the need to repetitively add ASOs or siRNAs to keep mascRNA down. This experimental feature significantly simplified long-term experiments (Figure 5, Figure 7 and Figure 9), revealing imbalance of cell–cell interactions system and angiogenesis, phagocytosis-related genes, and differentiation and polarization of ΔmascRNA monocytes and macrophages.

Critical defects of innate immune sensing: One key finding in ΔmenRNA cells was deregulation of membrane-bound (TLR2) and cytosolic immune sensors (Figure 3). NOD2 and CIITA were massively induced, while NLR-class receptors NLRC3 and MEFV were shut off in ΔmenRNA cells and could be neither be rescued by LPS nor virus exposure. TWIST2, a critical regulator of cytokines in human monocyte-derived macrophages [26], NFATC2 which translocates to the nucleus upon T cell receptor stimulation, and IL2 receptor subunits β and γ were likewise shut down and not rescuable. Further, there was massive downregulation of lncRNA ROCKI, apparently resulting from mere deletion of the small ‘menRNA’ sequence from an otherwise intact ~23 kb *NEAT1*. An important recent study [34] discovered that ROCKI is a master regulator of inflammatory responses. Beyond the transcription level, ΔmenRNA and ΔmascRNA cells displayed major changes of their epigenomes (Figure 3).

Loss of inflammatory cytokine control: ΔmenRNA and ΔmascRNA cells display anomalous antiviral responses (Figure 5). Even in absence of infectious agents or other immune challenges, however, ΔmenRNA cells display massive inductions of IL8 and TNF (Figure 4). Regarding IL8, clinical observational studies suggest a critical role of IL8 in cardiovascular diseases [73,74] and stroke [75]. IL8 is mechanistically involved in the innate immune response by TLR4 signaling, induces neutrophil extracellular traps (NETs) via activation NFκB signaling [76], mediates hyper-signaling in aortic aneurysms, and is involved in endothelial adhesion [77]. With regard to IL8, our prior transcriptome analysis of circulating immune cells from post-MI patients [1] is of interest. IL8 was the most decisively upregulated gene post-MI, contrary to an extremely low level in healthy subjects, although all patients were receiving state-of-the-art post-MI pharmacological treatment. In that study, the long primary lncRNA transcript *NEAT1* was significantly suppressed in post-MI PBMCs. The new data from ΔmenRNA cells are consistent with the assumption that reduction of the menRNA level in PBMCs (consecutive to or independent of the observed *NEAT1* reduction) directly contributes to IL8 induction. Further, the marked IL1B induction in post-MI PBMCs would parallel the high baseline IL1B expression in ΔmenRNA cells. It would have been most interesting to directly measure menRNA levels in the post-MI PBMCs. Due to the complex secondary and tertiary structure of mature menRNA this would have required, however, Northern blot analyses for which there was insufficient RNA available in that former post-MI study. For future translational studies, our approach to study menRNA and mascRNA directly suggests new avenues since their highly dynamic levels may be more closely related to clinical parameters and clinical course than those of their nuclear precursors.

Imbalance of angiogenic factors and chemokine systems: Excessive inflammatory cytokine production appears as one downstream consequence of defective immune sensing. Imbalance of angiogenic factors, chemokines, and cell–cell adhesion molecules may be considered as further sequelae of dysfunction at the sensor level. It is not unexpected that the anomalies at this level should have far-reaching impact upon the cells’ biological behavior. Thus, irrespective of differences between ΔmenRNA and ΔmascRNA cells, both significantly decreased tube formation in matrigel assay and displayed profound deregulation of cell–cell adhesion molecules (Figure 5). In the case of ΔmenRNA cells, the latter were immediately apparent by their anomalous growth in clusters and their defective endothelial flow adhesion to HAECs. While de-repression of multiple growth/angiogenetic factors appears directly linked to anomalous endothelial cell growth and morphology in the monocyte-endothelial cell co-cultures, this imbalance of growth/angiogenetic factors apparently does not support effective tube formation, but instead hinders it.

Defective foam cell formation and macrophage polarization: This occurs in the context of large-scale disturbance of the cells’ immune sensors from NOD-like and Toll-like (Figure 3) to phagocytosis-related receptors (Figure 7 and Figure 8). Overall, the cells are unable to adequately respond to immune challenges including LPS, viruses, oxLDL, and polarization cytokines (Figure 9). Defective interaction with endothelium and angiogenesis-promoting matrix may be added to their anomalous relationship with the environment (Figure 5).

**Figure 10:**
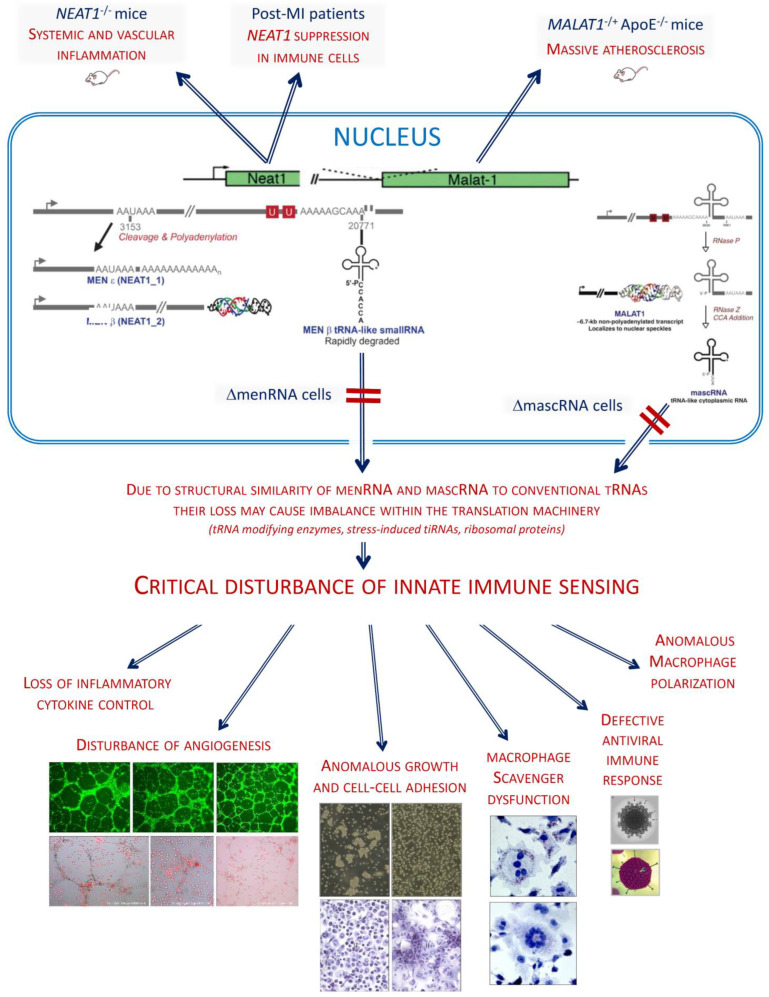
Working hypothesis. Analysis of monocytes-macrophages with narrowly targeted deletions of the tRNA-like menRNA and mascRNA suggests they are critical components required for balanced function of innate immunity. The grave disturbance of multiple immune sensor systems with complex downstream sequelae may be addressed by a working hypothesis invoking a primordial defect linked to the peculiar structure of menRNA and mascRNA. They closely resemble common tRNAs, suggesting they also interact with the ribosomal machinery. Accordingly, ablation of nucleus-to-cytosol supply of menRNA or mascRNA would cause imbalance within the translation system, consistent with the observed deregulation of multiple translation factors, tRNAs, tiRNAs, and ribosomal proteins in defective cells. Delineation of a specific molecular mechanism to prove this hypothesis is far beyond the scope of this study, which was designed to first identify any distinct biological functions of menRNA and to provide further data on mascRNA functions. Considering the minute and narrowly targeted genetic interventions employed, the extent of disturbances from key innate immune sensors to complex downstream sequelae is unexpected. Beyond primary defects at sensor level, the grave loss of cytokine control, angiogenesis-modifying effects of ΔmenRNA and ΔmascRNA monocytes, and defects of key ΔmenRNA and ΔmascRNA macrophage functions constitute the first known biological functionalities of these cytosolic molecules. From this starting point, it appears warranted to generate murine models with cell type-specific inducible knockout of menRNA or mascRNA only to further elucidate their functions in disease models. For future translational studies, our approach to study menRNA and mascRNA directly suggests new avenues since their highly dynamic levels may be more closely related to clinical parameters and clinical course than those of their nuclear precursors. They may also have value as therapeutic targets for pharmacological intervention since they are more easily accessible than their extremely complex nuclear-located precursor molecules. A prior observation that recombinant mascRNA [3] abolishes virus replication in cardiomyocytes does suggest potential of mascRNA- and menRNA-targeting interventions. From an evolutionary perspective, the *NEAT1-MALAT1* genomic region appears as a highly integrated RNA processing circuitry critically contributing to immune homeostasis. Its components MEN-β, MEN-ε, menRNA, *MALAT1*, *TALAM1*, and mascRNA are obviously set for well-balanced interactions with each other. Genetic ablation of any element therefore leads to major dysfunction.

Loss of nucleus-to-cytosol supply of menRNA or mascRNA: As both menRNA and mascRNA are continuously exported from nucleus to cytosol under normal conditions (Figure 10), changes in the translational/ribosomal apparatus may be linked to the complete loss of nucleus-to-cytosol supply of these tRNA-like molecules in the defective cells. Regarding definition of an underlying molecular mechanism, this is beyond the scope of the current study with an experimental strategy clearly focused upon identification of novel biological roles for mascRNA (and any such roles for menRNA) at the cellular and cell–cell interaction level. Given the extreme complexity of tRNA biology [15,78,79,80,81,82], a different experimental strategy will be needed to address the molecular level. 

Notably, mascRNA displays very high steady-state levels within immune cells, while other cell types are essentially devoid of it, although they do express precursor *MALAT1* at normal levels. Sun et al. conducted an in-depth study demonstrating that mascRNA differentially regulates TLR-induced proinflammatory and antiviral responses [9]. While the exact molecular function of the high mascRNA levels in immune cells remains undefined in humans and other species carrying *MALAT1*-like genomic loci [10], key importance for immune homeostasis was highly likely by inference and is definitely confirmed by the present data. In this context, a recent study by Lu et al. [10] is of interest, showing that mascRNA binds directly to multi-tRNA synthetase complex component glutaminyl-tRNA synthetase and promotes global protein translation and cell proliferation by positively regulating QARS protein levels. This is consistent with our working hypothesis that mascRNA and menRNA interact with the ribosomal machinery as a consequence of their tRNA-like structure. 

In the case of menRNA, on the other hand, the steady-state levels in immune cells are very low. This may be entirely due, however, to the known CCACCA-tagging of menRNA for rapid degradation [15]. menRNA’s primary nucleus-to-cytosol supply rate may be similar to that of mascRNA, which just remains stable as it is not CCACCA-tagged. ΔmenRNA cells display major disequilibrium at the tRNA/translational level with induction of initiation and elongation factors EIF3CL, EEF1A2 and CTIF, whereas translation initiation factor EIF1AY [29] was switched off. Further, there was deregulation of tRNAs, tRNA methyltransferases (TRMT61, TRMT2B), and RNA methyltransferase NSUN7. 

Pioneering recent studies discovered hitherto unknown immune functions of tRNAs and their enzymatic processing products and may contribute to explain our current findings regarding the impact of menRNA and mascRNA ablation. tRNA products encompass tRNA-derived small RNAs (tsRNAs), tRNA-derived stress-induced RNAs (tiRNAs), and tRNA-derived fragments (tRFs). Pereira et al. [56] found that m(5)U54 tRNA hypomodification, from lack of methyltransferase TRMT2A, drives tsRNAs generation. As similar tRNA methyltransferases are deregulated in ΔmenRNA and ΔmascRNA cells, this may change tsRNA generation therein. 

Yue et al. [57] found SLFN2 protection of tRNAs from stress-induced cleavage to be essential for T cell-mediated immunity. SLFN2 binds tRNAs and protects them from cleavage by the ribonuclease angiogenin (~12-fold-induced in ΔmascRNA cells) [57]. SLFN2-deficient T cells display accumulation of stress-induced tiRNAs which inhibit translation and promote stress-granule formation. They found IL2R-β and IL2R-γ fail to be translationally upregulated after T cell receptor stimulation, while we observed ~52-fold IL2R-β and ~13-fold IL2R-γ suppression in ΔmenRNA monocytes upon LPS stimulation. In these cells, SLFN family member SLFN12L was ~31-fold-induced, whereas SLNF5 was ~10-fold-suppressed, compared to controls. Yue et al. showed ROS trigger an oxidative stress response leading to translation repression which is countered by SLFN2. In ΔmenRNA monocytes, we found LPS-stimulated ROS production as well as NOS2 expression are blunted, suggesting disturbance of a regulatory circuit encompassing menRNA, SLFN family members [41,83], and NO and ROS biosynthetic enzymes in these defective cells.

Genetic variability of the *NEAT1-MALAT1* genomic region in humans: Since multiple data suggest inflammation control functions of the *NEAT1*-*MALAT1* cluster, we investigated variability of this region within a cohort of 7.500 individuals from Central Europe (Supplementary Figures S5 and S6A,B). One rare MALAT1 SNP (MAF = 0.01) in the MALAT1 promotor was associated (*p* = 0.0062) with systemic low-level inflammation (CRP > 3.0 mg/L) (Supplementary Figure S7). The current study was not designed to identify possible phenotype associations with mascRNA or menRNA sequence variants known to occur in humans (Supplementary Figure S4). Given the functional sensitivity of the tRNA-like sequences to point mutations, it seems worthwhile to obtain full sequences in patient cohorts suffering from atherosclerosis and other disorders associated with chronic low-level inflammation.

## 5. Conclusions

Beyond prior work in mice documenting immune function of the *NEAT1-MALAT1* region, the current study identifies menRNA and mascRNA as novel components of innate immunity with deep impact upon cytokine regulation, immune cell–endothelium interactions, angiogenesis, and macrophage formation and functions. These tRNA-like transcripts appear to be prototypes of a class of ncRNAs distinct from other small transcripts (miRNAs, siRNAs) by biosynthetic pathway (enzymatic excision from lncRNAs) and intracellular kinetics, suggesting a novel link for the apparent relevance of the *NEAT1-MALAT1* cluster in cardiovascular and neoplastic diseases. 

The *NEAT1-MALAT1* region has emerged as a highly integrated RNA processing circuitry. Its components MEN-β, MEN-ε, menRNA, *MALAT1*, *TALAM1*, and mascRNA are set for well-balanced interactions with each other, and ablation of any element appears to lead to major cellular and systemic dysfunction. In this context, our study has addressed several functional aspects of menRNA and mascRA at the level of cells and cell-to-cell interactions. Current knowledge, however, does not yet allow definition of the inner workings of this genomic region at the subcellular molecular level. 

For future translational studies, our approach to study menRNA and mascRNA directly may suggest new avenues since their highly dynamic levels may be more closely related to clinical parameters and clinical course than those of their nuclear precursors. They could possibly also have value as therapeutic targets for pharmacological intervention since they are more easily accessible than their extremely complex nuclear-located precursor molecules. 

## Figures and Tables

**Table 1:** sgRNAs employed for CRISPR-Cas9 deletion experiments.

G1_human_mascRNA	taatacgactcactataGGTTGGCACTCCTGGTTTCCgttttagagctagaaatagc
G5_human_mascRNA	taatacgactcactataGGACGGGGTTCAAATCCCTGgttttagagctagaaatagc
G9_human_menRNA	taatacgactcactataGGGGCACGTCCAGCACGGCTgttttagagctagaaatagc
G10_human_menRNA	taatacgactcactataGGTCCAGCACGGCTGGGCCGgttttagagctagaaatagc
universal reverse	AGCACCGACTCGGTGCCACT

## Data Availability

The RNA-sequencing (RNA-seq) data reported in this paper have been uploaded to the EMBL-EBI (European Molecular Biology Laboratory—European Bioinformatics Institute) https://www.ebi.ac.uk/ (last accessed on 1 November 2022) under project ID PRJEB55408 (runs-2022-08-16report.csv, samples-2022-08-16report.csv, study-2022-08-16report.csv).

## Supplementary Materials

Further details on SHIP cohorts, methods and associated references, and supplementary figures and tables are provided in the online Supplement which can be downloaded at: https://www.mdpi.com/article/10.3390/cells11243970/s1. Figure S1A: CRISPR-Cas9 target region to generate ΔmenRNA cells; Figure S1B: CRISPR-Cas9 target region to generate ΔmascRNA cells; Figure S1C: Recombinant expression and Northern blot analysis of menRNA; Figure S2: Structure prediction for residual sequences in ΔmenRNA and ΔmascRNA cells; Figure S3: In vitro transcription of single-guide RNAs; Figure S4: Known sequence polymorphisms in human menRNA and mascRNA sequences; Figure S5: Outline regarding SNPs analyses in SHIP cohorts; Figure S6A: Positions of MALAT1 region SNPs analyzed in SHIP cohorts; Figure S6B: Positions of NEAT1 region SNPs analyzed in SHIP cohorts; Figure S7: Human population genetic data; Figure S8A: Transduction of recombinant GFP-expressing adenovirus into CRISPR-Cas9-generated defective human macrophages; Figure S8B: Transduction of human coxsackievirus B3 into CRISPR-Cas9-generated defective human macrophages; Figure S9A: Anomalous expression of innate immune sensor molecules and interferon-induced genes in ΔmenRNA and ΔmascRNA macrophages; Figure S9B: FACS analysis of ΔmenRNA and ΔmascRNA monocyte to M0 macrophage transition; Table S1: RNA-seq FPKM data for ΔmenRNA monocytes; Table S2: RNA-seq FPKM data for ΔmascRNA monocytes; Table S3: Genes in the KEGG pathways displayed in Figure 2C.
