# Supplementary material for: tRNA-like Transcripts from the NEAT1-MALAT1 Genomic Region Critically Influence Human Innate Immunity and Macrophage Functions
Source: Cells. 2022 Dec 8;11(24):3970. doi: 10.3390/cells11243970 (PMC9777231; doi:10.3390/cells11243970)
Supplement: Supplementary file 1 [file cells-11-03970-s001.zip › cells-1977241-supplementary.pdf]

SUPPLEMENTAL MATERIAL

tRNA-like Transcripts from the *NEAT1-MALAT1* Genomic Region Critically Influence Human Innate Immunity and Macrophage Functions

Martina Gast [1,2,†], Vanasa Nageswaran [1,3,†], Andreas W. Kuss [4,†], Ana Tzvetkova [4,5], Xiaomin Wang [1], Liliana H. Mochmann [1], Pegah Ramezani Rad [1], Stefan Weiss [4,6], Stefan Simm [5], Tanja Zeller [7,8], Henry Voelzke [6,9], Wolfgang Hoffmann [6,9], Uwe Völker [4,6], Stefan B. Felix [6,10], Marcus Dörr [6,10], Antje Beling [2,11,12], Carsten Skurk [1,2], David-Manuel Leistner [1,2,12], Bernhard H. Rauch [6,13,14], Tetsuro Hirose [15], Bettina Heidecker [1], Karin Klingel [16], Shinichi Nakagawa [17,18], Wolfram C. Poller [19,20], Filip K. Swirski [19,20], Arash Haghikia [1,2,12,‡] and Wolfgang Poller [1,2,21,*,‡]

1 Department of Cardiology, Campus Benjamin Franklin, Charité—Universitätsmedizin Berlin, Corporate Member of Freie Universität Berlin, Humboldt-Universität zu Berlin, Berlin Institute of Health, 12200 Berlin, Germany
2 German Center for Cardiovascular Research (DZHK), Site Berlin, 12200 Berlin, Germany
3 Institute for Chemistry and Biochemistry, Freie Universität Berlin, 12200 Berlin, Germany
4 Department of Functional Genomics, Interfaculty Institute of Genetics and Functional Genomics, University Medicine Greifswald, 17475 Greifswald, Germany
5 Institute of Bioinformatics, University Medicine Greifswald, 17475 Greifswald, Germany
6 German Center for Cardiovascular Research (DZHK), Site Greifswald, 17487 Greifswald, Germany
7 University Center of Cardiovascular Science, University Heart and Vascular Center, 20246 Hamburg, Germany
8 German Center for Cardiovascular Research (DZHK), Site Hamburg/Lübeck/Kiel, 20246 Hamburg, Germany
9 Institute for Community Medicine, University Medicine Greifswald, 17475 Greifswald, Germany
10 Department of Cardiology, University Medicine Greifswald, 17475 Greifswald, Germany
11 Institute for Biochemistry, Charité—Universitätsmedizin Berlin, Corporate Member of Freie Universität Berlin, Humboldt-Universität zu Berlin, Berlin Institute of Health, 10178 Berlin, Germany
12 Berlin Institute of Health (BIH), 10178 Berlin, Germany
13 Institute for Pharmacology, University Medicine Greifswald, 17487 Greifswald, Germany
14 Department Human Medicine, Section Pharmacology and Toxicology, Carl von Ossietzky Universität, 26129 Oldenburg, Germany
15 Graduate School of Frontier Biosciences, Osaka University, 1-3 Yamadaoka, Suita 565-0871, Japan
16 Institute for Pathology and Neuropathology, Department of Pathology, University Hospital Tübingen, 72076 Tübingen, Germany
17 RNA Biology Laboratory, RIKEN Advanced Research Institute, Wako, Saitama 351-0198, Japan
18 Faculty of Pharmaceutical Sciences, Hokkaido University, Sapporo 060-0812, Japan
19 Center for Systems Biology, Massachusetts General Hospital, Harvard Medical School, Boston, MA 02114, USA
20 Cardiovascular Research Institute, Icahn School of Medicine at Mount Sinai, New York, NY 10029, USA
21 Berlin-Brandenburg Center for Regenerative Therapies (BCRT), Charité—Universitätsmedizin Berlin, Corporate Member of Freie Universität Berlin, Humboldt-Universität zu Berlin, Berlin Institute of Health, 13353 Berlin, Germany
* Correspondence: pollerwolfgang1@gmail.com
† These authors contributed equally to this work as first authors.
‡ These authors contributed equally to this work as last authors.

For high resolution versions of the supplemental figures please refer to the Supplementary Information source file.

Details of patient cohorts and experimental methods

For human studies approval was granted by the institutional ethics review board and the regulatory authorities. Investigation of human tissues was conform to the principles in the Declaration of Helsinki. Informed consent was given prior to inclusion of people in the study.

SHIP population study and cohorts

SHIP samples were genotyped using the *Affymetrix Genome-Wide Human SNP Array 6.0*. Genotyping of samples within SHIP-TREND was obtained in two batches using *Illumina Infinium HumanOmni2.5 BeadChip* and the *Illumina Infinium Global Screening Array*, respectively. Genotypes were determined using *Birdseed 2* for SHIP and the *GenomeStudio 2.0 Genotyping Module* (*GenCall algorithm*) for SHIP-TREND. Standard genotype quality control was performed excluding arrays < 92% sample call rate for SHIP and <94% sample call rate for SHIP-TREND and duplicates (based on estimated IBD), mismatches between reported and genotyped sex, genetic PCA outliers, and arrays with extreme heterozygosity. Variants with call rate < 0.95 and a Hardy-Weinberg equilibrium p-value < 0.0001 were removed before imputation. Pre-phasing and Imputation of genotypes was performed with the Eagle and Minimac3 software to HRC reference v1.1 panel on Michigan Imputation Server v1.0.1 (https://imputationserver.sph.umich.edu/).

CRISPR-Cas9 experiments

THP-1 cells were grown in RPMI medium containing FCS (10% [v/v]) and penicillin/streptomycin (50 I.U./50 µg/ml). Parts of the menRNA and mascRNA regions were deleted from THP-1 cells using an adaptation of the CRISPR/Cas9 protocol described in Gundry *et al*. 2016. Protospacer sequences for each target gene were identified using the CRISPRscan scoring algorithm [www.crisprscan.org (Moreno-Mateos *et al.*)]. Extensive target searches employing http://www.crisprscan.com/, http://crispor.tefor.net/, and https://cctop.cos.uni-heidelberg.de identified no off-targets of potential relevance. DNA templates for single guide RNAs (sgRNAs) were generated by PCR (KAPA HiFi HotStart ReadyMix PCR Kit) using the pX458 plasmid containing the sgRNA scaffold sequence and using the following primers:

G1_human_mascRNA	taatacgactcactataGGTTGGCACTCCTGGTTTCCgttttagagctagaaatagc
G5_human_mascRNA	taatacgactcactataGGACGGGGTTCAAATCCCTGgttttagagctagaaatagc
G9_human_menRNA	taatacgactcactataGGGGCACGTCCAGCACGGCTgttttagagctagaaatagc
G10_human_menRNA	taatacgactcactataGGTCCAGCACGGCTGGGCCGgttttagagctagaaatagc
universal reverse	AGCACCGACTCGGTGCCACT

PCR products were used to generate sgRNAs by *in vitro* transcription using HiScribe T7 High Yield RNA Synthesis Kit. 0.5µg of purified sgRNA was incubated with Cas9 protein (1 µg; PNA Bio) for 15-20 min at room temperature. $2x10^5$ THP-1 cells were electroporated with the sgRNA/Cas9 complex using the Neon Transfection System at 1400 V, 20 ms, and one pulse. For the small RNA-deficient cell lines, two sgRNAs were selected at either end of the target sequence to delete the region in between. Deletion of *mascRNA* and *menRNA* was confirmed by PCR with the following primer pairs:

CRISPR_hu-masc_fw	CGTATTGTTTTCTCAGGTTTTGC
CRISPR_hu-masc_rev	ACCTCCCCAAAACTCCAAGA
CRISPR_hu-men_fw	TCTGTGAAAGAGTGAGCAGGA
CRISPR_hu-masc_rev	CCCAATGCTACCCCTCTAGG

Single-cell clones were generated by single-cell plating of the parental cell line. Gene deletions in the single-cell clones were confirmed by sequencing and proved to be stable over >25 passages so far.

Cell culture studies

Human monocyte cultures

Human THP-1 cells were cultured in RPMI 1640 medium with 10% fetal bovine serum, 2 mM L-glutamine, 100 U/mL penicillin and 100 µg/mL streptomycin. For gene expression analysis of wildtype (WT) and CRISPR-Cas9 targeted THP-1 cells under immune challenge, cells were stimulated with 100 ng/ml LPS, 10 ng/ml LPS 1 µg/ml, 1 ng/ml LPS, or 1 µg/ml Concanavalin A (Con A). After 24 h, RNA was isolated by TRIzol/Chloroform.

THP-1 monocyte adhesion to flow-primed human aortic endothelial cells

For analysis of WT and CRISPR-Cas9 targeted THP-1 monocytes adhesion to flow-primed endothelial cells, primary human aortic endothelial cells (HAECs) were cultured in Endothelial Growth Medium-2 with 10% fetal bovine serum, 100 U/mL penicillin and 100 µg/mL streptomycin and seeded to confluence in µ-Slide y-shaped ibiTreat chambers . Endothelial cells were exposed to unidirectional flow (20 dyn/cm^2) for 48 h using yellow/green perfusion sets prior to the experiment. THP-1 monocytes were labelled with 1,1'-dioctadecyl-3,3,3',3'-tetramethylindocarbocyanine (DiI) for 15 min at 37°C, respectively, and after three times wahsing 1 × 10^6 labelled cells were added to the flow reservoir for 30 min. After flow-termination, non-adherent cells were gently washed out with PBS, then cells were fixed with 4% paraformaldehyde and the number of cells adhering to both the straight and branched channel regions was assessed by fluorescence phase-contrast microscope quantified using ImageJ Software.

Tube formation angiogenesis assay

(a) Conditioned media transfer experiment: Tube formation by HAECs treated with ΔmenRNA and ΔmascRNA monocyte-conditioned media was assayed on reduced growth factor basement membrane extract (BME) in 96-well tissue cultured-treated clear-bottom plates (15 × 10^3 cells per well). Briefly, 60 µl of ice-cold BME was added per well and incubated at 37°C and 5% CO_2 for 30 min to allow gel formation. HAECs were plated on top of the gelled BME at a density of 1.5 x 10^4 cells/well in 50 µl culture medium followed by transfer of 50 µl of monocyte-conditioned media of both ΔmenRNA and ΔmascRNA and incubated further for 24 h. After tube formation was observed, endothelial cells were stained by adding 50 µl of 6 µM Calcein AM solution per well for 15 min at 37°C. Tubular network of the cells was captured using a fluorescent inverted microscope and quantified by ImageJ Software.

(b) Monocyte-HAEC co-cultures in matrigel: Endothelial tube formation in reduced growth factor matrigel was performed similarly as described above. Here, direct DiI-labeled co-cultured ΔmenRNA and ΔmascRNA monocytes at a cell density of 5 x 10^3 per well together with HAECs (1.5 x 10^4 cells/well) were added on the BME gel and cultured for 24 h at 37°C and 5% CO^2 to form tubular networks.

Reactive oxygen species assay

Intracellular ROS production in WT and CRISPR-Cas9 targeted THP-1 macrophages was determined as previously described using 2',7'-dichlorodihydrofluorescein diacetate (H2DCFDA). Upon cleavage of the acetate groups by intracellular esterases, the cell-permeant H_2DCFDA is retained within the cells and easily oxidized to the highly fluorescent 2′,7′-dichlorofluorescin (DCF) in response to ROS production/oxidative stress. Control and CRISPR/Cas9-targeted THP-1 cells were cultured in black 96 wells (overnight treatment with 0,1 µM PMA) before LPS addition for 24h. After washing with HBSS, cells were incubated with 5 µM H2DCFDA (Molecular Probes) in HBSS for 1 h at 37 °C. Cells were washed again and ROS production was induced by 400 µM H_2O_2. Fluorescence intensity was quantified every 10 min (excitation 485 nm, emission 535 nm) at 37°C using a fluorescence plate reader (Tecan).

Cytokine measurements

Conditioned cell culture media of wildtype (WT) and CRISPR-Cas9 targeted THP-1 cells were tested for IFNγ, TNF, IL6, MCP1, IL10, and IL12p70 by use of *Mouse Inflammation Cytometric Bead Assay* (CBA) (*BD Biosciences, Heidelberg, Germany*) on a *FACS CantoII flow cytometer* (*BD Biosciences*) according to the manufacturer's protocol.

Cell proliferation studies

The proliferation assay of WT and CRISPR-Cas9 targeted THP-1 cells was conducted as follows: 5 x10^4 cells were seeded into clear 96 well plates, one plate for each time point. Proliferation was determined using WST1 reagent (*Sigma-Aldrich*) according to the manufacturer's instructions. Absorbance at 450 nm was measured 2 h after addition of WST1 and incubation at 37°C on a *Tecan* plate reader. Absorbance of WT cells was set to one at each time point.

Foam cell formation and oxLDL uptake

To induce foam cell formation, we seeded 5x10^5 THP-1 monocytes per well containing glass cover slips. Monocyte differentiation into macrophages was induced by adding 100 nM PMA to each well on days 0 and 1 and incubation for 48h hours. Thereafter the thus generated macrophages were washed with PBS, then incubated with 50µg/ml human ox-LDL for 24h in serum-free medium to induce foam cell formation. After ox-LDL incubation, Oil Red O staining (ORO) of the cells was performed as follows: wash with PBS, fix cells with 4% PFA/PBS for 15 min at RT, wash 3 times with PBS, rinse with 60% isopropanol for 15 s to facilitate the staining of neutral lipids, stain cells with filtered ORO working solution for 15 min at RT and in dark. ORO stock solution was prepared by dissolving 0.5 g ORO powder in 80 ml isopropanol (100%), mixed at 56 °C overnight, adjusted to 100 ml, mixed under gentle stirring, and filtered after pre-warming to 60°C. Working solution was prepared by diluting ORO stock solution with ddH2O 3:2 = factor 1.5. For staining, cells were rinsed with water and then hematoxylin used as a counterstain (cell nuclei) for 10 s. Destaining by washing with 60% isopropanol for 15 s, then PBS 3 times. Glass cover slips were taken out and mounted on slides for polarization microscopy and microphotography.

Monocyte-macrophage transition and macrophage polarization experiments

Monocyte-M0 macrophage differentiation and subsequent M1/M2 macrophage polarization studies employing FACS and TaqMan were conducted as follows: First, M0 macrophages were generated by incubation of THP-1 monocyte clones for seven days, with PMA at a concentration of 100 ng/ml. Thereafter, the cells were further incubated for another seven days, either with IFN-γ at 20 ng/ml plus LPS at 100 ng/ml to induce M1 polarization, or with IL-4 at 20 ng/ml plus IL-13 at 20 ng/ml to induce M2 polarization. The 'M0' TaqMan-based expression profiles and FACS data in Fig. 7 were obtained on day 7 of culture to characterize monocyte - M0 macrophage transition. The 'M1' and 'M2' expression profiles displayed there were obtained in day 14 of culture.

FACS analyses of the macrophage clones

After macrophage polarization, cells were washed once with ice-cold PBS and scrapped off using a mini scraper. Subsequently, macrophages were again washed with PBS + 5% FBS and incubated with 50 µl Fcγ-receptor block (*BD Biosciences*) in PBS for 10 min at RT to block unspecific binding. Cells were centrifuged at 300 x g for 5 min at 4°C. Then cells pellets were resuspended with 50 µl of FACS Buffer (PBS + 0,5%FBS + 0,05% NaN3) and stained with APC mouse anti-human CD14 (*BioLegend*), PE mouse anti-human CD11b (*BD Biosciences*) and Live/Dead Fixable Aqua Dead Cell Stain (*Invitrogen*) for 30 min at 4 °C in the dark. Cells were resuspended in 450 µl of FACS Buffer and analyzed with Attune™ NxT Flow Cytometer (*Thermo Fisher Scientific*). Statistical analysis was performed using GraphPad

Prism 9 software. Experimental data were analyzed by using one-way analysis of variance (ANOVA) with Dunnett´s post hoc test for multiple comparisons. The distribution of variables was assessed by Kolmogorov–Smirnov tests of normality.

Human adenovirus and coxsackievirus B3 studies

THP-1 cells were cultured in *RPMI 1640* medium (ATCC modification + 10 % fetal calf serum + 1 % P/S) at 37 °C, 5 % CO2. $5x10^5$ cells were seeded in 12-well culture plates and differentiation to macrophage-like cells was triggered by addition of 0.1 µM PMA o/n. Afterwards cells were transduced with coxsackievirus B3 at MOI 30. Detection of CVB3 genome, replicative intermediates, and plaque forming units (PFU) was conducted as described. In the adenovirus experiments, cells were instead transduced with a recombinant adenoviral virus expressing GFP (AdV5-GFP) or an "empty" adenovector expressing no transgene, at MOI 25, 12h post PMA addition. Virus structures were described previously.

RNA sequencing and data analysis

For the transcriptome mapping of control *vs.* CRISPR-Cas9 generated ΔmenRNA or ΔmascRNA cells, four biological cell culture replicates were grown for each of the three clones. From each of these cultures separate total RNA isolations were conducted by *TRIZOL*/Chloroform method. Thereafter the individual RNA preps were pooled for each of the clones, and the three resulting RNA pools (control, ΔmenRNA, ΔmascRNA) were subsequently used for RNA-seq analyses as follows. RNA integrity was visualized using *Agilent Bioanalyzer 2100*. For NGS-library preparation we used *Illumina TruSeq Stranded Total RNA Library Prep* Human/Mouse/Rat (S45-S56) or *NEBNext Ultr II Directional RNA Library Prep* Kit Illumina (E7760S) in combination with the *NEBNext rRNA Depletion Kit* (Human/Mouse/Rat) (E6310L) (S145-S154). For all samples paired end (2x75bp) sequencing was carried out on an *Illumina NextSeq* platform using *NextSeq 500/550 High Output Kit v2.5* (150 cycles). The resulting reads were mapped to the human genome (GRCh37 release 87/hg19) using *STAR v2.7.5b* with standard options. We then ran the *htseq-count module* from software package *HTSeq* with the stranded=reverse option reflecting the used library kit. As we used rRNA-depleted samples, all with rRNA-annotation were excluded from further analyses. To detect differentially expressed genes we used the R package DESeq v1.34.1. Next we normalized the raw gene specific read counts as Transcripts per Million base pairs (TPM) in order to perform a gene set enrichment analysis (GSEA) using the *R package ssGSEA2.0* by mapping them against a selection of gene set collections from *Molecular Signature Database (MsigDB)*. We thus generated Enrichment Scores (ES) for each sample and various gene sets. ES reflects how strongly the majority of genes from an individual gene set are expressed per regarded sample. This ES was then normalized to account for variations in gene set size. To correct for multiple testing the ssGSEA package uses Benjamini-Hochberg method, which calculates false discovery rates (FDR). For any combination of gene set and sample with a FDR value ≥ 0.05 we set the NES to zero. The gene set collections we used were: C2.CP: Canonical pathways; KEGG selection as a subset of CP; C2.CGP: chemical and genetic perturbations; C7: immunologic signature gene sets.

Quantitative RT-PCR

For quantitative gene expression analyses, isolated RNA was digested using *RNase free peqGOLD DNase I* (*Peqlab, Erlangen, Germany*) for 30 min to avoid genomic DNA contamination. To analyze the mRNA expression of target genes, 1 µg to 1,5 µg of total RNA was reverse transcribed using the *High Capacity RNA-to-cDNA* kit (*Life Technologies, Darmstadt, Germany*) according to the manufacturer's protocol and finally diluted to a concentration of 25 ng/µl. Assays for the following human target and reference genes were employed:

RT-PCR target	Gene name	TaqMan assay ID
AEN	apoptosis enhancing nuclease	Hs00224322_m1
AGO1	argonaute RISC component 1	Hs01084661_m1

AGO2	argonaute RISC component 2	Hs01085579_m1
ANG	angiogenin	Hs04195574_sH
ANGPT1	angiopoietin 1	Hs00919201_m1
ANGPT4	angiopoietin 4	Hs00907074_m1
ANGPTL4	angiopoietin like 6	Hs01101122_m1
BOK	BCL2 family apoptosis regulator BOK	Hs00261296_m1
CIITA	class II major histocompatibility complex transactivator	Hs00931699_m1
CCR5	C-C motif chemokine receptor 5	Hs00152917_m1
CCR7	C-C motif chemokine receptor 7	Hs01013469_m1
CD11b (ITGAM)	integrin subunit α_M	
CD11c (ITGAX)	integrin subunit α_X	
CD36 (SCARB3)	scavenger receptor	
CD64 (FCGR1A)	IgG Fc receptor 1	
CD80	CTLA-4 counter-receptor	
CD93 (C1QR1)		
CSF1 (MCSF)	macrophage colony stimulating factor	
CSF1R (CD115)	CSF1 receptor	
CTIF	cap binding complex dependent translation initiation factor	Hs00969548_m1
EEF1A2	eukaryotic translation elongation factor 1 $\alpha 2$	Hs00951287_m1
EIF3CL	eukaryotic translation initiation factor 3 subunit C like	Hs01105769_g1
ESAM	endothelial cell adhesion molecule	Hs01113367_g1
FAS	Fas cell surface death receptor	Hs00236330_m1
FASLG	Fas ligand	Hs00181226_g1
GADD45G	growth arrest and DNA damage inducible γ	Hs02566147_s1
HDAC4	histone deacetylase 4	Hs01041648_m1
HDAC 9	histone deacetylase 9	Hs01081548_g1
ICAM1	intercellular adhesion molecule 1	Hs00164932_m1
ICAM2	intercellular adhesion molecule 2	Hs00609563_m1
ICAM3	intercellular adhesion molecule 3	Hs00913466_g1
ICAM4	intercellular adhesion molecule 3	Hs05017468_s1
IFIT1	interferon induced protein with tetratricopeptide repeats 1	Hs01911452_s1
IFIT2	interferon induced protein with tetratricopeptide repeats 2	Hs01584837_s1
IFIT3	interferon induced protein with tetratricopeptide repeats 3	Hs01922752_s1
IFITM1	interferon induced transmembrane protein 1	Hs00705137_s1
IFITM2	interferon induced transmembrane protein 2	Hs00829485_sH
IFITM3	interferon induced transmembrane protein 3	Hs04194512_g1
IFITM5	interferon induced transmembrane protein 5	Hs00416846_m1
IFNAR1	interferon alpha and beta receptor subunit 1	Hs01066116_m1
IFNAR2	interferon α and β receptor subunit 2	Hs01022059_m1
IFNB1	interferon beta 1	Hs00277188_s1
IFNG	interferon gamma	Hs00989291_m1
IL1A	interleukin 1A	Hs00174092_m1
IL1B	interleukin 1B	Hs01555410_m1

IL10	interleukin 10	Hs00961619_m1
IL18R1	interleukin 18 receptor 1	Hs00977691_m1
IL18RAP	interleukin 18 receptor accessory protein	Hs00187256_m1
IL2	interleukin 2	Hs00174114_m1
IL2RB	interleukin 2 receptor β	Hs01081697_m1
IL2RG	interleukin 2 receptor γ	Hs00415671_m1
IL4R	interleukin 4 receptor	Hs00965057_m1
IL6 (IFNB2)	interleukin 6	Hs00174131_m1
IL6R	interleukin 6 receptor	Hs01075664_m1
IL8	interleukin 8	Hs00174103_m1
IRF3	interferon regulatory factor 3	Hs01547282_m1
IRF7	interferon regulatory factor 7	Hs01014809_g1
IRF9	interferon regulatory factor 3	Hs00196051_m1
ISG15	ISG15 ubiquitin like modifier	Hs01921425_s1
MAVS	mitochondrial antiviral signaling protein	Hs00920075_m1
MALAT1	metastasis associated lung adenocarcinoma transcript 1	Hs00273907_s1
MCAM (CD146)	melanoma cell adhesion molecule	
MEFV	MEFV innate immuity regulator pyrin	Hs00925524_m1
MRC1 (CD206)	macrophage mannose receptor 1	
MSR1 (SR-A)	macrophage scavenger receptor 1	
NAIP	NLR family apoptosis inhibitory protein	Hs03037952_m1
NCAM1	neural cell adhesion molecule 1	Hs00941830_m1
NCAM2	neural cell adhesion molecule 2	Hs01562296_m1
NEAT1	nuclear paraspeckle assembly transcript 1	Hs01008264_s1
NFATC2	nuclear factor of activated T cells 2	Hs00905451_m1
NOD2	nucleotide binding oligomerization domain containing 2	Hs01550753_m1
NOS2	nitric oxide synthase 2	Hs01075529_m1
NOS3	nitric oxide synthase 3	Hs01574665_m1
NLRC3	NLR family CARD domain containing 3	Hs01054713_m1
NLRC4	NLR family CARD domain containing 4	Hs00368367_m1
NLRC5	NLR family CARD domain containing 5	Hs01072143_m1
NT5C1A	5'-nucleotidase cytosolic IA	Hs00261369_m1
OLR1 (LOX1)	oxidized low density lipoprotein receptor 1	
RIPK3	receptor interacting serine/threonine kinase 3	Hs00179132_m1
RLR1 (DDX58)	RIG-like receptor 1	Hs00204833_m1
RLR2 (IFIH1)	RIG-like receptor 2	Hs01070332_m1
RLR3 (DHX58)	RIG-like receptor 3	Hs00225561_m1
ROCKI	MARCKS cis-regulating lncRNA promoter of cytokines and inflammation	
SERPING1		
SLFN12L	schlafen family member 12 like	Hs04334088_m1
SLFN5	schlafen family member 5	Hs00288058_m1
SOX4	SRY-box transcription factor 4	Hs05029754_s1
TGFA	transforming growth factor α	Hs00608187_m1

TGFB2	transforming growth factor β2	Hs00234244_m1
TGFBI	transforming growth factor β induced	Hs00932747_m1
TLR1 (LPRS)		
TLR2	Toll-like receptor 2	Hs02621280_s1
TLR8	Toll-like receptor 8	Hs00152972_m1
TLR9	Toll-like receptor 9	Hs00152973_m1
TLR10 (CD290)	Toll-like receptor 10	
TNF (TNFSF2)	tumour necrosis factor α	Hs00174128_m1
TWIST2	twist family bHLH transcription factor 2	Hs02379973_s1
VCAM	vascular cell adhesion molecule 1	Hs01003372_m1
VEGFA	vascular endothelial growth factor A	Hs00900055_m1
18S	18S-RNA	Hs9999901_s1
GAPDH	glyceraldehyde-3-phosphate dehydrogenase	Hs99999905_m1
HPRT1	hypoxanthine phosphoribosyltransferase 1	Hs01003267_m1

Expression levels measured by qPCR were quantified as delta-delta-C_t values, determined by the C_t value of a candidate RNA minus the C_t of the reference gene.

<u>Statistical analyses</u>

Cell culture experiments: Statistical data analyses were done using *IBM SPSS Statistics 24* or *GraphPad* software. Descriptive statistics include absolute and relative frequencies for categorial variables and mean and standard deviation, median, and range for quantitative measurements. For inter-group comparisons Student's t-test or the χ^2 test was used for quantitative or categorical variables, respectively. P-values ≤ 0.05 are considered significant, and no Bonferroni adjustment has been performed.

Human molecular genetics: Genome-wide association analyses of healthy samples versus samples with inflammatory, metabolic, and/or cardiovascular conditions were performed via logistic regression analysis implemented in *snptest v2.5.2*. For sensitivity analysis samples were stratified by sex. Summary-level results were meta-analyzed with *METAL* (*Willer CJ, Li Y, Abecasis GR. METAL: fast and efficient meta-analysis of genomewide association scans. Bioinformatics. 2010;26:2190–1*) using the classical approach which utilizes the effect size estimates and standard errors. Only variants with an imputation quality > 30% and Hardy-Weinberg equilibrium p-value > 0.0001 were included in the meta-analysis. Variants with p-value < 5×10^{-8} (the standard threshold) were considered to be genome-wide significant.

<u>References regarding the methods</u>

1. M. C. Gundry *et al.*, Highly Efficient Genome Editing of Murine and Human Hematopoietic Progenitor Cells by CRISPR/Cas9. *Cell Rep* 17, 1453-1461 (2016).
2. L. Brunetti, M. C. Gundry, A. Kitano, D. Nakada, M. A. Goodell, Highly Efficient Gene Disruption of Murine and Human Hematopoietic Progenitor Cells by CRISPR/Cas9. *J Vis Exp* (2018).
3. M. Kespohl *et al.*, Protein modification with ISG15 blocks coxsackievirus pathology by antiviral and metabolic reprogramming. *Sci Adv* 6, eaay1109 (2020).
4. S. Pinkert *et al.*, Prevention of cardiac dysfunction in acute coxsackievirus B3 cardiomyopathy by inducible expression of a soluble coxsackievirus-adenovirus receptor. *Circulation* 120, 2358-2366 (2009).
5. D. Werk *et al.*, Combination of soluble coxsackievirus-adenovirus receptor and anti-coxsackievirus siRNAs exerts synergistic antiviral activity against coxsackievirus B3. *Antiviral Res* 83, 298-306 (2009).
6. H. Fechner *et al.*, Coxsackievirus B3 and adenovirus infections of cardiac cells are efficiently inhibited by vector-mediated RNA interference targeting their common receptor. *Gene Ther* 14, 960-971 (2007).
7. L. Suckau *et al.*, Long-term cardiac-targeted RNA interference for the treatment of heart failure restores cardiac function and reduces pathological hypertrophy. *Circulation* 119, 1241-1252 (2009).

8. A. Dobin *et al.*, STAR: ultrafast universal RNA-seq aligner. *Bioinformatics* 29, 15-21 (2013).
9. S. Anders, P. T. Pyl, W. Huber, HTSeq--a Python framework to work with high-throughput sequencing data. *Bioinformatics* 31, 166-169 (2015).
10. S. Anders, W. Huber, Differential expression analysis for sequence count data. *Genome Biol* 11, R106 (2010).
11. D. A. Barbie *et al.*, Systematic RNA interference reveals that oncogenic KRAS-driven cancers require TBK1. *Nature* 462, 108-112 (2009).
12. A. Subramanian *et al.*, Gene set enrichment analysis: a knowledge-based approach for interpreting genome-wide expression profiles. *Proc Natl Acad Sci U S A* 102, 15545-15550 (2005).
13. A. Liberzon *et al.*, Molecular signatures database (MSigDB) 3.0. *Bioinformatics* 27, 1739-1740 (2011).

Supplemental Figure S1A

CRISPR-Cas9 target region to generate ∆menRNA cells

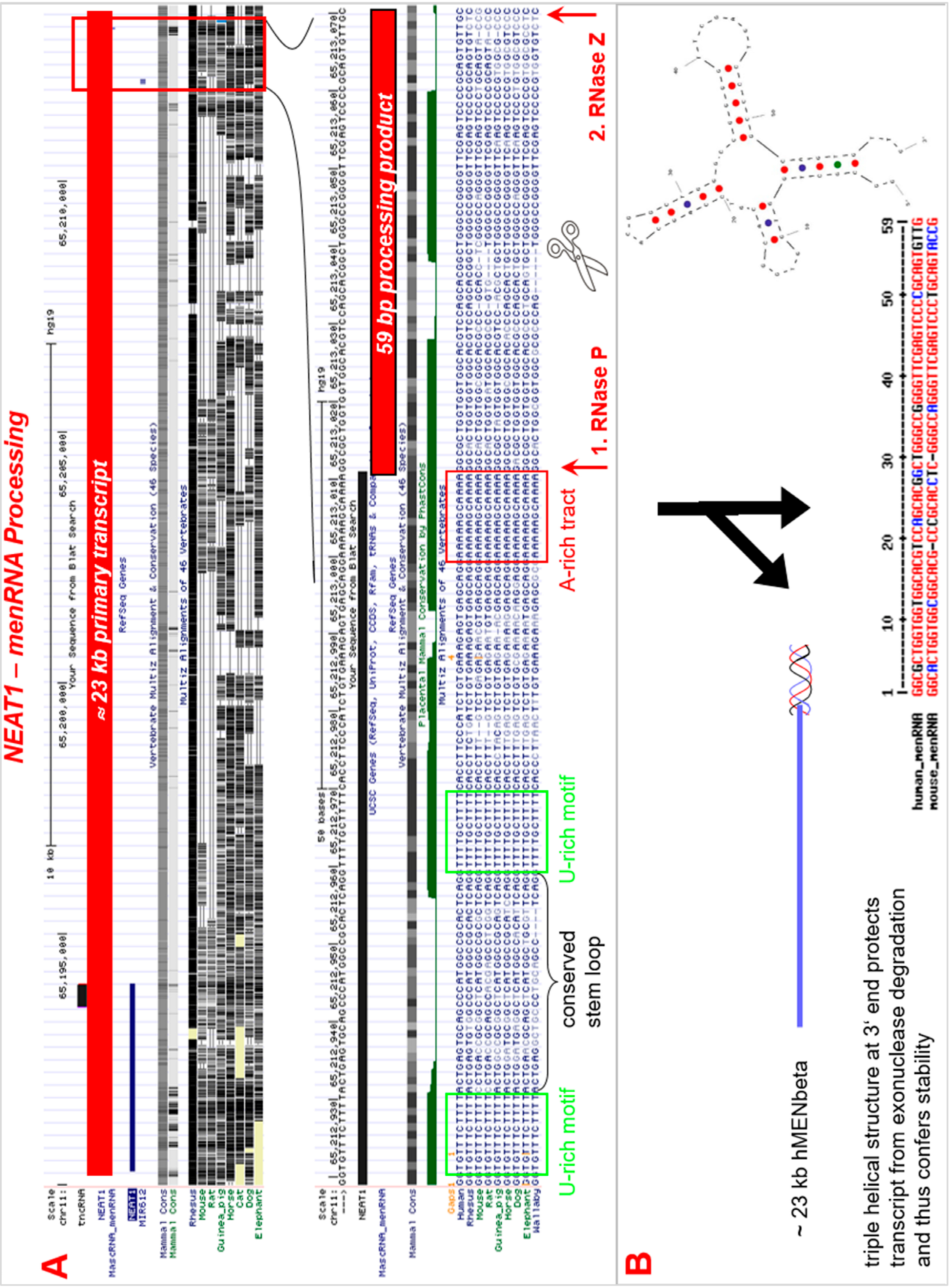

Supplemental Figure S1B

CRISPR-Cas9 target region to generate ∆mascRNA cells

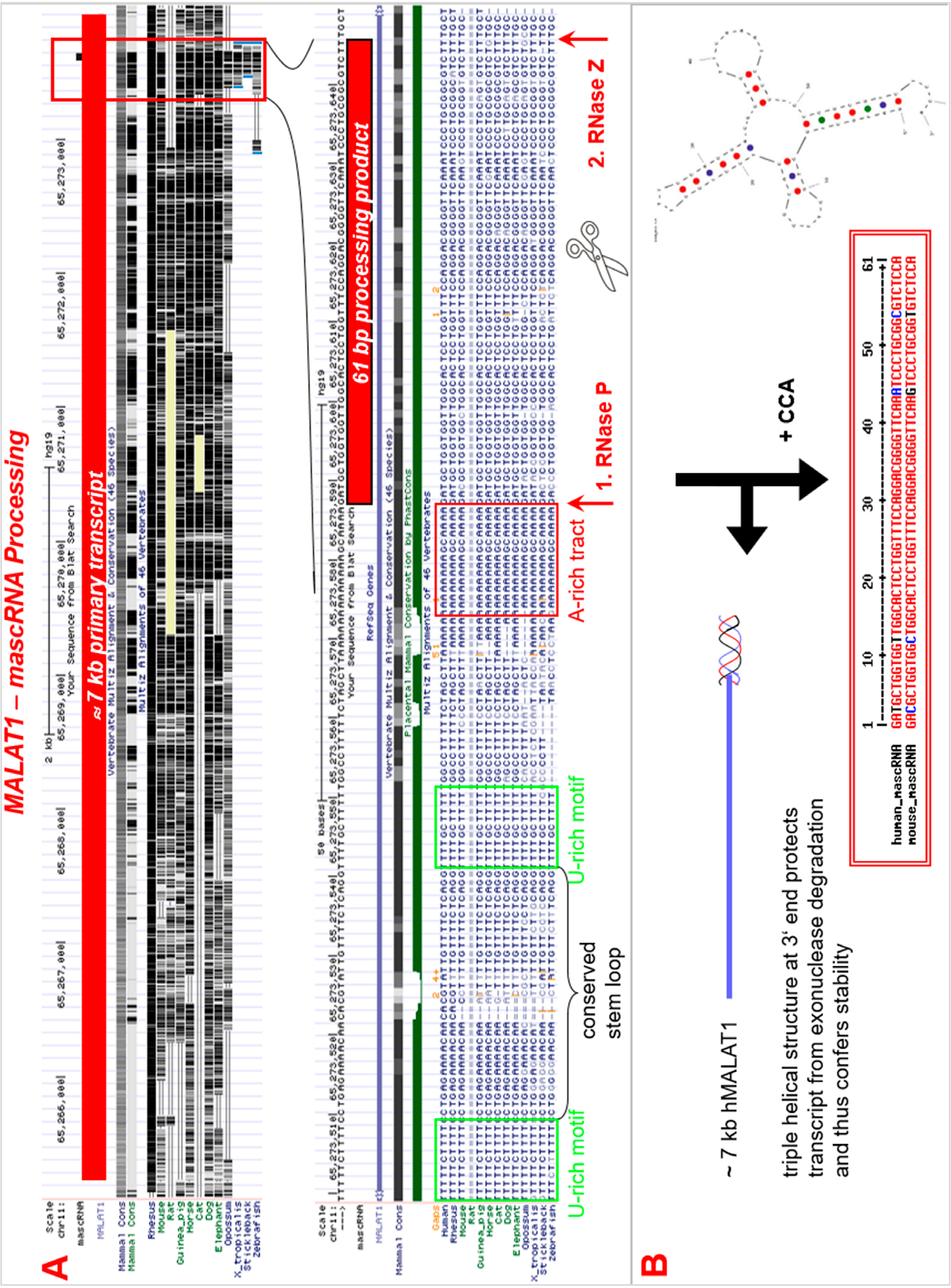

Supplemental Figure S1C

Recombinant expression and Northern blot analysis of menRNA

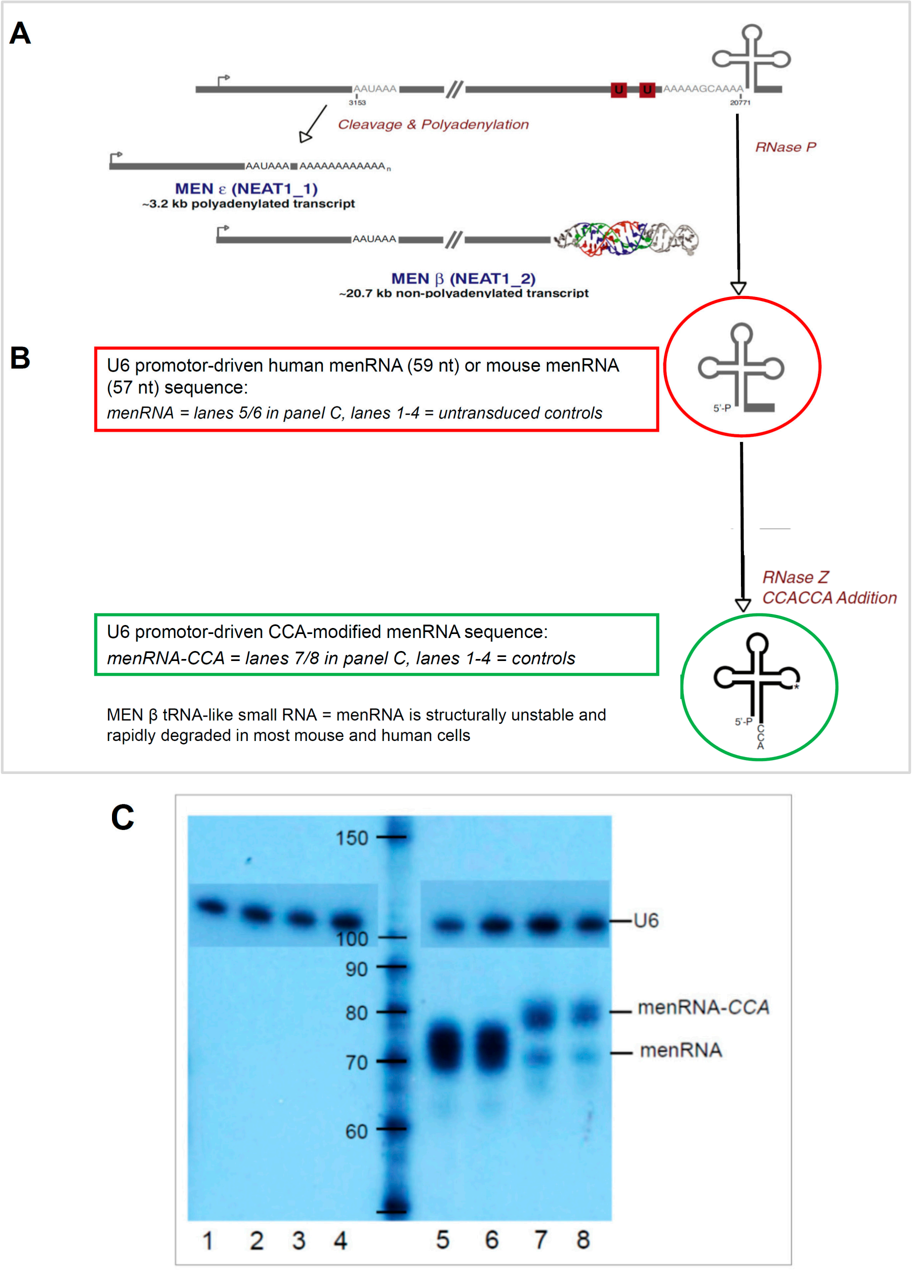

Panel A: Locations of two long primary transcripts from the *NEAT1* locus with size 3.7 kb and poly A tail (MEN ε), or size ≈23 kb and triple helical tail (MEN β). A tRNA-like 'menRNA' is enzymatically generated at the far 3' end of *NEAT1*. *Panel B*: Outline of the enzymatic biosynthesis of menRNA. This product is posttranscriptionally modified by CCA or CCACCA addition with CCACCA tagging it for rapid degradation (Wilusz *et al.* tRNAs marked with CCACCA are targeted for degradation. *Science* 2011;334:817-821 | Kuhn *et al.* On-enzyme refolding permits small RNA and tRNA surveillance by the CCA-adding enzyme. *Cell* 2015;160:644-658). *Panel C*: Northern blot analysis of HEK 293 cells transfected with recombinant adenovectors expressing menRNA (lanes 5/5) or menRNA±CCA (lanes 7/8). Lanes 1-4 show untransduced HEK 293 control cells. In these and several others including THP-1 cells there was no detectable menRNA. U6 RNA was employed as loading control.

Supplemental Figure S2

Structure prediction for residual sequences in ΔmenRNA and ΔmascRNA cells

Panel A

residual menRNA (human)

5'-ggcgctggtg gtggc*acgtc cagcacggct gggccggggt tcgagtcccc gcagtgttg-3'*

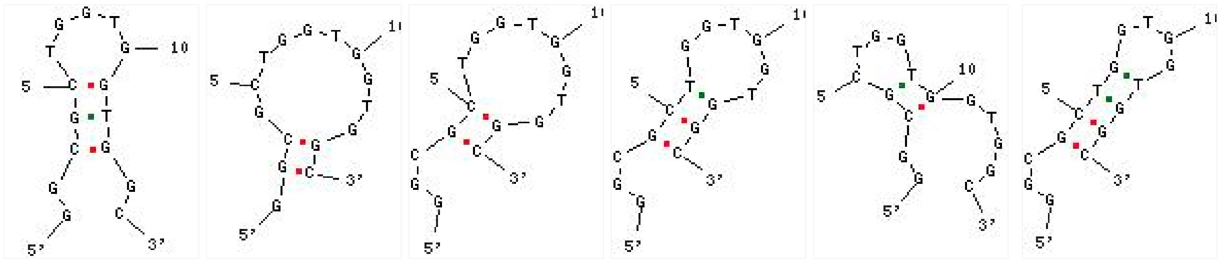

Panel B

residual mascRNA (human)

5'-GATGCTGGTGGTTGGCACTCCTGGTTTCCAGGA*CGGGGTTCAAATCCCTG*CGGCGTCT-3'

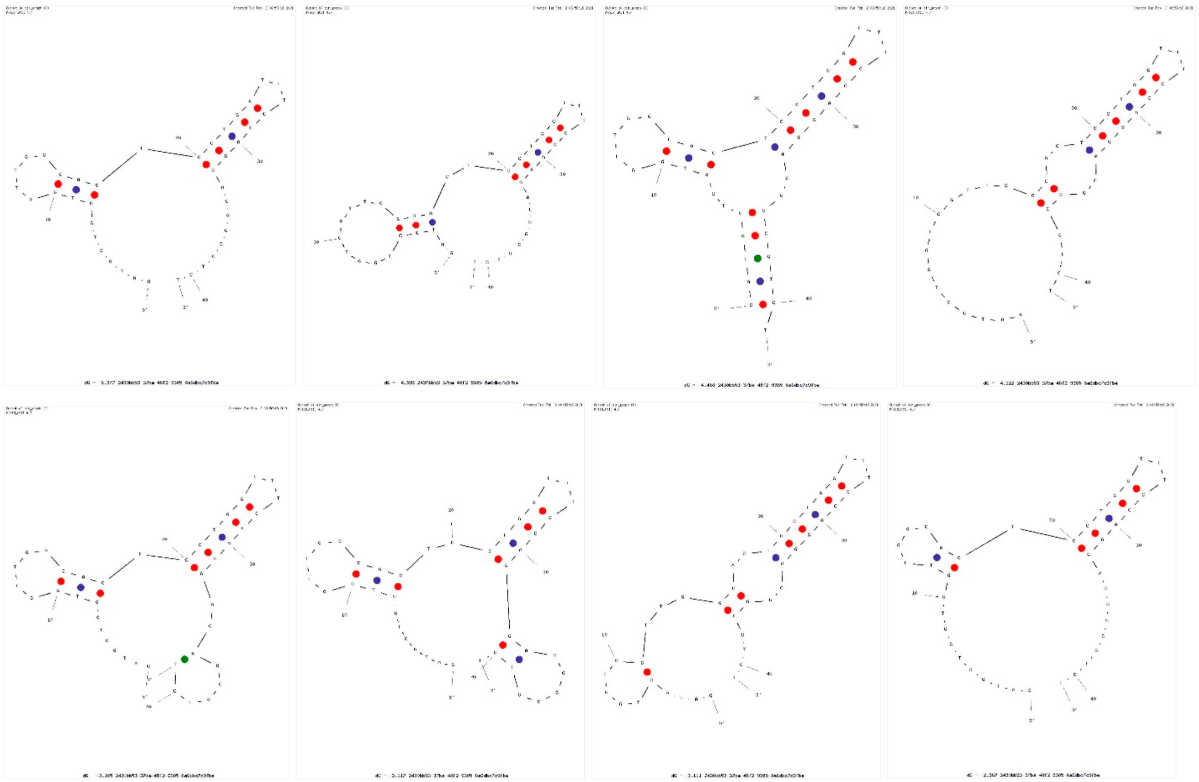

Possible structures acquired by sequence residues in ΔmenRNA and ΔmascRNA clones as predicted by OligoAnalyzer (IDT) https://eu.idtdna.com/calc/analyzer. None of these structures has resemblance with classical or atypical tRNAs.

Supplemental Figure S3A

In vitro transcription of single-guide RNAs

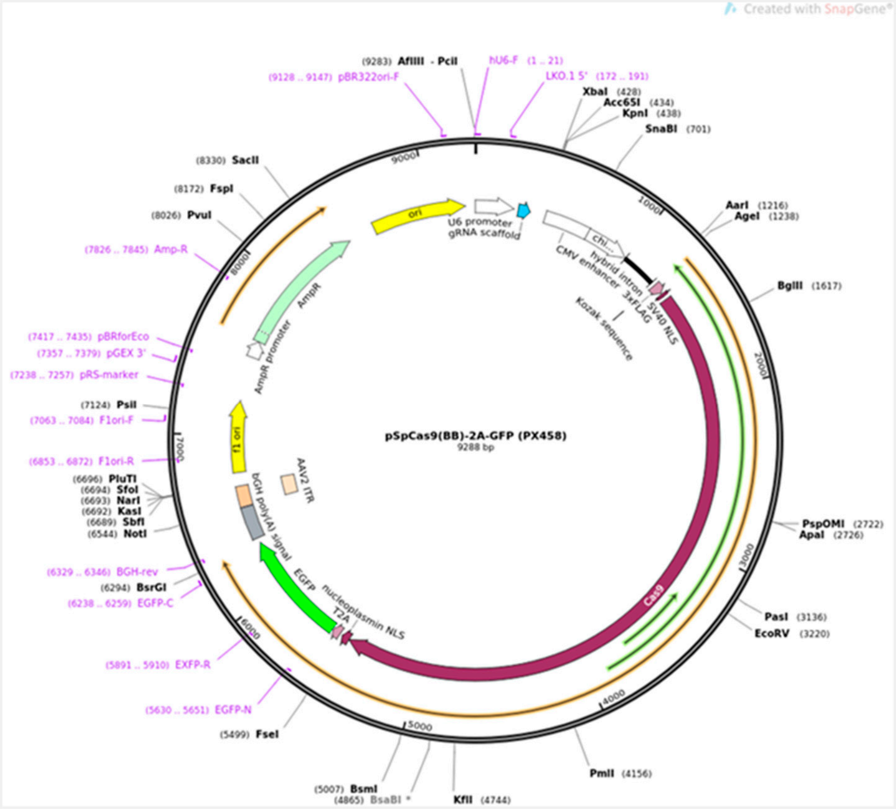

Supplemental Figure S3B

PCR analysis of ΔmenRNA and ΔmascRNA cells

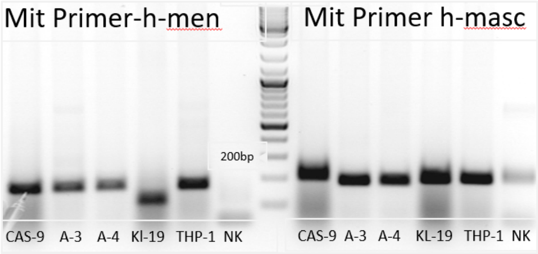

Supplemental Figure S4

Known sequence polymorphisms in the human menRNA and mascRNA sequences

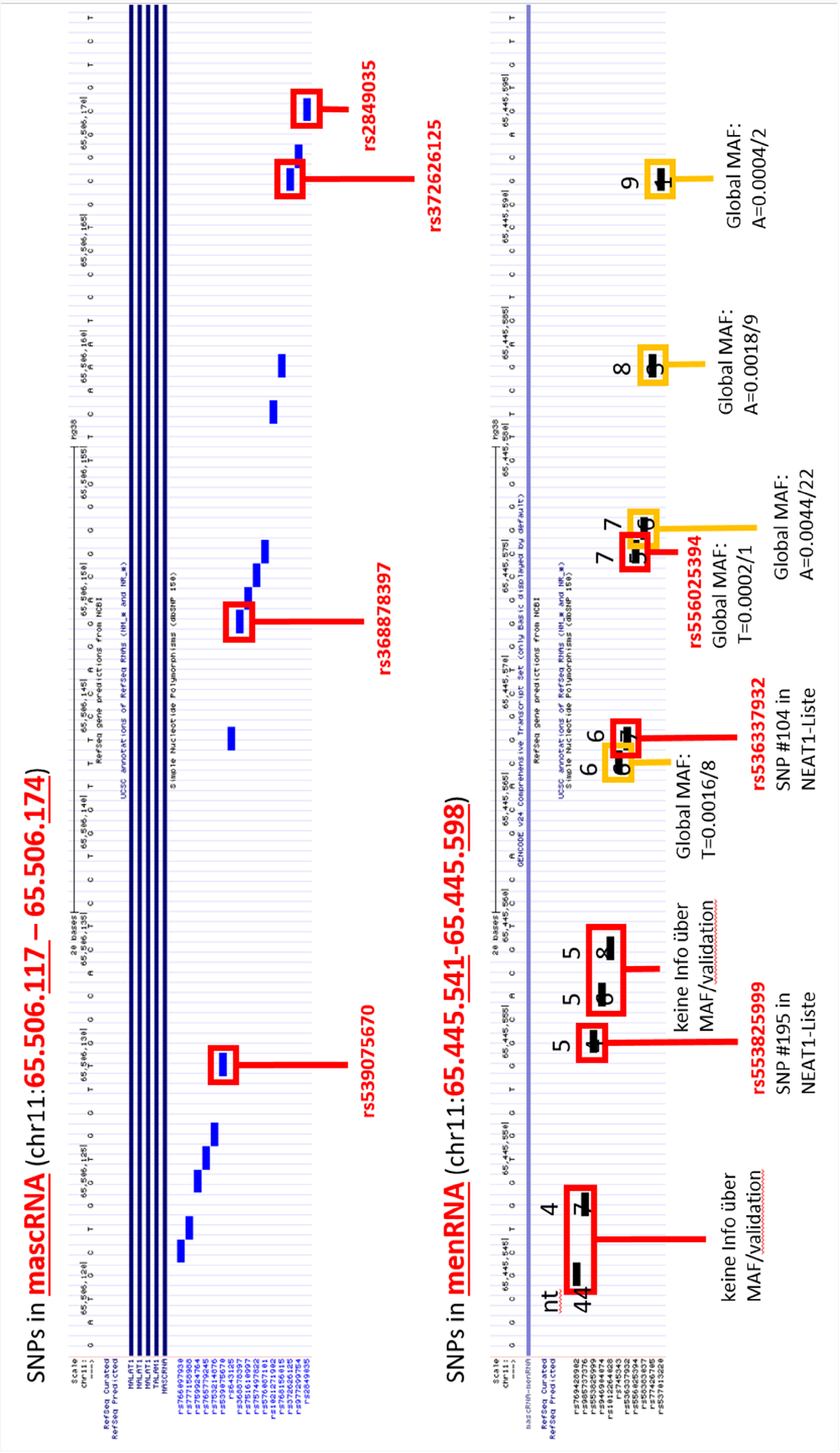

Supplemental Figure S5

Outline regarding SNPs analyses in SHIP cohorts

SHIP genotypes

GRCh38 SNPs in **menRNA** (chr11:**65.445.541 - 65.445.598**)
GRCh37 Δ -222.234 ⇒ 65.223.307 - 65.223.364

GRCh38 SNPs in **mascRNA** (chr11:**65.506.117 – 65.506.174**)
GRCh37 Δ -222.234 ⇒ 65.283.883 - 65.283.940

Gene	Build Chromosom:Position	cohort	variants* for 1KGp3v5
NEAT1	NR_002819.4<br>GRCh37 11:<br>**65.190.269 - 65.194.003**	SHIP-0	19
		SHIP-Trend	14
		SHIP-Trend (batch2)	13
MALAT1	NR_131012.1<br>GRCh37 11:<br>**65.265.224 - 65.273.940**	SHIP-0	43
		SHIP-Trend	24
		SHIP-Trend (batch2)	32

imputation panel for analyses: 1000G phase 3 version 5 (1KGp3v5)

most of the variants in *NEAT1* and *MALAT1* are rare, i.e. minor allele frequency (MAF) < 0.5 %

range of variants: *start of NEAT1 - end of MALAT1* ± 10K bp (i.e. 11: 65.180.269 - 65.283.940)

meta analysis of all SHIP cohorts via logistic regression

adjusted for smoking status, (BMI)**, (age)**, (sex)**

* polymorphic variants only, ** if applicable

Genetic heterogeneity of the *NEAT1-MALAT1* region in humans

SNPs available from the SHIP study for the *NEAT1-MALAT1* region, with their respective positions, are shown in Suppl. Fig. S6AB. No full sequence data for the specific target regions of the current study, *i.e.* mascRNA and menRNA, are currently available for the SHIP general population or for any patient cohorts, although several SNP are known to occur mascRNA and menRNA (Suppl. Fig. 4). Consistent with prior findings in cell culture and animal models (Gast *et al*. Immune system-mediated atherosclerosis caused by deficiency of long non-coding RNA *MALAT1* in ApoE$^{-/-}$ mice. Cardiovasc Res 2019;115: 302-314 | Cremer *et al.* Hematopoietic deficiency of the long noncoding RNA *MALAT1* promotes atherosclerosis and plaque inflammation. Circulation 2019;139:1320-1334), one *MALAT1* SNP with very low minor allele frequency (MAF=0.01) was associated (p=0.0062) with systemic low level inflammation (CRP>3.0 mg/L) (Suppl. Fig. S7). This SNP is located upstream of *MALAT1* in the promotor region regulating *MALAT1* expression (Suppl. Fig. S6A). Further, there was an association (p<0.01) of eight SNPs (low MAF=0.09 for all) within the *NEAT1* region with the phenotype of BMI >35 kg/m^2 and LDL >164 mg/dl (Suppl. Fig. S6B).

Supplemental Figure S6A

Positions of *MALAT1* region SNPs analyzed in SHIP cohorts

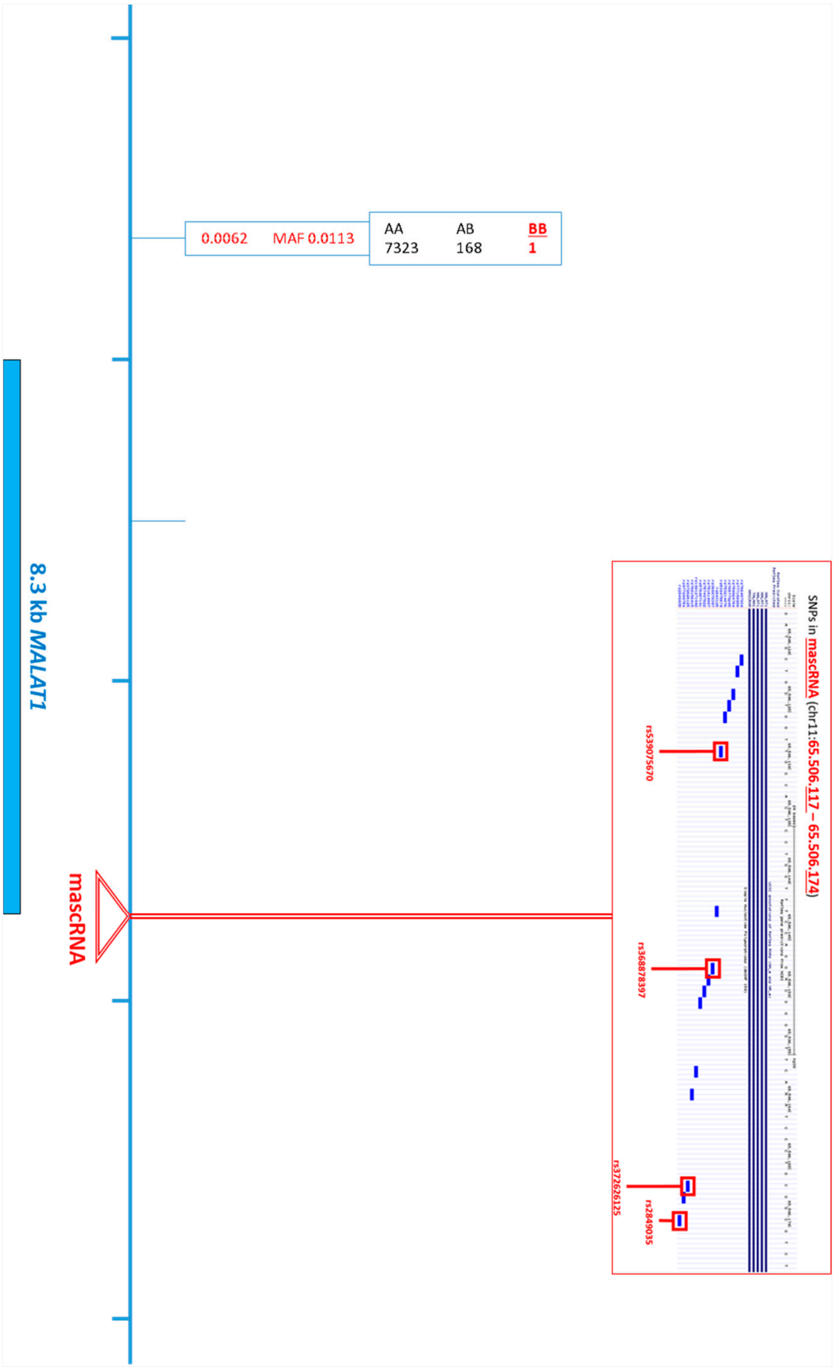

Supplemental Figure S6B

Positions of *NEAT1* region SNPs analyzed in SHIP cohorts

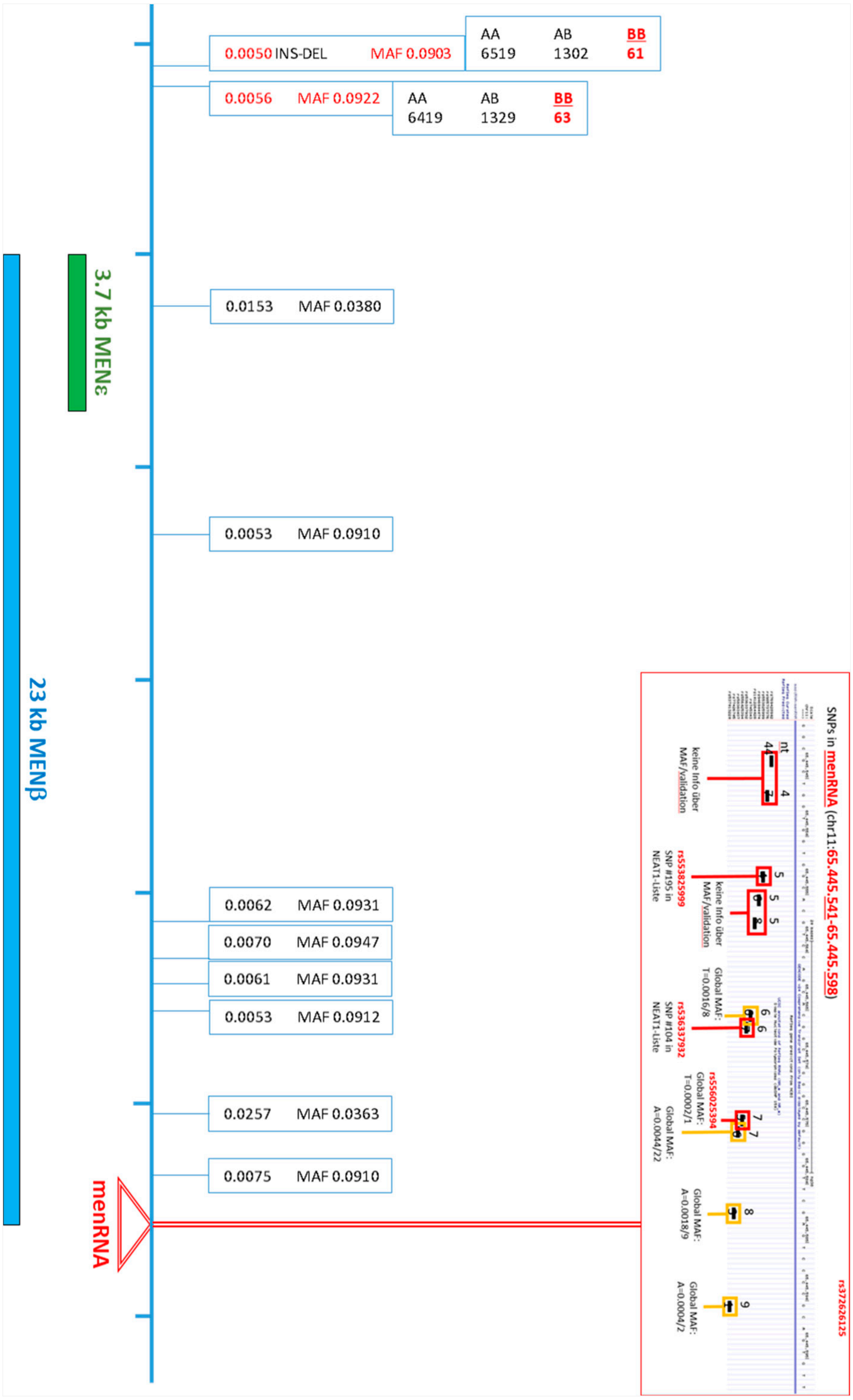

Supplemental Figure S7

Human population genetic data

SNP associated with systemic low level inflammation **CRP >3.0 mg/L**

rsID	CHR	POS_GRCh38	REF	ALT	MAF	AA/AB/BB	N	beta	se	p-value	gene
rs113637620	11	65.495.816	T	C	0,0113	7323/168/1	7494	0,5087	0,1859	0,0062	*MALAT1*

SNPs associated with class II obesity **BMI >35 kg/m2** and **LDL >164 mg/dl**

rsID	CHR	POS_GRCh38	REF	ALT	MAF	AA/AB/BB	N	beta	se	p-value	gene
11_65185874_IN_DEL	11	65.418.403	GA	G	0,0903	6519/1302/61	7883	-0,179	0,0638	0,0050	*NEAT1*
rs508286	11	65.440.647	A	G	0,0941	6463/1355/65	7883	-0,172	0,0615	0,0052	*NEAT1*
rs580933	11	65.429.413	C	G	0,0912	62/1315/6506	7883	-0,1743	0,0625	0,0053	*NEAT1*
rs550015	11	65.418.877	A	G	0,0922	63/1329/6491	7883	-0,1757	0,0634	0,0056	*NEAT1*
rs481335	11	65.439.998	T	C	0,0931	6479/1340/64	7883	-0,169	0,0617	0,0061	*NEAT1*
rs673753	11	65.438.546	C	T	0,0931	6479/1340/64	7883	-0,1686	0,0616	0,0062	*NEAT1*
rs475967	11	65.439.446	A	G	0,0947	6454/1362/66	7883	-0,1661	0,0616	0,0070	*NEAT1*
rs550894	11	65.444.469	C	A	0,0910	6510/1313/61	7883	-0,1681	0,0629	0,0075	*NEAT1*

Supplemental Figure S8A

Transduction of recombinant GFP-expressing adenovirus into CRISPR-Cas9-generated defective human macrophages

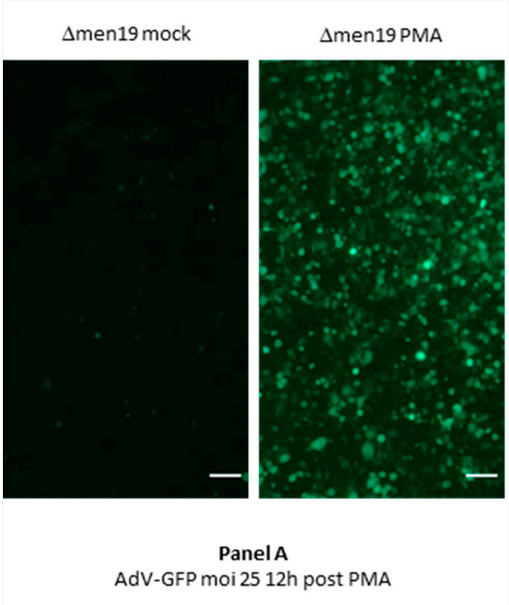

Supplemental Figure S8B

Transduction of human coxsackievirus B3 into CRISPR-Cas9-generated defective human macrophages

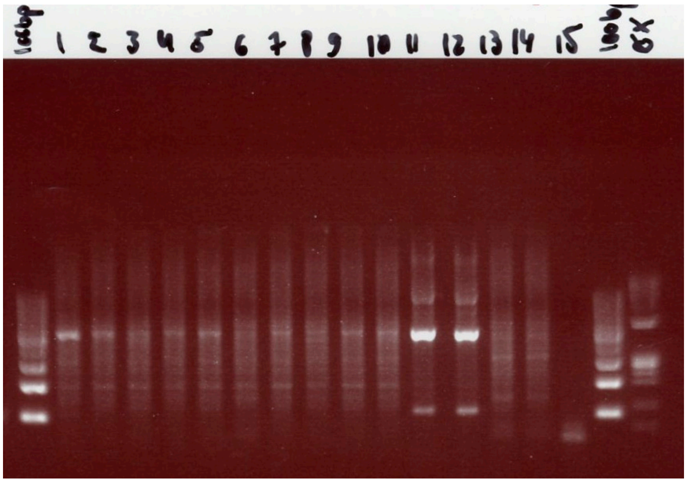

Marker: Øx bzw 100bp Peqlab
lane 1: Δmen19 cells + 30moi CVB3 (18.11.2020)
lane 2: Δmasc4 cells + 30moi CVB3 (18.11.2020)
lane 3: Δmasc3 cells + 30moi CVB3 (18.11.2020)
lane 4: cas 9 cells + 30moi CVB3 (18.11.2020)
lane 5: THP-1 cells + 30moi CVB3 (18.11.2020)
lane 6: Δmen19 cells + 30moi CVB3 (24.11.2020)
lane 7: Δmasc4 cells + 30moi CVB3 (24.11.2020)
lane 8: Δmasc3 cells + 30moi CVB3 (24.11.2020)
lane 9: cas 9 cells + 30moi CVB3 (24.11.2020)
lane10: THP-1 cells + 30moi CVB3 (24.11.2020)
lanes 11+12: Vero cells + 30moi CVB3 (positive control)
lanes 13+14: Vero cells w/o CVB3 (negative control)
lane 15: water control PCR reagents
Expected fragment size for CVB3 replicative intermediates: 478bp

Supplemental Figure S9A

Anomalous expression of innate immune sensor molecules and interferon-induced genes in ΔmenRNA and ΔmascRNA macrophages

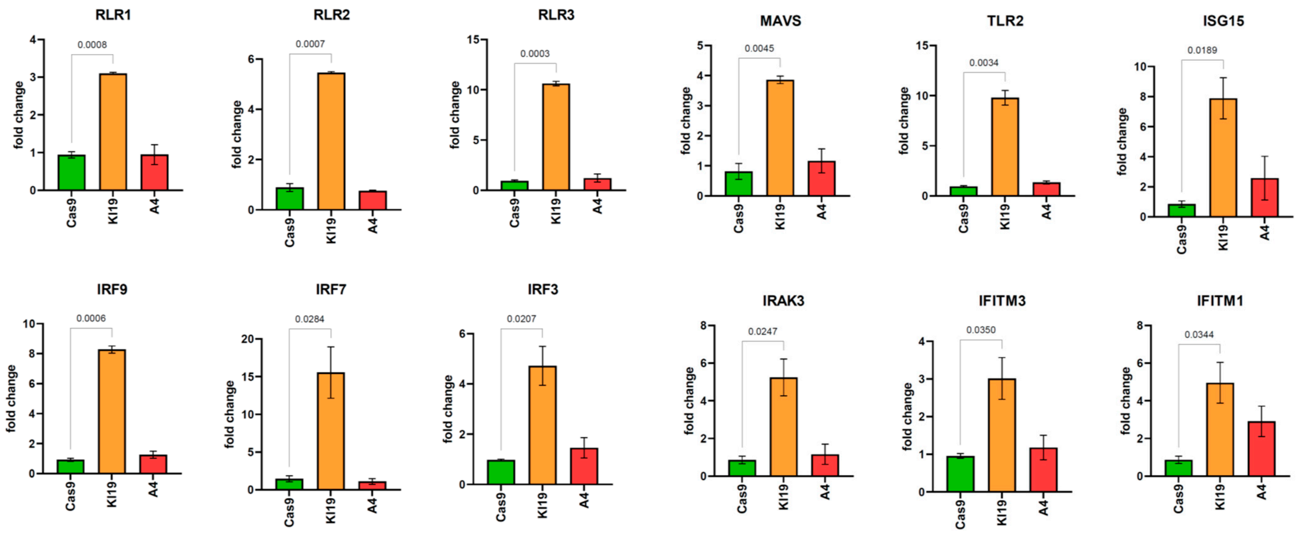

Upon PMA-induced differentiation of ΔmenRNA and ΔmascRNA monocytes into macrophages (Suppl. Fig. S8A), these displayed further disturbances of innate immune regulation under baseline conditions including constitutive induction of the cytosolic innate immune sensor molecules RLR1 (DExD/H-box helicase DDX58), RLR2 (IFIH1; MDA-5), RLR3 (RNA helicase DHX58; LGP2), and MAVS (mitochondrial antiviral signaling protein). In ΔmenRNA macrophages several interferon (IFN) system genes were also constitutively upregulated up to ~15-fold: the IFN regulatory factors IRF3, IRF7, and IRF9; IFN-stimulated gene ISG15; IFN-induced transmembrane proteins IFITM1 and IFITM3 In ΔmenRNA macrophages several interferon (IFN) system genes were also constitutively upregulated up to ~15-fold: the IFN regulatory factors IRF3, IRF7, and IRF9; IFN-stimulated gene ISG15; IFN-induced transmembrane proteins IFITM1, IFITM3; and GVINP1. Bar graphs show means ± SE from three biological replicates.

Supplemental Figure S9B

FACS analysis of ΔmenRNA and ΔmascRNA monocyte to M0 macrophage transition

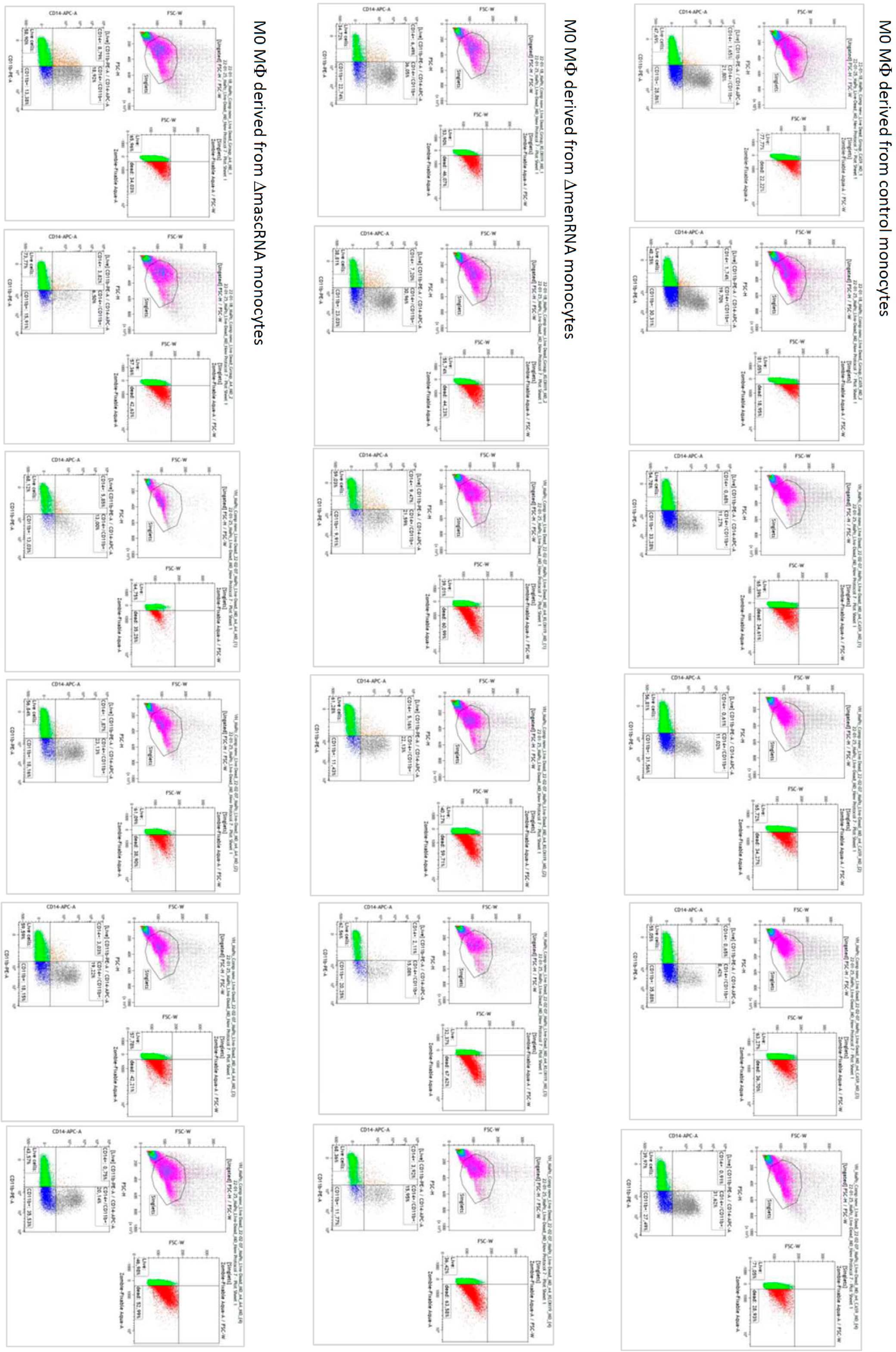

Supplemental Table S1

RNA-seq FPKM data for ∆menRNA monocytes

S1A - Innate immune sensors

BASELINE		control	∆men19	ratio
CIITA	class II major histocompatibility complex transactivator	6,669	115,166	17,270
NOD2	nucleotide binding oligomerization domain containing 2	21,117	157,454	7,456
NLRC5	NLR family CARD domain containing 5	230,068	870,944	3,786
TLR2	toll like receptor 2	379,000	1299,218	3,428
PYCARD	PYD and CARD domain containing	368,997	147,557	0,400
CARD10	caspase recruitment domain family member 10	33,343	8,997	0,270
NAIP	NLR family apoptosis inhibitory protein	54,460	8,997	0,165
NLRC4	NLR family CARD domain containing 4	33,343	5,398	0,162
MEFV	MEFV innate immuity regulator, pyrin	240,071	32,390	0,135
NLRC3	NLR family CARD domain containing 3	185,610	14,396	0,078

LPS100		control	∆men19	ratio
BOK	BCL2 family apoptosis regulator BOK	2,404	17,473	7,269
NOD2	nucleotide binding oligomerization domain containing 2	68,505	321,171	4,688
FAM129C	niban apoptosis regulator 3	2,404	9,985	4,154
NLRC5	NLR family CARD domain containing 5	292,050	1161,542	3,977
PYCARD	PYD and CARD domain containing	760,771	371,926	0,489
RIPK3	receptor interacting serine/threonine kinase 3	137,011	34,114	0,249
NAIP	NLR family apoptosis inhibitory protein	69,707	16,641	0,239
CARD14	caspase recruitment domain family member 14	4,807	0,832	0,173
TLR1	toll like receptor 1	104,561	15,809	0,151
NLRC4	NLR family CARD domain containing 4	63,698	6,656	0,104
MEFV	MEFV innate immuity regulator, pyrin	301,664	26,626	0,088
MPEG1	macrophage expressed 1	212,728	16,641	0,078
TLR8	toll like receptor 8	32,450	2,496	0,077
TLR10	toll like receptor 10	10,817	0,832	0,077
NLRC3	NLR family CARD domain containing 3	137,011	4,160	0,030
TLR7	toll like receptor 7	4,807	0,000	

S1B – Transcription and nuclear factors, epigenetic modifiers

BASELINE		control	men19	ratio
GDAP1L1	ganglioside induced differentiation associated protein 1 like 1	0,000	17,995	
HDAC11	histone deacetylase 11	0,000	3,599	
GCM1	glial cells missing transcription factor 1	0,000	8,098	
SIM1	SIM bHLH transcription factor 1	0,000	8,098	
CCPG1	cell cycle progression 1	52,238	556,936	10,662
NPDC1	neural proliferation, differentiation and control 1	4,446	44,987	10,119
MTURN	maturin, neural progenitor differentiation regulator homolog	24,452	233,931	9,567
RGCC	regulator of cell cycle	6,669	51,285	7,690
GATA2	GATA binding protein 2	21,117	91,773	4,346

NFATC2	nuclear factor of activated T cells 2	563,499	120,565	0,214
METTL7B	methyltransferase like 7B	166,716	33,290	0,200
TBX15	T-box transcription factor 15	73,355	13,496	0,184
GATA6	GATA binding protein 6	10,003	1,799	0,180
HIC1	HIC ZBTB transcriptional repressor 1	5,557	0,900	0,162
SOX4	SRY-box transcription factor 4	868,033	134,960	0,155
EBF1	EBF transcription factor 1	6,669	0,900	0,135
SPIB	Spi-B transcription factor	14,449	1,799	0,125
HDAC9	histone deacetylase 9	332,320	39,588	0,119
TWIST2	twist family bHLH transcription factor 2	17,783	1,799	0,101
TFAP2C	transcription factor AP-2 gamma	25,563	1,799	0,070
GDAP1	ganglioside induced differentiation associated protein 1	100,029	5,398	0,054
SAMSN1	SAM domain, SH3 domain and nuclear localization signals 1	184,499	5,398	0,029
KDM5D	lysine demethylase 5D	553,496	0,900	0,002

LPS100		control	Δmen19	ratio
MNDA	myeloid cell nuclear differentiation antigen	245,177	1499,355	6,115
MTURN	maturin, neural progenitor differentiation regulator homolog	51,680	255,439	4,943
RAVER2	ribonucleoprotein, PTB binding 2	87,735	428,506	4,884
RNASE2	ribonuclease A family member 2	567,273	2410,450	4,249
HDAC4	histone deacetylase 4	676,642	2322,252	3,432
GATA2	GATA binding protein 2	33,652	114,823	3,412
HDAC9	histone deacetylase 9	403,822	86,533	0,214
GATA3	GATA binding protein 3	22,835	2,496	0,109
NFATC2	nuclear factor of activated T cells 2	1471,065	78,213	0,053
GATA6	GATA binding protein 6	16,826	0,832	0,049
DNASE2	deoxyribonuclease 2, lysosomal	379,785	14,145	0,037
SAMSN1	SAM domain, SH3 domain and nuclear localization signals 1	627,366	17,473	0,028
TWIST2	twist family bHLH transcription factor 2	61,294	1,664	0,027
KDM5D	lysine demethylase 5D	656,210	14,977	0,023

S1C – Translation and ribosomal factors, nucleic acid modifiers

BASELINE		control	Δmen19	ratio
NSUN7	NOP2/Sun RNA methyltransferase family member 7	0,000	22,493	
EEF1A2	eukaryotic translation elongation factor 1 alpha 2	13,337	92,673	6,948
CTIF	cap binding complex dependent translation initiation factor	45,569	269,021	5,904
PPARGC1A	PPARG coactivator 1 alpha	11,114	55,784	5,019
SARS	seryl-tRNA synthetase 1 - serine	536,824	2288,927	4,264
AARS	alanyl-tRNA synthetase 1 - alanine	1134,778	3961,535	3,491
RNASE2	ribonuclease A family member 2	530,156	1765,281	3,330
GARS	glycyl-tRNA synthetase 1 - glycine	821,353	2439,183	2,970
TRMT61A	tRNA methyltransferase 61A	118,924	42,288	0,356
TRMT2B	tRNA methyltransferase 2 homolog B	261,188	82,776	0,317
RIMKLA	ribosomal modification protein rimK like family member A	27,786	4,499	0,162

MT-TD	mitochondrially encoded tRNA Asp (GAU/C) - aspartic acid	6,669	0,900	0,135
DNASE2	deoxyribonuclease 2 lysosomal	76,689	9,897	0,129
MT-TE	mitochondrially encoded tRNA Glu (GAA/G) - glutamic acid	8,892	0,900	0,101
DDX3Y	DEAD-box helicase 3 Y-linked	986,957	2,699	0,003
RPS4Y1	ribosomal protein S4 Y-linked 1	1050,309	0,900	0,001
EIF1AY	eukaryotic translation initiation factor 1A Y-linked	152,267	0,000	

LPS100				
NT5E	5'-nucleotidase ecto	9,615	54,083	5,625
RAVER2	ribonucleoprotein, PTB binding 2	87,735	428,506	4,884
RNASE2	ribonuclease A family member 2	567,273	2410,450	4,249
MT-TV	mitochondrially encoded tRNA Val (GUN) - valine	1,202	4,992	4,154
MT-TY	mitochondrially encoded tRNA Tyr (UAU/C) - tyrosine	1,202	4,992	4,154
PPARGC1A	PPARG coactivator 1 alpha	9,615	35,778	3,721
MT-TM	mitochondrially encoded tRNA Met (AUA/G) - methionine	14,422	4,160	0,288
METTL7B	methyltransferase like 7B	359,353	95,686	0,266
TPMT	thiopurine S-methyltransferase	322,096	75,717	0,235
RNASE6	ribonuclease A family member k6	27,643	3,328	0,120
MT-TD	mitochondrially encoded tRNA Asp (GAU/C) - aspartic acid	21,633	1,664	0,077
DNASE2	deoxyribonuclease 2 lysosomal	379,785	14,145	0,037
EIF1AY	eukaryotic translation initiation factor 1A Y-linked	167,057	4,992	0,030
DDX3Y	DEAD-box helicase 3 Y-linked	1022,775	28,290	0,028
RPS4Y1	ribosomal protein S4 Y-linked 1	1044,408	23,297	0,022

<u>S1D - Long noncoding RNAs, antisense transcripts, microRNAs</u>

BASELINE		control	Δmen19	ratio
MIR146A	MIR3142 host gene	0,000	51,285	
MIR616	microRNA 616	0,000	26,992	
RP11-1L12.3	BBOX1 antisense RNA 1	0,000	15,296	
VAC14-AS1	VAC14 antisense RNA 1	0,000	14,396	
AC008174.3	FSIP2 antisense RNA 1	0,000	11,697	
MIR181A1HG		105,587	19,794	0,187
RP1-249H1.4	MARCKS cis regulating lncRNA promoter of cytokines and inflammation (ROCKI)	102,252	15,296	0,150
DGUOK-AS1	DGUOK antisense RNA 1	12,226	1,799	0,147
PCED1B-AS1	PCED1B antisense RNA 1	524,599	58,483	0,111
SENCR	smooth muscle and endothelial cell enriched migration/differentiation-associated lncRNA	11,114	0,900	0,081
IL10RB-AS1		14,449	0,900	0,062
IL12A-AS1	IL12A antisense RNA 1	47,792	1,799	0,038

LPS100		control	Δmen19	ratio
PCED1B-AS1	PCED1B antisense RNA 1	343,729	69,892	0,203
RP1-249H1.4	MARCKS cis regulating lncRNA promoter of cytokines and inflammation (ROCKI)	82,928	15,809	0,191
RP4-612B15.3	CLCA4 antisense RNA 1	8,413	0,832	0,099
KIF25-AS1	KIF25 antisense RNA 1	12,019	0,832	0,069

RP11-366L20.2	HMGA2 antisense RNA 1	66,102	4,160	0,063
RP11-82L2.1	SMC2 antisense RNA 1 (head to head)	16,826	0,832	0,049
IL12A-AS1	IL12A antisense RNA 1	63,698	0,832	0,013
DPP10-AS1	DPP10 antisense RNA 1	8,413	0,000	
ZNF582-AS1	ZNF582 antisense RNA 1 (head to head)	7,211	0,000	

S1E – Interleukins and TNF systems, interferon-induced genes

BASELINE		control	Δmen19	ratio
ADAMTS20	ADAM metallopeptidase with thrombospondin type 1 motif 20	0,000	19,794	
IL8	C-X-C motif chemokine ligand 8	1,111	19,794	17,810
SLFN12L	schlafen family member 12 like	0,000	22,493	
C1QTNF3	C1q and TNF related 3	16,672	82,776	4,965
TNFAIP8	TNF alpha induced protein 8	315,648	1485,463	4,706
TNF	tumor necrosis factor	216,730	510,150	2,354
TNIK	TRAF2 and NCK interacting kinase	154,490	55,784	0,361
TNFSF13	TNF superfamily member 13	17,783	3,599	0,202
ADAMTS4	ADAM metallopeptidase with thrombospondin type 1 motif 4	12,226	1,799	0,147
IL4R	interleukin 4 receptor	198,947	27,892	0,140
TNFRSF10C	TNF receptor superfamily member 10c	6,669	0,900	0,135
IL16	interleukin 16	54,460	7,198	0,132
ADAM21	ADAM metallopeptidase domain 21	8,892	0,900	0,101
IL1RN	interleukin 1 receptor antagonist	156,713	12,596	0,080
IL18R1	interleukin 18 receptor 1	48,903	3,599	0,074
IL2RB	interleukin 2 receptor subunit beta	20,006	0,900	0,045
IL12A-AS1	IL12A antisense RNA 1	47,792	1,799	0,038
ADAM28	ADAM metallopeptidase domain 28	28,897	0,900	0,031
ADAMTS9	ADAM metallopeptidase with thrombospondin type 1 motif 9	526,822	5,398	0,010

LPS100		control	Δmen19	ratio
TNFRSF19	TNF receptor superfamily member 19	0,000	5,824	
SLFN12L	schlafen family member 12 like	1,202	30,786	25,615
IL9R	interleukin 9 receptor	1,202	8,321	6,923
IL17D	interleukin 17D	1,202	7,488	6,231
IL20RA	interleukin 20 receptor subunit alpha	1,202	4,992	4,154
TNFAIP8	TNF alpha induced protein 8	430,262	1611,681	3,746
IL8	C-X-C motif chemokine ligand 8	9,615	30,786	3,202
ADAMTS5	ADAM metallopeptidase with thrombospondin type 1 motif 5	14,422	4,160	0,288
ADAMTS4	ADAM metallopeptidase with thrombospondin type 1 motif 4	7,211	1,664	0,231
IL4R	interleukin 4 receptor	399,014	72,388	0,181
ADAMTS10	ADAM metallopeptidase with thrombospondin type 1 motif 10	90,139	13,313	0,148
IL10RA	interleukin 10 receptor subunit alpha	198,305	28,290	0,143
IRAK2	interleukin 1 receptor associated kinase 2	93,744	10,817	0,115
SLFN5	schlafen family member 5	292,050	29,122	0,100
IL4I1	interleukin 4 induced 1	486,749	43,267	0,089

TNFRSF25	TNF receptor superfamily member 25	82,928	6,656	0,080
IL2RG	interleukin 2 receptor subunit gamma	10,817	0,832	0,077
IL21R	interleukin 21 receptor	55,285	4,160	0,075
ADAMTS9	ADAM metallopeptidase with thrombospondin type 1 motif 9	462,712	29,954	0,065
ADAM28	ADAM metallopeptidase domain 28	222,342	11,649	0,052
IL16	interleukin 16	135,809	7,488	0,055
TNFRSF9	TNF receptor superfamily member 9	20,431	0,832	0,041
IL2RB	interleukin 2 receptor subunit beta	43,267	0,832	0,019
TNIP3	TNFAIP3 interacting protein 3	93,744	1,664	0,018
IL18R1	interleukin 18 receptor 1	58,891	0,832	0,014
ADAMTS3	ADAM metallopeptidase with thrombospondin type 1 motif 3	10,817	0,000	
IL18RAP	interleukin 18 receptor accessory protein	7,211	0,000	

S1F – Cluster of differentiation and other leukocyte markers

BASELINE		control	Δmen19	ratio
CD93		42,235	1454,872	34,483
MARCO	macrophage receptor with collagenous structure = SR-A6	21,594	2,223	9,709
FCGR2C	Fc fragment of IgG receptor IIc	10,003	1,799	0.180
C8G	complement C8 gamma chain	5,557	0,900	0,162
CD52	CD52 molecule	56,683	8,997	0,159
LILRB1	leukocyte immunoglobulin like receptor B1	34,455	5,398	0,157
LILRA1	leukocyte immunoglobulin like receptor A1	163,381	22,493	0,138
KIAA1462	junctional cadherin 5 associated	202,282	26,092	0,129
KIAA1462	junctional cadherin 5 associated	202,282	26,092	0,129
CD40	CD40 molecule	71,132	8,098	0,114
CD53	CD53 molecule	671,308	70,179	0,105
CD248	CD248 molecule	8,892	0,900	0,101
MPEG1	macrophage expressed 1	14,449	0,900	0,062
CD300A	CD300a molecule	417,901	23,393	0,056
CD300LB	CD300 molecule like family member b	18,894	0,900	0,048
CD180	CD180 molecule	296,754	8,098	0,027
CD36	scavenger receptor (SR-B2)	300,088	6,298	0,021
CFP	complement factor properdin	104,475	1,799	0,017
LILRB2	leukocyte immunoglobulin like receptor B2	60,018	0,900	0,015
PCDH10	protocadherin 10	316,760	3,599	0,011
FCGR2B	Fc fragment of IgG receptor IIb	12,226	0,000	

LPS100		control	Δmen19	ratio
MARCO	macrophage receptor with collagenous structure = SR-A6	0,000	8,321	
C1R	complement C1r	9,615	2,496	0,260
C5AR1	complement C5a receptor 1	57,689	14,145	0,245
LILRB1	leukocyte immunoglobulin like receptor B1	193,498	32,450	0,168
LILRA1	leukocyte immunoglobulin like receptor A1	426,657	67,396	0,158
LILRA2	leukocyte immunoglobulin like receptor A2	962,682	150,601	0,156

LILRB2	leukocyte immunoglobulin like receptor B2	300,463	17,473	0,058
LILRA5	leukocyte immunoglobulin like receptor A5	21,633	0,000	0,000
CD300C	CD300c molecule	97,350	19,969	0,205
CD180	CD180 molecule	463,914	69,060	0,149
CFP	complement factor properdin	348,537	34,946	0,100
CD248	CD248 molecule	16,826	1,664	0,099
CD36	scavenger receptor (SR-B2)	525,209	45,763	0,087
CD40	CD40 molecule	367,766	25,794	0,070
CD52	CD52 molecule	56,487	2,496	0,044
CD300A	CD300a molecule	296,857	11,649	0,039
FCGR2C	Fc fragment of IgG receptor IIc	54,083	1,664	0,031
CD48	CD48 molecule	33,652	0,832	0,025
FCGR2B	Fc fragment of IgG receptor IIb	43,267	0,832	0,019
CD80	CD80 molecule	57,689	0,000	
CD86	CD86 molecule	27,643	0,000	
CD1C	CD1c molecule	27,643	0,000	
CD1A	CD1a molecule	8,413	0,000	
CD1B	CD1b molecule	3,606	0,000	
CD1E	CD1e molecule	3,606	0,000	

S1G – Cell adhesion molecules

BASELINE		control	Δmen19	ratio
GPR126	adhesion G protein-coupled receptor G6	2,223	28,792	12,952
BAI3	adhesion G protein-coupled receptor B3	5,557	59,383	10,686
NCAM2	neural cell adhesion molecule 2	5,557	59,383	10,686
CEACAM19	CEA cell adhesion molecule 19	1,111	10,797	9,714
AMIGO1	adhesion molecule with Ig like domain 1	6,669	61,182	9,175
GPR97	adhesion G protein-coupled receptor G3	3,334	22,493	6,746
BAI2	adhesion G protein-coupled receptor B2	2,223	9,897	4,452
NCAM1	neural cell adhesion molecule 1	36,677	13,496	0,368
ICAM5	intercellular adhesion molecule 5	7,780	2,699	0,347
ICAM2	intercellular adhesion molecule 2	58,906	17,095	0,290
PVRL1	nectin cell adhesion molecule 1	45,569	12,596	0,276
GPR133	adhesion G protein-coupled receptor D1	14,449	3,599	0,249
ICAM3	intercellular adhesion molecule 3	103,364	23,393	0,226
KIRREL	kirre like nephrin family adhesion molecule 1	96,695	21,594	0,223
CEACAM21	CEA cell adhesion molecule 21	28,897	6,298	0,218
MCAM	melanoma cell adhesion molecule (CD146)	18,894	3,599	0,190
ICAM1	intercellular adhesion molecule 1	81,135	12,596	0,155
GPR124	adhesion G protein-coupled receptor A2	262,299	37,789	0,144
ITGB7	integrin subunit beta 7	58,906	8,098	0,137
ESAM	endothelial cell adhesion molecule	17,783	1,799	0,101
ITGAM	integrin subunit alpha M (CD11b)	294,531	11,697	0,040
ICAM4	intercellular adhesion molecule 4	13,337	0,000	0,000
NEGR1	neuronal growth regulator 1	74,466	2,699	0,036

AMICA1	junction adhesion molecule like	7,780	0,000	0,000

LPS100		control	Δmen19	ratio
CDH2	cadherin 2	0,000	3,328	
NCAM2	neural cell adhesion molecule 2	1,202	49,091	40,846
CIB3	calcium and integrin binding family member 3	1,202	20,801	17,308
AMIGO1	adhesion molecule with Ig like domain 1	4,807	74,885	15,577
BAI3	adhesion G protein-coupled receptor B3	6,009	33,282	5,538
GPR126	adhesion G protein-coupled receptor G6	8,413	30,786	3,659
MCAM	melanoma cell adhesion molecule (CD146)	27,643	7,488	0,271
ITGAL	integrin subunit alpha L	352,142	69,060	0,196
ITGB7	integrin subunit beta 7	74,515	14,145	0,190
L1CAM	L1 cell adhesion molecule	161,048	20,801	0,129
GPR124	adhesion G protein-coupled receptor A2	218,737	25,794	0,118
NCAM1	neural cell adhesion molecule 1	90,139	9,985	0,111
VCAM1	vascular cell adhesion molecule 1	42,065	4,160	0,099
CADM1	cell adhesion molecule 1	8,413	0,832	0,099
ITGAX	integrin subunit alpha X (CD11c)	1028,784	92,358	0,090
ICAM1	intercellular adhesion molecule 1	618,953	54,083	0,087
ITGA6	integrin subunit alpha 6	325,701	28,290	0,087
EMR1	adhesion G protein-coupled receptor E1	58,891	4,160	0,071
ITGAM	integrin subunit alpha M (CD11b)	835,286	33,282	0,040
ITGA1	integrin subunit alpha 1	58,891	1,664	0,028
ICAM4	intercellular adhesion molecule 4	7,211	0,000	
CEACAM1	CEA cell adhesion molecule 1	6,009	0,000	
ESAM	endothelial cell adhesion molecule	6,009	0,000	
EMR3	adhesion G protein-coupled receptor E3	4,807	0,000	
CEACAM3	CEA cell adhesion molecule 3	3,606	0,000	

S1H – Growth and angiogenic factor systems

BASELINE		control	Δmen19	ratio
GREB1	growth regulating estrogen receptor binding 1	0,000	26,092	
LTBP1	latent transforming growth factor beta binding protein 1	0,000	9,897	
GADD45G	growth arrest and DNA damage inducible gamma	0,000	5,398	
GDF9	growth differentiation factor 9	1,111	13,496	
GDF6	growth differentiation factor 6	2,223	23,393	
FGF9	fibroblast growth factor 9	2,223	21,594	12,143
IGFBPL1	insulin like growth factor binding protein like 1	2,223	16,195	10,524
GDF15	growth differentiation factor 15	2,223	16,195	9,714
LTBP2	latent transforming growth factor beta binding protein 2	2,223	11,697	7,286
TGFB2	transforming growth factor beta 2	188,944	634,313	7,286
CSF1	colony stimulating factor 1 = macrophage colony stimulating factor (M-CSF)	2.223	13.496	6.060
TGFBR3L	transforming growth factor beta receptor 3 like	16,672	47,686	5,262
VEGFA	vascular endothelial growth factor A	17,783	6,298	3,357

LTBP3	latent transforming growth factor beta binding protein 3	157,824	51,285	2,860
FGFR3	fibroblast growth factor receptor 3	40,012	11,697	0,354
FGFR1	fibroblast growth factor receptor 1	1571,573	364,393	0,325
GAS2L1	growth arrest specific 2 like 1	8,892	1,799	0,292
IGF2BP1	insulin like growth factor 2 mRNA binding protein 1	74,466	2,699	0,232
GRTP1	growth hormone regulated TBC protein 1	14,449	0,000	0,202
CSF1R	CSF1 receptor	704,652	98,071	0,139
TGFA	transforming growth factor alpha	6,669	0,000	0,067
NEGR1	neuronal growth regulator 1	74,466	2,699	0,036
PDGFD	platelet derived growth factor D	14,449	0,000	
PGF	placental growth factor	6,669	0,000	

LPS100		control	Δmen19	
TGFB2	transforming growth factor beta 2	0,000	41,603	
LTBP1	latent transforming growth factor beta binding protein 1	0,000	7,488	
IGFBP4	insulin like growth factor binding protein 4	52,881	297,874	5,633
TGFBR3L	transforming growth factor beta receptor 3 like	3,606	19,137	5,308
IGFBPL1	insulin like growth factor binding protein like 1	7,211	34,114	4,731
PDGFC	platelet derived growth factor C	51,680	168,906	3,268
GRB10	growth factor receptor bound protein 10	255,994	814,577	3,182
VEGFA	vascular endothelial growth factor A	192,296	341,141	1,774
GAS7	growth arrest specific 7	4074,273	1399,509	0,343
TGFB3	transforming growth factor beta 3	30,046	9,985	0,332
TGFBI	transforming growth factor beta induced	694,670	219,661	0,316
GDF9	growth differentiation factor 9	6,009	1,664	0,277
IGF2BP1	insulin like growth factor 2 mRNA binding protein 1	2573,162	546,657	0,212
CSF1R	CSF1 receptor	1118,923	212,173	0,190
GRTP1	growth hormone regulated TBC protein 1	13,220	2,496	0,189
GAS1	growth arrest specific 1	4,807	0,832	0,173
ANGPTL6	angiopoietin like 6	16,826	2,496	0,148
FGF17	fibroblast growth factor 17	8,413	0,832	0,099
IGF2	insulin like growth factor 2	55,285	4,160	0,075
PDGFRL	platelet derived growth factor receptor like	15,624	0,832	0,053
NEGR1	neuronal growth regulator 1	222,342	9,153	0,041
ANGPT1	angiopoietin 1	8,413	0,000	0,000

S1I – Chemokines and chemokine receptors

BASELINE		control	Δmen19	ratio
CCL20	C-C motif chemokine ligand 20	1,111	25,193	22,667
CCR2	C-C motif chemokine receptor 2	434,572	160,153	0,369
CXCR3	C-X-C motif chemokine receptor 3	13,337	0,900	0,067
CX3CR1	C-X3-C motif chemokine receptor 1 = fractalkine	843,581	13,496	0,016
CCR7	C-C motif chemokine receptor 7	7,780	0,000	0,000

LPS100		control	Δmen19	ratio
CXCR4	C-X-C motif chemokine receptor 4	242,774	865,332	3,564
CXCL14	C-X-C motif chemokine ligand 14	1,202	4,160	3,462
IL8	C-X-C motif chemokine ligand 8	9,615	30,786	3,202
CCL20	C-C motif chemokine ligand 20	4,807	14,977	3,115
CCR2	C-C motif chemokine receptor 2	189,892	559,138	2,944
CXCL10	C-X-C motif chemokine ligand 10	75,717	14,977	0,198
CXCL11	C-X-C motif chemokine ligand 11	4,807	0,832	0,173
CXCL13	C-X-C motif chemokine ligand 13	44,468	4,992	0,112
CX3CR1	C-X3-C motif chemokine receptor 1= fractalkine	735,532	81,541	0,111
CCR7	C-C motif chemokine receptor 7	73,313	1,664	0,023
CXCR3	C-X-C motif chemokine receptor 3	38,459	0,832	0,022
CXCL16	C-X-C motif chemokine ligand 16	13,220	0,000	
ACKR3	atypical chemokine receptor 3	7,211	0,000	
CCR9	C-C motif chemokine receptor 9	6,009	0,000	
CCR10	C-C motif chemokine receptor 10	6,009	0,000	

Supplemental Table S2

RNA-seq FPKM data for ΔmascRNA monocytes

S2A - Innate immune sensors

BASELINE		control	ΔmascA4	ratio
IFITM10	interferon induced transmembrane protein 10	2,121	18,385	8,667
IFITM3	interferon induced transmembrane protein 3	27,577	156,978	5,692
IFITM1	interferon induced transmembrane protein 1	9,899	45,255	4,571
CIITA	class II major histocompatibility complex transactivator	42,426	182,434	4,300
NAIP	NLR family apoptosis inhibitory protein	42,426	148,492	3,500
KIAA1324	endosome-lysosome associated apoptosis and autophagy regulator 1	1,414	4,243	3,000
IFRD2	interferon related developmental regulator 2	955,301	308,299	0,323
AEN	apoptosis enhancing nuclease	661,852	178,191	0,269
PERP	p53 apoptosis effector related to PMP22	164,756	32,527	0,197

S2B – Transcription and nuclear factors, epigenetic modifiers

BASELINE		control	ΔmascA4	ratio
SP5	Sp5 transcription factor	1,414	28,284	20,000
TP53INP1	tumor protein p53 inducible nuclear protein 1	22,627	302,642	13,375
SIM1	SIM bHLH transcription factor 1	3,536	45,255	12,800
NPIPB9	nuclear pore complex interacting protein family member B9	0,707	8,485	12,000
ETV1	ETS variant transcription factor 1	21,920	165,463	7,548
ELF3	E74 like ETS transcription factor 3	0,707	4,243	6,000
HDAC11	histone deacetylase 11	1,414	8,485	6,000
CHD5	chromodomain helicase DNA binding protein 5	6,364	36,770	5,778

METTL7A	methyltransferase like 7A	53,033	206,475	3,893
NFATC4	nuclear factor of activated T cells 4	67,882	260,215	3,833
DNASE1	deoxyribonuclease 1	436,285	1159,655	2,658
CCPG1	cell cycle progression 1	86,974	181,019	2,081
ETV4	ETS variant transcription factor 4	77,075	9,899	0,128

S2C – Translation and ribosomal factors, nucleic acid modifiers

BASELINE		control	ΔmascA4	ratio
RNASE6	ribonuclease A family member k6	18,385	57,983	3,154
DNASE1	deoxyribonuclease 1	436,285	1159,655	2,658
MRPS26	mitochondrial ribosomal protein S26	262,337	82,024	0,313
MRPS12	mitochondrial ribosomal protein S12	458,205	132,936	0,290
RRP9	ribosomal RNA processing 9, U3 small nucleolar RNA binding protein	498,510	123,037	0,247
NT5C1A	5'-nucleotidase, cytosolic IA	13,435	1,414	0,105

S2D - Long noncoding RNAs, antisense transcripts, microRNAs

BASELINE		control	ΔmascA4	ratio
RP11-443A13.5	KCNMA1 antisense RNA 1	0,000	24,042	
AC078883.3	ITGA6 antisense RNA 1	0,000	12,728	
RP11-496I9.1	LMNTD2 antisense RNA 1	0,000	12,728	
RP1-249H1.4	MARCKS cis regulating lncRNA promoter of cytokines and inflammation (ROCKI)	294,156	934,795	3,178
MIR3648	microRNA 3648-2	3661,399	574,171	0,157
MIRLET7D	microRNA let-7d	12,728	1,414	0,111
CELF2-AS1	CELF2 antisense RNA 1	14,142	1,414	0,100

S2E – Interleukins and TNF systems, interferon-induced genes

BASELINE		control	ΔmascA4	ratio
ADAMTS4	ADAM metallopeptidase with thrombospondin type 1 motif 4	4,243	26,870	6,333
SLFN5	schlafen family member 5	38,184	162,635	4,259
IL17C	interleukin 17C	0,707	2,828	4,000
IL1B	interleukin 1 beta	2,121	8,485	4,000
IL10RA	interleukin 10 receptor subunit alpha	32,527	100,409	3,087
ADAMTS10	ADAM metallopeptidase with thrombospondin type 1 motif 10	76,368	224,860	2,944
ADAM33	ADAM metallopeptidase domain 33	2,121	5,657	2,667
ADAMTS6	ADAM metallopeptidase with thrombospondin type 1 motif 6	17,678	46,669	2,640
IL8	C-X-C motif chemokine ligand 8	2,121	4,243	2,000
TRAF5	TNF receptor associated factor 5	33,234	5,657	0,170
IL12A	interleukin 12A	15,556	1,414	0,091

S2F – Cluster of differentiation and other leukocyte markers

BASELINE		control	ΔmascA4	ratio
FCGR2C	Fc fragment of IgG receptor IIc	7,778	288,500	37,091

FCGR2B	Fc fragment of IgG receptor IIb	7,071	207,889	29,400
LSP1	lymphocyte specific protein 1	12,728	309,713	24,333
C1R	complement C1r	0,707	12,728	18,000
C5AR2	complement component 5a receptor 2	1,414	18,385	13,000
LAX1	lymphocyte transmembrane adaptor 1	1,414	9,899	7,000
CFP	complement factor properdin	71,418	434,164	6,079
LILRA5	leukocyte immunoglobulin like receptor A5	1,414	5,657	4,000
LILRA4	leukocyte immunoglobulin like receptor A4	0,707	2,828	4,000
MST1	macrophage stimulating 1	34,648	138,593	4,000
C5AR1	complement C5a receptor 1	14,142	55,154	3,900
LILRB3	leukocyte immunoglobulin like receptor B3	2,121	7,071	3,333
LILRA6	leukocyte immunoglobulin like receptor A6	1,414	4,243	3,000
MPEG1	macrophage expressed 1	14,849	43,841	2,952
CD93	CD93 molecule	824,487	2,828	0,003

S2G – Cell adhesion molecules

BASELINE		control	ΔmascA4	ratio
JAM2	junctional adhesion molecule 2	0,000	15,556	
NCKAP1	NCK associated protein 1	0,707	9,899	14,000
CEACAM1	CEA cell adhesion molecule 1	0,707	8,485	12,000
KIRREL3	kirre like nephrin family adhesion molecule 3	0,707	7,071	10,000
CEACAM6	CEA cell adhesion molecule 6	103,945	840,043	8,082
AMICA1	junction adhesion molecule like	3,536	21,213	6,000
CEACAM4	CEA cell adhesion molecule 4	12,021	59,397	4,941
ITGAM	integrin subunit alpha M	298,399	1364,716	4,573
CEACAM19	CEA cell adhesion molecule 19	4,243	18,385	4,333
PCDH11X	protocadherin 11 X-linked	4,950	19,799	4,000
CDHR4	cadherin related family member 4	0,707	2,828	4,000
SMAGP	small cell adhesion glycoprotein	41,719	161,220	3,864
ITGA9	integrin subunit alpha 9	25,456	89,095	3,500
ITGAX	integrin subunit alpha X	328,805	1093,187	3,325
ICAM1	intercellular adhesion molecule 1	57,983	190,919	3,293
ICAM4	intercellular adhesion molecule 4	16,263	49,497	3,043
PCDHB9	protocadherin beta 9	4,950	14,142	2,857
CDH23	cadherin related 23	45,962	130,108	2,831
KIAA1462	junctional cadherin 5 associated	502,046	155,563	0,310
PCDH17	protocadherin 17	118,794	5,657	0,048

S2H – Growth and angiogenic factor systems

BASELINE		control	ΔmascA4	ratio
ANGPT4	angiopoietin 4	0,000	5,657	
ANGPT1	angiopoietin 1	0,000	5,657	
IGFALS	insulin like growth factor binding protein acid labile subunit	0,000	5,657	

ANG	angiogenin	0,707	8,485	12,000
LTBP2	latent transforming growth factor beta binding protein 2	2,121	18,385	8,667
GDF7	growth differentiation factor 7	1,414	11,314	8,000
TGFA	transforming growth factor alpha	3,536	28,284	8,000
FGF11	fibroblast growth factor 11	4,243	32,527	7,667
ANGPTL4	angiopoietin like 4	1,414	8,485	6,000
FGF17	fibroblast growth factor 17	2,828	15,556	5,500
GDF5	growth differentiation factor 5	0,707	2,828	4,000
NGFR	nerve growth factor receptor	2,828	8,485	3,000
GADD45G	growth arrest and DNA damage inducible gamma	1,414	4,243	3,000
VEGFA	vascular endothelial growth factor A	456,084	1294,005	2,837
TGFBI	transforming growth factor beta induced	717,006	2022,325	2,821
CSF1	colony stimulating factor 1 = macrophage colony-stimulating factor (M-CSF)	4,950	1,414	0,286

<u>S2I – Chemokines and chemokine receptors</u>

BASELINE		control	ΔmascA4	ratio
CXCL16	C-X-C motif chemokine ligand 16	1,414	11,314	8,000
CCL22	C-C motif chemokine ligand 22	4,950	22,627	4,571
ACKR2	atypical chemokine receptor 2	6,364	28,284	4,444
CCL24	C-C motif chemokine ligand 24	4,243	18,385	4,333
CCR10	C-C motif chemokine receptor 10	0,707	2,828	4,000
CRLF1	cytokine receptor like factor 1	2,828	11,314	4,000
CRLF2	cytokine receptor like factor 2	1,414	5,657	4,000

Supplemental Table S3

Genes in the KEGG pathways displayed in Figure 2C

S3A - Chemokines and chemokine receptors

https://www.gsea-msigdb.org/gsea/msigdb/human/geneset/KEGG_CHEMOKINE_SIGNALING_PATHWAY.html (189 genes)

NCBI (Entrez) Gene Id	Gene Symbol	Gene Description
10000	AKT3	AKT serine/threonine kinase 3 [Source:HGNC ...
10235	RASGRP2	RAS guanyl releasing protein 2 [Source:HGNC...
10344	CCL26	C-C motif chemokine ligand 26 [Source:HGNC ...
10451	VAV3	vav guanine nucleotide exchange factor 3 [S...
10563	CXCL13	C-X-C motif chemokine ligand 13 [Source:HGN...
10663	CXCR6	C-X-C motif chemokine receptor 6 [Source:HG...
10681	GNB5	G protein subunit beta 5 [Source:HGNC Symbo...
107	ADCY1	adenylate cyclase 1 [Source:HGNC Symbol;Acc...
108	ADCY2	adenylate cyclase 2 [Source:HGNC Symbol;Acc...
10803	CCR9	C-C motif chemokine receptor 9 [Source:HGNC...
10850	CCL27	C-C motif chemokine ligand 27 [Source:HGNC ...
109	ADCY3	adenylate cyclase 3 [Source:HGNC Symbol;Acc...
111	ADCY5	adenylate cyclase 5 [Source:HGNC Symbol;Acc...
112	ADCY6	adenylate cyclase 6 [Source:HGNC Symbol;Acc...
113	ADCY7	adenylate cyclase 7 [Source:HGNC Symbol;Acc...
114	ADCY8	adenylate cyclase 8 [Source:HGNC Symbol;Acc...
1147	CHUK	component of inhibitor of nuclear factor ka...
115	ADCY9	adenylate cyclase 9 [Source:HGNC Symbol;Acc...
1230	CCR1	C-C motif chemokine receptor 1 [Source:HGNC...
1232	CCR3	C-C motif chemokine receptor 3 [Source:HGNC...
1233	CCR4	C-C motif chemokine receptor 4 [Source:HGNC...
1234	CCR5	C-C motif chemokine receptor 5 [Source:HGNC...
1235	CCR6	C-C motif chemokine receptor 6 [Source:HGNC...
1236	CCR7	C-C motif chemokine receptor 7 [Source:HGNC...
1237	CCR8	C-C motif chemokine receptor 8 [Source:HGNC...
131890	GRK7	G protein-coupled receptor kinase 7 [Source...
1398	CRK	CRK proto-oncogene, adaptor protein [Source...
1399	CRKL	CRK like proto-oncogene, adaptor protein [S...
1445	CSK	C-terminal Src kinase [Source:HGNC Symbol;A...
1524	CX3CR1	C-X3-C motif chemokine receptor 1 [Source:H...
156	GRK2	G protein-coupled receptor kinase 2 [Source...
157	GRK3	G protein-coupled receptor kinase 3 [Source...
1794	DOCK2	dedicator of cytokinesis 2 [Source:HGNC Sym...
196883	ADCY4	adenylate cyclase 4 [Source:HGNC Symbol;Acc...
207	AKT1	AKT serine/threonine kinase 1 [Source:HGNC ...
208	AKT2	AKT serine/threonine kinase 2 [Source:HGNC ...
2185	PTK2B	protein tyrosine kinase 2 beta [Source:HGNC...
2268	FGR	FGR proto-oncogene, Src family tyrosine kin...
2309	FOXO3	forkhead box O3 [Source:HGNC Symbol;Acc:HGN...

23236	PLCB1	phospholipase C beta 1 [Source:HGNC Symbol;...
23533	PIK3R5	phosphoinositide-3-kinase regulatory subuni...
25759	SHC2	SHC adaptor protein 2 [Source:HGNC Symbol;A...
26230	TIAM2	TIAM Rac1 associated GEF 2 [Source:HGNC Sym...
2770	GNAI1	G protein subunit alpha i1 [Source:HGNC Sym...
2771	GNAI2	G protein subunit alpha i2 [Source:HGNC Sym...
2773	GNAI3	G protein subunit alpha i3 [Source:HGNC Sym...
2782	GNB1	G protein subunit beta 1 [Source:HGNC Symbo...
2783	GNB2	G protein subunit beta 2 [Source:HGNC Symbo...
2784	GNB3	G protein subunit beta 3 [Source:HGNC Symbo...
2785	GNG3	G protein subunit gamma 3 [Source:HGNC Symb...
2786	GNG4	G protein subunit gamma 4 [Source:HGNC Symb...
2787	GNG5	G protein subunit gamma 5 [Source:HGNC Symb...
2788	GNG7	G protein subunit gamma 7 [Source:HGNC Symb...
2790	GNG10	G protein subunit gamma 10 [Source:HGNC Sym...
2791	GNG11	G protein subunit gamma 11 [Source:HGNC Sym...
2792	GNGT1	G protein subunit gamma transducin 1 [Sourc...
2793	GNGT2	G protein subunit gamma transducin 2 [Sourc...
2826	CCR10	C-C motif chemokine receptor 10 [Source:HGN...
2829	XCR1	X-C motif chemokine receptor 1 [Source:HGNC...
2833	CXCR3	C-X-C motif chemokine receptor 3 [Source:HG...
2868	GRK4	G protein-coupled receptor kinase 4 [Source...
2869	GRK5	G protein-coupled receptor kinase 5 [Source...
2870	GRK6	G protein-coupled receptor kinase 6 [Source...
2885	GRB2	growth factor receptor bound protein 2 [Sou...
2919	CXCL1	C-X-C motif chemokine ligand 1 [Source:HGNC...
2920	CXCL2	C-X-C motif chemokine ligand 2 [Source:HGNC...
2921	CXCL3	C-X-C motif chemokine ligand 3 [Source:HGNC...
2931	GSK3A	glycogen synthase kinase 3 alpha [Source:HG...
2932	GSK3B	glycogen synthase kinase 3 beta [Source:HGN...
3055	HCK	HCK proto-oncogene, Src family tyrosine kin...
3265	HRAS	HRas proto-oncogene, GTPase [Source:HGNC Sy...
3551	IKBKB	inhibitor of nuclear factor kappa B kinase ...
3576	CXCL8	C-X-C motif chemokine ligand 8 [Source:HGNC...
3577	CXCR1	C-X-C motif chemokine receptor 1 [Source:HG...
3579	CXCR2	C-X-C motif chemokine receptor 2 [Source:HG...
3627	CXCL10	C-X-C motif chemokine ligand 10 [Source:HGN...
3702	ITK	IL2 inducible T cell kinase [Source:HGNC Sy...
3717	JAK2	Janus kinase 2 [Source:HGNC Symbol;Acc:HGNC...
3718	JAK3	Janus kinase 3 [Source:HGNC Symbol;Acc:HGNC...
3845	KRAS	KRAS proto-oncogene, GTPase [Source:HGNC Sy...
387	RHOA	ras homolog family member A [Source:HGNC Sy...
399694	SHC4	SHC adaptor protein 4 [Source:HGNC Symbol;A...
4067	LYN	LYN proto-oncogene, Src family tyrosine kin...
408	ARRB1	arrestin beta 1 [Source:HGNC Symbol;Acc:HGN...
409	ARRB2	arrestin beta 2 [Source:HGNC Symbol;Acc:HGN...

414062	CCL3L3	C-C motif chemokine ligand 3 like 3 [Source...
4283	CXCL9	C-X-C motif chemokine ligand 9 [Source:HGNC...
4790	NFKB1	nuclear factor kappa B subunit 1 [Source:HG...
4792	NFKBIA	NFKB inhibitor alpha [Source:HGNC Symbol;Ac...
4793	NFKBIB	NFKB inhibitor beta [Source:HGNC Symbol;Acc...
4893	NRAS	NRAS proto-oncogene, GTPase [Source:HGNC Sy...
5058	PAK1	p21 (RAC1) activated kinase 1 [Source:HGNC ...
51764	GNG13	G protein subunit gamma 13 [Source:HGNC Sym...
5196	PF4	platelet factor 4 [Source:HGNC Symbol;Acc:H...
5197	PF4V1	platelet factor 4 variant 1 [Source:HGNC Sy...
5290	PIK3CA	phosphatidylinositol-4,5-bisphosphate 3-kin...
5291	PIK3CB	phosphatidylinositol-4,5-bisphosphate 3-kin...
5293	PIK3CD	phosphatidylinositol-4,5-bisphosphate 3-kin...
5294	PIK3CG	phosphatidylinositol-4,5-bisphosphate 3-kin...
5295	PIK3R1	phosphoinositide-3-kinase regulatory subuni...
5296	PIK3R2	phosphoinositide-3-kinase regulatory subuni...
5330	PLCB2	phospholipase C beta 2 [Source:HGNC Symbol;...
5331	PLCB3	phospholipase C beta 3 [Source:HGNC Symbol;...
5332	PLCB4	phospholipase C beta 4 [Source:HGNC Symbol;...
53358	SHC3	SHC adaptor protein 3 [Source:HGNC Symbol;A...
54331	GNG2	G protein subunit gamma 2 [Source:HGNC Symb...
5473	PPBP	pro-platelet basic protein [Source:HGNC Sym...
5566	PRKACA	protein kinase cAMP-activated catalytic sub...
5567	PRKACB	protein kinase cAMP-activated catalytic sub...
5568	PRKACG	protein kinase cAMP-activated catalytic sub...
5579	PRKCB	protein kinase C beta [Source:HGNC Symbol;A...
5580	PRKCD	protein kinase C delta [Source:HGNC Symbol;...
5590	PRKCZ	protein kinase C zeta [Source:HGNC Symbol;A...
5594	MAPK1	mitogen-activated protein kinase 1 [Source:...
5595	MAPK3	mitogen-activated protein kinase 3 [Source:...
55970	GNG12	G protein subunit gamma 12 [Source:HGNC Sym...
5604	MAP2K1	mitogen-activated protein kinase kinase 1 [...
5613	PRKX	protein kinase X-linked [Source:HGNC Symbol...
56288	PARD3	par-3 family cell polarity regulator [Sourc...
56477	CCL28	C-C motif chemokine ligand 28 [Source:HGNC ...
5747	PTK2	protein tyrosine kinase 2 [Source:HGNC Symb...
57580	PREX1	phosphatidylinositol-3,4,5-trisphosphate de...
58191	CXCL16	C-X-C motif chemokine ligand 16 [Source:HGN...
5829	PXN	paxillin [Source:HGNC Symbol;Acc:HGNC:9718]
5879	RAC1	Rac family small GTPase 1 [Source:HGNC Symb...
5880	RAC2	Rac family small GTPase 2 [Source:HGNC Symb...
5894	RAF1	Raf-1 proto-oncogene, serine/threonine kina...
5906	RAP1A	RAP1A, member of RAS oncogene family [Sourc...
5908	RAP1B	RAP1B, member of RAS oncogene family [Sourc...
59345	GNB4	G protein subunit beta 4 [Source:HGNC Symbo...
5970	RELA	RELA proto-oncogene, NF-kB subunit [Source:...

6011	GRK1	G protein-coupled receptor kinase 1 [Source...
6093	ROCK1	Rho associated coiled-coil containing prote...
6346	CCL1	C-C motif chemokine ligand 1 [Source:HGNC S...
6347	CCL2	C-C motif chemokine ligand 2 [Source:HGNC S...
6348	CCL3	C-C motif chemokine ligand 3 [Source:HGNC S...
6349	CCL3L1	C-C motif chemokine ligand 3 like 1 [Source...
6351	CCL4	C-C motif chemokine ligand 4 [Source:HGNC S...
6352	CCL5	C-C motif chemokine ligand 5 [Source:HGNC S...
6354	CCL7	C-C motif chemokine ligand 7 [Source:HGNC S...
6355	CCL8	C-C motif chemokine ligand 8 [Source:HGNC S...
6356	CCL11	C-C motif chemokine ligand 11 [Source:HGNC ...
6357	CCL13	C-C motif chemokine ligand 13 [Source:HGNC ...
6358	CCL14	C-C motif chemokine ligand 14 [Source:HGNC ...
6359	CCL15	C-C motif chemokine ligand 15 [Source:HGNC ...
6360	CCL16	C-C motif chemokine ligand 16 [Source:HGNC ...
6361	CCL17	C-C motif chemokine ligand 17 [Source:HGNC ...
6362	CCL18	C-C motif chemokine ligand 18 [Source:HGNC ...
6363	CCL19	C-C motif chemokine ligand 19 [Source:HGNC ...
6364	CCL20	C-C motif chemokine ligand 20 [Source:HGNC ...
6366	CCL21	C-C motif chemokine ligand 21 [Source:HGNC ...
6367	CCL22	C-C motif chemokine ligand 22 [Source:HGNC ...
6368	CCL23	C-C motif chemokine ligand 23 [Source:HGNC ...
6369	CCL24	C-C motif chemokine ligand 24 [Source:HGNC ...
6370	CCL25	C-C motif chemokine ligand 25 [Source:HGNC ...
6372	CXCL6	C-X-C motif chemokine ligand 6 [Source:HGNC...
6373	CXCL11	C-X-C motif chemokine ligand 11 [Source:HGN...
6374	CXCL5	C-X-C motif chemokine ligand 5 [Source:HGNC...
6375	XCL1	X-C motif chemokine ligand 1 [Source:HGNC S...
6376	CX3CL1	C-X3-C motif chemokine ligand 1 [Source:HGN...
6387	CXCL12	C-X-C motif chemokine ligand 12 [Source:HGN...
643	CXCR5	C-X-C motif chemokine receptor 5 [Source:HG...
6464	SHC1	SHC adaptor protein 1 [Source:HGNC Symbol;A...
653361	NCF1	neutrophil cytosolic factor 1 [Source:HGNC ...
6654	SOS1	SOS Ras/Rac guanine nucleotide exchange fac...
6655	SOS2	SOS Ras/Rho guanine nucleotide exchange fac...
673	BRAF	B-Raf proto-oncogene, serine/threonine kina...
6772	STAT1	signal transducer and activator of transcri...
6773	STAT2	signal transducer and activator of transcri...
6774	STAT3	signal transducer and activator of transcri...
6777	STAT5B	signal transducer and activator of transcri...
6846	XCL2	X-C motif chemokine ligand 2 [Source:HGNC S...
7074	TIAM1	TIAM Rac1 associated GEF 1 [Source:HGNC Sym...
728045	PPBPP1	pro-platelet basic protein pseudogene 1 [So...
729230	CCR2	C-C motif chemokine receptor 2 [Source:HGNC...
7409	VAV1	vav guanine nucleotide exchange factor 1 [S...
7410	VAV2	vav guanine nucleotide exchange factor 2 [S...

7454	WAS	WASP actin nucleation promoting factor [Sou...
7852	CXCR4	C-X-C motif chemokine receptor 4 [Source:HG...
8503	PIK3R3	phosphoinositide-3-kinase regulatory subuni...
8517	IKBKG	inhibitor of nuclear factor kappa B kinase ...
8976	WASL	WASP like actin nucleation promoting factor...
94235	GNG8	G protein subunit gamma 8 [Source:HGNC Symb...
9475	ROCK2	Rho associated coiled-coil containing prote...
9547	CXCL14	C-X-C motif chemokine ligand 14 [Source:HGN...
9560	CCL4L2	C-C motif chemokine ligand 4 like 2 [Source...
9564	BCAR1	BCAR1 scaffold protein, Cas family member [...
9844	ELMO1	engulfment and cell motility 1 [Source:HGNC...
998	CDC42	cell division cycle 42 [Source:HGNC Symbol;...

S3B - Primary immunodeficiency

https://www.gsea-msigdb.org/gsea/msigdb/human/geneset/KEGG_PRIMARY_IMMUNODEFICIENCY.html

(35 genes)

NCBI (Entrez) Gene Id	Gene Symbol	Gene Description
100	ADA	adenosine deaminase [Source:HGNC Symbol;A...
115650	TNFRSF13C	TNF receptor superfamily member 13C [Sour...
23495	TNFRSF13B	TNF receptor superfamily member 13B [Sour...
29760	BLNK	B cell linker [Source:HGNC Symbol;Acc:HGN...
29851	ICOS	inducible T cell costimulator [Source:HGN...
326	AIRE	autoimmune regulator [Source:HGNC Symbol;...
3543	IGLL1	immunoglobulin lambda like polypeptide 1 ...
3561	IL2RG	interleukin 2 receptor subunit gamma [Sou...
3575	IL7R	interleukin 7 receptor [Source:HGNC Symbo...
3718	JAK3	Janus kinase 3 [Source:HGNC Symbol;Acc:HG...
3932	LCK	LCK proto-oncogene, Src family tyrosine k...
4261	CIITA	class II major histocompatibility complex...
57379	AICDA	activation induced cytidine deaminase [So...
5788	PTPRC	protein tyrosine phosphatase receptor typ...
5896	RAG1	recombination activating 1 [Source:HGNC S...
5897	RAG2	recombination activating 2 [Source:HGNC S...
5993	RFX5	regulatory factor X5 [Source:HGNC Symbol;...
5994	RFXAP	regulatory factor X associated protein [S...
64421	DCLRE1C	DNA cross-link repair 1C [Source:HGNC Sym...
6890	TAP1	transporter 1, ATP binding cassette subfa...
6891	TAP2	transporter 2, ATP binding cassette subfa...
695	BTK	Bruton tyrosine kinase [Source:HGNC Symbo...
7374	UNG	uracil DNA glycosylase [Source:HGNC Symbo...
7535	ZAP70	zeta chain of T cell receptor associated ...
8517	IKBKG	inhibitor of nuclear factor kappa B kinas...
8625	RFXANK	regulatory factor X associated ankyrin co...
915	CD3D	CD3d molecule [Source:HGNC Symbol;Acc:HGN...

916	CD3E	CD3e molecule [Source:HGNC Symbol;Acc:HGN...
920	CD4	CD4 molecule [Source:HGNC Symbol;Acc:HGNC...
925	CD8A	CD8a molecule [Source:HGNC Symbol;Acc:HGN...
926	CD8B	CD8b molecule [Source:HGNC Symbol;Acc:HGN...
930	CD19	CD19 molecule [Source:HGNC Symbol;Acc:HGN...
958	CD40	CD40 molecule [Source:HGNC Symbol;Acc:HGN...
959	CD40LG	CD40 ligand [Source:HGNC Symbol;Acc:HGNC:...
973	CD79A	CD79a molecule [Source:HGNC Symbol;Acc:HG...

S3C - Cytokine-cytokine receptor interaction

https://www.gsea-msigdb.org/gsea/msigdb/human/geneset/KEGG_CYTOKINE_CYTOKINE_RECEPTOR_INTERACTION.html (265 genes)

NCBI (Entrez) Gene Id	Gene Symbol	Gene Description
10344	CCL26	C-C motif chemokine ligand 26 [Source:HGN...
10563	CXCL13	C-X-C motif chemokine ligand 13 [Source:H...
10663	CXCR6	C-X-C motif chemokine receptor 6 [Source:...
10673	TNFSF13B	TNF superfamily member 13b [Source:HGNC S...
10803	CCR9	C-C motif chemokine receptor 9 [Source:HG...
10850	CCL27	C-C motif chemokine ligand 27 [Source:HGN...
10913	EDAR	ectodysplasin A receptor [Source:HGNC Sym...
11009	IL24	interleukin 24 [Source:HGNC Symbol;Acc:HG...
115650	TNFRSF13C	TNF receptor superfamily member 13C [Sour...
116379	IL22RA2	interleukin 22 receptor subunit alpha 2 [...
1230	CCR1	C-C motif chemokine receptor 1 [Source:HG...
1232	CCR3	C-C motif chemokine receptor 3 [Source:HG...
1233	CCR4	C-C motif chemokine receptor 4 [Source:HG...
1234	CCR5	C-C motif chemokine receptor 5 [Source:HG...
1235	CCR6	C-C motif chemokine receptor 6 [Source:HG...
1236	CCR7	C-C motif chemokine receptor 7 [Source:HG...
1237	CCR8	C-C motif chemokine receptor 8 [Source:HG...
1270	CNTF	ciliary neurotrophic factor [Source:HGNC ...
1271	CNTFR	ciliary neurotrophic factor receptor [Sou...
1435	CSF1	colony stimulating factor 1 [Source:HGNC ...
1436	CSF1R	colony stimulating factor 1 receptor [Sou...
1437	CSF2	colony stimulating factor 2 [Source:HGNC ...
1438	CSF2RA	colony stimulating factor 2 receptor subu...
1439	CSF2RB	colony stimulating factor 2 receptor subu...
1440	CSF3	colony stimulating factor 3 [Source:HGNC ...
1441	CSF3R	colony stimulating factor 3 receptor [Sou...
1489	CTF1	cardiotrophin 1 [Source:HGNC Symbol;Acc:H...
149233	IL23R	interleukin 23 receptor [Source:HGNC Symb...
1524	CX3CR1	C-X3-C motif chemokine receptor 1 [Source...
163702	IFNLR1	interferon lambda receptor 1 [Source:HGNC...
1896	EDA	ectodysplasin A [Source:HGNC Symbol;Acc:H...

1950	EGF	epidermal growth factor [Source:HGNC Symb...
1956	EGFR	epidermal growth factor receptor [Source:...
2056	EPO	erythropoietin [Source:HGNC Symbol;Acc:HG...
2057	EPOR	erythropoietin receptor [Source:HGNC Symb...
2277	VEGFD	vascular endothelial growth factor D [Sou...
2321	FLT1	fms related receptor tyrosine kinase 1 [S...
2322	FLT3	fms related receptor tyrosine kinase 3 [S...
2323	FLT3LG	fms related receptor tyrosine kinase 3 li...
2324	FLT4	fms related receptor tyrosine kinase 4 [S...
23495	TNFRSF13B	TNF receptor superfamily member 13B [Sour...
23529	CLCF1	cardiotrophin like cytokine factor 1 [Sou...
23765	IL17RA	interleukin 17 receptor A [Source:HGNC Sy...
268	AMH	anti-Mullerian hormone [Source:HGNC Symbo...
2688	GH1	growth hormone 1 [Source:HGNC Symbol;Acc:...
2689	GH2	growth hormone 2 [Source:HGNC Symbol;Acc:...
269	AMHR2	anti-Mullerian hormone receptor type 2 [S...
2690	GHR	growth hormone receptor [Source:HGNC Symb...
27190	IL17B	interleukin 17B [Source:HGNC Symbol;Acc:H...
27242	TNFRSF21	TNF receptor superfamily member 21 [Sourc...
2826	CCR10	C-C motif chemokine receptor 10 [Source:H...
282616	IFNL2	interferon lambda 2 [Source:HGNC Symbol;A...
282617	IFNL3	interferon lambda 3 [Source:HGNC Symbol;A...
282618	IFNL1	interferon lambda 1 [Source:HGNC Symbol;A...
2829	XCR1	X-C motif chemokine receptor 1 [Source:HG...
2833	CXCR3	C-X-C motif chemokine receptor 3 [Source:...
2919	CXCL1	C-X-C motif chemokine ligand 1 [Source:HG...
2920	CXCL2	C-X-C motif chemokine ligand 2 [Source:HG...
2921	CXCL3	C-X-C motif chemokine ligand 3 [Source:HG...
29949	IL19	interleukin 19 [Source:HGNC Symbol;Acc:HG...
3082	HGF	hepatocyte growth factor [Source:HGNC Sym...
338376	IFNE	interferon epsilon [Source:HGNC Symbol;Ac...
3439	IFNA1	interferon alpha 1 [Source:HGNC Symbol;Ac...
3440	IFNA2	interferon alpha 2 [Source:HGNC Symbol;Ac...
3441	IFNA4	interferon alpha 4 [Source:HGNC Symbol;Ac...
3442	IFNA5	interferon alpha 5 [Source:HGNC Symbol;Ac...
3443	IFNA6	interferon alpha 6 [Source:HGNC Symbol;Ac...
3444	IFNA7	interferon alpha 7 [Source:HGNC Symbol;Ac...
3445	IFNA8	interferon alpha 8 [Source:HGNC Symbol;Ac...
3446	IFNA10	interferon alpha 10 [Source:HGNC Symbol;A...
3447	IFNA13	interferon alpha 13 [Source:HGNC Symbol;A...
3448	IFNA14	interferon alpha 14 [Source:HGNC Symbol;A...
3449	IFNA16	interferon alpha 16 [Source:HGNC Symbol;A...
3451	IFNA17	interferon alpha 17 [Source:HGNC Symbol;A...
3452	IFNA21	interferon alpha 21 [Source:HGNC Symbol;A...
3454	IFNAR1	interferon alpha and beta receptor subuni...
3455	IFNAR2	interferon alpha and beta receptor subuni...

3456	IFNB1	interferon beta 1 [Source:HGNC Symbol;Acc...
3458	IFNG	interferon gamma [Source:HGNC Symbol;Acc:...
3459	IFNGR1	interferon gamma receptor 1 [Source:HGNC ...
3460	IFNGR2	interferon gamma receptor 2 [Source:HGNC ...
3467	IFNW1	interferon omega 1 [Source:HGNC Symbol;Ac...
355	FAS	Fas cell surface death receptor [Source:H...
3552	IL1A	interleukin 1 alpha [Source:HGNC Symbol;A...
3553	IL1B	interleukin 1 beta [Source:HGNC Symbol;Ac...
3554	IL1R1	interleukin 1 receptor type 1 [Source:HGN...
3556	IL1RAP	interleukin 1 receptor accessory protein ...
3558	IL2	interleukin 2 [Source:HGNC Symbol;Acc:HGN...
3559	IL2RA	interleukin 2 receptor subunit alpha [Sou...
356	FASLG	Fas ligand [Source:HGNC Symbol;Acc:HGNC:1...
3560	IL2RB	interleukin 2 receptor subunit beta [Sour...
3561	IL2RG	interleukin 2 receptor subunit gamma [Sou...
3562	IL3	interleukin 3 [Source:HGNC Symbol;Acc:HGN...
3563	IL3RA	interleukin 3 receptor subunit alpha [Sou...
3565	IL4	interleukin 4 [Source:HGNC Symbol;Acc:HGN...
3566	IL4R	interleukin 4 receptor [Source:HGNC Symbo...
3567	IL5	interleukin 5 [Source:HGNC Symbol;Acc:HGN...
3568	IL5RA	interleukin 5 receptor subunit alpha [Sou...
3569	IL6	interleukin 6 [Source:HGNC Symbol;Acc:HGN...
3570	IL6R	interleukin 6 receptor [Source:HGNC Symbo...
3572	IL6ST	interleukin 6 cytokine family signal tran...
3574	IL7	interleukin 7 [Source:HGNC Symbol;Acc:HGN...
3575	IL7R	interleukin 7 receptor [Source:HGNC Symbo...
3576	CXCL8	C-X-C motif chemokine ligand 8 [Source:HG...
3577	CXCR1	C-X-C motif chemokine receptor 1 [Source:...
3578	IL9	interleukin 9 [Source:HGNC Symbol;Acc:HGN...
3579	CXCR2	C-X-C motif chemokine receptor 2 [Source:...
3581	IL9R	interleukin 9 receptor [Source:HGNC Symbo...
3586	IL10	interleukin 10 [Source:HGNC Symbol;Acc:HG...
3587	IL10RA	interleukin 10 receptor subunit alpha [So...
3588	IL10RB	interleukin 10 receptor subunit beta [Sou...
3589	IL11	interleukin 11 [Source:HGNC Symbol;Acc:HG...
3590	IL11RA	interleukin 11 receptor subunit alpha [So...
3592	IL12A	interleukin 12A [Source:HGNC Symbol;Acc:H...
3593	IL12B	interleukin 12B [Source:HGNC Symbol;Acc:H...
3594	IL12RB1	interleukin 12 receptor subunit beta 1 [S...
3595	IL12RB2	interleukin 12 receptor subunit beta 2 [S...
3596	IL13	interleukin 13 [Source:HGNC Symbol;Acc:HG...
3597	IL13RA1	interleukin 13 receptor subunit alpha 1 [...
3600	IL15	interleukin 15 [Source:HGNC Symbol;Acc:HG...
3601	IL15RA	interleukin 15 receptor subunit alpha [So...
3604	TNFRSF9	TNF receptor superfamily member 9 [Source...
3605	IL17A	interleukin 17A [Source:HGNC Symbol;Acc:H...

3606	IL18	interleukin 18 [Source:HGNC Symbol;Acc:HG...
3624	INHBA	inhibin subunit beta A [Source:HGNC Symbo...
3625	INHBB	inhibin subunit beta B [Source:HGNC Symbo...
3626	INHBC	inhibin subunit beta C [Source:HGNC Symbo...
3627	CXCL10	C-X-C motif chemokine ligand 10 [Source:H...
3791	KDR	kinase insert domain receptor [Source:HGN...
3815	KIT	KIT proto-oncogene, receptor tyrosine kin...
3952	LEP	leptin [Source:HGNC Symbol;Acc:HGNC:6553]
3953	LEPR	leptin receptor [Source:HGNC Symbol;Acc:H...
3976	LIF	LIF interleukin 6 family cytokine [Source...
3977	LIFR	LIF receptor subunit alpha [Source:HGNC S...
4049	LTA	lymphotoxin alpha [Source:HGNC Symbol;Acc...
4050	LTB	lymphotoxin beta [Source:HGNC Symbol;Acc:...
4055	LTBR	lymphotoxin beta receptor [Source:HGNC Sy...
414062	CCL3L3	C-C motif chemokine ligand 3 like 3 [Sour...
4233	MET	MET proto-oncogene, receptor tyrosine kin...
4254	KITLG	KIT ligand [Source:HGNC Symbol;Acc:HGNC:6...
4283	CXCL9	C-X-C motif chemokine ligand 9 [Source:HG...
4352	MPL	MPL proto-oncogene, thrombopoietin recept...
4804	NGFR	nerve growth factor receptor [Source:HGNC...
4982	TNFRSF11B	TNF receptor superfamily member 11b [Sour...
5008	OSM	oncostatin M [Source:HGNC Symbol;Acc:HGNC...
50604	IL20	interleukin 20 [Source:HGNC Symbol;Acc:HG...
50615	IL21R	interleukin 21 receptor [Source:HGNC Symb...
50616	IL22	interleukin 22 [Source:HGNC Symbol;Acc:HG...
51330	TNFRSF12A	TNF receptor superfamily member 12A [Sour...
5154	PDGFA	platelet derived growth factor subunit A ...
5155	PDGFB	platelet derived growth factor subunit B ...
5156	PDGFRA	platelet derived growth factor receptor a...
51561	IL23A	interleukin 23 subunit alpha [Source:HGNC...
5159	PDGFRB	platelet derived growth factor receptor b...
5196	PF4	platelet factor 4 [Source:HGNC Symbol;Acc...
5197	PF4V1	platelet factor 4 variant 1 [Source:HGNC ...
53832	IL20RA	interleukin 20 receptor subunit alpha [So...
53833	IL20RB	interleukin 20 receptor subunit beta [Sou...
5473	PPBP	pro-platelet basic protein [Source:HGNC S...
55504	TNFRSF19	TNF receptor superfamily member 19 [Sourc...
55540	IL17RB	interleukin 17 receptor B [Source:HGNC Sy...
55801	IL26	interleukin 26 [Source:HGNC Symbol;Acc:HG...
56034	PDGFC	platelet derived growth factor C [Source:...
5617	PRL	prolactin [Source:HGNC Symbol;Acc:HGNC:9445]
5618	PRLR	prolactin receptor [Source:HGNC Symbol;Ac...
56477	CCL28	C-C motif chemokine ligand 28 [Source:HGN...
56832	IFNK	interferon kappa [Source:HGNC Symbol;Acc:...
58191	CXCL16	C-X-C motif chemokine ligand 16 [Source:H...
58985	IL22RA1	interleukin 22 receptor subunit alpha 1 [...

59067	IL21	interleukin 21 [Source:HGNC Symbol;Acc:HG...
60401	EDA2R	ectodysplasin A2 receptor [Source:HGNC Sy...
608	TNFRSF17	TNF receptor superfamily member 17 [Sourc...
6346	CCL1	C-C motif chemokine ligand 1 [Source:HGNC...
6347	CCL2	C-C motif chemokine ligand 2 [Source:HGNC...
6348	CCL3	C-C motif chemokine ligand 3 [Source:HGNC...
6349	CCL3L1	C-C motif chemokine ligand 3 like 1 [Sour...
6351	CCL4	C-C motif chemokine ligand 4 [Source:HGNC...
6352	CCL5	C-C motif chemokine ligand 5 [Source:HGNC...
6354	CCL7	C-C motif chemokine ligand 7 [Source:HGNC...
6355	CCL8	C-C motif chemokine ligand 8 [Source:HGNC...
6356	CCL11	C-C motif chemokine ligand 11 [Source:HGN...
6357	CCL13	C-C motif chemokine ligand 13 [Source:HGN...
6358	CCL14	C-C motif chemokine ligand 14 [Source:HGN...
6359	CCL15	C-C motif chemokine ligand 15 [Source:HGN...
6360	CCL16	C-C motif chemokine ligand 16 [Source:HGN...
6361	CCL17	C-C motif chemokine ligand 17 [Source:HGN...
6362	CCL18	C-C motif chemokine ligand 18 [Source:HGN...
6363	CCL19	C-C motif chemokine ligand 19 [Source:HGN...
6364	CCL20	C-C motif chemokine ligand 20 [Source:HGN...
6366	CCL21	C-C motif chemokine ligand 21 [Source:HGN...
6367	CCL22	C-C motif chemokine ligand 22 [Source:HGN...
6368	CCL23	C-C motif chemokine ligand 23 [Source:HGN...
6369	CCL24	C-C motif chemokine ligand 24 [Source:HGN...
6370	CCL25	C-C motif chemokine ligand 25 [Source:HGN...
6372	CXCL6	C-X-C motif chemokine ligand 6 [Source:HG...
6373	CXCL11	C-X-C motif chemokine ligand 11 [Source:H...
6374	CXCL5	C-X-C motif chemokine ligand 5 [Source:HG...
6375	XCL1	X-C motif chemokine ligand 1 [Source:HGNC...
6376	CX3CL1	C-X3-C motif chemokine ligand 1 [Source:H...
6387	CXCL12	C-X-C motif chemokine ligand 12 [Source:H...
64109	CRLF2	cytokine receptor like factor 2 [Source:H...
643	CXCR5	C-X-C motif chemokine receptor 5 [Source:...
64806	IL25	interleukin 25 [Source:HGNC Symbol;Acc:HG...
650	BMP2	bone morphogenetic protein 2 [Source:HGNC...
655	BMP7	bone morphogenetic protein 7 [Source:HGNC...
657	BMPR1A	bone morphogenetic protein receptor type ...
658	BMPR1B	bone morphogenetic protein receptor type ...
659	BMPR2	bone morphogenetic protein receptor type ...
6846	XCL2	X-C motif chemokine ligand 2 [Source:HGNC...
7040	TGFB1	transforming growth factor beta 1 [Source...
7042	TGFB2	transforming growth factor beta 2 [Source...
7043	TGFB3	transforming growth factor beta 3 [Source...
7046	TGFBR1	transforming growth factor beta receptor ...
7048	TGFBR2	transforming growth factor beta receptor ...
7124	TNF	tumor necrosis factor [Source:HGNC Symbol...

7132	TNFRSF1A	TNF receptor superfamily member 1A [Sourc...
7133	TNFRSF1B	TNF receptor superfamily member 1B [Sourc...
7173	TPO	thyroid peroxidase [Source:HGNC Symbol;Ac...
728045	PPBPP1	pro-platelet basic protein pseudogene 1 [...
7292	TNFSF4	TNF superfamily member 4 [Source:HGNC Sym...
729230	CCR2	C-C motif chemokine receptor 2 [Source:HG...
7293	TNFRSF4	TNF receptor superfamily member 4 [Source...
7422	VEGFA	vascular endothelial growth factor A [Sou...
7423	VEGFB	vascular endothelial growth factor B [Sou...
7424	VEGFC	vascular endothelial growth factor C [Sou...
7850	IL1R2	interleukin 1 receptor type 2 [Source:HGN...
7852	CXCR4	C-X-C motif chemokine receptor 4 [Source:...
80301	PLEKHO2	pleckstrin homology domain containing O2 ...
8200	GDF5	growth differentiation factor 5 [Source:H...
83729	INHBE	inhibin subunit beta E [Source:HGNC Symbo...
84957	RELT	RELT TNF receptor [Source:HGNC Symbol;Acc...
85480	TSLP	thymic stromal lymphopoietin [Source:HGNC...
8600	TNFSF11	TNF superfamily member 11 [Source:HGNC Sy...
8718	TNFRSF25	TNF receptor superfamily member 25 [Sourc...
8740	TNFSF14	TNF superfamily member 14 [Source:HGNC Sy...
8741	TNFSF13	TNF superfamily member 13 [Source:HGNC Sy...
8742	TNFSF12	TNF superfamily member 12 [Source:HGNC Sy...
8743	TNFSF10	TNF superfamily member 10 [Source:HGNC Sy...
8744	TNFSF9	TNF superfamily member 9 [Source:HGNC Sym...
8764	TNFRSF14	TNF receptor superfamily member 14 [Sourc...
8771	TNFRSF6B	TNF receptor superfamily member 6b [Sourc...
8784	TNFRSF18	TNF receptor superfamily member 18 [Sourc...
8792	TNFRSF11A	TNF receptor superfamily member 11a [Sour...
8793	TNFRSF10D	TNF receptor superfamily member 10d [Sour...
8794	TNFRSF10C	TNF receptor superfamily member 10c [Sour...
8795	TNFRSF10B	TNF receptor superfamily member 10b [Sour...
8797	TNFRSF10A	TNF receptor superfamily member 10a [Sour...
8807	IL18RAP	interleukin 18 receptor accessory protein...
8809	IL18R1	interleukin 18 receptor 1 [Source:HGNC Sy...
8995	TNFSF18	TNF superfamily member 18 [Source:HGNC Sy...
90	ACVR1	activin A receptor type 1 [Source:HGNC Sy...
91	ACVR1B	activin A receptor type 1B [Source:HGNC S...
9180	OSMR	oncostatin M receptor [Source:HGNC Symbol...
92	ACVR2A	activin A receptor type 2A [Source:HGNC S...
93	ACVR2B	activin A receptor type 2B [Source:HGNC S...
939	CD27	CD27 molecule [Source:HGNC Symbol;Acc:HGN...
94	ACVRL1	activin A receptor like type 1 [Source:HG...
943	TNFRSF8	TNF receptor superfamily member 8 [Source...
944	TNFSF8	TNF superfamily member 8 [Source:HGNC Sym...
9547	CXCL14	C-X-C motif chemokine ligand 14 [Source:H...
9560	CCL4L2	C-C motif chemokine ligand 4 like 2 [Sour...

958	CD40	CD40 molecule [Source:HGNC Symbol;Acc:HGN...
959	CD40LG	CD40 ligand [Source:HGNC Symbol;Acc:HGNC:...
970	CD70	CD70 molecule [Source:HGNC Symbol;Acc:HGN...
9966	TNFSF15	TNF superfamily member 15 [Source:HGNC Sy...

S3D - JAK-STAT-signaling

https://www.gsea-msigdb.org/gsea/msigdb/human/geneset/KEGG_JAK_STAT_SIGNALING_PATHWAY.html (155 genes)

NCBI (Entrez) Gene Id	Gene Symbol	Gene Description
10000	AKT3	AKT serine/threonine kinase 3 [Source:HGNC ...
10251	SPRY3	sprouty RTK signaling antagonist 3 [Source:...
10252	SPRY1	sprouty RTK signaling antagonist 1 [Source:...
10253	SPRY2	sprouty RTK signaling antagonist 2 [Source:...
10254	STAM2	signal transducing adaptor molecule 2 [Sour...
10379	IRF9	interferon regulatory factor 9 [Source:HGNC...
10401	PIAS3	protein inhibitor of activated STAT 3 [Sour...
11009	IL24	interleukin 24 [Source:HGNC Symbol;Acc:HGNC...
1154	CISH	cytokine inducible SH2 containing protein [...
116379	IL22RA2	interleukin 22 receptor subunit alpha 2 [So...
122809	SOCS4	suppressor of cytokine signaling 4 [Source:...
1270	CNTF	ciliary neurotrophic factor [Source:HGNC Sy...
1271	CNTFR	ciliary neurotrophic factor receptor [Sourc...
1387	CREBBP	CREB binding protein [Source:HGNC Symbol;Ac...
1437	CSF2	colony stimulating factor 2 [Source:HGNC Sy...
1438	CSF2RA	colony stimulating factor 2 receptor subuni...
1439	CSF2RB	colony stimulating factor 2 receptor subuni...
1440	CSF3	colony stimulating factor 3 [Source:HGNC Sy...
1441	CSF3R	colony stimulating factor 3 receptor [Sourc...
1442	CSH1	chorionic somatomammotropin hormone 1 [Sour...
1489	CTF1	cardiotrophin 1 [Source:HGNC Symbol;Acc:HGN...
149233	IL23R	interleukin 23 receptor [Source:HGNC Symbol...
161742	SPRED1	sprouty related EVH1 domain containing 1 [S...
163702	IFNLR1	interferon lambda receptor 1 [Source:HGNC S...
200734	SPRED2	sprouty related EVH1 domain containing 2 [S...
2033	EP300	E1A binding protein p300 [Source:HGNC Symbo...
2056	EPO	erythropoietin [Source:HGNC Symbol;Acc:HGNC...
2057	EPOR	erythropoietin receptor [Source:HGNC Symbol...
207	AKT1	AKT serine/threonine kinase 1 [Source:HGNC ...
208	AKT2	AKT serine/threonine kinase 2 [Source:HGNC ...
23529	CLCF1	cardiotrophin like cytokine factor 1 [Sourc...
23533	PIK3R5	phosphoinositide-3-kinase regulatory subuni...
23624	CBLC	Cbl proto-oncogene C [Source:HGNC Symbol;Ac...
2688	GH1	growth hormone 1 [Source:HGNC Symbol;Acc:HG...
2689	GH2	growth hormone 2 [Source:HGNC Symbol;Acc:HG...

2690	GHR	growth hormone receptor [Source:HGNC Symbol...
282616	IFNL2	interferon lambda 2 [Source:HGNC Symbol;Acc...
282617	IFNL3	interferon lambda 3 [Source:HGNC Symbol;Acc...
282618	IFNL1	interferon lambda 1 [Source:HGNC Symbol;Acc...
2885	GRB2	growth factor receptor bound protein 2 [Sou...
29949	IL19	interleukin 19 [Source:HGNC Symbol;Acc:HGNC...
30837	SOCS7	suppressor of cytokine signaling 7 [Source:...
338376	IFNE	interferon epsilon [Source:HGNC Symbol;Acc:...
3439	IFNA1	interferon alpha 1 [Source:HGNC Symbol;Acc:...
3440	IFNA2	interferon alpha 2 [Source:HGNC Symbol;Acc:...
3441	IFNA4	interferon alpha 4 [Source:HGNC Symbol;Acc:...
3442	IFNA5	interferon alpha 5 [Source:HGNC Symbol;Acc:...
3443	IFNA6	interferon alpha 6 [Source:HGNC Symbol;Acc:...
3444	IFNA7	interferon alpha 7 [Source:HGNC Symbol;Acc:...
3445	IFNA8	interferon alpha 8 [Source:HGNC Symbol;Acc:...
3446	IFNA10	interferon alpha 10 [Source:HGNC Symbol;Acc...
3447	IFNA13	interferon alpha 13 [Source:HGNC Symbol;Acc...
3448	IFNA14	interferon alpha 14 [Source:HGNC Symbol;Acc...
3449	IFNA16	interferon alpha 16 [Source:HGNC Symbol;Acc...
3451	IFNA17	interferon alpha 17 [Source:HGNC Symbol;Acc...
3452	IFNA21	interferon alpha 21 [Source:HGNC Symbol;Acc...
3454	IFNAR1	interferon alpha and beta receptor subunit ...
3455	IFNAR2	interferon alpha and beta receptor subunit ...
3456	IFNB1	interferon beta 1 [Source:HGNC Symbol;Acc:H...
3458	IFNG	interferon gamma [Source:HGNC Symbol;Acc:HG...
3459	IFNGR1	interferon gamma receptor 1 [Source:HGNC Sy...
3460	IFNGR2	interferon gamma receptor 2 [Source:HGNC Sy...
3467	IFNW1	interferon omega 1 [Source:HGNC Symbol;Acc:...
3558	IL2	interleukin 2 [Source:HGNC Symbol;Acc:HGNC:...
3559	IL2RA	interleukin 2 receptor subunit alpha [Sourc...
3560	IL2RB	interleukin 2 receptor subunit beta [Source...
3561	IL2RG	interleukin 2 receptor subunit gamma [Sourc...
3562	IL3	interleukin 3 [Source:HGNC Symbol;Acc:HGNC:...
3563	IL3RA	interleukin 3 receptor subunit alpha [Sourc...
3565	IL4	interleukin 4 [Source:HGNC Symbol;Acc:HGNC:...
3566	IL4R	interleukin 4 receptor [Source:HGNC Symbol;...
3567	IL5	interleukin 5 [Source:HGNC Symbol;Acc:HGNC:...
3568	IL5RA	interleukin 5 receptor subunit alpha [Sourc...
3569	IL6	interleukin 6 [Source:HGNC Symbol;Acc:HGNC:...
3570	IL6R	interleukin 6 receptor [Source:HGNC Symbol;...
3572	IL6ST	interleukin 6 cytokine family signal transd...
3574	IL7	interleukin 7 [Source:HGNC Symbol;Acc:HGNC:...
3575	IL7R	interleukin 7 receptor [Source:HGNC Symbol;...
3578	IL9	interleukin 9 [Source:HGNC Symbol;Acc:HGNC:...
3581	IL9R	interleukin 9 receptor [Source:HGNC Symbol;...
3586	IL10	interleukin 10 [Source:HGNC Symbol;Acc:HGNC...

3587	IL10RA	interleukin 10 receptor subunit alpha [Sour...
3588	IL10RB	interleukin 10 receptor subunit beta [Sourc...
3589	IL11	interleukin 11 [Source:HGNC Symbol;Acc:HGNC...
3590	IL11RA	interleukin 11 receptor subunit alpha [Sour...
3592	IL12A	interleukin 12A [Source:HGNC Symbol;Acc:HGN...
3593	IL12B	interleukin 12B [Source:HGNC Symbol;Acc:HGN...
3594	IL12RB1	interleukin 12 receptor subunit beta 1 [Sou...
3595	IL12RB2	interleukin 12 receptor subunit beta 2 [Sou...
3596	IL13	interleukin 13 [Source:HGNC Symbol;Acc:HGNC...
3597	IL13RA1	interleukin 13 receptor subunit alpha 1 [So...
3598	IL13RA2	interleukin 13 receptor subunit alpha 2 [So...
3600	IL15	interleukin 15 [Source:HGNC Symbol;Acc:HGNC...
3601	IL15RA	interleukin 15 receptor subunit alpha [Sour...
3716	JAK1	Janus kinase 1 [Source:HGNC Symbol;Acc:HGNC...
3717	JAK2	Janus kinase 2 [Source:HGNC Symbol;Acc:HGNC...
3718	JAK3	Janus kinase 3 [Source:HGNC Symbol;Acc:HGNC...
3952	LEP	leptin [Source:HGNC Symbol;Acc:HGNC:6553]
3953	LEPR	leptin receptor [Source:HGNC Symbol;Acc:HGN...
3976	LIF	LIF interleukin 6 family cytokine [Source:H...
3977	LIFR	LIF receptor subunit alpha [Source:HGNC Sym...
4352	MPL	MPL proto-oncogene, thrombopoietin receptor...
4609	MYC	MYC proto-oncogene, bHLH transcription fact...
5008	OSM	oncostatin M [Source:HGNC Symbol;Acc:HGNC:8...
50604	IL20	interleukin 20 [Source:HGNC Symbol;Acc:HGNC...
50615	IL21R	interleukin 21 receptor [Source:HGNC Symbol...
50616	IL22	interleukin 22 [Source:HGNC Symbol;Acc:HGNC...
51561	IL23A	interleukin 23 subunit alpha [Source:HGNC S...
51588	PIAS4	protein inhibitor of activated STAT 4 [Sour...
5290	PIK3CA	phosphatidylinositol-4,5-bisphosphate 3-kin...
5291	PIK3CB	phosphatidylinositol-4,5-bisphosphate 3-kin...
5292	PIM1	Pim-1 proto-oncogene, serine/threonine kina...
5293	PIK3CD	phosphatidylinositol-4,5-bisphosphate 3-kin...
5294	PIK3CG	phosphatidylinositol-4,5-bisphosphate 3-kin...
5295	PIK3R1	phosphoinositide-3-kinase regulatory subuni...
5296	PIK3R2	phosphoinositide-3-kinase regulatory subuni...
53832	IL20RA	interleukin 20 receptor subunit alpha [Sour...
53833	IL20RB	interleukin 20 receptor subunit beta [Sourc...
55801	IL26	interleukin 26 [Source:HGNC Symbol;Acc:HGNC...
5617	PRL	prolactin [Source:HGNC Symbol;Acc:HGNC:9445]
5618	PRLR	prolactin receptor [Source:HGNC Symbol;Acc:...
56832	IFNK	interferon kappa [Source:HGNC Symbol;Acc:HG...
5777	PTPN6	protein tyrosine phosphatase non-receptor t...
5781	PTPN11	protein tyrosine phosphatase non-receptor t...
58985	IL22RA1	interleukin 22 receptor subunit alpha 1 [So...
59067	IL21	interleukin 21 [Source:HGNC Symbol;Acc:HGNC...
595	CCND1	cyclin D1 [Source:HGNC Symbol;Acc:HGNC:1582]

598	BCL2L1	BCL2 like 1 [Source:HGNC Symbol;Acc:HGNC:992]
64109	CRLF2	cytokine receptor like factor 2 [Source:HGN...
6654	SOS1	SOS Ras/Rac guanine nucleotide exchange fac...
6655	SOS2	SOS Ras/Rho guanine nucleotide exchange fac...
6772	STAT1	signal transducer and activator of transcri...
6773	STAT2	signal transducer and activator of transcri...
6774	STAT3	signal transducer and activator of transcri...
6775	STAT4	signal transducer and activator of transcri...
6776	STAT5A	signal transducer and activator of transcri...
6777	STAT5B	signal transducer and activator of transcri...
6778	STAT6	signal transducer and activator of transcri...
7173	TPO	thyroid peroxidase [Source:HGNC Symbol;Acc:...
7297	TYK2	tyrosine kinase 2 [Source:HGNC Symbol;Acc:H...
8027	STAM	signal transducing adaptor molecule [Source...
81848	SPRY4	sprouty RTK signaling antagonist 4 [Source:...
8503	PIK3R3	phosphoinositide-3-kinase regulatory subuni...
85480	TSLP	thymic stromal lymphopoietin [Source:HGNC S...
8554	PIAS1	protein inhibitor of activated STAT 1 [Sour...
8651	SOCS1	suppressor of cytokine signaling 1 [Source:...
867	CBL	Cbl proto-oncogene [Source:HGNC Symbol;Acc:...
868	CBLB	Cbl proto-oncogene B [Source:HGNC Symbol;Ac...
8835	SOCS2	suppressor of cytokine signaling 2 [Source:...
894	CCND2	cyclin D2 [Source:HGNC Symbol;Acc:HGNC:1583]
896	CCND3	cyclin D3 [Source:HGNC Symbol;Acc:HGNC:1585]
9021	SOCS3	suppressor of cytokine signaling 3 [Source:...
9063	PIAS2	protein inhibitor of activated STAT 2 [Sour...
9180	OSMR	oncostatin M receptor [Source:HGNC Symbol;A...
9655	SOCS5	suppressor of cytokine signaling 5 [Source:...

S3E - Focal adhesion
https://www.gsea-msigdb.org/gsea/msigdb/human/geneset/KEGG_FOCAL_ADHESION.html
(199 genes)

**NCBI (Entrez) Gene Id**	**Gene Symbol**	**Gene Description**
10000	AKT3	AKT serine/threonine kinase 3 [Source:HGNC...
10298	PAK4	p21 (RAC1) activated kinase 4 [Source:HGNC...
10319	LAMC3	laminin subunit gamma 3 [Source:HGNC Symbo...
103910	MYL12B	myosin light chain 12B [Source:HGNC Symbol...
10398	MYL9	myosin light chain 9 [Source:HGNC Symbol;A...
10451	VAV3	vav guanine nucleotide exchange factor 3 [...
10627	MYL12A	myosin light chain 12A [Source:HGNC Symbol...
1101	CHAD	chondroadherin [Source:HGNC Symbol;Acc:HGN...
1277	COL1A1	collagen type I alpha 1 chain [Source:HGNC...
1278	COL1A2	collagen type I alpha 2 chain [Source:HGNC...

1280	COL2A1	collagen type II alpha 1 chain [Source:HGN...
1281	COL3A1	collagen type III alpha 1 chain [Source:HG...
1282	COL4A1	collagen type IV alpha 1 chain [Source:HGN...
1284	COL4A2	collagen type IV alpha 2 chain [Source:HGN...
1286	COL4A4	collagen type IV alpha 4 chain [Source:HGN...
1288	COL4A6	collagen type IV alpha 6 chain [Source:HGN...
1289	COL5A1	collagen type V alpha 1 chain [Source:HGNC...
1290	COL5A2	collagen type V alpha 2 chain [Source:HGNC...
1291	COL6A1	collagen type VI alpha 1 chain [Source:HGN...
1292	COL6A2	collagen type VI alpha 2 chain [Source:HGN...
1293	COL6A3	collagen type VI alpha 3 chain [Source:HGN...
1301	COL11A1	collagen type XI alpha 1 chain [Source:HGN...
1302	COL11A2	collagen type XI alpha 2 chain [Source:HGN...
1311	COMP	cartilage oligomeric matrix protein [Sourc...
131873	COL6A6	collagen type VI alpha 6 chain [Source:HGN...
1398	CRK	CRK proto-oncogene, adaptor protein [Sourc...
1399	CRKL	CRK like proto-oncogene, adaptor protein [...
1499	CTNNB1	catenin beta 1 [Source:HGNC Symbol;Acc:HGN...
1729	DIAPH1	diaphanous related formin 1 [Source:HGNC S...
1793	DOCK1	dedicator of cytokinesis 1 [Source:HGNC Sy...
1950	EGF	epidermal growth factor [Source:HGNC Symbo...
1956	EGFR	epidermal growth factor receptor [Source:H...
2002	ELK1	ETS transcription factor ELK1 [Source:HGNC...
2064	ERBB2	erb-b2 receptor tyrosine kinase 2 [Source:...
207	AKT1	AKT serine/threonine kinase 1 [Source:HGNC...
208	AKT2	AKT serine/threonine kinase 2 [Source:HGNC...
2277	VEGFD	vascular endothelial growth factor D [Sour...
22798	LAMB4	laminin subunit beta 4 [Source:HGNC Symbol...
22801	ITGA11	integrin subunit alpha 11 [Source:HGNC Sym...
2316	FLNA	filamin A [Source:HGNC Symbol;Acc:HGNC:3754]
2317	FLNB	filamin B [Source:HGNC Symbol;Acc:HGNC:3755]
2318	FLNC	filamin C [Source:HGNC Symbol;Acc:HGNC:3756]
2321	FLT1	fms related receptor tyrosine kinase 1 [So...
2324	FLT4	fms related receptor tyrosine kinase 4 [So...
2335	FN1	fibronectin 1 [Source:HGNC Symbol;Acc:HGNC...
23396	PIP5K1C	phosphatidylinositol-4-phosphate 5-kinase ...
23533	PIK3R5	phosphoinositide-3-kinase regulatory subun...
2534	FYN	FYN proto-oncogene, Src family tyrosine ki...
25759	SHC2	SHC adaptor protein 2 [Source:HGNC Symbol;...
284217	LAMA1	laminin subunit alpha 1 [Source:HGNC Symbo...
2885	GRB2	growth factor receptor bound protein 2 [So...
2889	RAPGEF1	Rap guanine nucleotide exchange factor 1 [...
2909	ARHGAP35	Rho GTPase activating protein 35 [Source:H...
2932	GSK3B	glycogen synthase kinase 3 beta [Source:HG...
29780	PARVB	parvin beta [Source:HGNC Symbol;Acc:HGNC:1...
29895	MYLPF	myosin light chain, phosphorylatable, fast...

3082	HGF	hepatocyte growth factor [Source:HGNC Symb...
3265	HRAS	HRas proto-oncogene, GTPase [Source:HGNC S...
329	BIRC2	baculoviral IAP repeat containing 2 [Sourc...
330	BIRC3	baculoviral IAP repeat containing 3 [Sourc...
331	XIAP	X-linked inhibitor of apoptosis [Source:HG...
3371	TNC	tenascin C [Source:HGNC Symbol;Acc:HGNC:5318]
3381	IBSP	integrin binding sialoprotein [Source:HGNC...
3479	IGF1	insulin like growth factor 1 [Source:HGNC ...
3480	IGF1R	insulin like growth factor 1 receptor [Sou...
3611	ILK	integrin linked kinase [Source:HGNC Symbol...
3655	ITGA6	integrin subunit alpha 6 [Source:HGNC Symb...
3672	ITGA1	integrin subunit alpha 1 [Source:HGNC Symb...
3673	ITGA2	integrin subunit alpha 2 [Source:HGNC Symb...
3674	ITGA2B	integrin subunit alpha 2b [Source:HGNC Sym...
3675	ITGA3	integrin subunit alpha 3 [Source:HGNC Symb...
3676	ITGA4	integrin subunit alpha 4 [Source:HGNC Symb...
3678	ITGA5	integrin subunit alpha 5 [Source:HGNC Symb...
3679	ITGA7	integrin subunit alpha 7 [Source:HGNC Symb...
3680	ITGA9	integrin subunit alpha 9 [Source:HGNC Symb...
3685	ITGAV	integrin subunit alpha V [Source:HGNC Symb...
3688	ITGB1	integrin subunit beta 1 [Source:HGNC Symbo...
3690	ITGB3	integrin subunit beta 3 [Source:HGNC Symbo...
3691	ITGB4	integrin subunit beta 4 [Source:HGNC Symbo...
3693	ITGB5	integrin subunit beta 5 [Source:HGNC Symbo...
3694	ITGB6	integrin subunit beta 6 [Source:HGNC Symbo...
3695	ITGB7	integrin subunit beta 7 [Source:HGNC Symbo...
3696	ITGB8	integrin subunit beta 8 [Source:HGNC Symbo...
3725	JUN	Jun proto-oncogene, AP-1 transcription fac...
3791	KDR	kinase insert domain receptor [Source:HGNC...
387	RHOA	ras homolog family member A [Source:HGNC S...
3908	LAMA2	laminin subunit alpha 2 [Source:HGNC Symbo...
3909	LAMA3	laminin subunit alpha 3 [Source:HGNC Symbo...
3910	LAMA4	laminin subunit alpha 4 [Source:HGNC Symbo...
3911	LAMA5	laminin subunit alpha 5 [Source:HGNC Symbo...
3912	LAMB1	laminin subunit beta 1 [Source:HGNC Symbol...
3913	LAMB2	laminin subunit beta 2 [Source:HGNC Symbol...
3914	LAMB3	laminin subunit beta 3 [Source:HGNC Symbol...
3915	LAMC1	laminin subunit gamma 1 [Source:HGNC Symbo...
3918	LAMC2	laminin subunit gamma 2 [Source:HGNC Symbo...
394	ARHGAP5	Rho GTPase activating protein 5 [Source:HG...
399694	SHC4	SHC adaptor protein 4 [Source:HGNC Symbol;...
4233	MET	MET proto-oncogene, receptor tyrosine kina...
4633	MYL2	myosin light chain 2 [Source:HGNC Symbol;A...
4636	MYL5	myosin light chain 5 [Source:HGNC Symbol;A...
4638	MYLK	myosin light chain kinase [Source:HGNC Sym...
4659	PPP1R12A	protein phosphatase 1 regulatory subunit 1...

50509	COL5A3	collagen type V alpha 3 chain [Source:HGNC...
5058	PAK1	p21 (RAC1) activated kinase 1 [Source:HGNC...
5062	PAK2	p21 (RAC1) activated kinase 2 [Source:HGNC...
5063	PAK3	p21 (RAC1) activated kinase 3 [Source:HGNC...
5154	PDGFA	platelet derived growth factor subunit A [...
5155	PDGFB	platelet derived growth factor subunit B [...
5156	PDGFRA	platelet derived growth factor receptor al...
5159	PDGFRB	platelet derived growth factor receptor be...
5170	PDPK1	3-phosphoinositide dependent protein kinas...
5228	PGF	placental growth factor [Source:HGNC Symbo...
5290	PIK3CA	phosphatidylinositol-4,5-bisphosphate 3-ki...
5291	PIK3CB	phosphatidylinositol-4,5-bisphosphate 3-ki...
5293	PIK3CD	phosphatidylinositol-4,5-bisphosphate 3-ki...
5294	PIK3CG	phosphatidylinositol-4,5-bisphosphate 3-ki...
5295	PIK3R1	phosphoinositide-3-kinase regulatory subun...
5296	PIK3R2	phosphoinositide-3-kinase regulatory subun...
53358	SHC3	SHC adaptor protein 3 [Source:HGNC Symbol;...
5499	PPP1CA	protein phosphatase 1 catalytic subunit al...
5500	PPP1CB	protein phosphatase 1 catalytic subunit be...
5501	PPP1CC	protein phosphatase 1 catalytic subunit ga...
55742	PARVA	parvin alpha [Source:HGNC Symbol;Acc:HGNC:...
5578	PRKCA	protein kinase C alpha [Source:HGNC Symbol...
5579	PRKCB	protein kinase C beta [Source:HGNC Symbol;...
5582	PRKCG	protein kinase C gamma [Source:HGNC Symbol...
5594	MAPK1	mitogen-activated protein kinase 1 [Source...
5595	MAPK3	mitogen-activated protein kinase 3 [Source...
5599	MAPK8	mitogen-activated protein kinase 8 [Source...
5601	MAPK9	mitogen-activated protein kinase 9 [Source...
5602	MAPK10	mitogen-activated protein kinase 10 [Sourc...
56034	PDGFC	platelet derived growth factor C [Source:H...
5604	MAP2K1	mitogen-activated protein kinase kinase 1 ...
5649	RELN	reelin [Source:HGNC Symbol;Acc:HGNC:9957]
56924	PAK6	p21 (RAC1) activated kinase 6 [Source:HGNC...
57144	PAK5	p21 (RAC1) activated kinase 5 [Source:HGNC...
572	BAD	BCL2 associated agonist of cell death [Sou...
5728	PTEN	phosphatase and tensin homolog [Source:HGN...
5747	PTK2	protein tyrosine kinase 2 [Source:HGNC Sym...
5829	PXN	paxillin [Source:HGNC Symbol;Acc:HGNC:9718]
58498	MYL7	myosin light chain 7 [Source:HGNC Symbol;A...
5879	RAC1	Rac family small GTPase 1 [Source:HGNC Sym...
5880	RAC2	Rac family small GTPase 2 [Source:HGNC Sym...
5881	RAC3	Rac family small GTPase 3 [Source:HGNC Sym...
5894	RAF1	Raf-1 proto-oncogene, serine/threonine kin...
5906	RAP1A	RAP1A, member of RAS oncogene family [Sour...
5908	RAP1B	RAP1B, member of RAS oncogene family [Sour...
5923	RASGRF1	Ras protein specific guanine nucleotide re...

595	CCND1	cyclin D1 [Source:HGNC Symbol;Acc:HGNC:1582]
596	BCL2	BCL2 apoptosis regulator [Source:HGNC Symb...
60	ACTB	actin beta [Source:HGNC Symbol;Acc:HGNC:132]
6093	ROCK1	Rho associated coiled-coil containing prot...
63923	TNN	tenascin N [Source:HGNC Symbol;Acc:HGNC:22...
64098	PARVG	parvin gamma [Source:HGNC Symbol;Acc:HGNC:...
6464	SHC1	SHC adaptor protein 1 [Source:HGNC Symbol;...
6654	SOS1	SOS Ras/Rac guanine nucleotide exchange fa...
6655	SOS2	SOS Ras/Rho guanine nucleotide exchange fa...
6696	SPP1	secreted phosphoprotein 1 [Source:HGNC Sym...
6714	SRC	SRC proto-oncogene, non-receptor tyrosine ...
673	BRAF	B-Raf proto-oncogene, serine/threonine kin...
7057	THBS1	thrombospondin 1 [Source:HGNC Symbol;Acc:H...
7058	THBS2	thrombospondin 2 [Source:HGNC Symbol;Acc:H...
7059	THBS3	thrombospondin 3 [Source:HGNC Symbol;Acc:H...
7060	THBS4	thrombospondin 4 [Source:HGNC Symbol;Acc:H...
7094	TLN1	talin 1 [Source:HGNC Symbol;Acc:HGNC:11845]
71	ACTG1	actin gamma 1 [Source:HGNC Symbol;Acc:HGNC...
7143	TNR	tenascin R [Source:HGNC Symbol;Acc:HGNC:11...
7148	TNXB	tenascin XB [Source:HGNC Symbol;Acc:HGNC:1...
7408	VASP	vasodilator stimulated phosphoprotein [Sou...
7409	VAV1	vav guanine nucleotide exchange factor 1 [...
7410	VAV2	vav guanine nucleotide exchange factor 2 [...
7414	VCL	vinculin [Source:HGNC Symbol;Acc:HGNC:12665]
7422	VEGFA	vascular endothelial growth factor A [Sour...
7423	VEGFB	vascular endothelial growth factor B [Sour...
7424	VEGFC	vascular endothelial growth factor C [Sour...
7448	VTN	vitronectin [Source:HGNC Symbol;Acc:HGNC:1...
7450	VWF	von Willebrand factor [Source:HGNC Symbol;...
7791	ZYX	zyxin [Source:HGNC Symbol;Acc:HGNC:13200]
80310	PDGFD	platelet derived growth factor D [Source:H...
81	ACTN4	actinin alpha 4 [Source:HGNC Symbol;Acc:HG...
824	CAPN2	calpain 2 [Source:HGNC Symbol;Acc:HGNC:1479]
83660	TLN2	talin 2 [Source:HGNC Symbol;Acc:HGNC:15447]
8503	PIK3R3	phosphoinositide-3-kinase regulatory subun...
8515	ITGA10	integrin subunit alpha 10 [Source:HGNC Sym...
8516	ITGA8	integrin subunit alpha 8 [Source:HGNC Symb...
85366	MYLK2	myosin light chain kinase 2 [Source:HGNC S...
857	CAV1	caveolin 1 [Source:HGNC Symbol;Acc:HGNC:1527]
858	CAV2	caveolin 2 [Source:HGNC Symbol;Acc:HGNC:1528]
859	CAV3	caveolin 3 [Source:HGNC Symbol;Acc:HGNC:1529]
87	ACTN1	actinin alpha 1 [Source:HGNC Symbol;Acc:HG...
88	ACTN2	actinin alpha 2 [Source:HGNC Symbol;Acc:HG...
89	ACTN3	actinin alpha 3 [Source:HGNC Symbol;Acc:HG...
894	CCND2	cyclin D2 [Source:HGNC Symbol;Acc:HGNC:1583]
896	CCND3	cyclin D3 [Source:HGNC Symbol;Acc:HGNC:1585]

91807	MYLK3	myosin light chain kinase 3 [Source:HGNC S...
93408	MYL10	myosin light chain 10 [Source:HGNC Symbol;...
9475	ROCK2	Rho associated coiled-coil containing prot...
9564	BCAR1	BCAR1 scaffold protein, Cas family member ...
998	CDC42	cell division cycle 42 [Source:HGNC Symbol...

S3F - GAP junction
https://www.gsea-msigdb.org/gsea/msigdb/human/geneset/KEGG_GAP_JUNCTION.html
(90 genes)

NCBI (Entrez) Gene Id	Gene Symbol	Gene Description
10376	TUBA1B	tubulin alpha 1b [Source:HGNC Symbol;Acc:HG...
10381	TUBB3	tubulin beta 3 class III [Source:HGNC Symbo...
10382	TUBB4A	tubulin beta 4A class IVa [Source:HGNC Symb...
10383	TUBB4B	tubulin beta 4B class IVb [Source:HGNC Symb...
107	ADCY1	adenylate cyclase 1 [Source:HGNC Symbol;Acc...
10746	MAP3K2	mitogen-activated protein kinase kinase kin...
108	ADCY2	adenylate cyclase 2 [Source:HGNC Symbol;Acc...
109	ADCY3	adenylate cyclase 3 [Source:HGNC Symbol;Acc...
111	ADCY5	adenylate cyclase 5 [Source:HGNC Symbol;Acc...
112	ADCY6	adenylate cyclase 6 [Source:HGNC Symbol;Acc...
112714	TUBA3E	tubulin alpha 3e [Source:HGNC Symbol;Acc:HG...
113	ADCY7	adenylate cyclase 7 [Source:HGNC Symbol;Acc...
113457	TUBA3D	tubulin alpha 3d [Source:HGNC Symbol;Acc:HG...
114	ADCY8	adenylate cyclase 8 [Source:HGNC Symbol;Acc...
115	ADCY9	adenylate cyclase 9 [Source:HGNC Symbol;Acc...
1453	CSNK1D	casein kinase 1 delta [Source:HGNC Symbol;A...
153	ADRB1	adrenoceptor beta 1 [Source:HGNC Symbol;Acc...
1812	DRD1	dopamine receptor D1 [Source:HGNC Symbol;Ac...
1813	DRD2	dopamine receptor D2 [Source:HGNC Symbol;Ac...
1902	LPAR1	lysophosphatidic acid receptor 1 [Source:HG...
1950	EGF	epidermal growth factor [Source:HGNC Symbol...
1956	EGFR	epidermal growth factor receptor [Source:HG...
196883	ADCY4	adenylate cyclase 4 [Source:HGNC Symbol;Acc...
203068	TUBB	tubulin beta class I [Source:HGNC Symbol;Ac...
23236	PLCB1	phospholipase C beta 1 [Source:HGNC Symbol;...
2697	GJA1	gap junction protein alpha 1 [Source:HGNC S...
2767	GNA11	G protein subunit alpha 11 [Source:HGNC Sym...
2770	GNAI1	G protein subunit alpha i1 [Source:HGNC Sym...
2771	GNAI2	G protein subunit alpha i2 [Source:HGNC Sym...
2773	GNAI3	G protein subunit alpha i3 [Source:HGNC Sym...
2776	GNAQ	G protein subunit alpha q [Source:HGNC Symb...
2778	GNAS	GNAS complex locus [Source:HGNC Symbol;Acc:...
2885	GRB2	growth factor receptor bound protein 2 [Sou...
2911	GRM1	glutamate metabotropic receptor 1 [Source:H...

2915	GRM5	glutamate metabotropic receptor 5 [Source:H...
2977	GUCY1A2	guanylate cyclase 1 soluble subunit alpha 2...
2982	GUCY1A1	guanylate cyclase 1 soluble subunit alpha 1...
2983	GUCY1B1	guanylate cyclase 1 soluble subunit beta 1 ...
3265	HRAS	HRas proto-oncogene, GTPase [Source:HGNC Sy...
3356	HTR2A	5-hydroxytryptamine receptor 2A [Source:HGN...
3357	HTR2B	5-hydroxytryptamine receptor 2B [Source:HGN...
3358	HTR2C	5-hydroxytryptamine receptor 2C [Source:HGN...
347688	TUBB8	tubulin beta 8 class VIII [Source:HGNC Symb...
347733	TUBB2B	tubulin beta 2B class IIb [Source:HGNC Symb...
3708	ITPR1	inositol 1,4,5-trisphosphate receptor type ...
3709	ITPR2	inositol 1,4,5-trisphosphate receptor type ...
3710	ITPR3	inositol 1,4,5-trisphosphate receptor type ...
3845	KRAS	KRAS proto-oncogene, GTPase [Source:HGNC Sy...
4893	NRAS	NRAS proto-oncogene, GTPase [Source:HGNC Sy...
5154	PDGFA	platelet derived growth factor subunit A [S...
5155	PDGFB	platelet derived growth factor subunit B [S...
5156	PDGFRA	platelet derived growth factor receptor alp...
5159	PDGFRB	platelet derived growth factor receptor bet...
51807	TUBA8	tubulin alpha 8 [Source:HGNC Symbol;Acc:HGN...
5330	PLCB2	phospholipase C beta 2 [Source:HGNC Symbol;...
5331	PLCB3	phospholipase C beta 3 [Source:HGNC Symbol;...
5332	PLCB4	phospholipase C beta 4 [Source:HGNC Symbol;...
5566	PRKACA	protein kinase cAMP-activated catalytic sub...
5567	PRKACB	protein kinase cAMP-activated catalytic sub...
5568	PRKACG	protein kinase cAMP-activated catalytic sub...
5578	PRKCA	protein kinase C alpha [Source:HGNC Symbol;...
5579	PRKCB	protein kinase C beta [Source:HGNC Symbol;A...
5582	PRKCG	protein kinase C gamma [Source:HGNC Symbol;...
5592	PRKG1	protein kinase cGMP-dependent 1 [Source:HGN...
5593	PRKG2	protein kinase cGMP-dependent 2 [Source:HGN...
5594	MAPK1	mitogen-activated protein kinase 1 [Source:...
5595	MAPK3	mitogen-activated protein kinase 3 [Source:...
5598	MAPK7	mitogen-activated protein kinase 7 [Source:...
56034	PDGFC	platelet derived growth factor C [Source:HG...
5604	MAP2K1	mitogen-activated protein kinase kinase 1 [...
5605	MAP2K2	mitogen-activated protein kinase kinase 2 [...
5607	MAP2K5	mitogen-activated protein kinase kinase 5 [...
5613	PRKX	protein kinase X-linked [Source:HGNC Symbol...
56604	TUBB7P	tubulin beta 7 pseudogene [Source:HGNC Symb...
57369	GJD2	gap junction protein delta 2 [Source:HGNC S...
5894	RAF1	Raf-1 proto-oncogene, serine/threonine kina...
6654	SOS1	SOS Ras/Rac guanine nucleotide exchange fac...
6655	SOS2	SOS Ras/Rho guanine nucleotide exchange fac...
6714	SRC	SRC proto-oncogene, non-receptor tyrosine k...
7082	TJP1	tight junction protein 1 [Source:HGNC Symbo...

7277	TUBA4A	tubulin alpha 4a [Source:HGNC Symbol;Acc:HG...
7278	TUBA3C	tubulin alpha 3c [Source:HGNC Symbol;Acc:HG...
7280	TUBB2A	tubulin beta 2A class IIa [Source:HGNC Symb...
7846	TUBA1A	tubulin alpha 1a [Source:HGNC Symbol;Acc:HG...
79861	TUBAL3	tubulin alpha like 3 [Source:HGNC Symbol;Ac...
80310	PDGFD	platelet derived growth factor D [Source:HG...
81027	TUBB1	tubulin beta 1 class VI [Source:HGNC Symbol...
84617	TUBB6	tubulin beta 6 class V [Source:HGNC Symbol;...
84790	TUBA1C	tubulin alpha 1c [Source:HGNC Symbol;Acc:HG...
983	CDK1	cyclin dependent kinase 1 [Source:HGNC Symb...

S3G - Extracellular matrix receptor interaction
https://www.gsea-msigdb.org/gsea/msigdb/human/geneset/KEGG_ECM_RECEPTOR_INTERACTION.html
(84 genes)

NCBI (Entrez) Gene Id	Gene Symbol	Gene Description
10319	LAMC3	laminin subunit gamma 3 [Source:HGNC Symbol...
1101	CHAD	chondroadherin [Source:HGNC Symbol;Acc:HGNC...
1277	COL1A1	collagen type I alpha 1 chain [Source:HGNC ...
1278	COL1A2	collagen type I alpha 2 chain [Source:HGNC ...
1280	COL2A1	collagen type II alpha 1 chain [Source:HGNC...
1281	COL3A1	collagen type III alpha 1 chain [Source:HGN...
1282	COL4A1	collagen type IV alpha 1 chain [Source:HGNC...
1284	COL4A2	collagen type IV alpha 2 chain [Source:HGNC...
1286	COL4A4	collagen type IV alpha 4 chain [Source:HGNC...
1288	COL4A6	collagen type IV alpha 6 chain [Source:HGNC...
1289	COL5A1	collagen type V alpha 1 chain [Source:HGNC ...
1290	COL5A2	collagen type V alpha 2 chain [Source:HGNC ...
1291	COL6A1	collagen type VI alpha 1 chain [Source:HGNC...
1292	COL6A2	collagen type VI alpha 2 chain [Source:HGNC...
1293	COL6A3	collagen type VI alpha 3 chain [Source:HGNC...
1301	COL11A1	collagen type XI alpha 1 chain [Source:HGNC...
1302	COL11A2	collagen type XI alpha 2 chain [Source:HGNC...
1311	COMP	cartilage oligomeric matrix protein [Source...
131873	COL6A6	collagen type VI alpha 6 chain [Source:HGNC...
1605	DAG1	dystroglycan 1 [Source:HGNC Symbol;Acc:HGNC...
22798	LAMB4	laminin subunit beta 4 [Source:HGNC Symbol;...
22801	ITGA11	integrin subunit alpha 11 [Source:HGNC Symb...
22987	SV2C	synaptic vesicle glycoprotein 2C [Source:HG...
2335	FN1	fibronectin 1 [Source:HGNC Symbol;Acc:HGNC:...
2811	GP1BA	glycoprotein Ib platelet subunit alpha [Sou...
2812	GP1BB	glycoprotein Ib platelet subunit beta [Sour...
2814	GP5	glycoprotein V platelet [Source:HGNC Symbol...
2815	GP9	glycoprotein IX platelet [Source:HGNC Symbo...

284217	LAMA1	laminin subunit alpha 1 [Source:HGNC Symbol...
3161	HMMR	hyaluronan mediated motility receptor [Sour...
3339	HSPG2	heparan sulfate proteoglycan 2 [Source:HGNC...
3371	TNC	tenascin C [Source:HGNC Symbol;Acc:HGNC:5318]
3381	IBSP	integrin binding sialoprotein [Source:HGNC ...
3655	ITGA6	integrin subunit alpha 6 [Source:HGNC Symbo...
3672	ITGA1	integrin subunit alpha 1 [Source:HGNC Symbo...
3673	ITGA2	integrin subunit alpha 2 [Source:HGNC Symbo...
3674	ITGA2B	integrin subunit alpha 2b [Source:HGNC Symb...
3675	ITGA3	integrin subunit alpha 3 [Source:HGNC Symbo...
3676	ITGA4	integrin subunit alpha 4 [Source:HGNC Symbo...
3678	ITGA5	integrin subunit alpha 5 [Source:HGNC Symbo...
3679	ITGA7	integrin subunit alpha 7 [Source:HGNC Symbo...
3680	ITGA9	integrin subunit alpha 9 [Source:HGNC Symbo...
3685	ITGAV	integrin subunit alpha V [Source:HGNC Symbo...
3688	ITGB1	integrin subunit beta 1 [Source:HGNC Symbol...
3690	ITGB3	integrin subunit beta 3 [Source:HGNC Symbol...
3691	ITGB4	integrin subunit beta 4 [Source:HGNC Symbol...
3693	ITGB5	integrin subunit beta 5 [Source:HGNC Symbol...
3694	ITGB6	integrin subunit beta 6 [Source:HGNC Symbol...
3695	ITGB7	integrin subunit beta 7 [Source:HGNC Symbol...
3696	ITGB8	integrin subunit beta 8 [Source:HGNC Symbol...
375790	AGRN	agrin [Source:HGNC Symbol;Acc:HGNC:329]
3908	LAMA2	laminin subunit alpha 2 [Source:HGNC Symbol...
3909	LAMA3	laminin subunit alpha 3 [Source:HGNC Symbol...
3910	LAMA4	laminin subunit alpha 4 [Source:HGNC Symbol...
3911	LAMA5	laminin subunit alpha 5 [Source:HGNC Symbol...
3912	LAMB1	laminin subunit beta 1 [Source:HGNC Symbol;...
3913	LAMB2	laminin subunit beta 2 [Source:HGNC Symbol;...
3914	LAMB3	laminin subunit beta 3 [Source:HGNC Symbol;...
3915	LAMC1	laminin subunit gamma 1 [Source:HGNC Symbol...
3918	LAMC2	laminin subunit gamma 2 [Source:HGNC Symbol...
50509	COL5A3	collagen type V alpha 3 chain [Source:HGNC ...
51206	GP6	glycoprotein VI platelet [Source:HGNC Symbo...
5649	RELN	reelin [Source:HGNC Symbol;Acc:HGNC:9957]
6382	SDC1	syndecan 1 [Source:HGNC Symbol;Acc:HGNC:10658]
6383	SDC2	syndecan 2 [Source:HGNC Symbol;Acc:HGNC:10659]
6385	SDC4	syndecan 4 [Source:HGNC Symbol;Acc:HGNC:10661]
63923	TNN	tenascin N [Source:HGNC Symbol;Acc:HGNC:22942]
6696	SPP1	secreted phosphoprotein 1 [Source:HGNC Symb...
7057	THBS1	thrombospondin 1 [Source:HGNC Symbol;Acc:HG...
7058	THBS2	thrombospondin 2 [Source:HGNC Symbol;Acc:HG...
7059	THBS3	thrombospondin 3 [Source:HGNC Symbol;Acc:HG...
7060	THBS4	thrombospondin 4 [Source:HGNC Symbol;Acc:HG...
7143	TNR	tenascin R [Source:HGNC Symbol;Acc:HGNC:11953]
7148	TNXB	tenascin XB [Source:HGNC Symbol;Acc:HGNC:11...

7448	VTN	vitronectin [Source:HGNC Symbol;Acc:HGNC:12...
7450	VWF	von Willebrand factor [Source:HGNC Symbol;A...
8515	ITGA10	integrin subunit alpha 10 [Source:HGNC Symb...
8516	ITGA8	integrin subunit alpha 8 [Source:HGNC Symbo...
948	CD36	CD36 molecule [Source:HGNC Symbol;Acc:HGNC:...
960	CD44	CD44 molecule (Indian blood group) [Source:...
961	CD47	CD47 molecule [Source:HGNC Symbol;Acc:HGNC:...
9672	SDC3	syndecan 3 [Source:HGNC Symbol;Acc:HGNC:10660]
9899	SV2B	synaptic vesicle glycoprotein 2B [Source:HG...
9900	SV2A	synaptic vesicle glycoprotein 2A [Source:HG...

S3H - Regulation of actin cytoskeleton
https://www.gsea-msigdb.org/gsea/msigdb/human/geneset/KEGG_REGULATION_OF_ACTIN_CYTOSKELETON.html
(213 genes)

NCBI (Entrez) Gene Id	Gene Symbol	Gene Description
10092	ARPC5	actin related protein 2/3 complex subunit ...
10093	ARPC4	actin related protein 2/3 complex subunit ...
10094	ARPC3	actin related protein 2/3 complex subunit ...
10095	ARPC1B	actin related protein 2/3 complex subunit ...
10109	ARPC2	actin related protein 2/3 complex subunit ...
10152	ABI2	abl interactor 2 [Source:HGNC Symbol;Acc:H...
10163	WASF2	WASP family member 2 [Source:HGNC Symbol;A...
10297	APC2	APC regulator of WNT signaling pathway 2 [...
10298	PAK4	p21 (RAC1) activated kinase 4 [Source:HGNC...
103910	MYL12B	myosin light chain 12B [Source:HGNC Symbol...
10398	MYL9	myosin light chain 9 [Source:HGNC Symbol;A...
10451	VAV3	vav guanine nucleotide exchange factor 3 [...
10458	BAIAP2	BAR/IMD domain containing adaptor protein ...
10552	ARPC1A	actin related protein 2/3 complex subunit ...
10627	MYL12A	myosin light chain 12A [Source:HGNC Symbol...
10672	GNA13	G protein subunit alpha 13 [Source:HGNC Sy...
1072	CFL1	cofilin 1 [Source:HGNC Symbol;Acc:HGNC:1874]
1073	CFL2	cofilin 2 [Source:HGNC Symbol;Acc:HGNC:1875]
10787	NCKAP1	NCK associated protein 1 [Source:HGNC Symb...
10788	IQGAP2	IQ motif containing GTPase activating prot...
1128	CHRM1	cholinergic receptor muscarinic 1 [Source:...
1129	CHRM2	cholinergic receptor muscarinic 2 [Source:...
1131	CHRM3	cholinergic receptor muscarinic 3 [Source:...
1132	CHRM4	cholinergic receptor muscarinic 4 [Source:...
1133	CHRM5	cholinergic receptor muscarinic 5 [Source:...
128239	IQGAP3	IQ motif containing GTPase activating prot...
1398	CRK	CRK proto-oncogene, adaptor protein [Sourc...
1399	CRKL	CRK like proto-oncogene, adaptor protein [...

1445	CSK	C-terminal Src kinase [Source:HGNC Symbol;...
1729	DIAPH1	diaphanous related formin 1 [Source:HGNC S...
1730	DIAPH2	diaphanous related formin 2 [Source:HGNC S...
1793	DOCK1	dedicator of cytokinesis 1 [Source:HGNC Sy...
1950	EGF	epidermal growth factor [Source:HGNC Symbo...
1956	EGFR	epidermal growth factor receptor [Source:H...
200576	PIKFYVE	phosphoinositide kinase, FYVE-type zinc fi...
2147	F2	coagulation factor II, thrombin [Source:HG...
2149	F2R	coagulation factor II thrombin receptor [S...
2245	FGD1	FYVE, RhoGEF and PH domain containing 1 [S...
2246	FGF1	fibroblast growth factor 1 [Source:HGNC Sy...
2247	FGF2	fibroblast growth factor 2 [Source:HGNC Sy...
2248	FGF3	fibroblast growth factor 3 [Source:HGNC Sy...
2249	FGF4	fibroblast growth factor 4 [Source:HGNC Sy...
2250	FGF5	fibroblast growth factor 5 [Source:HGNC Sy...
2251	FGF6	fibroblast growth factor 6 [Source:HGNC Sy...
2252	FGF7	fibroblast growth factor 7 [Source:HGNC Sy...
2253	FGF8	fibroblast growth factor 8 [Source:HGNC Sy...
2254	FGF9	fibroblast growth factor 9 [Source:HGNC Sy...
2255	FGF10	fibroblast growth factor 10 [Source:HGNC S...
2256	FGF11	fibroblast growth factor 11 [Source:HGNC S...
2257	FGF12	fibroblast growth factor 12 [Source:HGNC S...
2258	FGF13	fibroblast growth factor 13 [Source:HGNC S...
2259	FGF14	fibroblast growth factor 14 [Source:HGNC S...
2260	FGFR1	fibroblast growth factor receptor 1 [Sourc...
2261	FGFR3	fibroblast growth factor receptor 3 [Sourc...
2263	FGFR2	fibroblast growth factor receptor 2 [Sourc...
2264	FGFR4	fibroblast growth factor receptor 4 [Sourc...
22800	RRAS2	RAS related 2 [Source:HGNC Symbol;Acc:HGNC...
22801	ITGA11	integrin subunit alpha 11 [Source:HGNC Sym...
22808	MRAS	muscle RAS oncogene homolog [Source:HGNC S...
23191	CYFIP1	cytoplasmic FMR1 interacting protein 1 [So...
2335	FN1	fibronectin 1 [Source:HGNC Symbol;Acc:HGNC...
23365	ARHGEF12	Rho guanine nucleotide exchange factor 12 ...
23396	PIP5K1C	phosphatidylinositol-4-phosphate 5-kinase ...
23533	PIK3R5	phosphoinositide-3-kinase regulatory subun...
26230	TIAM2	TIAM Rac1 associated GEF 2 [Source:HGNC Sy...
26281	FGF20	fibroblast growth factor 20 [Source:HGNC S...
26291	FGF21	fibroblast growth factor 21 [Source:HGNC S...
26999	CYFIP2	cytoplasmic FMR1 interacting protein 2 [So...
27006	FGF22	fibroblast growth factor 22 [Source:HGNC S...
2768	GNA12	G protein subunit alpha 12 [Source:HGNC Sy...
28964	GIT1	GIT ArfGAP 1 [Source:HGNC Symbol;Acc:HGNC:...
2909	ARHGAP35	Rho GTPase activating protein 35 [Source:H...
2934	GSN	gelsolin [Source:HGNC Symbol;Acc:HGNC:4620]
29895	MYLPF	myosin light chain, phosphorylatable, fast...

3071	NCKAP1L	NCK associated protein 1 like [Source:HGNC...
324	APC	APC regulator of WNT signaling pathway [So...
3265	HRAS	HRas proto-oncogene, GTPase [Source:HGNC S...
345456	PFN3	profilin 3 [Source:HGNC Symbol;Acc:HGNC:18...
3630	INS	insulin [Source:HGNC Symbol;Acc:HGNC:6081]
3645	INSRR	insulin receptor related receptor [Source:...
3655	ITGA6	integrin subunit alpha 6 [Source:HGNC Symb...
3672	ITGA1	integrin subunit alpha 1 [Source:HGNC Symb...
3673	ITGA2	integrin subunit alpha 2 [Source:HGNC Symb...
3674	ITGA2B	integrin subunit alpha 2b [Source:HGNC Sym...
3675	ITGA3	integrin subunit alpha 3 [Source:HGNC Symb...
3676	ITGA4	integrin subunit alpha 4 [Source:HGNC Symb...
3678	ITGA5	integrin subunit alpha 5 [Source:HGNC Symb...
3679	ITGA7	integrin subunit alpha 7 [Source:HGNC Symb...
3680	ITGA9	integrin subunit alpha 9 [Source:HGNC Symb...
3681	ITGAD	integrin subunit alpha D [Source:HGNC Symb...
3682	ITGAE	integrin subunit alpha E [Source:HGNC Symb...
3683	ITGAL	integrin subunit alpha L [Source:HGNC Symb...
3684	ITGAM	integrin subunit alpha M [Source:HGNC Symb...
3685	ITGAV	integrin subunit alpha V [Source:HGNC Symb...
3687	ITGAX	integrin subunit alpha X [Source:HGNC Symb...
3688	ITGB1	integrin subunit beta 1 [Source:HGNC Symbo...
3689	ITGB2	integrin subunit beta 2 [Source:HGNC Symbo...
369	ARAF	A-Raf proto-oncogene, serine/threonine kin...
3690	ITGB3	integrin subunit beta 3 [Source:HGNC Symbo...
3691	ITGB4	integrin subunit beta 4 [Source:HGNC Symbo...
3693	ITGB5	integrin subunit beta 5 [Source:HGNC Symbo...
3694	ITGB6	integrin subunit beta 6 [Source:HGNC Symbo...
3695	ITGB7	integrin subunit beta 7 [Source:HGNC Symbo...
3696	ITGB8	integrin subunit beta 8 [Source:HGNC Symbo...
375189	PFN4	profilin family member 4 [Source:HGNC Symb...
3845	KRAS	KRAS proto-oncogene, GTPase [Source:HGNC S...
387	RHOA	ras homolog family member A [Source:HGNC S...
3984	LIMK1	LIM domain kinase 1 [Source:HGNC Symbol;Ac...
3985	LIMK2	LIM domain kinase 2 [Source:HGNC Symbol;Ac...
4342	MOS	MOS proto-oncogene, serine/threonine kinas...
4478	MSN	moesin [Source:HGNC Symbol;Acc:HGNC:7373]
4627	MYH9	myosin heavy chain 9 [Source:HGNC Symbol;A...
4628	MYH10	myosin heavy chain 10 [Source:HGNC Symbol;...
4633	MYL2	myosin light chain 2 [Source:HGNC Symbol;A...
4636	MYL5	myosin light chain 5 [Source:HGNC Symbol;A...
4638	MYLK	myosin light chain kinase [Source:HGNC Sym...
4659	PPP1R12A	protein phosphatase 1 regulatory subunit 1...
4893	NRAS	NRAS proto-oncogene, GTPase [Source:HGNC S...
5058	PAK1	p21 (RAC1) activated kinase 1 [Source:HGNC...
5062	PAK2	p21 (RAC1) activated kinase 2 [Source:HGNC...

5063	PAK3	p21 (RAC1) activated kinase 3 [Source:HGNC...
50649	ARHGEF4	Rho guanine nucleotide exchange factor 4 [...
5154	PDGFA	platelet derived growth factor subunit A [...
5155	PDGFB	platelet derived growth factor subunit B [...
5156	PDGFRA	platelet derived growth factor receptor al...
5159	PDGFRB	platelet derived growth factor receptor be...
5216	PFN1	profilin 1 [Source:HGNC Symbol;Acc:HGNC:8881]
5217	PFN2	profilin 2 [Source:HGNC Symbol;Acc:HGNC:8882]
5290	PIK3CA	phosphatidylinositol-4,5-bisphosphate 3-ki...
5291	PIK3CB	phosphatidylinositol-4,5-bisphosphate 3-ki...
5293	PIK3CD	phosphatidylinositol-4,5-bisphosphate 3-ki...
5294	PIK3CG	phosphatidylinositol-4,5-bisphosphate 3-ki...
5295	PIK3R1	phosphoinositide-3-kinase regulatory subun...
5296	PIK3R2	phosphoinositide-3-kinase regulatory subun...
5305	PIP4K2A	phosphatidylinositol-5-phosphate 4-kinase ...
54434	SSH1	slingshot protein phosphatase 1 [Source:HG...
54961	SSH3	slingshot protein phosphatase 3 [Source:HG...
5499	PPP1CA	protein phosphatase 1 catalytic subunit al...
5500	PPP1CB	protein phosphatase 1 catalytic subunit be...
5501	PPP1CC	protein phosphatase 1 catalytic subunit ga...
55740	ENAH	ENAH actin regulator [Source:HGNC Symbol;A...
55845	BRK1	BRICK1 subunit of SCAR/WAVE actin nucleati...
5594	MAPK1	mitogen-activated protein kinase 1 [Source...
5595	MAPK3	mitogen-activated protein kinase 3 [Source...
55970	GNG12	G protein subunit gamma 12 [Source:HGNC Sy...
56034	PDGFC	platelet derived growth factor C [Source:H...
5604	MAP2K1	mitogen-activated protein kinase kinase 1 ...
5605	MAP2K2	mitogen-activated protein kinase kinase 2 ...
56924	PAK6	p21 (RAC1) activated kinase 6 [Source:HGNC...
57144	PAK5	p21 (RAC1) activated kinase 5 [Source:HGNC...
5747	PTK2	protein tyrosine kinase 2 [Source:HGNC Sym...
5829	PXN	paxillin [Source:HGNC Symbol;Acc:HGNC:9718]
58498	MYL7	myosin light chain 7 [Source:HGNC Symbol;A...
5879	RAC1	Rac family small GTPase 1 [Source:HGNC Sym...
5880	RAC2	Rac family small GTPase 2 [Source:HGNC Sym...
5881	RAC3	Rac family small GTPase 3 [Source:HGNC Sym...
5894	RAF1	Raf-1 proto-oncogene, serine/threonine kin...
5962	RDX	radixin [Source:HGNC Symbol;Acc:HGNC:9944]
60	ACTB	actin beta [Source:HGNC Symbol;Acc:HGNC:132]
6093	ROCK1	Rho associated coiled-coil containing prot...
623	BDKRB1	bradykinin receptor B1 [Source:HGNC Symbol...
6237	RRAS	RAS related [Source:HGNC Symbol;Acc:HGNC:1...
624	BDKRB2	bradykinin receptor B2 [Source:HGNC Symbol...
6548	SLC9A1	solute carrier family 9 member A1 [Source:...
6654	SOS1	SOS Ras/Rac guanine nucleotide exchange fa...
6655	SOS2	SOS Ras/Rho guanine nucleotide exchange fa...

673	BRAF	B-Raf proto-oncogene, serine/threonine kin...
7074	TIAM1	TIAM Rac1 associated GEF 1 [Source:HGNC Sy...
71	ACTG1	actin gamma 1 [Source:HGNC Symbol;Acc:HGNC...
7114	TMSB4X	thymosin beta 4 X-linked [Source:HGNC Symb...
7117	TMSB4XP8	TMSB4X pseudogene 8 [Source:HGNC Symbol;Ac...
7409	VAV1	vav guanine nucleotide exchange factor 1 [...
7410	VAV2	vav guanine nucleotide exchange factor 2 [...
7414	VCL	vinculin [Source:HGNC Symbol;Acc:HGNC:12665]
7430	EZR	ezrin [Source:HGNC Symbol;Acc:HGNC:12691]
7454	WAS	WASP actin nucleation promoting factor [So...
79784	MYH14	myosin heavy chain 14 [Source:HGNC Symbol;...
79837	PIP4K2C	phosphatidylinositol-5-phosphate 4-kinase ...
80310	PDGFD	platelet derived growth factor D [Source:H...
8074	FGF23	fibroblast growth factor 23 [Source:HGNC S...
81	ACTN4	actinin alpha 4 [Source:HGNC Symbol;Acc:HG...
81624	DIAPH3	diaphanous related formin 3 [Source:HGNC S...
81873	ARPC5L	actin related protein 2/3 complex subunit ...
8394	PIP5K1A	phosphatidylinositol-4-phosphate 5-kinase ...
8395	PIP5K1B	phosphatidylinositol-4-phosphate 5-kinase ...
8396	PIP4K2B	phosphatidylinositol-5-phosphate 4-kinase ...
8503	PIK3R3	phosphoinositide-3-kinase regulatory subun...
8515	ITGA10	integrin subunit alpha 10 [Source:HGNC Sym...
8516	ITGA8	integrin subunit alpha 8 [Source:HGNC Symb...
85366	MYLK2	myosin light chain kinase 2 [Source:HGNC S...
85464	SSH2	slingshot protein phosphatase 2 [Source:HG...
85477	SCIN	scinderin [Source:HGNC Symbol;Acc:HGNC:21695]
87	ACTN1	actinin alpha 1 [Source:HGNC Symbol;Acc:HG...
88	ACTN2	actinin alpha 2 [Source:HGNC Symbol;Acc:HG...
8817	FGF18	fibroblast growth factor 18 [Source:HGNC S...
8822	FGF17	fibroblast growth factor 17 [Source:HGNC S...
8823	FGF16	fibroblast growth factor 16 [Source:HGNC S...
8826	IQGAP1	IQ motif containing GTPase activating prot...
8874	ARHGEF7	Rho guanine nucleotide exchange factor 7 [...
89	ACTN3	actinin alpha 3 [Source:HGNC Symbol;Acc:HG...
8936	WASF1	WASP family member 1 [Source:HGNC Symbol;A...
8976	WASL	WASP like actin nucleation promoting facto...
89846	FGD3	FYVE, RhoGEF and PH domain containing 3 [S...
9087	TMSB4Y	thymosin beta 4 Y-linked [Source:HGNC Symb...
9138	ARHGEF1	Rho guanine nucleotide exchange factor 1 [...
91807	MYLK3	myosin light chain kinase 3 [Source:HGNC S...
929	CD14	CD14 molecule [Source:HGNC Symbol;Acc:HGNC...
93408	MYL10	myosin light chain 10 [Source:HGNC Symbol;...
9459	ARHGEF6	Rac/Cdc42 guanine nucleotide exchange fact...
9475	ROCK2	Rho associated coiled-coil containing prot...
9564	BCAR1	BCAR1 scaffold protein, Cas family member ...
9965	FGF19	fibroblast growth factor 19 [Source:HGNC S...

998	CDC42	cell division cycle 42 [Source:HGNC Symbol...

S3I - Hedgehog signaling
https://www.gsea-msigdb.org/gsea/msigdb/human/geneset/KEGG_HEDGEHOG_SIGNALING_PATHWAY.html (56 genes)

NCBI (Entrez) Gene Id	Gene Symbol	Gene Description
122011	CSNK1A1L	casein kinase 1 alpha 1 like [Source:HGNC ...
1452	CSNK1A1	casein kinase 1 alpha 1 [Source:HGNC Symbo...
1453	CSNK1D	casein kinase 1 delta [Source:HGNC Symbol;...
1454	CSNK1E	casein kinase 1 epsilon [Source:HGNC Symbo...
1455	CSNK1G2	casein kinase 1 gamma 2 [Source:HGNC Symbo...
1456	CSNK1G3	casein kinase 1 gamma 3 [Source:HGNC Symbo...
23291	FBXW11	F-box and WD repeat domain containing 11 [...
2619	GAS1	growth arrest specific 1 [Source:HGNC Symb...
27148	STK36	serine/threonine kinase 36 [Source:HGNC Sy...
2735	GLI1	GLI family zinc finger 1 [Source:HGNC Symb...
2736	GLI2	GLI family zinc finger 2 [Source:HGNC Symb...
2737	GLI3	GLI family zinc finger 3 [Source:HGNC Symb...
2932	GSK3B	glycogen synthase kinase 3 beta [Source:HG...
353500	BMP8A	bone morphogenetic protein 8a [Source:HGNC...
3549	IHH	Indian hedgehog signaling molecule [Source...
4036	LRP2	LDL receptor related protein 2 [Source:HGN...
50846	DHH	desert hedgehog signaling molecule [Source...
51384	WNT16	Wnt family member 16 [Source:HGNC Symbol;A...
51684	SUFU	SUFU negative regulator of hedgehog signal...
51715	RAB23	RAB23, member RAS oncogene family [Source:...
53944	CSNK1G1	casein kinase 1 gamma 1 [Source:HGNC Symbo...
54361	WNT4	Wnt family member 4 [Source:HGNC Symbol;Ac...
5566	PRKACA	protein kinase cAMP-activated catalytic su...
5567	PRKACB	protein kinase cAMP-activated catalytic su...
5568	PRKACG	protein kinase cAMP-activated catalytic su...
5613	PRKX	protein kinase X-linked [Source:HGNC Symbo...
5727	PTCH1	patched 1 [Source:HGNC Symbol;Acc:HGNC:9585]
64399	HHIP	hedgehog interacting protein [Source:HGNC ...
6469	SHH	sonic hedgehog signaling molecule [Source:...
650	BMP2	bone morphogenetic protein 2 [Source:HGNC ...
652	BMP4	bone morphogenetic protein 4 [Source:HGNC ...
653	BMP5	bone morphogenetic protein 5 [Source:HGNC ...
654	BMP6	bone morphogenetic protein 6 [Source:HGNC ...
655	BMP7	bone morphogenetic protein 7 [Source:HGNC ...
656	BMP8B	bone morphogenetic protein 8b [Source:HGNC...
6608	SMO	smoothened, frizzled class receptor [Sourc...
7471	WNT1	Wnt family member 1 [Source:HGNC Symbol;Ac...
7472	WNT2	Wnt family member 2 [Source:HGNC Symbol;Ac...

7473	WNT3	Wnt family member 3 [Source:HGNC Symbol;Ac...
7474	WNT5A	Wnt family member 5A [Source:HGNC Symbol;A...
7475	WNT6	Wnt family member 6 [Source:HGNC Symbol;Ac...
7476	WNT7A	Wnt family member 7A [Source:HGNC Symbol;A...
7477	WNT7B	Wnt family member 7B [Source:HGNC Symbol;A...
7478	WNT8A	Wnt family member 8A [Source:HGNC Symbol;A...
7479	WNT8B	Wnt family member 8B [Source:HGNC Symbol;A...
7480	WNT10B	Wnt family member 10B [Source:HGNC Symbol;...
7481	WNT11	Wnt family member 11 [Source:HGNC Symbol;A...
7482	WNT2B	Wnt family member 2B [Source:HGNC Symbol;A...
7483	WNT9A	Wnt family member 9A [Source:HGNC Symbol;A...
7484	WNT9B	Wnt family member 9B [Source:HGNC Symbol;A...
7546	ZIC2	Zic family member 2 [Source:HGNC Symbol;Ac...
80326	WNT10A	Wnt family member 10A [Source:HGNC Symbol;...
81029	WNT5B	Wnt family member 5B [Source:HGNC Symbol;A...
8643	PTCH2	patched 2 [Source:HGNC Symbol;Acc:HGNC:9586]
8945	BTRC	beta-transducin repeat containing E3 ubiqu...
89780	WNT3A	Wnt family member 3A [Source:HGNC Symbol;A...

Manuscript with complete references (n=275)

Introduction

The evolutionary conserved *NEAT1-MALAT1* gene cluster encounters high interest in both cardiovascular medicine and oncology. In the cardiovascular field, we observed suppression of lncRNA *NEAT1* in circulating immune cells of post-myocardial infarction (MI) patients [1]. Mice lacking lncRNAs *NEAT1* [1] or *MALAT1* [2-4] displayed immune disturbances affecting monocyte-macrophage and T cell differentiation and rendering the immune system highly vulnerable to stress stimuli, thereby promoting the development of atherosclerosis [1, 2, 4-8]. Uncontrolled inflammation is also a key driver of multiple other diseases [1, 2, 9-19].

Here, we report biological functions of two tRNA-like transcripts from the *NEAT1-MALAT1* cluster (Fig. 1) and describe their deep impact upon innate immunity and macrophage functions. While we previously investigated mice deficient in the entire *NEAT1* or *MALAT1* locus, we now aimed to selectively disrupt only the tRNA-like transcript 'menRNA' arising from *NEAT1* [17, 20, 21], or 'mascRNA' arising from *MALAT1.* No biological function independent of its precursor *NEAT1* has been assigned to menRNA so far, while a few studies have addressed mascRNA. After a report that mascRNA is involved in cardiovascular innate immunity [3], Sun *et al.* conducted an in-depth study demonstrating that mascRNA differentially regulates TLR-induced proinflammatory and antiviral responses [22]. Lu et al. showed that mascRNA promotes global protein translation, uncovering another role of mascRNA that is independent of *MALAT1* [23].

The two closely neighbouring gene loci give rise to transcripts of vastly different size (*NEAT1*: 23kb MEN-β, 3.7kb MEN-ε, 59nt 'menRNA'; *MALAT1*: 8.3 kb primary, 58nt 'mascRNA'), and traditional knockout methods are unsuitable to selectively inactivate one of the small transcripts only. Through CRISPR-Cas9 editing [24-27], we therefore developed human monocyte-macrophage cell lines with short deletions in the respective tRNA-encoding sequences to disrupt normal menRNA or mascRNA formation, respectively. These editing procedures occur outside of the primary transcript sequences required for regular formation of the triple-helix structures at their 3′-ends which support stabilization of the respective lncRNAs (Fig. 1AB). Our study may be considered extension of a previous pioneering study which has identified, for the first time, functional domains within MEN-β through CRIPSR-Cas9 based deletion mapping [17]. While covering the entire length of MEN-β, that study has not reported on deletions downstream of the 3′-terminal A-rich motif essential for triple-helix formation and stabilization of MEN-β. Our ΔmenRNA clone disrupts the menRNA sequence 21nt downstream of the A-rich motif, thus leaving triple helix formation and MEN-β intact (Fig. 1C). Our CRISPR-Cas9-based editing of menRNA or mascRNA selectively prevented only the normal transcript folding and formation of mature menRNA or mascRNA, respectively. Unlike monocytes and macrophages in *NEAT1*$^{-/-}$ mice [1], CRISPR-Cas9-generated ΔmenRNA cells retained MEN-β and MEN-ε expression (Fig. 1D). Similarly, ΔmascRNA monocytes preserved expression of the long *MALAT1* precursor while mascRNA, normally highly enriched in this cell type, became ablated (Fig. 1E).

Beyond prior work documenting immune function of the *NEAT1-MALAT1* cluster, the current study identifies menRNA as a novel element of innate immunity impacting upon cytokine regulation, immune cell-endothelium interactions, angiogenesis, and monocyte-macrophage differentiation and functions. For mascRNA, impact upon cell-cell interactions and angiogenesis are described. These small transcript may be considered as prototypes of a new class of RNAs distinct from other small transcripts (miRNAs, siRNAs) by biosynthetic pathway and intracellular kinetics. From an evolutionary perspective, the *NEAT1-MALAT1* genomic region appears as archetype of a functionally highly integrated RNA processing system.

Results

Targeted deletion of tRNA-like transcripts from the NEAT1-MALAT1 cluster

While previously investigated mice were deficient in the entire *NEAT1* or *MALAT1* locus [1, 2, 4], we here aimed to selectively disrupt only the novel 59-nt tRNA-like transcript 'menRNA' with so far unknown biological functions (Fig. 1A). Through CRISPR-Cas9 editing, we developed human THP-1 monocyte-macrophage cell lines

with deletions immendiately downstream of the triple-helix ends of MEN-β (Fig. 1AC) or *MALAT1* (Fig. 1B), respectively. All of theseprevent normal transcript folding and formation of menRNA or mascRNA, respectively, as shown in detail in Fig. 1E.

We examined whether the absence of menRNA or mascRNA in ΔmenRNA of ΔmascRNA monocytes affects, by some cytosolic-nuclear or other feedback mechanism, the cellular expression levels of the long precursors. Unlike monocytes/macrophages in *NEAT1*$^{-/-}$ [1] or *MALAT1*$^{-/-}$ [2] mice, the CRISPR/Cas9-generated ΔmenRNA monocytes/macrophages retained apparently normal MEN-β and MEN-ε expression as assessed by RT-PCR (Fig. 1D). Similarly, ΔmascRNA monocytes/macrophages preserved expression of the *MALAT1* precursor (Fig. 1D) although mascRNA, normally highly enriched in this cell type, became ablated (Fig. 1E).

As a consequence of its rapid turnover, the cellular steady-state level of menRNA is very low. Low menRNA abundance in immune cells is in sharp contrast to the enrichment of mascRNA in monocytes first described by our group [3]. In that former study we documented high efficacy of antisense oligonucleotides (ASOs) targeting the mascRNA sequence, regarding reduction of the mascRNA level in monocytes [3]. Similarly, in the present CRISPR-Cas9 study it was straighforward to confirm absence of mascRNA or mascRNA-homologous transcripts in ΔmascRNA cells by Northern blot analysis (Fig. 1G).

Prove of efficacy and specificity of menRNA deletion was less straightforward due to its low abundance in organs and cell lines. Successful and precise disruption of the menRNA-forming sequence 3′ of the triple-helix was proven by regular sequencing of the target region (Fig. 1D) in each of the ΔmenRNA clone passages used for the experiments in this project. Of note, this deletion remained stable over >30 passages, enabling conduction of a major series of experiments. On the other hand, proof of menRNA expression in wildtype THP-1 cells required Northern blot analysis with high RNA input loading and very long exposure times (Fig. 1F).

Conventionally, rescue experiments employing vector-mediated overexpression of menRNA in ΔmenRNA cells would be considered suitable to further prove specificity of cellular effects of menRNA deletion. We conducted "rescue" experiments with recombinant menRNA overexpression employing vectors as specified in Fig. 1H. We previously used and characterized these vectors for short hairpin RNA (shRNA) expression and RNA interference (RNAi) induction [28, 29]. The U6 promoter therein allows for efficient RNA polymerase III dependent expression of short transcripts without poly-A tail *e.g.* menRNA. Results of a short-term experiment with partial recovery of cytokine gene deregulations are shown in Fig. 4D. Of note, it was technically unfeasible to conduct long-term "rescue" in the experiments shown in Fig. 5-9, because recombinant menRNA levels decayed rapidly and repeat vector transduction was unpracticable in these complex long-term assays.

Beyond these technical issues, a principal problem of menRNA "rescue" arises from the fact that recombinant menRNA is generated by direct linear synthesis out of the vector upon its entry into the cytosol *via* endocytosis. In contrast, endogenous menRNA biosynthesis occurs through RNase P and Z cleavage out of the nuclear MEN-β transcript, normally followed by 3′-terminal CCA and CCACCA addition [21, 30] with strong impact upon menRNA stability [30] and induction of the immune response [31]. As a side remark it may be noted that the menRNA is stabilized when expressed from the constitutive RNA polymerase III promoter in the vector, and that an additional CCA motif in the construct impairs its stability. In the particular case of menRNA we therefore consider clearcut "rescue" precisely recreating the wildtype situation in ΔmenRNA cells technically unfeasible.

Due to their known posttranscriptional mechanisms of action, neither ASOs nor siRNAs selectively interact only with the short menRNA or mascRNA sequences. Instead, they will also interact with these sequences when still embedded in the long precursors transcripts from *NEAT1* and *MALAT1* and may mediate their premature decay, making unequivocal distinction between consequences of menRNA/mascRNA ablation from those of *NEAT1*/*MALAT1* reduction [1, 2, 4] impossible. In the peculiar case of the high-turnover menRNA, the CRISPR-Cas9 deletion clone with its deletion at the DNA level is obviously a particularly useful highly specific and stable system for selective menRNA ablation and functional assignment of cellular functions to menRNA.

Regarding *in vivo* studies, any attempt to generate germ-line menRNA or mascRNA deficient animal models will be technically demanding and likely also display developmental/embryonic anomalies and disturbance of diverse somatic cells other than immune cells/monocytes. One would instead need mice with a monocyte-specific, adult-age inducible, selective knockout of the menRNA or mascRNA sequence only to resolve these issues. Beyond these technical challenges, there exists no murine homologue to human IL-8 which was, however, the

most strongly deregulated cytokine in circulating immune cells of post-MI patients [1]. As a first step, these ambiguities are avoided by CRISPR-mediated deletion in one specific cell type/line of human origin. We examined whether absence of menRNA in ΔmenRNA monocytes affects, by some cytosolic-nuclear feedback mechanism, the cellular expression level of *NEAT1*. Under the experimental conditions employed here, however, there was no change as detectable by RNA-sequencing (RNA-seq) and qRT-PCR

Defective innate immune sensing by ΔmenRNA and ΔmascRNA cells

RNA-seq was employed as genome-wide screening tool to detect any alterations in the protein-coding or noncoding transcriptome of wildtype vs. ΔmenRNA or ΔmascRNA cells (Fig. 2). Based on the RNA-seq data, we subsequently investigated a large number of deregulated genes by qRT-PCR under different conditions (Fig. 3 to 9), in monocytes (Fig. 4 to 6) as well as the corresponding differentiated macrophage (Fig. 7-9) clones.

RNA-seq identified profound alterations in the baseline transcriptomes of ΔmenRNA and ΔmascRNA monocytes. These are shown in Fig. 2AB as Gene set enrichment analysis (GSEA) plots. The Suppl. Tables S1/S2 provide FKPM details of this RNA-seq study for a large number of key deregulated transcripts. The rainbow heat maps in Fig. 3-5 and Fig. 8 are excerpts of functional gene groups relevant for the respective chapter (e.g. innate immune sensors in Fig. 3AB). GSEA identified immune response (red dots) and cell-cell interaction (green dots) associated biological processes enriched by genes deregulated in ΔmenRNA cells (Inset): primary immunodeficiency, cytokine-cytokine receptor interaction, JAK-STAT-signaling [32], hedgehog signaling [33, 34], focal adhesion, GAP junction, extracellular matrix receptfor interaction, regulation of actin cytoskeleton.

A prominent finding was gross dysbalance between multiple innate immune sensors in ΔmenRNA monocytes (Fig. 3AB, Table S1A), including cytosolic NOD-like receptors NOD2 [35-42] and CIITA [43-45], NLR-class receptors NLRC3 [46-48], NLRC4 [48, 49] and MEFV [48, 50, 51], and membrane-bound Toll-like receptors (TLR1, TLR2, TLR7, TLR10). In the context of baseline induction of NOD2 and TLR2, it is notable that twist family bHLH transcription factor 2 (TWIST2) [52] was ~10-fold down in ΔmenRNA cells (Fig. 3GH), and loss of NLRC3 and TWIST2 expression in ΔmenRNA monocytes could not be rescued by LPS (Fig. 3E)(compare Fig. 6A). ΔmascRNA monocytes displayed induction of interferon (IFN)-induced transmembrane (IFITM) proteins [53](Fig. 3C, Table S2A).

Transcription, translation, and epigenome level anomalies in defective monocytes

Transcription and nuclear factors and epigenome modifiers (Fig. 3G-I, Tables S1B/S2B), as well as translation factors, ribosomal proteins and nucleic acid modifiers (Fig. 3K-M, Tables S1C/S2C) were deregulated in defective cells. At the transcriptional level, in addition to TWIST2, transcription factor (TF) GATA2 was ~4-fold induced and GATA6 ~20-fold suppressed [54] (Fig. 3DE), and NFATC2 [55] switched off in ΔmenRNA cells (Fig. 3G). Further TFs and epigenome modifiers turned into disequilibrium upon LPS challenge (Fig. 3H), among them histone deacetylases, lysine demethylases, and METTL methyltransferases [56]. At the level of translation, three initiation and elongation-related factors (EIF3CL, EEF1A2, CTIF) were induced while initiation factor EIF1AY [57, 58] and ribosomal protein RPS4Y1 were 33-fold and 45-fold, respectively, downregulated in ΔmenRNA cells (Fig. 3KL). Nucleic acid-modifying enzymes deregulated include DEAD-box helicase DDX3Y (333-fold downregulated), and NOP2/Sun methyltransferase NSUN7 [59-65] undetectable in controls but robustly expressed in ΔmenRNA cells (FKPM 22)(Fig. 3K, Table S1C). At the level of tRNAs, genomically or mitochondrially encoded tRNAs [61, 63] and tRNA methyltransferases turned into imbalance. In ΔmascRNA cells (Fig. 3M), cytosolic nucleotidase NT5C1A [66] dephosphorylating 5' and 2'(3')-phosphates of deoxyribonucleotides with broad substrate specificity, is suppressed. Furthermore, RNA-seq identified deregulation of long noncoding RNAs (lncRNAs) and antisense (AS) RNAs in ΔmenRNA and ΔmascRNA monocytes (Tables S1D/2D). Thus, ΔmenRNA cells showed ~7-fold downregulation of a recently discovered novel transcript designated 'MARCKS cis-regulating lncRNA promoter of cytokines and inflammation' or 'regulator of cytokines and inflammation' (ROCKI) (Fig. 4DE, Table S1D/S2D). This transcript of particular interest was identified by Zhang *et al.* [67-70] by genome-wide scan of macrophages for pairs of cis-acting lncRNAs and protein-coding genes involved in innate immunity.

Excessive inflammatory cytokine production by ΔmenRNA and ΔmascRNA cells

We found massively elevated basal expression and secretion of IL-8, TNF, and IL1B in ΔmenRNA monocytes (Fig. 4AB). ΔmascRNA monocytes displayed exaggerated LPS induction of IL1A, IL1B, IL10, and ISG15 (Fig. 4C). Conversely, ΔmenRNA but not ΔmascRNA macrophages displayed blunted NOS2 expression and impaired ROS production upon LPS or H2O2 challenge (Fig. 7C). There were anomalies of further interleukin (IL)/receptor and TNF cytokine/receptor systems (Fig. 4E-G, Tables S1E/S2E). Receptor IL4R was ~7-fold and M2 macrophage polarization regulator IL4I1 [71-76] ~11-fold down in ΔmenRNA monocytes (Fig. 4EF). Even more pronounced was disequilibrium within the IL18 system [77, 78].. Receptor IL18R1 and IL18 receptor accessory protein (IL18RAP) were switched off. IL2 receptor IL2R-β (IL2RB) was ~52-fold and IL2R-γ (IL2Rc) ~13-fold suppressed [79-85]. IL1 receptor antagonist (IL1RN) was ~12-fold and IL1 receptor associated kinase 2 (IRAK2) ~8-fold down in ΔmenRNA cells. Among ligands, IL16 remained ~18-fold suppressed even after LPS stimulation. Partial "rescue" of cytokine control in ΔmenRNA monocytes was obtained by recombinant human menRNA (Fig. 4D).

'Regulator of cytokines and inflammation' (ROCKI) was ~7-fold and SLFN5, a transcriptional co-repressor of interferon (IFN) responses and antiviral restriction factor [86-89], ~10-fold suppressed in ΔmenRNA monocytes (Fig. 4EF) while another member (SLFN12L) of the SLFN family of IFN-induced genes was ~26-fold up. Several ADAM metallopeptidases (Tables S1E/S2E) as well as cluster of differentiation (CD) and other leukocyte marker proteins [90-99] (Tables S1F/S2F) were likewise deregulated and some of them switched off in ΔmenRNA (Fig. 4EF) or ΔmascRNA cells (Fig. 4G).

Disturbed growth pattern and endothelium interactions of ΔmenRNA monocytes

At the cellular level, ΔmenRNA monocytes displayed an anomalous growth pattern with spontaneous cell cluster formation in liquid culture (Fig. 5A), and defective endothelial adhesion under multiple flow conditions (Fig. 5B). The LPS-dependent induction of cell adhesion molecules (ICAM1, VCAM1) was defective in ΔmenRNA monocytes (Fig. 5C), contrasting with exacerbated induction in ΔmascRNA monocytes. ΔmenRNA monocyte-conditioned medium altered expression of multiple gene in HAEC monolayers (Fig. 5D), whereas ΔmascRNA monocyte medium had no such effect. This observation of transcriptome changes in endothelial cells under influence of the ΔmenRNA monocyte secretome prompted further studies regarding indirect influence of the anomalous monocyte clones upon endothelial cell behaviour, mediated *via* their secretomes, or *via* interaction between the monocytes and endothelial cells in co-cultures of both cell types in matrigel (Fig. 5HI). RNA-seq identified profound deregulation of further cell adhesion molecules in ΔmenRNA monocytes (Fig. 5EF, Table S1G), including adhesion G protein-coupled receptors [100], intercellular and neural cell adhesion molecules, integrins, vascular cell adhesion molecule, melanoma cell adhesion molecule MCAM (CD146), and endothelial cell-specific adhesion molecule (ESAM). ΔmascRNA cells also displayed anomalous cell adhesion molecule expression (Fig. 5G, Table S2G), however distinct from ΔmenRNA monocytes. Neuronal growth regulator NEGR1 [101, 102] is ~28-fold suppressed in ΔmenRNA but unaltered in ΔmascRNA monocytes, whereas cytoskeleton regulating NCKAP1 [103-107] was ~14-fold increased in ΔmascRNA while unaltered in ΔmenRNA cells. lncRNA SENCR, involved in maintenance of endothelial cell homeostasis [108-110], is ~12-fold down in ΔmenRNA monocytes (Table S2D), suggesting menRNA loss may lead to SENCR downregulation and cellular dysfunction in endothelial cells, too.

Impact of ΔmenRNA and ΔmascRNA monocytes upon angiogenesis

A quantitative effect of ΔmenRNA and ΔmascRNA monocyte-conditioned media upon HAEC-based tube formation was observed in matrigel assays. Supernatant from each of the defective cell clones significantly reduced the tube number (Fig. 5H). Beyond this quantitative effect of secreted factors from the cells, direct co-culture of ΔmenRNA as well as of ΔmascRNA monocytes with the HAECs in the matrigel assay lead to profound alterations of HAEC cell morphology (Fig. 5I). In co-cultures with either of the defective monocyte clones, there was a massive increase in the size of HAECs at branch side nodes where ≥3 endothelial cells came into contact. When the defective monocytes clones were stained red before addition to the HAEC-matrigel mixture, there was significant accumulation of red cells at branch side nodes where HAEC hypertrophy occurred (Fig. 5I). Consistent

with these observations, multiple growth and angiogenesis-associated factors and chemokines [111-122] were in disequilibrium in ΔmenRNA (Fig. 5KL, Table S1H) and ΔmascRNA monocytes (Fig. 5M, Table S12H).

Response of ΔmenRNA and ΔmascRNA macrophages to human-pathogenic viruses

PMA-based *in vitro* differentiation of ΔmenRNA and ΔmascRNA_monocytes into adherent macrophages rendered the cells susceptible to transduction by recombinant adenovirus expressing GFP (Suppl. Fig. S8A). Similarly, ΔmenRNA and ΔmascRNA macrophages became transducible with human coxsackievirus B3 (CVB3) and displayed an immune response to the internalized CVB3 ssRNA genome, in the absence of active CVB3 virus replication (Suppl. Fig. S8B). In ΔmenRNA macrophages, without LPS stimulation or adenovirus transduction, IL8 expression was >100-fold induced compared to controls (Fig. 6A). Adenovirus resulted in moderate IL8 decrease, while control cells retained extremely low IL8 expression upon transduction. Several other genes had significantly higher baseline expression in ΔmenRNA macrophages, either *without* response to virus (TNF, SLFN12L, NOD2, TLR2, Il1B), or with significant *further* induction upon exposure (RIG-like receptors RLR1 and RLR2, ISG 15, TGFB2). Most conspicuous was a complete shutdown of genes suppressed in ΔmenRNA monocytes (Fig. 3A-E, Fig. 4EF) already before their transformation into macrophages: NOD-like innate immune genes NLRC3 and MEFV, IL2 receptor subunits β and γ [79-85], transcription factor TWIST2 [52], and NFATC2 [55]. None of these genes fully silenced in macrophages could be 'rescued' by adenovirus exposure (Fig. 6A), similar to findings in LPS-stimulated monocytes (Fig. 3A-E, Fig. 4EF). Fig. 6B shows the response of ΔmenRNA macrophages to CVB3. Regarding IL8, their response to CVB3 with further IL8 induction beyond their already very high baseline level, was opposite to their anti-adenovirus reaction. ISG15 and Il1B responded similarly to CVB3 and RR5, while IRF7 and IRF9 expression were downregulated by CVB3 only. Inducible NO synthase (NOS2) expression as well as ROS production upon LPS or H2O2 challenge was significantly reduced in ΔmenRNA macrophages (Fig. 6C). Fig. 6D summarizes key defects of the antiviral response.

menRNA deletion critically disturbs scavenger receptor expression and oxLDL uptake

ΔmenRNA macrophages display loss of oxLDL uptake, consistent with their loss of scavenger receptor expression (Fig. 7A-C) [123-129]. ΔmenRNA cells showed further anomalies regarding receptors involved in phagocytosis: Fcγ receptors (FcγRs) [130-133] and complement receptors (CRs) [134] (Fig. 8).

Defective monocyte-macrophage transition and polarization of ΔmenRNA and ΔmascRNA cells

ΔmenRNA monocytes are unable to normally differentiate into M0 macrophages upon PMA exposure (Fig. 9A). This is consistent with disturbances of CD molecule expression in these cells, including CD11b (ITGAM), CD11c (ITGAX) [135, 136], and CD93 [97, 137-148].

Beyond monocyte-macrophage transition, there was defective M1/M2 polarization [149, 150] of ΔmenRNA and ΔmascRNA cells. Rather simple expression profiles allowed distinction between ΔmascRNA and ΔmenRNA macrophages and controls (Fig. 9BC). A "M2-like" pattern CD163^{hi} CD200R^{hi} CD206^{hi} TGFB3^{hi} TLR10^{hi} was observed in ΔmascRNA cells [151, 152]. A profile involving IL1B, CD93, TGFB2, TLR7, CSF1 and its receptor CSF1R [153-165] unequivocally characterizes ΔmenRNA monocytes-macrophages and is preserved through polarization. Upon prolonged culture, the morphological aspect of M2-polarized ΔmenRNA cell cultures was clearly distinct from control and ΔmascRNA cells (Fig. 9D).

Discussion

NEAT1 and *MALAT1* are involved in two fields of medicine (cardiovascular and malignant diseases) linked to some degree by common immune-related mechanisms [5, 6, 8, 9, 11]. A unified concept to explain the connections of this genomic region to apparently diverse diseases may be derived from recent observations regarding immunoregulatory functions of transcripts from this cluster, encompassing the current study assigning novel immune functions to both tRNA-like transcripts. Despite differences in their specific regulatory properties,

it appears that the fundamental principle of 'employing' this peculiar type of small ncRNAs was evolutionarily advantageous.

Prior studies of the NEAT1-MALAT1 cluster: We reported suppression of lncRNA *NEAT1* in circulating immune cells of post-MI patients. In mice lacking lncRNAs *NEAT1* [1] or *MALAT1* [2-4] we observed immune disturbances rendering the immune system unstable and highly vulnerable to immune stress. *MALAT1*$^{-/+}$ ApoE$^{-/-}$ mice suffered accelerated atherosclerosis despite normal diet compared to ApoE$^{-/-}$ mice. *NEAT1*$^{-/-}$ mice showed anomalous T cell and monocyte-macrophage differentiation, and systemic inflammation. *NEAT1* promotes inflammasome activation in macrophages, regulates M2 polarization [166], and influences Th17/CD4^{+} T cell differentiation. *NEAT1* knockdown induces a tolerogenic phenotype in dendritic cells by inhibiting NLRP3 inflammasome activation [167]. Further *NEAT1* or *MALAT1* related anomalies were reported in non-immune cell types: cardiomyocytes [168-171] [172], endothelial cells, and smooth muscle cells where an HDAC9-*MALAT1*-BRG1 complex mediates dysfunction [173, 174]. Clinically, *NEAT1* correlated with increased exacerbation risk, severity, and inflammation in asthma [175], and with worse disease condition and poor recurrence-free survival in acute ischemic stroke. *NEAT1* is elevated in peripheral blood cells of Parkinson disease patients [176] and abnormally expressed in a wide variety of human cancers [177].

Functional dissection of the NEAT1-menRNA system: A subset of lncRNAs, termed architectural RNAs (arcRNAs), function in formation and maintenance of phase-separated membraneless organelles. In the crowded intracellular environment these are important forms of compartmentalization. Thus, *NEAT1* is a well-characterized arcRNA acting as an essential scaffold of paraspeckle nuclear bodies [178-185].

In contrast, no biological function of menRNA independent of its precursor *NEAT1* have been described, while a few studies have already addressed mascRNA in that regard. Our functional data were obtained by CRISPR-Cas9 mediated highly selective disruption of the menRNA sequence 21nt downstream of the 3′-terminus MEN-β. Importantly, this leaves the regular triple-helix formation at the 3′-end of MEN-β unaffected, which is essential for its stabilization. Consistent with the current model of the *NEAT1*-menRNA system, the CRISPR-Cas9-generated ΔmenRNA cells displayed no alteration of MEN-β or MEN-ε expression levels.

Although the very low abundance of menRNA generates experimental challenges, specificity of the observed effects of menRNA disruption may be derived by synopsis of the following findings: 1. Northern analysis detects menRNA in the experimental model cells (wildtype and CRISPR-Cas9 control THP-1 cells); 2, sequencing confirms exactly stable deletion of essentially all of the menRNA-generating sequence; 3. consistent with this no menRNA signal is detected in the ΔmenRNA clone; 4. this deletion is clearly separate by 21nt from the upstream MEN-β transcript with its 3′ triple-helix terminus; 5. The cellular levels of MEN-β and MEN-ε appear unaffected by the menRNA disruption.

Our study may be considered extension of previous pioneering work by Yamazaki *et al.* identifying the key functional domains of MEN-β through CRIPSR-Cas9 based deletion mapping [17]. While covering the entire length of MEN-β, that study has not reported on deletions downstream of the 3′-terminal A-rich motif essential for triple-helix formation and stabilization of MEN-β.

Unless the RNaseZ cleavage site at the 3′-end of menRNA (deleted in the ΔmenRNA clone) has some unknown function other than supporting menRNA formation, the data indicate deep impact of selective menRNA disruption upon innate immunity and macrophage functions. While the current dataset does not allow to derive a mechanism of action for menRNA, the chosen experimental design should allow assignment of the observed cellular anomalies – discussed below - to menRNA *per se*.

MALAT1-independent functions of mascRNA: After a report that mascRNA is involved in cardiovascular innate immunity [3], Sun *et al.* conducted an in-depth study demonstrating that mascRNA differentially regulates TLR-induced proinflammatory and antiviral responses [22]. Lu *et al.* showed that mascRNA promotes global protein translation, uncovering another role of mascRNA that is independent of *MALAT1* [23].

Our CRISPR-Cas9-based editing of mascRNA essentially confirmed the prior reports on immunomodulating functions of mascRNA. Permanent mascRNA depletion obviates the need to repetitively add ASOs or siRNAs to

keep mascRNA down. This experimental feature significantly simplified long-term experiments (Fig. 5, 7, 9) revealing imbalance of cell-cell interactions system and angiogenesis, phagocytosis-related genes, and differentiation and polarization of ΔmascRNA monocytes and macrophages.

Critical defects of innate immune sensing: One key finding in ΔmenRNA cells was deregulation of membrane-bound (TLR2) and cytosolic immune sensors (Fig. 3). NOD2 NOD2 [35, 36, 39, 40] and CIITA [43-45] were massively induced while NLR-class receptors NLRC3 [46-48] and MEFV [48, 50, 51] were shut off in ΔmenRNA cells and could be rescued neither by LPS nor virus exposure. TWIST2, a critical regulator of cytokines in human monocyte-derived macrophages [52], NFATC2 which translocates to the nucleus upon T cell receptor stimulation [55], and IL2 receptor subunits β and γ [79-82]}[83-85] were likewise shut down and not rescuable. Further, there was massive downregulation of lncRNA ROCKI, apparently resulting from mere deletion of the small 'menRNA' sequence from an otherwise intact ~23 kb *NEAT1*. An important recent study [67] discovered that ROCKI is a master regulator of inflammatory responses. Beyond the transcription level, ΔmenRNA and ΔmascRNA cells displayed major changes of their epigenomes (Fig. 3).

Loss of inflammatory cytokine control: ΔmenRNA and ΔmascRNA cells display anomalous antiviral responses (Fig. 5). Even in absence of infectious agents or other immune challenges, however, ΔmenRNA cells display massive inductions of IL8 and TNF (Fig. 4). Regarding IL8, clinical observational studies suggest a critical role of IL8 in cardiovascular diseases [186-194] and stroke [195-197]. IL8 is mechanistically involved in the innate immune response by TLR4 signaling, induces neutrophil extracellular traps (NETs) *via* activation NFκB signaling [198], mediates hyper-signaling in aortic aneurysms, and is involved in endothelial adhesion [199]. With regard to IL8, our prior transcriptome analysis of circulating immune cells from post-MI patients [1] is of interest. IL8 was the most decisively upregulated gene post-MI, contrary to an extremely low level in healthy subjects, although all patients were receiving state-of-the-art post-MI pharmacological treatment. In that study, the long primary lncRNA transcript *NEAT1* was significantly suppressed in post-MI PBMCs. The new data from ΔmenRNA cells are consistent with the assumption that reduction of the menRNA level in PBMCs (consecutive to or independent of the observed *NEAT1* reduction) directly contributes to IL8 induction. Further, the marked IL1B induction in post-MI PBMCs would parallel the high baseline IL1B expression in ΔmenRNA cells. It would have been most interesting to directly measure menRNA levels in the post-MI PBMCs. Due to the complex secondary and tertiary structure of mature menRNA this would have required, however, Northern blot analyses for which there was insufficient RNA available in that former post-MI study. For future translational studies, our approach to study menRNA and mascRNA directly suggests new avenue since their highly dynamic levels may be more closely related to clinical parameters and clinical course than those of their nuclear precursors.

Imbalance of angiogenic factors and chemokine systems: Excessive inflammatory cytokine production appears as one downstream consequence of defective immune sensing. Imbalance of angiogenic factors, chemokines, and cell-cell adhesion molecules may be considered as further sequelae of dysfunction at the sensor level. It is not unexpected that the anomalies at this level should have far-reaching impact upon the cells' biological behavior. Thus, irrespective of differences between ΔmenRNA and ΔmascRNA cells, both significantly decreased tube formation in matrigel assay and displayed profound deregulation of cell-cell adhesion molecules (Fig. 5). In the case of ΔmenRNA cells, the latter was immediately apparent by their anomalous growth in clusters, and their defective endothelial flow adhesion to HAECs. While de-repression of multiple growth/angiogenetic factors appears directly linked to anomalous endothelial cell growth and morphology in the monocyte-endothelial cell co-cultures, this imbalance of growth/angiogenetic factors apparently does not support effective tube formation, but instead hinders it.

Defective foam cell formation and macrophage polarization: This occurs in the context of large-scale disturbance of the cells' immune sensors from NOD-like and Toll-like (Fig. 3) to phagocytosis-related receptors (Fig. 7 and 8). Overall, the cells are unable to adequately respond to immune challenges including LPS, viruses,

oxLDL, and polarization cytokines (Fig. 9). Defective interaction with endothelium and angiogenesis-promoting matrix may be added to their anomalous relationship with the environment (Fig. 5).

Loss of nucleus-to-cytosol supply of menRNA or mascRNA: As both menRNA and mascRNA are continuously exported from nucleus to cytosol under normal conditions (Fig. 10), changes of the translational/ribosomal apparatus may be linked to the complete loss of nucleus-to-cytosol supply of these tRNA-like molecules in the defective cells. Regarding definition of an underlying molecular mechanism, this is beyond the scope of the current study with an experimental strategy clearly focused upon identification of novel biological roles for mascRNA – and any such roles for menRNA – at the cellular and cell-cell interaction level. Given the extreme complexity of tRNA biology [30, 122, 200-203], a different experimental strategy will be needed to address the molecular level.

Notably, mascRNA displays very high steady-state levels within immune cells, while other cell types are essentially devoid of it, although they do express precursor *MALAT1* at normal levels [3]. Sun *et al.* conducted an in-depth study demonstrating that mascRNA differentially regulates TLR-induced proinflammatory and antiviral responses [22]. While the exact molecular function of the high mascRNA levels in immune cells remains undefined in humans and other species carrying *MALAT1*-like genomic loci [23], key importance for immune homeostasis was highly likely by inference and is definitely confirmed by the present data. In this context, a recent study by Lu *et al.* [23] is of interest showing that mascRNA binds directly to multi-tRNA synthetase complex component glutaminyl-tRNA synthetase, and promotes global protein translation and cell proliferation by positively regulating QARS protein levels. This is consistent with our working hypothesis that mascRNA and menRNA interact with the ribosomal machinery as a consequence of their tRNA-like structure.

In the case of menRNA, on the other hand, the steady-state levels in immune cells are very low. This may be entirely due, however, to the known CCACCA-tagging of menRNA for rapid degradation [21, 30, 200, 203, 204]. menRNA's primary nucleus-to-cytosol supply rate may be similar to that of mascRNA, which just remains stable as it is not CCACCA-tagged. ΔmenRNA cells display major disequilibrium at the tRNA/translational level with induction of initiation and elongation factors EIF3CL, EEF1A2 and CTIF, whereas translation initiation factor EIF1AY [58] was switched off. Further, there was deregulation of tRNAs, tRNA methyltransferases (TRMT61, TRMT2B), and RNA methyltransferase NSUN7.

Pioneering recent studies discovered hitherto unknown immune functions of tRNAs and their enzymatic processing products and may contribute to explain our current findings regarding the impact of menRNA and mascRNA ablation. tRNA products encompass tRNA-derived small RNAs (tsRNAs), tRNA-derived stress-induced RNAs (tiRNAs), and tRNA-derived fragments (tRFs). Pereira *et al.* [120] found that m(5)U54 tRNA hypomodification, from lack of methyltransferase TRMT2A, drives tsRNAs generation. As similar tRNA methyltransferases are deregulated in ΔmenRNA and ΔmascRNA cells, this may change tsRNA generation therein.

Yue *et al.* [121] found SLFN2 protection of tRNAs from stress-induced cleavage to be essential for T cell-mediated immunity. SLFN2 binds tRNAs and protects them from cleavage by the ribonuclease angiogenin (~12-fold induced in ΔmascRNA cells) [116, 120]. SLFN2 deficient T cells display accumulation of stress-induced tiRNAs which inhibit translation and promote stress-granule formation. They found IL2R-β and IL2R-γ fail to be translationally up-regulated after T cell receptor stimulation, while we observed ~52-fold IL2R-β and ~13-fold IL2R-γ suppression in ΔmenRNA monocytes upon LPS stimulation. In these cells, SLFN family member SLFN12L was ~31-fold induced whereas SLNF5 was ~10-fold suppressed compared to controls. Yue *et al.* showed ROS trigger an oxidative stress response leading to translation repression which is countered by SLFN2. In ΔmenRNA monocytes we found LPS-stimulated ROS production as well as NOS2 expression are blunted, suggesting disturbance of a regulatory circuit encompassing menRNA, SLFN family members [86, 205-211], and NO and ROS biosynthetic enzymes in these defective cells.

Genetic variability of the NEAT1-MALAT1 genomic region in humans: Since multiple data suggests inflammation control functions of the NEAT1-MALAT1 cluster, we investigated variability of this region within a cohort of 7.500 individuals from Central Europe (Suppl. Fig. S5 and S6AB). One rare MALAT1 SNP (MAF=0.01)

in the MALAT1 promotor was associated (p=0.0062) with systemic low level inflammation (CRP>3.0 mg/L) (Suppl. Fig. S7A). The current study was not designed to identify possible phenotype associations with mascRNA or menRNA sequence variants known to occur in humans (Suppl. Fig. S4). Given the functional sensitivity of the tRNA-like sequences to point mutations, it seems worthwhile to obtain full sequences in patient cohorts suffering from atherosclerosis and other disorders associated with chronic low-level inflammation.

5. Conclusions

Beyond prior work in mice documenting immune function of the *NEAT1-MALAT1* region, the current study identifies menRNA and mascRNA as novel components of innate immunity with deep impact upon cytokine regulation, immune cell - endothelium interactions, angiogenesis, and macrophage formation and functions. These tRNA-like transcripts are prototypes of a new class of ncRNAs distinct from other small transcripts (miRNAs, siRNAs) by biosynthetic pathway and intracellular kinetics, suggesting a novel link for the apparent relevance of the *NEAT1-MALAT1* cluster in cardiovascular and neoplastic diseases. The *NEAT1-MALAT1* region has emerged as a highly integrated RNA processing circuitry. Its components MEN-β, MEN-ε, menRNA, *MALAT1*, *TALAM1*, and mascRNA are set for well-balanced interactions with each other, and ablation of any element leads to major cellular and systemic dysfunction. For future translational studies, our approach to study menRNA and mascRNA directly suggests new avenue since their highly dynamic levels may be more closely related to clinical parameters and clinical course than those of their nuclear precursors. They may also have value as therapeutic targets for pharmacological intervention since they are more easily accessible than their extremely complex nuclear-located precursor molecules.

References

1. Abramiuk M, Grywalska E, Korona-Glowniak I, Niedzwiedzka-Rystwej P, Polak G, Kotarski J, Rolinski J (2020) CD200 and CD200R Expression on Peripheral Blood Lymphocytes and Serum CD200 Concentration as a New Marker of Endometriosis. J Clin Med 9 doi:10.3390/jcm9093035
2. Afzal TA, Luong LA, Chen D, Zhang C, Yang F, Chen Q, An W, Wilkes E, Yashiro K, Cutillas PR, Zhang L, Xiao Q (2016) NCK Associated Protein 1 Modulated by miRNA-214 Determines Vascular Smooth Muscle Cell Migration, Proliferation, and Neointima Hyperplasia. J Am Heart Assoc 5 doi:10.1161/JAHA.116.004629
3. Aguilo F, Li S, Balasubramaniyan N, Sancho A, Benko S, Zhang F, Vashisht A, Rengasamy M, Andino B, Chen CH, Zhou F, Qian C, Zhou MM, Wohlschlegel JA, Zhang W, Suchy FJ, Walsh MJ (2016) Deposition of 5-Methylcytosine on Enhancer RNAs Enables the Coactivator Function of PGC-1alpha. Cell Rep 14:479-492 doi:10.1016/j.celrep.2015.12.043
4. Akiyama Y, Tomioka Y, Abe T, Anderson P, Ivanov P (2021) In lysate RNA digestion provides insights into the angiogenin's specificity towards transfer RNAs. RNA Biol:1-10 doi:10.1080/15476286.2021.1930758
5. Akkuratov EE, Walters L, Saha-Mandal A, Khandekar S, Crawford E, Zirbel CL, Leisner S, Prakash A, Fedorova L, Fedorov A (2014) Bioinformatics analysis of plant orthologous introns: identification of an intronic tRNA-like sequence. Gene 548:81-90 doi:10.1016/j.gene.2014.07.012
6. Alehagen U, Shamoun L, Wagsater D (2020) Genetic variance and plasma concentration of CD93 is associated with cardiovascular mortality: Results from a 6.7year followup of a healthy communityliving elderly population. Mol Med Rep 22:4629-4636 doi:10.3892/mmr.2020.11555
7. An Z, Li J, Yu J, Wang X, Gao H, Zhang W, Wei Z, Zhang J, Zhang Y, Zhao J, Liang X (2019) Neutrophil extracellular traps induced by IL-8 aggravate atherosclerosis via activation NF-kappaB signaling in macrophages. Cell Cycle 18:2928-2938 doi:10.1080/15384101.2019.1662678
8. Angelos MG, Abrahante JE, Blum RH, Kaufman DS (2018) Single Cell Resolution of Human Hematoendothelial Cells Defines Transcriptional Signatures of Hemogenic Endothelium. Stem Cells 36:206-217 doi:10.1002/stem.2739
9. Apostolakis S, Vogiatzi K, Amanatidou V, Spandidos DA (2009) Interleukin 8 and cardiovascular disease. Cardiovasc Res 84:353-360 doi:10.1093/cvr/cvp241
10. Ard R, Maillet JC, Daher E, Phan M, Zinoviev R, Parks RJ, Gee SH (2021) PKCalpha-mediated phosphorylation of the diacylglycerol kinase zeta MARCKS domain switches cell migration modes by regulating interactions with Rac1 and RhoA. J Biol Chem:100516 doi:10.1016/j.jbc.2021.100516

11. Arslan AD, Sassano A, Saleiro D, Lisowski P, Kosciuczuk EM, Fischietti M, Eckerdt F, Fish EN, Platanias LC (2017) Human SLFN5 is a transcriptional co-repressor of STAT1-mediated interferon responses and promotes the malignant phenotype in glioblastoma. Oncogene 36:6006-6019 doi:10.1038/onc.2017.205

12. Bailey CC, Zhong G, Huang IC, Farzan M (2014) IFITM-Family Proteins: The Cell's First Line of Antiviral Defense. Annu Rev Virol 1:261-283 doi:10.1146/annurev-virology-031413-085537

13. Banani SF, Lee HO, Hyman AA, Rosen MK (2017) Biomolecular condensates: organizers of cellular biochemistry. Nat Rev Mol Cell Biol 18:285-298 doi:10.1038/nrm.2017.7

14. Bassilana F, Nash M, Ludwig MG (2019) Adhesion G protein-coupled receptors: opportunities for drug discovery. Nat Rev Drug Discov 18:869-884 doi:10.1038/s41573-019-0039-y

15. Bauche D, Joyce-Shaikh B, Jain R, Grein J, Ku KS, Blumenschein WM, Ganal-Vonarburg SC, Wilson DC, McClanahan TK, Malefyt RW, Macpherson AJ, Annamalai L, Yearley JH, Cua DJ (2018) LAG3(+) Regulatory T Cells Restrain Interleukin-23-Producing CX3CR1(+) Gut-Resident Macrophages during Group 3 Innate Lymphoid Cell-Driven Colitis. Immunity 49:342-352 e345 doi:10.1016/j.immuni.2018.07.007

16. Betat H, Morl M (2015) The CCA-adding enzyme: A central scrutinizer in tRNA quality control. Bioessays 37:975-982 doi:10.1002/bies.201500043

17. Boels MGS, Koudijs A, Avramut MC, Sol W, Wang G, van Oeveren-Rietdijk AM, van Zonneveld AJ, de Boer HC, van der Vlag J, van Kooten C, Eulberg D, van den Berg BM, DHT IJ, Rabelink TJ (2017) Systemic Monocyte Chemotactic Protein-1 Inhibition Modifies Renal Macrophages and Restores Glomerular Endothelial Glycocalyx and Barrier Function in Diabetic Nephropathy. Am J Pathol 187:2430-2440 doi:10.1016/j.ajpath.2017.07.020

18. Boros FA, Maszlag-Torok R, Vecsei L, Klivenyi P (2020) Increased level of NEAT1 long non-coding RNA is detectable in peripheral blood cells of patients with Parkinson's disease. Brain Res 1730:146672 doi:10.1016/j.brainres.2020.146672

19. Boulberdaa M, Scott E, Ballantyne M, Garcia R, Descamps B, Angelini GD, Brittan M, Hunter A, McBride M, McClure J, Miano JM, Emanueli C, Mills NL, Mountford JC, Baker AH (2016) A Role for the Long Noncoding RNA SENCR in Commitment and Function of Endothelial Cells. Mol Ther 24:978-990 doi:10.1038/mt.2016.41

20. Brocheriou I, Maouche S, Durand H, Braunersreuther V, Le Naour G, Gratchev A, Koskas F, Mach F, Kzhyshkowska J, Ninio E (2011) Antagonistic regulation of macrophage phenotype by M-CSF and GM-CSF: implication in atherosclerosis. Atherosclerosis 214:316-324 doi:10.1016/j.atherosclerosis.2010.11.023

21. Browne T, Dearman RJ, Poles A (2021) Human neutrophil antigens: Nature, clinical significance and detection. Int J Immunogenet 48:145-156 doi:10.1111/iji.12514

22. Buechler C, Eisinger K, Krautbauer S (2013) Diagnostic and prognostic potential of the macrophage specific receptor CD163 in inflammatory diseases. Inflamm Allergy Drug Targets 12:391-402

23. Callejas BE, Blyth GAD, Jendzjowsky N, Wang A, Babbar A, Koro K, Wilson RJA, Kelly MM, Cobo ER, McKay DM (2021) Interleukin-4 Programmed Macrophages Suppress Colitis and Do Not Enhance Infectious-Colitis, Inflammation-Associated Colon Cancer or Airway Hypersensitivity. Front Immunol 12:744738 doi:10.3389/fimmu.2021.744738

24. Cannarile MA, Weisser M, Jacob W, Jegg AM, Ries CH, Ruttinger D (2017) Colony-stimulating factor 1 receptor (CSF1R) inhibitors in cancer therapy. J Immunother Cancer 5:53 doi:10.1186/s40425-017-0257-y

25. Canton J, Neculai D, Grinstein S (2013) Scavenger receptors in homeostasis and immunity. Nat Rev Immunol 13:621-634 doi:10.1038/nri3515

26. Carpio MA, Michaud M, Zhou W, Fisher JK, Walensky LD, Katz SG (2015) BCL-2 family member BOK promotes apoptosis in response to endoplasmic reticulum stress. Proc Natl Acad Sci U S A 112:7201-7206 doi:10.1073/pnas.1421063112

27. Caruso R, Warner N, Inohara N, Nunez G (2014) NOD1 and NOD2: signaling, host defense, and inflammatory disease. Immunity 41:898-908 doi:10.1016/j.immuni.2014.12.010

28. Castrillo A, Tontonoz P (2004) Nuclear receptors in macrophage biology: at the crossroads of lipid metabolism and inflammation. Annu Rev Cell Dev Biol 20:455-480 doi:10.1146/annurev.cellbio.20.012103.134432

29. Chakarov S, Lim HY, Tan L, Lim SY, See P, Lum J, Zhang XM, Foo S, Nakamizo S, Duan K, Kong WT, Gentek R, Balachander A, Carbajo D, Bleriot C, Malleret B, Tam JKC, Baig S, Shabeer M, Toh SES, Schlitzer A, Larbi A, Marichal T, Malissen B, Chen J, Poidinger M, Kabashima K, Bajenoff M, Ng LG, Angeli V, Ginhoux F (2019) Two distinct interstitial macrophage populations coexist across tissues in specific subtissular niches. Science 363 doi:10.1126/science.aau0964

30. Chang SF, Chang PY, Chou YC, Lu SC (2020) Electronegative LDL Induces M1 Polarization of Human Macrophages Through a LOX-1-Dependent Pathway. Inflammation 43:1524-1535 doi:10.1007/s10753-020-01229-6

31. Chen DD, Hui LL, Zhang XC, Chang Q (2018) NEAT1 contributes to ox-LDL-induced inflammation and oxidative stress in macrophages through inhibiting miR-128. J Cell Biochem doi:10.1002/jcb.27541

32. Chen H, Xia W, Hou M (2020) LncRNA-NEAT1 from the competing endogenous RNA network promotes cardioprotective efficacy of mesenchymal stem cell-derived exosomes induced by macrophage migration inhibitory factor via the miR-142-3p/FOXO1 signaling pathway. Stem Cell Res Ther 11:31 doi:10.1186/s13287-020-1556-7

33. Chen L, Cao SQ, Lin ZM, He SJ, Zuo JP (2021) NOD-like receptors in autoimmune diseases. Acta Pharmacol Sin doi:10.1038/s41401-020-00603-2

34. Chen W, Yan Z, Li S, Huang N, Huang X, Zhang J, Zhong S (2018) RNAs as Proximity-Labeling Media for Identifying Nuclear Speckle Positions Relative to the Genome. iScience 4:204-215 doi:10.1016/j.isci.2018.06.005

35. Chen Y, Zhang Y, Wang Y, Zhang L, Brinkman EK, Adam SA, Goldman R, van Steensel B, Ma J, Belmont AS (2018) Mapping 3D genome organization relative to nuclear compartments using TSA-Seq as a cytological ruler. J Cell Biol 217:4025-4048 doi:10.1083/jcb.201807108

36. Chernykh ER, Shevela EY, Starostina NM, Morozov SA, Davydova MN, Menyaeva EV, Ostanin AA (2016) Safety and Therapeutic Potential of M2 Macrophages in Stroke Treatment. Cell Transplant 25:1461-1471 doi:10.3727/096368915X690279

37. Chevrier S, Genton C, Kallies A, Karnowski A, Otten LA, Malissen B, Malissen M, Botto M, Corcoran LM, Nutt SL, Acha-Orbea H (2009) CD93 is required for maintenance of antibody secretion and persistence of plasma cells in the bone marrow niche. Proc Natl Acad Sci U S A 106:3895-3900 doi:10.1073/pnas.0809736106

38. Chidambaram H, Das R, Chinnathambi S (2020) Interaction of Tau with the chemokine receptor, CX3CR1 and its effect on microglial activation, migration and proliferation. Cell Biosci 10:109 doi:10.1186/s13578-020-00474-4

39. Chistiakov DA, Melnichenko AA, Myasoedova VA, Grechko AV, Orekhov AN (2017) Mechanisms of foam cell formation in atherosclerosis. J Mol Med (Berl) 95:1153-1165 doi:10.1007/s00109-017-1575-8

40. Cho YS, Challa S, Moquin D, Genga R, Ray TD, Guildford M, Chan FK (2009) Phosphorylation-driven assembly of the RIP1-RIP3 complex regulates programmed necrosis and virus-induced inflammation. Cell 137:1112-1123 doi:10.1016/j.cell.2009.05.037

41. Cimini FA, Barchetta I, Porzia A, Mainiero F, Costantino C, Bertoccini L, Ceccarelli V, Morini S, Baroni MG, Lenzi A, Cavallo MG (2017) Circulating IL-8 levels are increased in patients with type 2 diabetes and associated with worse inflammatory and cardiometabolic profile. Acta Diabetol 54:961-967 doi:10.1007/s00592-017-1039-1

42. Clynes R, Maizes JS, Guinamard R, Ono M, Takai T, Ravetch JV (1999) Modulation of immune complex-induced inflammation in vivo by the coordinate expression of activation and inhibitory Fc receptors. J Exp Med 189:179-185 doi:10.1084/jem.189.1.179

43. Cominelli A, Gaide Chevronnay HP, Lemoine P, Courtoy PJ, Marbaix E, Henriet P (2014) Matrix metalloproteinase-27 is expressed in CD163+/CD206+ M2 macrophages in the cycling human endometrium and in superficial endometriotic lesions. Mol Hum Reprod 20:767-775 doi:10.1093/molehr/gau034

44. Cremer S, Michalik KM, Fischer A, Pfisterer L, Jae N, Winter C, Boon RA, Muhly-Reinholz M, John D, Uchida S, Weber C, Poller W, Gunther S, Braun T, Li DY, Maegdefessel L, Perisic Matic L, Hedin U, Soehnlein O, Zeiher A, Dimmeler S (2019) Hematopoietic Deficiency of the Long Noncoding RNA MALAT1 Promotes Atherosclerosis and Plaque Inflammation. Circulation 139:1320-1334 doi:10.1161/CIRCULATIONAHA.117.029015

45. Cruikshank WW, Kornfeld H, Center DM (2000) Interleukin-16. J Leukoc Biol 67:757-766 doi:10.1002/jlb.67.6.757

46. Czabotar PE, Lessene G, Strasser A, Adams JM (2014) Control of apoptosis by the BCL-2 protein family: implications for physiology and therapy. Nat Rev Mol Cell Biol 15:49-63 doi:10.1038/nrm3722

47. D'Souza AR, Minczuk M (2018) Mitochondrial transcription and translation: overview. Essays Biochem 62:309-320 doi:10.1042/EBC20170102

48. Dagoneau N, Bellais S, Blanchet P, Sarda P, Al-Gazali LI, Di Rocco M, Huber C, Djouadi F, Le Goff C, Munnich A, Cormier-Daire V (2007) Mutations in cytokine receptor-like factor 1 (CRLF1) account for both Crisponi and cold-induced sweating syndromes. Am J Hum Genet 80:966-970 doi:10.1086/513608

49. de Crecy-Lagard V, Boccaletto P, Mangleburg CG, Sharma P, Lowe TM, Leidel SA, Bujnicki JM (2019) Matching tRNA modifications in humans to their known and predicted enzymes. Nucleic Acids Res 47:2143-2159 doi:10.1093/nar/gkz011

50. DeNardo DG, Ruffell B (2019) Macrophages as regulators of tumour immunity and immunotherapy. Nat Rev Immunol 19:369-382 doi:10.1038/s41577-019-0127-6

51. Denny WA, Flanagan JU (2021) Small-molecule CSF1R kinase inhibitors; review of patents 2015-present. Expert Opin Ther Pat 31:107-117 doi:10.1080/13543776.2021.1839414

52. Du M, Yang L, Liu B, Yang L, Mao X, Liang M, Huang K (2021) Inhibition of NFAT suppresses foam cell formation and the development of diet-induced atherosclerosis. FASEB J 35:e21951 doi:10.1096/fj.202100947R

53. Duong CN, Nottebaum AF, Butz S, Volkery S, Zeuschner D, Stehling M, Vestweber D (2020) Interference With ESAM (Endothelial Cell-Selective Adhesion Molecule) Plus Vascular Endothelial-Cadherin Causes Immediate Lethality and Lung-Specific Blood Coagulation. Arterioscler Thromb Vasc Biol 40:378-393 doi:10.1161/ATVBAHA.119.313545

54. Eren E, Berber M, Ozoren N (2017) NLRC3 protein inhibits inflammation by disrupting NALP3 inflammasome assembly via competition with the adaptor protein ASC for pro-caspase-1 binding. J Biol Chem 292:12691-12701 doi:10.1074/jbc.M116.769695

55. Fadini GP, de Kreutzenberg SV, Boscaro E, Albiero M, Cappellari R, Krankel N, Landmesser U, Toniolo A, Bolego C, Cignarella A, Seeger F, Dimmeler S, Zeiher A, Agostini C, Avogaro A (2013) An unbalanced monocyte polarisation in peripheral blood and bone marrow of patients with type 2 diabetes has an impact on microangiopathy. Diabetologia 56:1856-1866 doi:10.1007/s00125-013-2918-9

56. Fagerholm SC, MacPherson M, James MJ, Sevier-Guy C, Lau CS (2013) The CD11b-integrin (ITGAM) and systemic lupus erythematosus. Lupus 22:657-663 doi:10.1177/0961203313491851

57. Fei J, Jadaliha M, Harmon TS, Li ITS, Hua B, Hao Q, Holehouse AS, Reyer M, Sun Q, Freier SM, Pappu RV, Prasanth KV, Ha T (2017) Quantitative analysis of multilayer organization of proteins and RNA in nuclear speckles at super resolution. J Cell Sci 130:4180-4192 doi:10.1242/jcs.206854

58. Fischietti M, Eckerdt F, Blyth GT, Arslan AD, Mati WM, Oku CV, Perez RE, Lee-Chang C, Kosciuczuk EM, Saleiro D, Beauchamp EM, Lesniak MS, Verzella D, Sun L, Fish EN, Yang GY, Qiang W, Platanias LC (2021) Schlafen 5 as a novel therapeutic target in pancreatic ductal adenocarcinoma. Oncogene 40:3273-3286 doi:10.1038/s41388-021-01761-1

59. Fore F, Indriputri C, Mamutse J, Nugraha J (2020) TLR10 and Its Unique Anti-Inflammatory Properties and Potential Use as a Target in Therapeutics. Immune Netw 20:e21 doi:10.4110/in.2020.20.e21

60. Frantz S, Vincent KA, Feron O, Kelly RA (2005) Innate immunity and angiogenesis. Circ Res 96:15-26 doi:10.1161/01.RES.0000153188.68898.ac

61. Galon J, Bruni D (2019) Approaches to treat immune hot, altered and cold tumours with combination immunotherapies. Nat Rev Drug Discov 18:197-218 doi:10.1038/s41573-018-0007-y

62. Galvagni F, Nardi F, Spiga O, Trezza A, Tarticchio G, Pellicani R, Andreuzzi E, Caldi E, Toti P, Tosi GM, Santucci A, Iozzo RV, Mongiat M, Orlandini M (2017) Dissecting the CD93-Multimerin 2 interaction involved in cell adhesion and migration of the activated endothelium. Matrix Biol 64:112-127 doi:10.1016/j.matbio.2017.08.003

63. Gao Y, Fang P, Li WJ, Zhang J, Wang GP, Jiang DF, Chen FP (2020) LncRNA NEAT1 sponges miR-214 to regulate M2 macrophage polarization by regulation of B7-H3 in multiple myeloma. Mol Immunol 117:20-28 doi:10.1016/j.molimm.2019.10.026

64. Gast M, Rauch BH, Haghikia A, Nakagawa S, Haas J, Stroux A, Schmidt D, Schumann P, Weiss S, Jensen L, Kratzer A, Kraenkel N, Muller C, Bornigen D, Hirose T, Blankenberg S, Escher F, Kuhl AA, Kuss AW, Meder B, Landmesser U, Zeller T, Poller W (2019) Long noncoding RNA NEAT1 modulates immune cell functions and is suppressed in early onset myocardial infarction patients. Cardiovasc Res 115:1886-1906 doi:10.1093/cvr/cvz085

65. Gast M, Rauch BH, Nakagawa S, Haghikia A, Jasina A, Haas J, Nath N, Jensen L, Stroux A, Bohm A, Friebel J, Rauch U, Skurk C, Blankenberg S, Zeller T, Prasanth KV, Meder B, Kuss A, Landmesser U, Poller W (2019) Immune system-mediated atherosclerosis caused by deficiency of long non-coding RNA MALAT1 in ApoE-/-mice. Cardiovasc Res 115:302-314 doi:10.1093/cvr/cvy202

66. Gast M, Schroen B, Voigt A, Haas J, Kuehl U, Lassner D, Skurk C, Escher F, Wang X, Kratzer A, Michalik K, Papageorgiou A, Peters T, Loebel M, Wilk S, Althof N, Prasanth KV, Katus H, Meder B, Nakagawa S, Scheibenbogen C, Schultheiss HP, Landmesser U, Dimmeler S, Heymans S, Poller W (2016) Long noncoding RNA MALAT1-derived mascRNA is involved in cardiovascular innate immunity. J Mol Cell Biol 8:178-181 doi:10.1093/jmcb/mjw003

67. Geirsson A, Bothwell AL, Hammond GL (2004) Inhibition of alloresponse by a human trophoblast non-coding RNA suppressing class II transactivator promoter III and major histocompatibility class II expression in murine B-lymphocytes. J Heart Lung Transplant 23:1077-1081 doi:10.1016/j.healun.2004.07.020

68. Geirsson A, Lynch RJ, Paliwal I, Bothwell AL, Hammond GL (2003) Human trophoblast noncoding RNA suppresses CIITA promoter III activity in murine B-lymphocytes. Biochem Biophys Res Commun 301:718-724

69. Geirsson A, Paliwal I, Lynch RJ, Bothwell AL, Hammond GL (2003) Class II transactivator promoter activity is suppressed through regulation by a trophoblast noncoding RNA. Transplantation 76:387-394 doi:10.1097/01.TP.0000073612.04525.46

70. Gerszten RE, Garcia-Zepeda EA, Lim YC, Yoshida M, Ding HA, Gimbrone MA, Jr., Luster AD, Luscinskas FW, Rosenzweig A (1999) MCP-1 and IL-8 trigger firm adhesion of monocytes to vascular endothelium under flow conditions. Nature 398:718-723 doi:10.1038/19546

71. Geserick P, Kaiser F, Klemm U, Kaufmann SH, Zerrahn J (2004) Modulation of T cell development and activation by novel members of the Schlafen (slfn) gene family harbouring an RNA helicase-like motif. Int Immunol 16:1535-1548 doi:10.1093/intimm/dxh155

72. Godfrey AK, Naqvi S, Chmatal L, Chick JM, Mitchell RN, Gygi SP, Skaletsky H, Page DC (2020) Quantitative analysis of Y-Chromosome gene expression across 36 human tissues. Genome Res 30:860-873 doi:10.1101/gr.261248.120

73. Greenlee-Wacker MC, Galvan MD, Bohlson SS (2012) CD93: recent advances and implications in disease. Curr Drug Targets 13:411-420 doi:10.2174/138945012799424651

74. Griffiths MR, Botto M, Morgan BP, Neal JW, Gasque P (2018) CD93 regulates central nervous system inflammation in two mouse models of autoimmune encephalomyelitis. Immunology 155:346-355 doi:10.1111/imm.12974

75. Gu P, Theiss A, Han J, Feagins LA (2017) Increased Cell Adhesion Molecules, PECAM-1, ICAM-3, or VCAM-1, Predict Increased Risk for Flare in Patients With Quiescent Inflammatory Bowel Disease. J Clin Gastroenterol 51:522-527 doi:10.1097/MCG.0000000000000618

76. Gultekin Y, Eren E, Ozoren N (2015) Overexpressed NLRC3 acts as an anti-inflammatory cytosolic protein. J Innate Immun 7:25-36 doi:10.1159/000363602

77. Guo H, Zhang Q, Dai R, Yu B, Hoekzema K, Tan J, Tan S, Jia X, Chung WK, Hernan R, Alkuraya FS, Alsulaiman A, Al-Muhaizea MA, Lesca G, Pons L, Labalme A, Laux L, Bryant E, Brown NJ, Savva E, Ayres S, Eratne D, Peeters H, Bilan F, Letienne-Cejudo L, Gilbert-Dussardier B, Ruiz-Arana IL, Merlini JM, Boizot A, Bartoloni L, Santoni F, Karlowicz D, McDonald M, Wu H, Hu Z, Chen G, Ou J, Brasch-Andersen C, Fagerberg CR, Dreyer I, Chun-Hui Tsai A, Slegesky V, McGee RB, Daniels B, Sellars EA, Carpenter LA, Schaefer B, Sacoto MJG, Begtrup A, Schnur RE, Punj S, Wentzensen IM, Rhodes L, Pan Q, Bernier RA, Chen C, Eichler EE, Xia K (2020) NCKAP1 Disruptive Variants Lead to a Neurodevelopmental Disorder with Core Features of Autism. Am J Hum Genet 107:963-976 doi:10.1016/j.ajhg.2020.10.002

78. Hagan N, Kane JL, Grover D, Woodworth L, Madore C, Saleh J, Sancho J, Liu J, Li Y, Proto J, Zelic M, Mahan A, Kothe M, Scholte AA, Fitzgerald M, Gisevius B, Haghikia A, Butovsky O, Ofengeim D (2020) CSF1R signaling is a regulator of pathogenesis in progressive MS. Cell Death Dis 11:904 doi:10.1038/s41419-020-03084-7

79. Hamann J, Aust G, Arac D, Engel FB, Formstone C, Fredriksson R, Hall RA, Harty BL, Kirchhoff C, Knapp B, Krishnan A, Liebscher I, Lin HH, Martinelli DC, Monk KR, Peeters MC, Piao X, Promel S, Schoneberg T, Schwartz TW, Singer K, Stacey M, Ushkaryov YA, Vallon M, Wolfrum U, Wright MW, Xu L, Langenhan T, Schioth HB (2015) International Union of Basic and Clinical Pharmacology. XCIV. Adhesion G protein-coupled receptors. Pharmacol Rev 67:338-367 doi:10.1124/pr.114.009647

80. Hansen M, Kuhlman ACB, Sahl RE, Kelly B, Morville T, Dohlmann TL, Chrois KM, Larsen S, Helge JW, Dela F (2019) Inflammatory biomarkers in patients in Simvastatin treatment: No effect of co-enzyme Q10 supplementation. Cytokine 113:393-399 doi:10.1016/j.cyto.2018.10.011

81. Haralambieva IH, Zimmermann MT, Ovsyannikova IG, Grill DE, Oberg AL, Kennedy RB, Poland GA (2016) Whole Transcriptome Profiling Identifies CD93 and Other Plasma Cell Survival Factor Genes Associated with Measles-Specific Antibody Response after Vaccination. PLoS One 11:e0160970 doi:10.1371/journal.pone.0160970

82. Hartman MHT, Groot HE, Leach IM, Karper JC, van der Harst P (2018) Translational overview of cytokine inhibition in acute myocardial infarction and chronic heart failure. Trends Cardiovasc Med 28:369-379 doi:10.1016/j.tcm.2018.02.003

83. He S, Wang L, Miao L, Wang T, Du F, Zhao L, Wang X (2009) Receptor interacting protein kinase-3 determines cellular necrotic response to TNF-alpha. Cell 137:1100-1111 doi:10.1016/j.cell.2009.05.021

84. Helmke A, Nordlohne J, Balzer MS, Dong L, Rong S, Hiss M, Shushakova N, Haller H, von Vietinghoff S (2019) CX3CL1-CX3CR1 interaction mediates macrophage-mesothelial cross talk and promotes peritoneal fibrosis. Kidney Int 95:1405-1417 doi:10.1016/j.kint.2018.12.030

85. Henry RJ, Ritzel RM, Barrett JP, Doran SJ, Jiao Y, Leach JB, Szeto GL, Wu J, Stoica BA, Faden AI, Loane DJ (2020) Microglial Depletion with CSF1R Inhibitor During Chronic Phase of Experimental Traumatic Brain Injury Reduces Neurodegeneration and Neurological Deficits. J Neurosci 40:2960-2974 doi:10.1523/JNEUROSCI.2402-19.2020

86. Hille F, Charpentier E (2016) CRISPR-Cas: biology, mechanisms and relevance. Philos Trans R Soc Lond B Biol Sci 371 doi:10.1098/rstb.2015.0496

87. Hille F, Richter H, Wong SP, Bratovic M, Ressel S, Charpentier E (2018) The Biology of CRISPR-Cas: Backward and Forward. Cell 172:1239-1259 doi:10.1016/j.cell.2017.11.032

88. Hogg MC, Rayner M, Susdalzew S, Monsefi N, Crivello M, Woods I, Resler A, Blackbourn L, Fabbrizio P, Trolese MC, Nardo G, Bendotti C, van den Berg LH, van Es MA, Prehn JHM (2020) 5'ValCAC tRNA fragment generated as part of a protective angiogenin response provides prognostic value in amyotrophic lateral sclerosis. Brain Commun 2:fcaa138 doi:10.1093/braincomms/fcaa138

89. Hsieh WY, Zhou QD, York AG, Williams KJ, Scumpia PO, Kronenberger EB, Hoi XP, Su B, Chi X, Bui VL, Khialeeva E, Kaplan A, Son YM, Divakaruni AS, Sun J, Smale ST, Flavell RA, Bensinger SJ (2020) Toll-Like Receptors Induce Signal-Specific Reprogramming of the Macrophage Lipidome. Cell Metab 32:128-143 e125 doi:10.1016/j.cmet.2020.05.003

90. Huang-Fu N, Cheng JS, Wang Y, Li ZW, Wang SH (2018) Neat1 regulates oxidized low-density lipoprotein-induced inflammation and lipid uptake in macrophages via paraspeckle formation. Mol Med Rep 17:3092-3098 doi:10.3892/mmr.2017.8211

91. Huang S, Xu Y, Ge X, Xu B, Peng W, Jiang X, Shen L, Xia L (2019) Long noncoding RNA NEAT1 accelerates the proliferation and fibrosis in diabetic nephropathy through activating Akt/mTOR signaling pathway. J Cell Physiol 234:11200-11207 doi:10.1002/jcp.27770

92. Huangfu N, Xu Z, Zheng W, Wang Y, Cheng J, Chen X (2018) LncRNA MALAT1 regulates oxLDL-induced CD36 expression via activating beta-catenin. Biochem Biophys Res Commun 495:2111-2117 doi:10.1016/j.bbrc.2017.12.086

93. Hume DA, Caruso M, Ferrari-Cestari M, Summers KM, Pridans C, Irvine KM (2020) Phenotypic impacts of CSF1R deficiencies in humans and model organisms. J Leukoc Biol 107:205-219 doi:10.1002/JLB.MR0519-143R

94. Hunsucker SA, Spychala J, Mitchell BS (2001) Human cytosolic 5'-nucleotidase I: characterization and role in nucleoside analog resistance. J Biol Chem 276:10498-10504 doi:10.1074/jbc.M011218200

95. Husebye T, Eritsland J, Arnesen H, Bjornerheim R, Mangschau A, Seljeflot I, Andersen GO (2014) Association of interleukin 8 and myocardial recovery in patients with ST-elevation myocardial infarction complicated by acute heart failure. PLoS One 9:e112359 doi:10.1371/journal.pone.0112359

96. Ignatova VV, Jansen P, Baltissen MP, Vermeulen M, Schneider R (2019) The interactome of a family of potential methyltransferases in HeLa cells. Sci Rep 9:6584 doi:10.1038/s41598-019-43010-2

97. Isobe M, Toya H, Mito M, Chiba T, Asahara H, Hirose T, Nakagawa S (2020) Forced isoform switching of Neat1_1 to Neat1_2 leads to the loss of Neat1_1 and the hyperformation of paraspeckles but does not affect the development and growth of mice. RNA 26:251-264 doi:10.1261/rna.072587.119

98. Iyer DN, Faruq O, Zhang L, Rastgoo N, Liu A, Chang H (2021) Pathophysiological roles of myristoylated alanine-rich C-kinase substrate (MARCKS) in hematological malignancies. Biomark Res 9:34 doi:10.1186/s40364-021-00286-9

99. Janovitz T, Wong S, Young NS, Oliveira T, Falck-Pedersen E (2017) Parvovirus B19 integration into human CD36+ erythroid progenitor cells. Virology 511:40-48 doi:10.1016/j.virol.2017.08.011

100. Ji H, Ding Z, Hawke D, Xing D, Jiang BH, Mills GB, Lu Z (2012) AKT-dependent phosphorylation of Niban regulates nucleophosmin- and MDM2-mediated p53 stability and cell apoptosis. EMBO Rep 13:554-560 doi:10.1038/embor.2012.53

101. Jia Z, Zhang Y, Li Q, Ye Z, Liu Y, Fu C, Cang X, Wang M, Guan MX (2019) A coronary artery disease-associated tRNAThr mutation altered mitochondrial function, apoptosis and angiogenesis. Nucleic Acids Res 47:2056-2074 doi:10.1093/nar/gky1241

102. Johansson ME, Zhang XY, Edfeldt K, Lundberg AM, Levin MC, Boren J, Li W, Yuan XM, Folkersen L, Eriksson P, Hedin U, Low H, Sviridov D, Rios FJ, Hansson GK, Yan ZQ (2014) Innate immune receptor NOD2 promotes vascular inflammation and formation of lipid-rich necrotic cores in hypercholesterolemic mice. Eur J Immunol 44:3081-3092 doi:10.1002/eji.201444755

103. Jones SA, Jenkins BJ (2018) Recent insights into targeting the IL-6 cytokine family in inflammatory diseases and cancer. Nat Rev Immunol 18:773-789 doi:10.1038/s41577-018-0066-7

104. Junttila IS (2018) Tuning the Cytokine Responses: An Update on Interleukin (IL)-4 and IL-13 Receptor Complexes. Front Immunol 9:888 doi:10.3389/fimmu.2018.00888

105. Kawabata K, Makino T, Makino K, Kajihara I, Fukushima S, Ihn H (2020) IL-16 expression is increased in the skin and sera of patients with systemic sclerosis. Rheumatology (Oxford) 59:519-523 doi:10.1093/rheumatology/kez318

106. Kawase T, Ichikawa H, Ohta T, Nozaki N, Tashiro F, Ohki R, Taya Y (2008) p53 target gene AEN is a nuclear exonuclease required for p53-dependent apoptosis. Oncogene 27:3797-3810 doi:10.1038/onc.2008.32

107. Kenneweg F, Bang C, Xiao K, Boulanger CM, Loyer X, Mazlan S, Schroen B, Hermans-Beijnsberger S, Foinquinos A, Hirt MN, Eschenhagen T, Funcke S, Stojanovic S, Genschel C, Schimmel K, Just A, Pfanne A, Scherf K, Dehmel S, Raemon-Buettner SM, Fiedler J, Thum T (2019) Long Noncoding RNA-Enriched Vesicles Secreted by Hypoxic Cardiomyocytes Drive Cardiac Fibrosis. Mol Ther Nucleic Acids 18:363-374 doi:10.1016/j.omtn.2019.09.003

108. Khan KA, Naylor AJ, Khan A, Noy PJ, Mambretti M, Lodhia P, Athwal J, Korzystka A, Buckley CD, Willcox BE, Mohammed F, Bicknell R (2017) Multimerin-2 is a ligand for group 14 family C-type lectins CLEC14A, CD93 and CD248 spanning the endothelial pericyte interface. Oncogene 36:6097-6108 doi:10.1038/onc.2017.214

109. Kielar M, Dumnicka P, Ignacak E, Bedkowska-Prokop A, Gala-Bladzinska A, Maziarz B, Ceranowicz P, Kusnierz-Cabala B (2021) Soluble Complement Component 1q Receptor 1 (sCD93) Is Associated with Graft Function in Kidney Transplant Recipients. Biomolecules 11 doi:10.3390/biom11111623

110. Kienhorst LB, van Lochem E, Kievit W, Dalbeth N, Merriman ME, Phipps-Green A, Loof A, van Heerde W, Vermeulen S, Stamp LK, van Koolwijk E, de Graaf J, Holzinger D, Roth J, Janssens HJ, Merriman TR, Broen JC, Janssen M, Radstake TR (2015) Gout Is a Chronic Inflammatory Disease in Which High Levels of Interleukin-8 (CXCL8), Myeloid-Related Protein 8/Myeloid-Related Protein 14 Complex, and an Altered Proteome Are Associated With Diabetes Mellitus and Cardiovascular Disease. Arthritis Rheumatol 67:3303-3313 doi:10.1002/art.39318

111. Kim ET, Dybas JM, Kulej K, Reyes ED, Price AM, Akhtar LN, Orr A, Garcia BA, Boutell C, Weitzman MD (2021) Comparative proteomics identifies Schlafen 5 (SLFN5) as a herpes simplex virus restriction factor that suppresses viral transcription. Nat Microbiol 6:234-245 doi:10.1038/s41564-020-00826-3

112. Kim H, Hwang JS, Lee B, Hong J, Lee S (2014) Newly Identified Cancer-Associated Role of Human Neuronal Growth Regulator 1 (NEGR1). J Cancer 5:598-608 doi:10.7150/jca.8052

113. Knutsen E, Lellahi SM, Aure MR, Nord S, Fismen S, Larsen KB, Gabriel MT, Hedberg A, Bjorklund SS, Oslo Breast Cancer Research C, Bofin AM, Maelandsmo GM, Sorlie T, Mortensen ES, Perander M (2020) The expression of the long NEAT1_2 isoform is associated with human epidermal growth factor receptor 2-positive breast cancers. Sci Rep 10:1277 doi:10.1038/s41598-020-57759-4

114. Korbecki J, Siminska D, Kojder K, Grochans S, Gutowska I, Chlubek D, Baranowska-Bosiacka I (2020) Fractalkine/CX3CL1 in Neoplastic Processes. Int J Mol Sci 21 doi:10.3390/ijms21103723

115. Kotwica-Mojzych K, Jodlowska-Jedrych B, Mojzych M (2021) CD200:CD200R Interactions and Their Importance in Immunoregulation. Int J Mol Sci 22 doi:10.3390/ijms22041602

116. Kraler S, Wenzl FA, Georgiopoulos G, Obeid S, Liberale L, von Eckardstein A, Muller O, Mach F, Raber L, Losdat S, Schmiady MO, Stellos K, Stamatelopoulos K, Camici GG, Srdic A, Paneni F, Akhmedov A, Luscher TF (2022) Soluble lectin-like oxidized low-density lipoprotein receptor-1 predicts premature death in acute coronary syndromes. Eur Heart J doi:10.1093/eurheartj/ehac143

117. Kuhn CD, Wilusz JE, Zheng Y, Beal PA, Joshua-Tor L (2015) On-enzyme refolding permits small RNA and tRNA surveillance by the CCA-adding enzyme. Cell 160:644-658 doi:10.1016/j.cell.2015.01.005

118. Lancellotti P, Marechal P, Donis N, Oury C (2019) Inflammation, cardiovascular disease, and cancer: a common link with far-reaching implications. Eur Heart J 40:3910-3912 doi:10.1093/eurheartj/ehz645

119. Lewis Marffy AL, McCarthy AJ (2020) Leukocyte Immunoglobulin-Like Receptors (LILRs) on Human Neutrophils: Modulators of Infection and Immunity. Front Immunol 11:857 doi:10.3389/fimmu.2020.00857

120. Ley K, Pramod AB, Croft M, Ravichandran KS, Ting JP (2016) How Mouse Macrophages Sense What Is Going On. Front Immunol 7:204 doi:10.3389/fimmu.2016.00204

121. Li H, Kniep E, Emmendorffer A, Lohmann-Matthes ML (1991) Differentiation of macrophage precursors to cells with LAK activity under the influence of CSF-1 and high dose IL-2. Scand J Immunol 33:511-520 doi:10.1111/j.1365-3083.1991.tb02521.x

122. Li H, Pohler U, Strehlow I, Hertig S, Baccarini M, Emmendorffer A, Tschopp J, Lohmann-Matthes ML (1994) Macrophage precursor cells produce perforin and perform Yac-1 lytic activity in response to stimulation with interleukin-2. J Leukoc Biol 56:117-123 doi:10.1002/jlb.56.2.117

123. Li H, Schwinzer R, Baccarini M, Lohmann-Matthes ML (1989) Cooperative effects of colony-stimulating factor 1 and recombinant interleukin 2 on proliferation and induction of cytotoxicity of macrophage precursors generated from mouse bone marrow cell cultures. J Exp Med 169:973-986 doi:10.1084/jem.169.3.973

124. Li M, Kao E, Gao X, Sandig H, Limmer K, Pavon-Eternod M, Jones TE, Landry S, Pan T, Weitzman MD, David M (2012) Codon-usage-based inhibition of HIV protein synthesis by human schlafen 11. Nature 491:125-128 doi:10.1038/nature11433

125. Li X, Xiong X, Zhang M, Wang K, Chen Y, Zhou J, Mao Y, Lv J, Yi D, Chen XW, Wang C, Qian SB, Yi C (2017) Base-Resolution Mapping Reveals Distinct m(1)A Methylome in Nuclear- and Mitochondrial-Encoded Transcripts. Mol Cell 68:993-1005 e1009 doi:10.1016/j.molcel.2017.10.019

126. Li X, Ye S, Lu Y (2020) Long non-coding RNA NEAT1 overexpression associates with increased exacerbation risk, severity, and inflammation, as well as decreased lung function through the interaction with microRNA-124 in asthma. J Clin Lab Anal 34:e23023 doi:10.1002/jcla.23023

127. Liang Q, Su L, Zhang D, Jiao J (2020) CD93 negatively regulates astrogenesis in response to MMRN2 through the transcriptional repressor ZFP503 in the developing brain. Proc Natl Acad Sci U S A 117:9413-9422 doi:10.1073/pnas.1922713117

128. Libby P (2015) Fanning the flames: inflammation in cardiovascular diseases. Cardiovasc Res 107:307-309 doi:10.1093/cvr/cvv188

129. Lino Cardenas CL, Kessinger CW, Cheng Y, MacDonald C, MacGillivray T, Ghoshhajra B, Huleihel L, Nuri S, Yeri AS, Jaffer FA, Kaminski N, Ellinor P, Weintraub NL, Malhotra R, Isselbacher EM, Lindsay ME (2018) An HDAC9-MALAT1-BRG1 complex mediates smooth muscle dysfunction in thoracic aortic aneurysm. Nat Commun 9:1009 doi:10.1038/s41467-018-03394-7

130. Lino Cardenas CL, Kessinger CW, Chou EL, Ghoshhajra B, Yeri AS, Das S, Weintraub NL, Malhotra R, Jaffer FA, Lindsay ME (2019) HDAC9 complex inhibition improves smooth muscle-dependent stenotic vascular disease. JCI Insight 4 doi:10.1172/jci.insight.124706

131. Little FF, de Bie J, van Oosterhout A, Kornfeld H, Center DM, Cruikshank WW (2003) Immunomodulatory effect of interleukin-16 on allergic airway inflammation. Chest 123:431S-432S doi:10.1378/chest.123.3_suppl.431s

132. Liu F, Zhou P, Wang Q, Zhang M, Li D (2018) The Schlafen family: complex roles in different cell types and virus replication. Cell Biol Int 42:2-8 doi:10.1002/cbin.10778

133. Liu HQ, Zhang XY, Edfeldt K, Nijhuis MO, Idborg H, Back M, Roy J, Hedin U, Jakobsson PJ, Laman JD, de Kleijn DP, Pasterkamp G, Hansson GK, Yan ZQ (2013) NOD2-mediated innate immune signaling regulates the eicosanoids in atherosclerosis. Arterioscler Thromb Vasc Biol 33:2193-2201 doi:10.1161/ATVBAHA.113.301715

134. Liu J, Chen SJ, Hsu SW, Zhang J, Li JM, Yang DC, Gu S, Pinkerton KE, Chen CH (2021) MARCKS cooperates with NKAP to activate NF-kB signaling in smoke-related lung cancer. Theranostics 11:4122-4136 doi:10.7150/thno.53558

135. Liu JQ, Hu A, Zhu J, Yu J, Talebian F, Bai XF (2020) CD200-CD200R Pathway in the Regulation of Tumor Immune Microenvironment and Immunotherapy. Adv Exp Med Biol 1223:155-165 doi:10.1007/978-3-030-35582-1_8

136. Liu S, Lei Z, Li J, Wang L, Jia R, Liu Z, Jiang C, Gao Y, Liu M, Kuang L, Qian Z, Zhou D, Speck SH, Liang X (2020) Interleukin 16 contributes to gammaherpesvirus pathogenesis by inhibiting viral reactivation. PLoS Pathog 16:e1008701 doi:10.1371/journal.ppat.1008701

137. Liu X, Liu J, Zhao S, Zhang H, Cai W, Cai M, Ji X, Leak RK, Gao Y, Chen J, Hu X (2016) Interleukin-4 Is Essential for Microglia/Macrophage M2 Polarization and Long-Term Recovery After Cerebral Ischemia. Stroke 47:498-504 doi:10.1161/STROKEAHA.115.012079

138. Liu Y, Given KS, Dickson EL, Owens GP, Macklin WB, Bennett JL (2019) Concentration-dependent effects of CSF1R inhibitors on oligodendrocyte progenitor cells ex vivo and in vivo. Exp Neurol 318:32-41 doi:10.1016/j.expneurol.2019.04.011

139. Liu Y, Yang H, Liu LX, Yan W, Guo HJ, Li WJ, Tian C, Li HH, Wang HX (2016) NOD2 contributes to myocardial ischemia/reperfusion injury by regulating cardiomyocyte apoptosis and inflammation. Life Sci 149:10-17 doi:10.1016/j.lfs.2016.02.039

140. Lohmann-Matthes ML, Emmendoerffer A, Hao L (1991) Influence of interleukin-2 on the differentiation of macrophages. Pathobiology 59:117-121 doi:10.1159/000163627

141. Lu X, Huang J, Wu S, Zheng Q, Liu P, Feng H, Su X, Fu H, Xi Q, Wang G (2020) The tRNA-like small noncoding RNA mascRNA promotes global protein translation. EMBO Rep 21:e49684 doi:10.15252/embr.201949684

142. Luo Y, Duan H, Qian Y, Feng L, Wu Z, Wang F, Feng J, Yang D, Qin Z, Yan X (2017) Macrophagic CD146 promotes foam cell formation and retention during atherosclerosis. Cell Res 27:352-372 doi:10.1038/cr.2017.8

143. Lyons SM, Fay MM, Akiyama Y, Anderson PJ, Ivanov P (2017) RNA biology of angiogenin: Current state and perspectives. RNA Biol 14:171-178 doi:10.1080/15476286.2016.1272746

144. Lyu Q, Xu S, Lyu Y, Choi M, Christie CK, Slivano OJ, Rahman A, Jin ZG, Long X, Xu Y, Miano JM (2019) SENCR stabilizes vascular endothelial cell adherens junctions through interaction with CKAP4. Proc Natl Acad Sci U S A 116:546-555 doi:10.1073/pnas.1810729116

145. Ma M, Hui J, Zhang QY, Zhu Y, He Y, Liu XJ (2018) Long non-coding RNA nuclear-enriched abundant transcript 1 inhibition blunts myocardial ischemia reperfusion injury via autophagic flux arrest and apoptosis in streptozotocin-induced diabetic rats. Atherosclerosis 277:113-122 doi:10.1016/j.atherosclerosis.2018.08.031

146. Machyna M, Kiefer L, Simon MD (2020) Enhanced nucleotide chemistry and toehold nanotechnology reveals lncRNA spreading on chromatin. Nat Struct Mol Biol 27:297-304 doi:10.1038/s41594-020-0390-z

147. Malinauskas T, Peer TV, Bishop B, Mueller TD, Siebold C (2020) Repulsive guidance molecules lock growth differentiation factor 5 in an inhibitory complex. Proc Natl Acad Sci U S A 117:15620-15631 doi:10.1073/pnas.2000561117

148. Marino F, Tozzi M, Schembri L, Ferraro S, Tarallo A, Scanzano A, Legnaro M, Castelli P, Cosentino M (2015) Production of IL-8, VEGF and Elastase by Circulating and Intraplaque Neutrophils in Patients with Carotid Atherosclerosis. PLoS One 10:e0124565 doi:10.1371/journal.pone.0124565

149. Matsui K (1999) Role of interleukin-2 receptor expression on macrophages from Salmonella-infected mice. FEMS Immunol Med Microbiol 24:97-103 doi:10.1111/j.1574-695X.1999.tb01270.x

150. Matsumoto S, Yamamichi T, Shinzawa K, Kasahara Y, Nojima S, Kodama T, Obika S, Takehara T, Morii E, Okuyama H, Kikuchi A (2019) GREB1 induced by Wnt signaling promotes development of hepatoblastoma by suppressing TGFbeta signaling. Nat Commun 10:3882 doi:10.1038/s41467-019-11533-x

151. Mavrommatis E, Fish EN, Platanias LC (2013) The schlafen family of proteins and their regulation by interferons. J Interferon Cytokine Res 33:206-210 doi:10.1089/jir.2012.0133

152. McCormack R, Podack ER (2015) Perforin-2/Mpeg1 and other pore-forming proteins throughout evolution. J Leukoc Biol 98:761-768 doi:10.1189/jlb.4MR1114-523RR

153. Mellett M (2020) Regulation and dysregulation of CARD14 signalling and its physiological consequences in inflammatory skin disease. Cell Immunol 354:104147 doi:10.1016/j.cellimm.2020.104147

154. Metzner FJ, Huber E, Hopfner KP, Lammens K (2022) Structural and biochemical characterization of human Schlafen 5. Nucleic Acids Res 50:1147-1161 doi:10.1093/nar/gkab1278

155. Mickeviciute GC, Valiuskyte M, Platten M, Wszolek ZK, Andersen O, Danylaite Karrenbauer V, Ineichen BV, Granberg T (2021) Neuroimaging phenotypes of CSF1R-related leukoencephalopathy: Systematic review, meta-analysis, and imaging recommendations. J Intern Med doi:10.1111/joim.13420

156. Mikulska M, Lanini S, Gudiol C, Drgona L, Ippolito G, Fernandez-Ruiz M, Salzberger B (2018) ESCMID Study Group for Infections in Compromised Hosts (ESGICH) Consensus Document on the safety of targeted and biological therapies: an infectious diseases perspective (Agents targeting lymphoid cells surface antigens [I]: CD19, CD20 and CD52). Clin Microbiol Infect 24 Suppl 2:S71-S82 doi:10.1016/j.cmi.2018.02.003

157. Modic M, Grosch M, Rot G, Schirge S, Lepko T, Yamazaki T, Lee FCY, Rusha E, Shaposhnikov D, Palo M, Merl-Pham J, Cacchiarelli D, Rogelj B, Hauck SM, von Mering C, Meissner A, Lickert H, Hirose T, Ule J, Drukker M (2019) Cross-Regulation between TDP-43 and Paraspeckles Promotes Pluripotency-Differentiation Transition. Mol Cell 74:951-965 e913 doi:10.1016/j.molcel.2019.03.041

158. Moreno Velasquez I, Gajulapuri A, Leander K, Berglund A, de Faire U, Gigante B (2019) Serum IL8 is not associated with cardiovascular events but with all-cause mortality. BMC Cardiovasc Disord 19:34 doi:10.1186/s12872-019-1014-6

159. Moss WN (2018) RNA2DMut: a web tool for the design and analysis of RNA structure mutations. RNA 24:273-286 doi:10.1261/rna.063933.117

160. Murugaiah V, Varghese PM, Beirag N, De Cordova S, Sim RB, Kishore U (2021) Complement Proteins as Soluble Pattern Recognition Receptors for Pathogenic Viruses. Viruses 13 doi:10.3390/v13050824

161. Musunuru K (2017) Genome Editing: The Recent History and Perspective in Cardiovascular Diseases. J Am Coll Cardiol 70:2808-2821 doi:10.1016/j.jacc.2017.10.002

162. Naganuma T, Nakagawa S, Tanigawa A, Sasaki YF, Goshima N, Hirose T (2012) Alternative 3'-end processing of long noncoding RNA initiates construction of nuclear paraspeckles. EMBO J 31:4020-4034 doi:10.1038/emboj.2012.251

163. Nakagawa S, Ip JY, Shioi G, Tripathi V, Zong X, Hirose T, Prasanth KV (2012) Malat1 is not an essential component of nuclear speckles in mice. RNA 18:1487-1499 doi:10.1261/rna.033217.112

164. Nakagawa S, Shimada M, Yanaka K, Mito M, Arai T, Takahashi E, Fujita Y, Fujimori T, Standaert L, Marine JC, Hirose T (2014) The lncRNA Neat1 is required for corpus luteum formation and the establishment of pregnancy in a subpopulation of mice. Development 141:4618-4627 doi:10.1242/dev.110544

165. Nakagawa S, Yamazaki T, Hirose T (2018) Molecular dissection of nuclear paraspeckles: towards understanding the emerging world of the RNP milieu. Open Biol 8 doi:10.1098/rsob.180150

166. Nativel B, Ramin-Mangata S, Mevizou R, Figuester A, Andries J, Iwema T, Ikewaki N, Gasque P, Viranaicken W (2019) CD93 is a cell surface lectin receptor involved in the control of the inflammatory response stimulated by exogenous DNA. Immunology 158:85-93 doi:10.1111/imm.13100

167. Negroni A, Pierdomenico M, Cucchiara S, Stronati L (2018) NOD2 and inflammation: current insights. J Inflamm Res 11:49-60 doi:10.2147/JIR.S137606

168. Nelson BJ, Danielpour D, Rossio JL, Turpin J, Nacy CA (1994) Interleukin-2 suppresses activated macrophage intracellular killing activity by inducing macrophages to secrete TGF-beta. J Leukoc Biol 55:81-90 doi:10.1002/jlb.55.1.81

169. Nimmerjahn F, Ravetch JV (2008) Fcgamma receptors as regulators of immune responses. Nat Rev Immunol 8:34-47 doi:10.1038/nri2206

170. Norsworthy PJ, Fossati-Jimack L, Cortes-Hernandez J, Taylor PR, Bygrave AE, Thompson RD, Nourshargh S, Walport MJ, Botto M (2004) Murine CD93 (C1qRp) contributes to the removal of apoptotic cells in vivo but is not required for C1q-mediated enhancement of phagocytosis. J Immunol 172:3406-3414 doi:10.4049/jimmunol.172.6.3406

171. Oosting M, Cheng SC, Bolscher JM, Vestering-Stenger R, Plantinga TS, Verschueren IC, Arts P, Garritsen A, van Eenennaam H, Sturm P, Kullberg BJ, Hoischen A, Adema GJ, van der Meer JW, Netea MG, Joosten LA (2014) Human TLR10 is an anti-inflammatory pattern-recognition receptor. Proc Natl Acad Sci U S A 111:E4478-4484 doi:10.1073/pnas.1410293111

172. Otsuka F, Zhao X, Trout HH, Qiao Y, Wasserman BA, Nakano M, Macphee CH, Brandt M, Krug-Gourley S, Guo L, Ladich ER, Cheng Q, Davis HR, Finn AV, Virmani R, Kolodgie FD (2017) Community-based statins and advanced carotid plaque: Role of CD163 positive macrophages in lipoprotein-associated phospholipase A2 activity in atherosclerotic plaque. Atherosclerosis 267:78-89 doi:10.1016/j.atherosclerosis.2017.10.014

173. Ozaki K, Leonard WJ (2002) Cytokine and cytokine receptor pleiotropy and redundancy. J Biol Chem 277:29355-29358 doi:10.1074/jbc.R200003200

174. Panigrahy D, Gartung A, Yang J, Yang H, Gilligan MM, Sulciner ML, Bhasin SS, Bielenberg DR, Chang J, Schmidt BA, Piwowarski J, Fishbein A, Soler-Ferran D, Sparks MA, Staffa SJ, Sukhatme V, Hammock BD, Kieran MW, Huang S, Bhasin M, Serhan CN,

Sukhatme VP (2019) Preoperative stimulation of resolution and inflammation blockade eradicates micrometastases. J Clin Invest 129:2964-2979 doi:10.1172/JCI127282

175. Pascoal Ramos MI, Kesmir C, Stok JE, Geerdink R, Satravelas N, Westerlaken GHA, Meyaard L, van der Vlist M (2022) CD200R1L is a functional evolutionary conserved activating receptor in human neutrophils. J Leukoc Biol 111:367-377 doi:10.1002/JLB.2A0520-334R

176. Pereira M, Ribeiro DR, Pinheiro MM, Ferreira M, Kellner S, Soares AR (2021) m(5)U54 tRNA Hypomodification by Lack of TRMT2A Drives the Generation of tRNA-Derived Small RNAs. Int J Mol Sci 22 doi:10.3390/ijms22062941

177. Pickar-Oliver A, Gersbach CA (2019) The next generation of CRISPR-Cas technologies and applications. Nat Rev Mol Cell Biol 20:490-507 doi:10.1038/s41580-019-0131-5

178. Poderoso T, De la Riva PM, Alvarez B, Dominguez J, Ezquerra A, Revilla C (2022) CD200R family receptors are expressed on porcine monocytes and modulate the production of IL-8 and TNF-alpha triggered by TLR4 or TLR7 in these cells. Mol Immunol 144:166-177 doi:10.1016/j.molimm.2022.02.019

179. Poller W, Dimmeler S, Heymans S, Zeller T, Haas J, Karakas M, Leistner DM, Jakob P, Nakagawa S, Blankenberg S, Engelhardt S, Thum T, Weber C, Meder B, Hajjar R, Landmesser U (2018) Non-coding RNAs in cardiovascular diseases: diagnostic and therapeutic perspectives. Eur Heart J 39:2704-2716 doi:10.1093/eurheartj/ehx165

180. Prabhudas M, Bowdish D, Drickamer K, Febbraio M, Herz J, Kobzik L, Krieger M, Loike J, Means TK, Moestrup SK, Post S, Sawamura T, Silverstein S, Wang XY, El Khoury J (2014) Standardizing scavenger receptor nomenclature. J Immunol 192:1997-2006 doi:10.4049/jimmunol.1490003

181. Prehn JHM, Jirstrom E (2020) Angiogenin and tRNA fragments in Parkinson's disease and neurodegeneration. Acta Pharmacol Sin 41:442-446 doi:10.1038/s41401-020-0375-9

182. Puck A, Aigner R, Modak M, Cejka P, Blaas D, Stockl J (2015) Expression and regulation of Schlafen (SLFN) family members in primary human monocytes, monocyte-derived dendritic cells and T cells. Results Immunol 5:23-32 doi:10.1016/j.rinim.2015.10.001

183. Pyonteck SM, Akkari L, Schuhmacher AJ, Bowman RL, Sevenich L, Quail DF, Olson OC, Quick ML, Huse JT, Teijeiro V, Setty M, Leslie CS, Oei Y, Pedraza A, Zhang J, Brennan CW, Sutton JC, Holland EC, Daniel D, Joyce JA (2013) CSF-1R inhibition alters macrophage polarization and blocks glioma progression. Nat Med 19:1264-1272 doi:10.1038/nm.3337

184. Quinodoz SA, Ollikainen N, Tabak B, Palla A, Schmidt JM, Detmar E, Lai MM, Shishkin AA, Bhat P, Takei Y, Trinh V, Aznauryan E, Russell P, Cheng C, Jovanovic M, Chow A, Cai L, McDonel P, Garber M, Guttman M (2018) Higher-Order Inter-chromosomal Hubs Shape 3D Genome Organization in the Nucleus. Cell 174:744-757 e724 doi:10.1016/j.cell.2018.05.024

185. Raggi F, Pelassa S, Pierobon D, Penco F, Gattorno M, Novelli F, Eva A, Varesio L, Giovarelli M, Bosco MC (2017) Regulation of Human Macrophage M1-M2 Polarization Balance by Hypoxia and the Triggering Receptor Expressed on Myeloid Cells-1. Front Immunol 8:1097 doi:10.3389/fimmu.2017.01097

186. Ramesh A, Kumar S, Nandi D, Kulkarni A (2019) CSF1R- and SHP2-Inhibitor-Loaded Nanoparticles Enhance Cytotoxic Activity and Phagocytosis in Tumor-Associated Macrophages. Adv Mater 31:e1904364 doi:10.1002/adma.201904364

187. Rashad S, Niizuma K, Tominaga T (2020) tRNA cleavage: a new insight. Neural Regen Res 15:47-52 doi:10.4103/1673-5374.264447

188. Remmerie A, Scott CL (2018) Macrophages and lipid metabolism. Cell Immunol 330:27-42 doi:10.1016/j.cellimm.2018.01.020

189. Roberts O, Paraoan L (2020) PERP-ing into diverse mechanisms of cancer pathogenesis: Regulation and role of the p53/p63 effector PERP. Biochim Biophys Acta Rev Cancer 1874:188393 doi:10.1016/j.bbcan.2020.188393

190. Rojo R, Raper A, Ozdemir DD, Lefevre L, Grabert K, Wollscheid-Lengeling E, Bradford B, Caruso M, Gazova I, Sanchez A, Lisowski ZM, Alves J, Molina-Gonzalez I, Davtyan H, Lodge RJ, Glover JD, Wallace R, Munro DAD, David E, Amit I, Miron VE, Priller J, Jenkins SJ, Hardingham GE, Blurton-Jones M, Mabbott NA, Summers KM, Hohenstein P, Hume DA, Pridans C (2019) Deletion of a Csf1r enhancer selectively impacts CSF1R expression and development of tissue macrophage populations. Nat Commun 10:3215 doi:10.1038/s41467-019-11053-8

191. Ruan Z, Wang S, Yu W, Deng F (2019) LncRNA NEAT1 aggravates diabetic myocardial ischemia-reperfusion injury through regulating PINK1 by targeting miR-27b. Int J Cardiol 286:136 doi:10.1016/j.ijcard.2019.03.046

192. Sadik A, Somarribas Patterson LF, Ozturk S, Mohapatra SR, Panitz V, Secker PF, Pfander P, Loth S, Salem H, Prentzell MT, Berdel B, Iskar M, Faessler E, Reuter F, Kirst I, Kalter V, Foerster KI, Jager E, Guevara CR, Sobeh M, Hielscher T, Poschet G, Reinhardt A, Hassel JC, Zapatka M, Hahn U, von Deimling A, Hopf C, Schlichting R, Escher BI, Burhenne J, Haefeli WE, Ishaque N, Bohme A, Schauble S, Thedieck K, Trump S, Seiffert M, Opitz CA (2020) IL4I1 Is a Metabolic Immune Checkpoint that Activates the AHR and Promotes Tumor Progression. Cell 182:1252-1270 e1234 doi:10.1016/j.cell.2020.07.038

193. Sarangdhar MA, Allam R (2021) Angiogenin (ANG)-Ribonuclease Inhibitor (RNH1) System in Protein Synthesis and Disease. Int J Mol Sci 22 doi:10.3390/ijms22031287

194. Scheibenbogen C, Keilholz U, Richter M, Andreesen R, Hunstein W (1992) The interleukin-2 receptor in human monocytes and macrophages: regulation of expression and release of the alpha and beta chains (p55 and p75). Res Immunol 143:33-37 doi:10.1016/0923-2494(92)80077-x

195. Schmall A, Al-Tamari HM, Herold S, Kampschulte M, Weigert A, Wietelmann A, Vipotnik N, Grimminger F, Seeger W, Pullamsetti SS, Savai R (2015) Macrophage and cancer cell cross-talk via CCR2 and CX3CR1 is a fundamental mechanism driving lung cancer. Am J Respir Crit Care Med 191:437-447 doi:10.1164/rccm.201406-1137OC

196. Schnappauf O, Chae JJ, Kastner DL, Aksentijevich I (2019) The Pyrin Inflammasome in Health and Disease. Front Immunol 10:1745 doi:10.3389/fimmu.2019.01745

197. Schulman IG (2017) Liver X receptors link lipid metabolism and inflammation. FEBS Lett 591:2978-2991 doi:10.1002/1873-3468.12702

198. Schwarz DA, Katayama CD, Hedrick SM (1998) Schlafen, a new family of growth regulatory genes that affect thymocyte development. Immunity 9:657-668 doi:10.1016/s1074-7613(00)80663-9

199. Seizer P, Schiemann S, Merz T, Daub K, Bigalke B, Stellos K, Muller I, Stockle C, Muller K, Gawaz M, May AE (2010) CD36 and macrophage scavenger receptor a modulate foam cell formation via inhibition of lipid-laden platelet phagocytosis. Semin Thromb Hemost 36:157-162 doi:10.1055/s-0030-1251499

200. Sheng J, Xu Z (2016) Three decades of research on angiogenin: a review and perspective. Acta Biochim Biophys Sin (Shanghai) 48:399-410 doi:10.1093/abbs/gmv131

201. Shiga T, Nozaki Y, Tomita D, Kishimoto K, Hirooka Y, Kinoshita K, Funauchi M, Matsumura I (2021) Usefulness of Interleukin-18 as a Diagnostic Biomarker to Differentiate Adult-Onset Still's Disease With/Without Macrophage Activation Syndrome From Other Secondary Hemophagocytic Lymphohistiocytosis in Adults. Front Immunol 12:750114 doi:10.3389/fimmu.2021.750114

202. Shin SP, Goh AR, Ju JM, Kang HG, Kim SJ, Kim JK, Park EJ, Bae YS, Choi K, Jung YS, Lee SJ (2021) Local adenoviral delivery of soluble CD200R-Ig enhances antitumor immunity by inhibiting CD200-beta-catenin-driven M2 macrophage. Mol Ther Oncolytics 23:138-150 doi:10.1016/j.omto.2021.09.001

203. Shingai Y, Yokota T, Okuzaki D, Sudo T, Ishibashi T, Doi Y, Ueda T, Ozawa T, Nakai R, Tanimura A, Ichii M, Shibayama H, Kanakura Y, Hosen N (2021) Autonomous TGFbeta signaling induces phenotypic variation in human acute myeloid leukemia. Stem Cells 39:723-736 doi:10.1002/stem.3348

204. Simchovitz A, Hanan M, Niederhoffer N, Madrer N, Yayon N, Bennett ER, Greenberg DS, Kadener S, Soreq H (2019) NEAT1 is overexpressed in Parkinson's disease substantia nigra and confers drug-inducible neuroprotection from oxidative stress. FASEB J 33:11223-11234 doi:10.1096/fj.201900830R

205. Singh K, Loreth D, Pottker B, Hefti K, Innos J, Schwald K, Hengstler H, Menzel L, Sommer CJ, Radyushkin K, Kretz O, Philips MA, Haas CA, Frauenknecht K, Lillevali K, Heimrich B, Vasar E, Schafer MKE (2018) Neuronal Growth and Behavioral Alterations in Mice Deficient for the Psychiatric Disease-Associated Negr1 Gene. Front Mol Neurosci 11:30 doi:10.3389/fnmol.2018.00030

206. Sjaarda J, Gerstein H, Chong M, Yusuf S, Meyre D, Anand SS, Hess S, Pare G (2018) Blood CSF1 and CXCL12 as Causal Mediators of Coronary Artery Disease. J Am Coll Cardiol 72:300-310 doi:10.1016/j.jacc.2018.04.067

207. Smith KP, Hall LL, Lawrence JB (2020) Nuclear hubs built on RNAs and clustered organization of the genome. Curr Opin Cell Biol 64:67-76 doi:10.1016/j.ceb.2020.02.015

208. Sohn WJ, Kim D, Lee KW, Kim MS, Kwon S, Lee Y, Kim DS, Kwon HJ (2007) Novel transcriptional regulation of the schlafen-2 gene in macrophages in response to TLR-triggered stimulation. Mol Immunol 44:3273-3282 doi:10.1016/j.molimm.2007.03.001

209. Stefanovic L, Stefanovic B (2012) Role of cytokine receptor-like factor 1 in hepatic stellate cells and fibrosis. World J Hepatol 4:356-364 doi:10.4254/wjh.v4.i12.356

210. Su Y, Yamazaki S, Morisue R, Suzuki J, Yoshikawa T, Nakatsura T, Tsuboi M, Ochiai A, Ishii G (2021) Tumor-Infiltrating T Cells Concurrently Overexpress CD200R with Immune Checkpoints PD-1, CTLA-4, and TIM-3 in Non-Small-Cell Lung Cancer. Pathobiology 88:218-227 doi:10.1159/000511557

211. Sun GD, Kobayashi T, Abe M, Tada N, Adachi H, Shiota A, Totsuka Y, Hino O (2007) The endoplasmic reticulum stress-inducible protein Niban regulates eIF2alpha and S6K1/4E-BP1 phosphorylation. Biochem Biophys Res Commun 360:181-187 doi:10.1016/j.bbrc.2007.06.021

212. Sun H, He X, Tao X, Hou T, Chen M, He M, Liao H (2020) The CD200/CD200R signaling pathway contributes to spontaneous functional recovery by enhancing synaptic plasticity after stroke. J Neuroinflammation 17:171 doi:10.1186/s12974-020-01845-x

213. Sun R, Hedl M, Abraham C (2019) Twist1 and Twist2 Induce Human Macrophage Memory upon Chronic Innate Receptor Treatment by HDAC-Mediated Deacetylation of Cytokine Promoters. J Immunol 202:3297-3308 doi:10.4049/jimmunol.1800757

214. Sun Y, Chen W, Torphy RJ, Yao S, Zhu G, Lin R, Lugano R, Miller EN, Fujiwara Y, Bian L, Zheng L, Anand S, Gao F, Zhang W, Ferrara SE, Goodspeed AE, Dimberg A, Wang XJ, Edil BH, Barnett CC, Schulick RD, Chen L, Zhu Y (2021) Blockade of the CD93 pathway

normalizes tumor vasculature to facilitate drug delivery and immunotherapy. Sci Transl Med 13 doi:10.1126/scitranslmed.abc8922

215. Sundaram B, Kanneganti TD (2021) Advances in Understanding Activation and Function of the NLRC4 Inflammasome. Int J Mol Sci 22 doi:10.3390/ijms22031048

216. Suzuki T, Yashiro Y, Kikuchi I, Ishigami Y, Saito H, Matsuzawa I, Okada S, Mito M, Iwasaki S, Ma D, Zhao X, Asano K, Lin H, Kirino Y, Sakaguchi Y, Suzuki T (2020) Complete chemical structures of human mitochondrial tRNAs. Nat Commun 11:4269 doi:10.1038/s41467-020-18068-6

217. Suzuki Y, Shirai M, Asada K, Yasui H, Karayama M, Hozumi H, Furuhashi K, Enomoto N, Fujisawa T, Nakamura Y, Inui N, Shirai T, Hayakawa H, Suda T (2018) Macrophage mannose receptor, CD206, predict prognosis in patients with pulmonary tuberculosis. Sci Rep 8:13129 doi:10.1038/s41598-018-31565-5

218. Swaminathan K, Campbell A, Papalazarou V, Jaber-Hijazi F, Nixon C, McGhee E, Strathdee D, Sansom OJ, Machesky LM (2021) The RAC1 Target NCKAP1 Plays a Crucial Role in the Progression of Braf;Pten-Driven Melanoma in Mice. J Invest Dermatol 141:628-637 e615 doi:10.1016/j.jid.2020.06.029

219. Swanson KV, Deng M, Ting JP (2019) The NLRP3 inflammasome: molecular activation and regulation to therapeutics. Nat Rev Immunol 19:477-489 doi:10.1038/s41577-019-0165-0

220. Szomjak E, Der H, Kerekes G, Veres K, Csiba L, Toth J, Peter M, Soltesz P, Szodoray P (2010) Immunological parameters, including CXCL8 (IL-8) characterize cerebro- and cardiovascular events in patients with peripheral artery diseases. Scand J Immunol 71:283-291 doi:10.1111/j.1365-3083.2010.02368.x

221. Takai T, Ono M, Hikida M, Ohmori H, Ravetch JV (1996) Augmented humoral and anaphylactic responses in Fc gamma RII-deficient mice. Nature 379:346-349 doi:10.1038/379346a0

222. Takenaka M, Yabuta A, Takahashi Y, Takakura Y (2021) Interleukin-4-carrying small extracellular vesicles with a high potential as anti-inflammatory therapeutics based on modulation of macrophage function. Biomaterials 278:121160 doi:10.1016/j.biomaterials.2021.121160

223. Teng Y, Qin H, Bahassan A, Bendzunas NG, Kennedy EJ, Cowell JK (2016) The WASF3-NCKAP1-CYFIP1 Complex Is Essential for Breast Cancer Metastasis. Cancer Res 76:5133-5142 doi:10.1158/0008-5472.CAN-16-0562

224. Thornton S, Tan R, Sproles A, Do T, Schick J, Grom AA, DeLay M, Schulert GS (2019) A Multiparameter Flow Cytometry Analysis Panel to Assess CD163 mRNA and Protein in Monocyte and Macrophage Populations in Hyperinflammatory Diseases. J Immunol 202:1635-1643 doi:10.4049/jimmunol.1800765

225. Tosi GM, Caldi E, Parolini B, Toti P, Neri G, Nardi F, Traversi C, Cevenini G, Marigliani D, Nuti E, Bacci T, Galvagni F, Orlandini M (2017) CD93 as a Potential Target in Neovascular Age-Related Macular Degeneration. J Cell Physiol 232:1767-1773 doi:10.1002/jcp.25689

226. Tremblay M, Sanchez-Ferras O, Bouchard M (2018) GATA transcription factors in development and disease. Development 145 doi:10.1242/dev.164384

227. Trindade BC, Chen GY (2020) NOD1 and NOD2 in inflammatory and infectious diseases. Immunol Rev 297:139-161 doi:10.1111/imr.12902

228. Ushach I, Zlotnik A (2016) Biological role of granulocyte macrophage colony-stimulating factor (GM-CSF) and macrophage colony-stimulating factor (M-CSF) on cells of the myeloid lineage. J Leukoc Biol 100:481-489 doi:10.1189/jlb.3RU0316-144R

229. Van't Klooster CC, Ridker PM, Hjortnaes J, van der Graaf Y, Asselbergs FW, Westerink J, Aerts J, Visseren FLJ (2019) The relation between systemic inflammation and incident cancer in patients with stable cardiovascular disease: a cohort study. Eur Heart J 40:3901-3909 doi:10.1093/eurheartj/ehz587

230. Van Haute L, Lee SY, McCann BJ, Powell CA, Bansal D, Vasiliauskaite L, Garone C, Shin S, Kim JS, Frye M, Gleeson JG, Miska EA, Rhee HW, Minczuk M (2019) NSUN2 introduces 5-methylcytosines in mammalian mitochondrial tRNAs. Nucleic Acids Res 47:8720-8733 doi:10.1093/nar/gkz559

231. Velasquez IM, Frumento P, Johansson K, Berglund A, de Faire U, Leander K, Gigante B (2014) Association of interleukin 8 with myocardial infarction: results from the Stockholm Heart Epidemiology Program. Int J Cardiol 172:173-178 doi:10.1016/j.ijcard.2013.12.170

232. Vitalle J, Terren I, Orrantia A, Bilbao A, Gamboa PM, Borrego F, Zenarruzabeitia O (2020) The Expression and Function of CD300 Molecules in the Main Players of Allergic Responses: Mast Cells, Basophils and Eosinophils. Int J Mol Sci 21 doi:10.3390/ijms21093173

233. Vitalle J, Terren I, Orrantia A, Zenarruzabeitia O, Borrego F (2019) CD300 receptor family in viral infections. Eur J Immunol 49:364-374 doi:10.1002/eji.201847951

234. Vogel A, Brunner JS, Hajto A, Sharif O, Schabbauer G (2022) Lipid scavenging macrophages and inflammation. Biochim Biophys Acta Mol Cell Biol Lipids 1867:159066 doi:10.1016/j.bbalip.2021.159066

235. Wang J, Li Y (2019) CD36 tango in cancer: signaling pathways and functions. Theranostics 9:4893-4908 doi:10.7150/thno.36037

236. Wang L, Liu Y, Yan S, Du T, Fu X, Gong X, Zhou X, Zhang T, Wang X (2020) Disease Progression-Dependent Expression of CD200R1 and CX3CR1 in Mouse Models of Parkinson's Disease. Aging Dis 11:254-268 doi:10.14336/AD.2019.0615

237. Wang Y, Hu SB, Wang MR, Yao RW, Wu D, Yang L, Chen LL (2018) Genome-wide screening of NEAT1 regulators reveals cross-regulation between paraspeckles and mitochondria. Nat Cell Biol 20:1145-1158 doi:10.1038/s41556-018-0204-2

238. Wang Z, Xu Q, Zhang N, Du X, Xu G, Yan X (2020) CD146, from a melanoma cell adhesion molecule to a signaling receptor. Signal Transduct Target Ther 5:148 doi:10.1038/s41392-020-00259-8

239. Wellner K, Czech A, Ignatova Z, Betat H, Morl M (2018) Examining tRNA 3'-ends in Escherichia coli: teamwork between CCA-adding enzyme, RNase T, and RNase R. RNA 24:361-370 doi:10.1261/rna.064436.117

240. West JA, Mito M, Kurosaka S, Takumi T, Tanegashima C, Chujo T, Yanaka K, Kingston RE, Hirose T, Bond C, Fox A, Nakagawa S (2016) Structural, super-resolution microscopy analysis of paraspeckle nuclear body organization. J Cell Biol 214:817-830 doi:10.1083/jcb.201601071

241. Wilusz JE (2015) Controlling translation via modulation of tRNA levels. Wiley Interdiscip Rev RNA 6:453-470 doi:10.1002/wrna.1287

242. Wilusz JE, Freier SM, Spector DL (2008) 3' end processing of a long nuclear-retained noncoding RNA yields a tRNA-like cytoplasmic RNA. Cell 135:919-932 doi:10.1016/j.cell.2008.10.012

243. Wilusz JE, Whipple JM, Phizicky EM, Sharp PA (2011) tRNAs marked with CCACCA are targeted for degradation. Science 334:817-821 doi:10.1126/science.1213671

244. Wu HJ, Tang GM, Shao PY, Zou HX, Shen WF, Huang MD, Pan HH, Zhai CL, Qian G (2019) Long non-coding RNA NEAT1 modulates hypoxia/reoxygenation-induced cardiomyocyte injury via targeting microRNA-520a. Exp Ther Med 18:2199-2206 doi:10.3892/etm.2019.7788

245. Xie SJ, Diao LT, Cai N, Zhang LT, Xiang S, Jia CC, Qiu DB, Liu C, Sun YJ, Lei H, Hou YR, Tao S, Hu YX, Xiao ZD, Zhang Q (2021) mascRNA and its parent lncRNA MALAT1 promote proliferation and metastasis of hepatocellular carcinoma cells by activating ERK/MAPK signaling pathway. Cell Death Discov 7:110 doi:10.1038/s41420-021-00497-x

246. Xiong Y, He L, Shay C, Lang L, Loveless J, Yu J, Chemmalakuzhy R, Jiang H, Liu M, Teng Y (2019) Nck-associated protein 1 associates with HSP90 to drive metastasis in human non-small-cell lung cancer. J Exp Clin Cancer Res 38:122 doi:10.1186/s13046-019-1124-0

247. Xu Y, Cao Z, Ding Y, Li Z, Xiang X, Lai R, Sheng Z, Liu Y, Cai W, Hu R, Wang H, Xie Q (2019) Long Non-coding RNA NEAT1 Alleviates Acute-on-Chronic Liver Failure Through Blocking TRAF6 Mediated Inflammatory Response. Front Physiol 10:1503 doi:10.3389/fphys.2019.01503

248. Xun Q, Wang Z, Hu X, Ding K, Lu X (2020) Small-Molecule CSF1R Inhibitors as Anticancer Agents. Curr Med Chem 27:3944-3966 doi:10.2174/1573394715666190618121649

249. Yamazaki T, Fujikawa C, Kubota A, Takahashi A, Hirose T (2018) CRISPRa-mediated NEAT1 lncRNA upregulation induces formation of intact paraspeckles. Biochem Biophys Res Commun 504:218-224 doi:10.1016/j.bbrc.2018.08.158

250. Yamazaki T, Souquere S, Chujo T, Kobelke S, Chong YS, Fox AH, Bond CS, Nakagawa S, Pierron G, Hirose T (2018) Functional Domains of NEAT1 Architectural lncRNA Induce Paraspeckle Assembly through Phase Separation. Mol Cell 70:1038-1053 e1037 doi:10.1016/j.molcel.2018.05.019

251. Yao Y, Xu XH, Jin L (2019) Macrophage Polarization in Physiological and Pathological Pregnancy. Front Immunol 10:792 doi:10.3389/fimmu.2019.00792

252. Yin S, Lu K, Tan T, Tang J, Wei J, Liu X, Hu X, Wan H, Huang W, Fan Y, Xie D, Yu Y (2020) Transcriptomic and open chromatin atlas of high-resolution anatomical regions in the rhesus macaque brain. Nat Commun 11:474 doi:10.1038/s41467-020-14368-z

253. Yoon T, Pyo JY, Ahn SS, Song JJ, Park YB, Lee SW (2020) Serum interleukin-16 significantly correlates with the Vasculitis Damage Index in antineutrophil cytoplasmic antibody-associated vasculitis. Arthritis Res Ther 22:73 doi:10.1186/s13075-020-02172-5

254. Youn JC, Yu HT, Jeon JW, Lee HS, Jang Y, Park YW, Park YB, Shin EC, Ha JW (2014) Soluble CD93 levels in patients with acute myocardial infarction and its implication on clinical outcome. PLoS One 9:e96538 doi:10.1371/journal.pone.0096538

255. Yu B, Wang S (2018) Angio-LncRs: LncRNAs that regulate angiogenesis and vascular disease. Theranostics 8:3654-3675 doi:10.7150/thno.26024

256. Yu Y, Wang Z, Zheng Q, Li J (2021) GREB1L overexpression correlates with prognosis and immune cell infiltration in lung adenocarcinoma. Sci Rep 11:13281 doi:10.1038/s41598-021-92695-x

257. Yuan H, Zelkha S, Burkatovskaya M, Gupte R, Leeman SE, Amar S (2013) Pivotal role of NOD2 in inflammatory processes affecting atherosclerosis and periodontal bone loss. Proc Natl Acad Sci U S A 110:E5059-5068 doi:10.1073/pnas.1320862110

258. Yuasa T, Kubo S, Yoshino T, Ujike A, Matsumura K, Ono M, Ravetch JV, Takai T (1999) Deletion of fcgamma receptor IIB renders H-2(b) mice susceptible to collagen-induced arthritis. J Exp Med 189:187-194 doi:10.1084/jem.189.1.187

259. Yue T, Zhan X, Zhang D, Jain R, Wang KW, Choi JH, Misawa T, Su L, Quan J, Hildebrand S, Xu D, Li X, Turer E, Sun L, Moresco EMY, Beutler B (2021) SLFN2 protection of tRNAs from stress-induced cleavage is essential for T cell-mediated immunity. Science 372 doi:10.1126/science.aba4220

260. Yue Y, Huang W, Liang J, Guo J, Ji J, Yao Y, Zheng M, Cai Z, Lu L, Wang J (2015) IL4I1 Is a Novel Regulator of M2 Macrophage Polarization That Can Inhibit T Cell Activation via L-Tryptophan and Arginine Depletion and IL-10 Production. PLoS One 10:e0142979 doi:10.1371/journal.pone.0142979

261. Zani IA, Stephen SL, Mughal NA, Russell D, Homer-Vanniasinkam S, Wheatcroft SB, Ponnambalam S (2015) Scavenger receptor structure and function in health and disease. Cells 4:178-201 doi:10.3390/cells4020178

262. Zhang B, Li B, Sun C, Tu T, Xiao Y, Liu Q (2021) Identification of key gene modules and pathways of human platelet transcriptome in acute myocardial infarction patients through co-expression network. Am J Transl Res 13:3890-3905

263. Zhang B, Mao YS, Diermeier SD, Novikova IV, Nawrocki EP, Jones TA, Lazar Z, Tung CS, Luo W, Eddy SR, Sanbonmatsu KY, Spector DL (2017) Identification and Characterization of a Class of MALAT1-like Genomic Loci. Cell Rep 19:1723-1738 doi:10.1016/j.celrep.2017.05.006

264. Zhang DW, Shao J, Lin J, Zhang N, Lu BJ, Lin SC, Dong MQ, Han J (2009) RIP3, an energy metabolism regulator that switches TNF-induced cell death from apoptosis to necrosis. Science 325:332-336 doi:10.1126/science.1172308

265. Zhang M, Zheng Y, Sun Y, Li S, Chen L, Jin X, Hou X, Liu X, Chen Q, Li J, Liu M, Zheng X, Zhang Y, Wu J, Yu B (2019) Knockdown of NEAT1 induces tolerogenic phenotype in dendritic cells by inhibiting activation of NLRP3 inflammasome. Theranostics 9:3425-3442 doi:10.7150/thno.33178

266. Zhang P, Cao L, Zhou R, Yang X, Wu M (2019) The lncRNA Neat1 promotes activation of inflammasomes in macrophages. Nat Commun 10:1495 doi:10.1038/s41467-019-09482-6

267. Zhang Q, Chao TC, Patil VS, Qin Y, Tiwari SK, Chiou J, Dobin A, Tsai CM, Li Z, Dang J, Gupta S, Urdahl K, Nizet V, Gingeras TR, Gaulton KJ, Rana TM (2019) The long noncoding RNA ROCKI regulates inflammatory gene expression. EMBO J 38 doi:10.15252/embj.2018100041

268. Zhang Q, Lian Z, Zhang W, Cui Y, Wang W, Wu J, Chen Z, Wang W (2019) Association between interleukin-8 gene -251 A/T polymorphism and the risk of coronary artery disease: A meta-analysis. Medicine (Baltimore) 98:e17866 doi:10.1097/MD.0000000000017866

269. Zhang X, Cui J, Qian H, Wang B, Yan F, Zhao Z (2020) CD200R Is Involved in the Anti-inflammatory Effect of Dexmedetomidine in Lipopolysaccharide-Stimulated Microglia. Inflammation 43:1707-1715 doi:10.1007/s10753-020-01244-7

270. Zhao G, Zhang H, Zhu S, Wang S, Zhu K, Zhao Y, Xu L, Zhang P, Xie J, Sun A, Zou Y, Ge J (2021) Interleukin-18 accelerates cardiac inflammation and dysfunction during ischemia/reperfusion injury by transcriptional activation of CXCL16. Cell Signal 87:110141 doi:10.1016/j.cellsig.2021.110141

271. Zhao Y, Su H, Shen X, Du J, Zhang X, Zhao Y (2017) The immunological function of CD52 and its targeting in organ transplantation. Inflamm Res 66:571-578 doi:10.1007/s00011-017-1032-8

272. Zheng D, Liwinski T, Elinav E (2020) Inflammasome activation and regulation: toward a better understanding of complex mechanisms. Cell Discov 6:36 doi:10.1038/s41421-020-0167-x

273. Zhou ZW, Zheng LJ, Ren X, Li AP, Zhou WS (2019) LncRNA NEAT1 facilitates survival and angiogenesis in oxygen-glucose deprivation (OGD)-induced brain microvascular endothelial cells (BMECs) via targeting miR-377 and upregulating SIRT1, VEGFA, and BCL-XL. Brain Res 1707:90-98 doi:10.1016/j.brainres.2018.10.031

274. Zong T, Yang Y, Zhao H, Li L, Liu M, Fu X, Tang G, Zhou H, Aung LHH, Li P, Wang J, Wang Z, Yu T (2021) tsRNAs: Novel small molecules from cell function and regulatory mechanism to therapeutic targets. Cell Prolif 54:e12977 doi:10.1111/cpr.12977

275. Zong X, Nakagawa S, Freier SM, Fei J, Ha T, Prasanth SG, Prasanth KV (2016) Natural antisense RNA promotes 3' end processing and maturation of MALAT1 lncRNA. Nucleic Acids Res 44:2898-2908 doi:10.1093/nar/gkw047
